# Author Correction: TriQuinoline

**DOI:** 10.1038/s41467-019-13860-5

**Published:** 2020-01-21

**Authors:** Shinya Adachi, Masakatsu Shibasaki, Naoya Kumagai

**Affiliations:** Institute of Microbial Chemistry, 3-14-23 Kamiosaki, Shinagawa-ku, Tokyo, 141-0021 Japan

**Keywords:** Chemical synthesis, Organic chemistry, Supramolecular chemistry

Correction to: *Nature Communications* 10.1038/s41467-019-11818-1, published online 23 August 2019.

The original version of this article contained an error in the third paragraph of the ‘Physicochemical properties of TQ’ section of the Results, which incorrectly read ‘The electron density *ρ*(*r*_c_), the Laplacian of the electron density (∇^2^*ρ*(*r*_c_)), and the ratio of the curvature of the density at the BCPs (|*λ*_1_|/*λ*_3_) have been proposed as the principal descriptors to characterise CH–π interactions, and typically fall in the ranges of 0.002 < *ρ*(*r*_c_) < 0.034, 0.02 < ∇^2^*ρ*(*r*_c_) < 0.14, and |*λ*_1_|/*λ*_3_ > 1, respectively. Among the 15 intermolecular BCPs found, 12 of these involve the protons at the 4-, 5-, and 6-positions of each quinoline unit that are geometrically close to the π-plane of [12]CPP and satisfy the aforementioned criteria, which strongly suggests that CH–π interactions play a dominant role in the formation of inclusion complex 15 (Fig. 6a)’. The correct version states ‘0.002 < *ρ*(*r*_c_) < 0.0034’ in place of ‘0.002 < *ρ*(*r*_c_) < 0.034’, ‘|*λ*_1_|/*λ*_3_ < 1’ in place of ‘|*λ*_1_|/*λ*_3_ > 1’, and ‘9 of these’ in place of ‘12 of these’.

This has been corrected in both the PDF and HTML versions of the article.

