# Supplementary material for: TriQuinoline
Source: Nat Commun. 2019 Aug 23;10:3820. doi: 10.1038/s41467-019-11818-1 (PMC6707140; doi:10.1038/s41467-019-11818-1)
Supplement: Supplementary file 1 — Supplementary Information [file 41467_2019_11818_MOESM1_ESM.pdf]

TriQuinoline

Shinya Adachi, Masakatsu Shibasaki, Naoya Kumagai*

Institute of Microbial Chemistry (BIKAKEN), Tokyo, Japan

nkumagai@bikaken.or.jp

Supplementary Methods

1. General Methods 3

1-1. Reactions and purifications. 3
1-2. Characterizations. 3
1-3. Solvents and reagents. 3
1-4. Computational details. 3

2. General Procedures 4

2-1. Synthesis of ***S1****. 4*
2-2. Synthesis of ***10****. 6*
2-3. Synthesis of 2-hydroxylethyl-TQ•TFA ***12****. 9*
2-4. Reaction of DQ-Im·TFA ***9*** *with TBS ketene acetal. 9*
2-5. Synthesis of ***13****●TFA. 10*
2-6. Synthesis of ***S5****●TFA. 10*

3. Crystal Structures 12

3-1. Crystal structure of compound ***6****. 12*
3-2. Crystal structure of compound ***S4****. 13*

4. ^{1}H NMR Monitoring of Triquinoline Formation 14

4-1. Reaction of DQ-Im●TFA ***9*** *with butyl vinyl ether (BVE). 14*
4-2. Amine formation via the reduction of DQ-Im●TFA ***9*** *and oxidation of DQ-Am●TFA* ***11*** *into DQ-Im●TFA* ***9*** *under air. 15*
4-3. Reaction of DQ-Im●TFA ***9*** *with butyl vinyl ether (BVE) in a glove box. 17*
4-4. Reaction of DQ-Im●TFA ***9*** *with acetaldehyde. 19*

5. Measurement of H^+/D^+ Exchange Rate 21

5-1. H^+/D^+ exchange rate in TQ●TFA ***10*** *in the absence of TFA. 21*
5-2. H^+/D^+ exchange rate in TQ●TFA ***10*** *in the presence of 3.3 ppm of CF_3COOD. 23*
5-2. H^+/D^+ exchange rate in TQ●TFA ***10*** *in the presence of 6.7 ppm of CF_3COOD. 25*
5-3. H^+/D^+ exchange rate in TQ●TFA ***10*** *in the presence of 10 ppm of CF_3COOD. 27*
5-4. H^+/D^+ exchange rate in ***13****●TFA. 29*
5-5. H^+/D^+ exchange rate in DQ-Im·TFA ***9*** *or* ***S5****●TFA. 30*

6. ESI-TOF-MS Spectra of Supramolecular Complexes 32

7. Determination of Association Constants from Titration Experiments 33

7-1. Complexation of TQ●TFA ***10*** *and [12]CPP. 33*
7-2. Complexation of TQ●TFA ***10*** *and coronene. 34*

8. ^{1}H NMR Spectra of Mixtures Containing CPP 37

8-1. ^{1}H NMR spectrum of a mixture of DQ-Im●TFA ***9*** *and [12]CPP. 37*

*8-2. ^{1}H NMR spectrum of a mixture of **S5**•TFA and [12]CPP.* 38
*8-3. ^{1}H NMR spectrum of a mixture of 2-hydroxylethyl-TQ **12** and [12]CPP.* 39
*8-4. ^{1}H NMR spectrum of a mixture of TQ•TFA **10** and [10]CPP.* 40
8-5. ^{1}H NMR spectrum of a mixture of coronene and [12]CPP. 41

9. Fluorescence Quenching Experiment 42

*9-1. Addition of TQ•TFA **10** to [12]CPP.* 42
*9-2. Addition of DQ-Im·TFA **9** to [12]CPP.* 43
*9-3. Addition of 2,8'-biquinoline **S5**•TFA to [12]CPP.* 44
*9-4. Addition of 2-hydroxylethyl-TQ•TFA **12** to [12]CPP.* 45

10 Computational Study on Non-Stop Povarov Reaction of Diquinoline-Imine (DQ-Im) 46

10-1. General. 46
10-2. Reaction profile. 46
10-3. Formation of π-complex I and II. 47
10-4. Distortion/interation analysis along IRC of TS (hydride transfer). 48

11. Homodesmotic Reaction of TQ 49

12. Proton Affinity of TQ 50

13. Conformational Analysis of TQ•H$^+$ and DQ-Im•H$^+$ 50

14. DFT Calculations of Supramolecular Complexes of TQ 51

14-1. Inclusion complex TQ•H$^+$ and [12]CPP. 51
14-2. Complex of TQ•H$^+$ and coronene. 52
14-3. Complex of TQ•H$^+$, [12]CPP, and coronene. 53
14-4. DFT-GIAO calculations of supramolecular complexes. 54
14-5. SAPT calculation. 54
14-6 NCIPLOT of supramolecular complexes. 55

15. Topoisomerase I Assay 55

16. ^{1}H, ^{13}C, and ^{19}F NMR Spectra of New Compounds 57

17. Cartesian Coordinates of Optimized Structure 72

18. References 110

1. General Methods

1-1. Reactions and purifications.

Unless otherwise noted, all reactions were carried out in an oven-dried flask fitted with a septum under an argon atmosphere with magnetically stirred chips. All work-up and purification procedures were carried out with reagent-grade solvents under ambient atmosphere. Thin layer chromatography (TLC) was performed on Merck TLC plates (0.25 mm) with silica gel 60 F254 and visualized by UV quenching and staining with $KMnO_4$. Flash column chromatography was performed using SiO_2 [Kanto Chemical 60N (neutral, spherical, 50–60 μm)] or CombiFlash systems with a Redisep column.

1-2. Characterizations.

Infrared (IR) spectra were recorded on a HORIBA FT210 Fourier transform infrared spectrophotometer. NMR spectra were recorded on a Bruker AVANCE III HD400 or a JEOL ECZ-600R. Chemical shifts (δ) are given in ppm relative to residual solvent peaks.[1] Data for ^{1}H NMR are reported as follows: chemical shift (multiplicity, coupling constants where applicable, number of hydrogens). Abbreviations are as follows: s (singlet), d (doublet), t (triplet), dd (doublet of doublet), dt (doublet of triplet), ddd (doublet of doublet of doublet), q (quartet), m (multiplet), br (broad). For ^{19}F NMR, chemical shifts were reported in the scale relative to $PhCF_3$ (δ –62.7680 ppm in $CDCl_3$) as an external reference. High-resolution mass spectra were measured on a ThermoFisher Scientific LTQ Orbitrap XL. ESI-TOF-MS spectra of supramolecular complexes are measured on a Bruker micrOTOF-II implemented with a CryoSpray source.

1-3. Solvents and reagents.

THF, CH_2Cl_2, and CH_3CN were purified by passing through a solvent purification system (Glass Contour). All other materials were used as purchased from commercial suppliers or prepared by the method described in the corresponding references.

1-4. Computational details.

All quantum chemical calculations were performed using the Gaussian 16 program.* Density functional theory (DFT) calculations employed an ultrafine integral grid (99 radial shells, 590 angular points). Structural optimizations were conducted at the level of theory specified in the corresponding section. Frequency calculations confirmed the identity of geometry minima (no imaginary frequencies) and transitions states (one imaginary frequency). All transition state structures were verified to connect the reactant and the product of interest by performing IRC calculations. Zero-point energies and thermal corrections were obtained at 298K and are unscaled.

*Gaussian 16, Revision A.03, M. J. Frisch, G. W. Trucks, H. B. Schlegel, G. E. Scuseria, M. A. Robb, J. R. Cheeseman, G. Scalmani, V. Barone, G. A. Petersson, H. Nakatsuji, X. Li, M. Caricato, A. V. Marenich, J. Bloino, B. G. Janesko, R. Gomperts, B. Mennucci, H. P. Hratchian, J. V. Ortiz, A. F. Izmaylov, J. L. Sonnenberg, D. Williams-Young, F. Ding, F. Lipparini, F. Egidi, J. Goings, B. Peng, A. Petrone, T. Henderson, D. Ranasinghe, V. G. Zakrzewski, J. Gao, N. Rega, G. Zheng, W. Liang, M. Hada, M. Ehara, K. Toyota, R. Fukuda, J. Hasegawa, M. Ishida, T. Nakajima, Y. Honda, O. Kitao, H. Nakai, T. Vreven, K. Throssell, J. A. Montgomery, Jr., J. E. Peralta, F. Ogliaro, M. J. Bearpark, J. J. Heyd, E. N. Brothers, K. N. Kudin, V. N. Staroverov, T. A. Keith, R. Kobayashi, J. Normand, K. Raghavachari, A. P. Rendell, J. C. Burant, S. S. Iyengar, J. Tomasi, M. Cossi, J. M. Millam, M. Klene, C. Adamo, R. Cammi, J. W. Ochterski, R. L. Martin, K. Morokuma, O. Farkas, J. B. Foresman, and D. J. Fox, Gaussian, Inc., Wallingford CT, 2016. Gaussian 09; Gaussian, Inc.: Wallingford, CT.

2. General Procedures

*2-1. Synthesis of **S1**.*

8-Chloro-2,8'-biquinoline (**S1**)

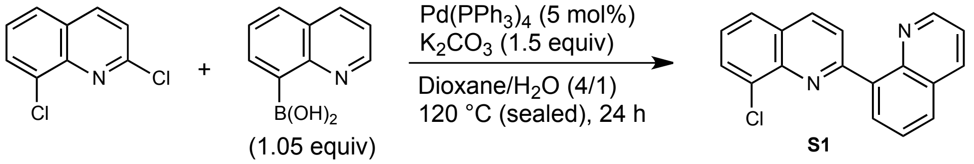

A pressure-proof glass vial capable of being sealed with a Teflon cap (for microwave experiments) was charged with 2,8-dichloroquinoline (230 mg, 1.16 mol,1.0 equiv), quinoline-8-boronic acid (211 mg, 1.22 mol, 1.1 equiv), K_2CO_3 (241 mg, 1.74 mmol, 1.5 equiv), and $Pd(PPh_3)_4$ (67.0 mg, 0.0580 mmol, 5.0 mol%) and was evacuated and back-filled with argon. 1,4-dioxane (4.0 mL) and H_2O (1.0 mL) was added, and the vial was sealed with a Teflon cap, evacuated, and back-filled with argon. The resulting reaction mixture was stirred at 120 °C for 24 h. The resulting mixture was diluted with EtOAc/H_2O and extracted three times with EtOAc. The combined organic layer was dried over Na_2SO_4, filtered, and concentrated. The residue was purified by flash column chromatography (*n*-hexane/EtOAc, 100/0 – 3/1) to **S1** (225 mg, 67% yield).

Pink solid; m.p.: 161–163 °C; ^{1}H NMR (400 MHz, 298 K, $CDCl_3$): δ 8.97 (dd, *J* = 4.2, 1.8 Hz, 1H), 8.43 (dd, *J* = 7.2, 1.5 Hz, 1H), 8.37 (d, *J* = 8.5 Hz, 1H), 8.25 (dd, *J* = 8.4, 1.5 Hz, 2H), 7.94 (dd, *J* = 8.2, 1.5 Hz, 1H), 7.85 (dd, *J* = 7.5, 1.3 Hz, 1H), 7.80 (dd, *J* = 8.2, 1.4 Hz, 1H), 7.74 (dd, *J* = 8.1, 7.2 Hz, 1H), 7.53–7.33 (m, 2H); ^{13}C NMR (100 MHz, 298 K, $CDCl_3$) δ; 158.7, 150.3, 146.1, 144.7, 138.7, 136.7, 135.2, 133.8, 132.5, 129.6, 129.5, 128.8, 128.7, 127.0, 126.8, 126.3, 126.1, 121.2; IR (KBr): $\tilde{\nu}$= 3042, 2996, 1601, 1497, 1425, 1292, 1281, 1183, 1066, 1011, 972, 824, 789, 759, 749, 669 cm^{-1}; HRMS (ESI) *m*/*z* calculated for $C_{18}H_{12}N_2Cl$ [M+H]$^+$: 291.0684, found: 291.0683.

8-Bromo-2,8':2',8''-terquinoline (**S2**) [2-3]

Note that the palladium catalyzed borylation/Suzuki–Miyaura cross-coupling reported by G. A. Molander, et al.[2-3] was utilized for the synthesis of compound **S2**.

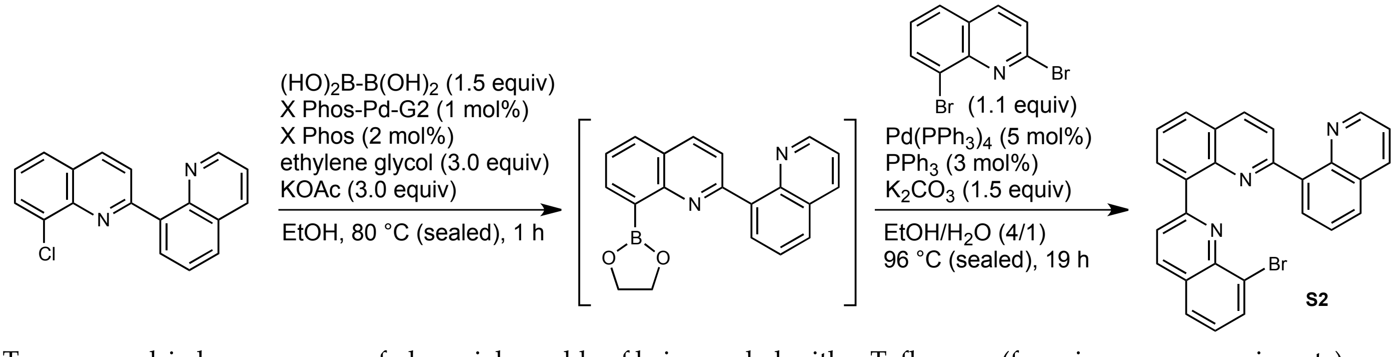

To an oven dried pressure-proof glass vial capable of being sealed with a Teflon cap (for microwave experiments) were added XPhos Pd G2 (12.2 mg, 0.0155 mmol, 1.0 mol%), XPhos (14.8 mg, 0.031 mmol, 2.0 mol%), tetrahydroxydiboron (208 mg, 2.32 mmol, 1.5 equiv), KOAc (456 mg, 4.64 mmol, 3.0 equiv), and 8-chloro-2,8'-biquinoline **S1** (450 mg, 1.54 mmol, 1.0 equiv). The vial was sealed with a Teflon cap, evacuated, and back-filled with argon (four times). EtOH (15.5 mL) was added via a syringe followed by the addition of ethylene glycol (259 μL 4.64 mmol, 3.0 equiv). The resulting mixture was stirred at 80 °C for 1 h. After the mixture was cooled to room temperature, the seal was removed. To the vial were added K_2CO_3 (320 mg, 2.32 mmol, 1.50 equiv), PPh_3 (12.2 mg, 0.0464 mmol, 3.0 mol%), and $Pd(PPh_3)_4$ (89.4 mg, 0.0773 mmol, 5.0 mol%). After the addition of H_2O (4.5 mL) via a syringe, and the mixture was left stand for 5 min. After subsequent addition of 2,8-diboromoquinoline[4] (489 mg, 1.70 mmol, 1.1 equiv), the vial was sealed, evacuated, and filled with argon (four times). The resulting mixture was stirred at 96 °C for 19 h. The resulting mixture was diluted with $CHCl_3$/H_2O and extracted three times with $CHCl_3$. The combined organic layer was dried over Na_2SO_4, filtered, and concentrated. The residue was purified by flash column chromatography (*n*-hexane/EtOAc, 100/0 – 2.3/1 – 0/100), and the resulting solids were recrystallized from *n*-hexane/EtOAc to give **S2** (364 mg, 51% yield).

White solid; m.p.: 201–203 °C; ^{1}H NMR (600 MHz, 298 K, $CDCl_3$): δ 9.11–8.90 (m, 1H), 8.70 (d, *J* = 8.6 Hz, 1H), 8.58 (dd, *J* = 7.2, 1.5 Hz, 1H), 8.48–8.30 (m, 2H), 8.24 (td, *J* = 8.4, 1.7 Hz, 2H), 8.13 (d, *J* = 8.6 Hz, 1H), 8.05 (dd, *J* = 7.4, 1.3 Hz, 1H), 8.01 (dd, *J* = 8.1, 1.5 Hz, 1H), 7.91 (dd, *J* = 8.1, 1.5 Hz, 1H), 7.80 (dd, *J* = 8.1, 1.3 Hz, 1H), 7.76 (t, *J* = 7.6 Hz, 1H), 7.65 (t, *J* = 7.6 Hz, 1H), 7.47 (dd, *J* = 8.3, 4.1 Hz, 1H), 7.38 (t, *J* = 7.7 Hz, 1H); ^{13}C NMR (150 MHz, 298 K, $CDCl_3$) δ 159.1, 157.6, 150.4, 146.2 (2C), 145.4, 139.3, 138.5, 136.6, 135.5, 134.8, 132.8, 132.6, 132.2, 129.5, 129.3, 128.83, 128.78, 127.9, 127.5, 126.92, 126.86, 126.7, 126.6, 125.5, 125.1, 121.3; IR (KBr): $\tilde{\nu}$ = 3042, 1600, 1558, 1499, 1418, 1278, 959, 822, 789, 758, 748, 656 cm^{-1}; HRMS (ESI) *m/z* calculated for $C_{27}H_{17}N_3Br$ [M+H]$^+$: 462.0600, found: 462.0596.

TQ•HCO_2H (**S3**)

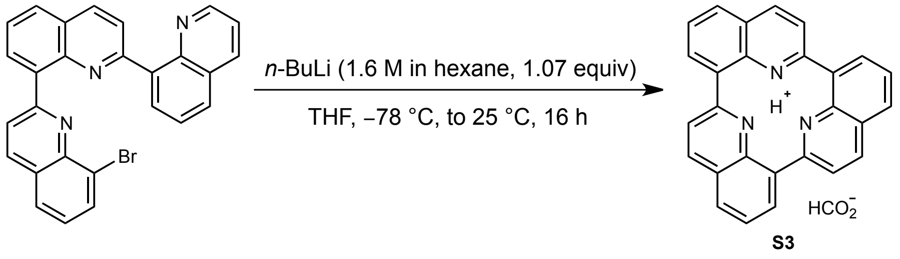

To a stirred THF solution (2.0 mL) of 8-bromo-2,8':2',8''-terquinoline **S2** (46.2 mg, 0.100 mmol, 1.0 equiv) at –78 °C was slowly added *n*-BuLi (1.606 M in hexane, 66.6 μL, 0.107 mmol, 1.07 equiv). The reaction mixture was allowed to warm to room temperature and stirred at this temperature for 16 h. The resulting mixture was diluted with water and washed with DCM (8 times). The aqueous layer was lyophilized to give the crude product. Purification was performed by reverse phase preparative HPLC [A: 0.1% HCO_2H in H_2O, B: 0.1% HCO_2H in CH_3CN] using Shiseido CAPCELL PAK MGII C18 column (*ϕ* 20 mm x 250 mm) with a gradient of [A 75%/B 25%] to [A 5%/B 95%] in 35 min. The fractions corresponding to the peak at t_R = 12.8 min were collected and lyophilized to give **S3** (2.19 mg, 5.7% yield).

The ^{1}H NMR spectrum of 1:1 mixture of TQ•HCO_2H (530 μL of 0.8 mM in CD_3CN) and TQ•TFA (530 μL of 0.8

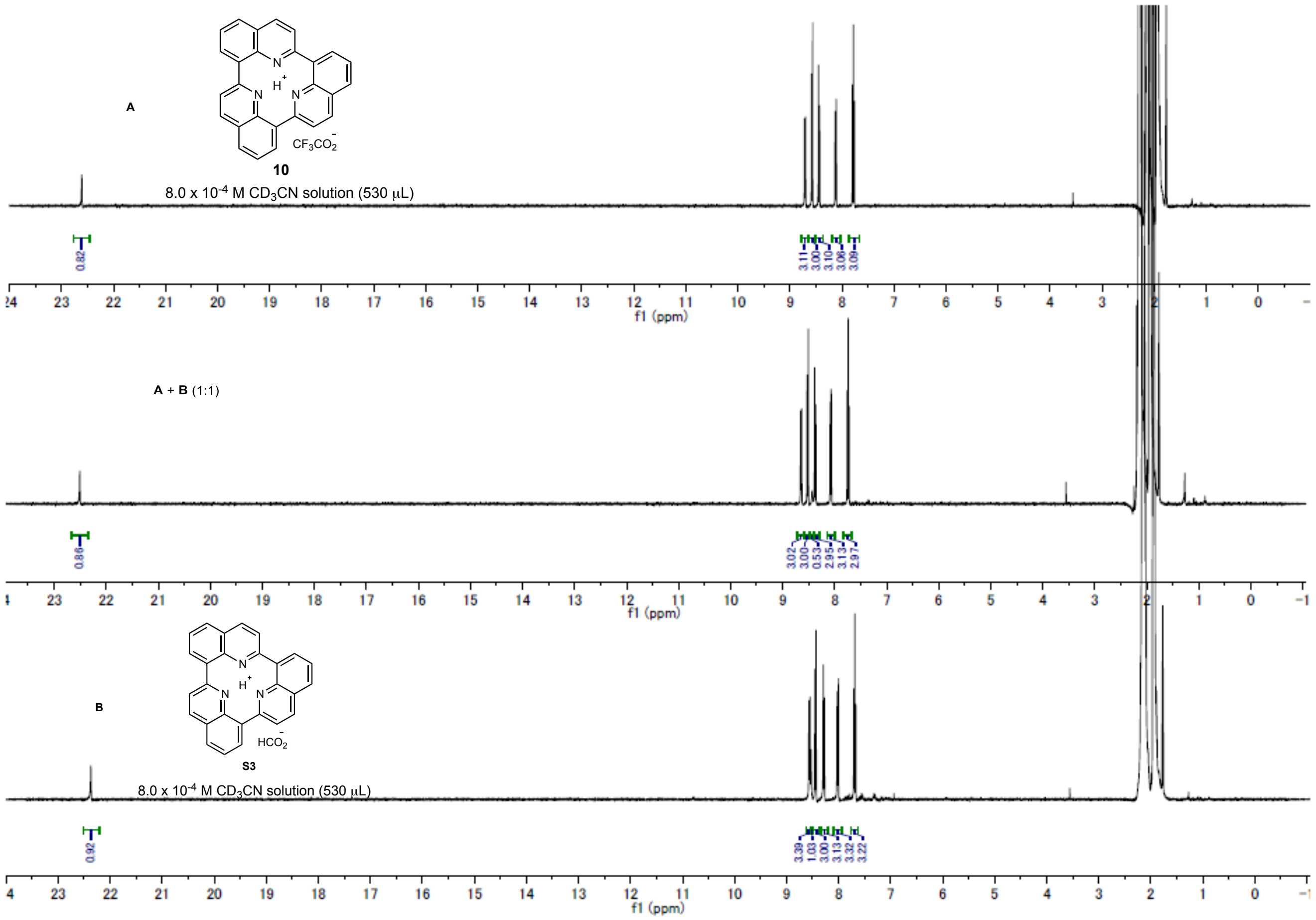

Supplementary Fig. 1 Comparison of ^{1}H NMR spectra (–1 ppm to 24 ppm) of TQ•TFA synthesized from DQ-Im•TFA **9** (A, top), TQ•HCO_2H **S3** synthesized from **S2** (B, bottom), and 1:1 mixture of A and B (middle).

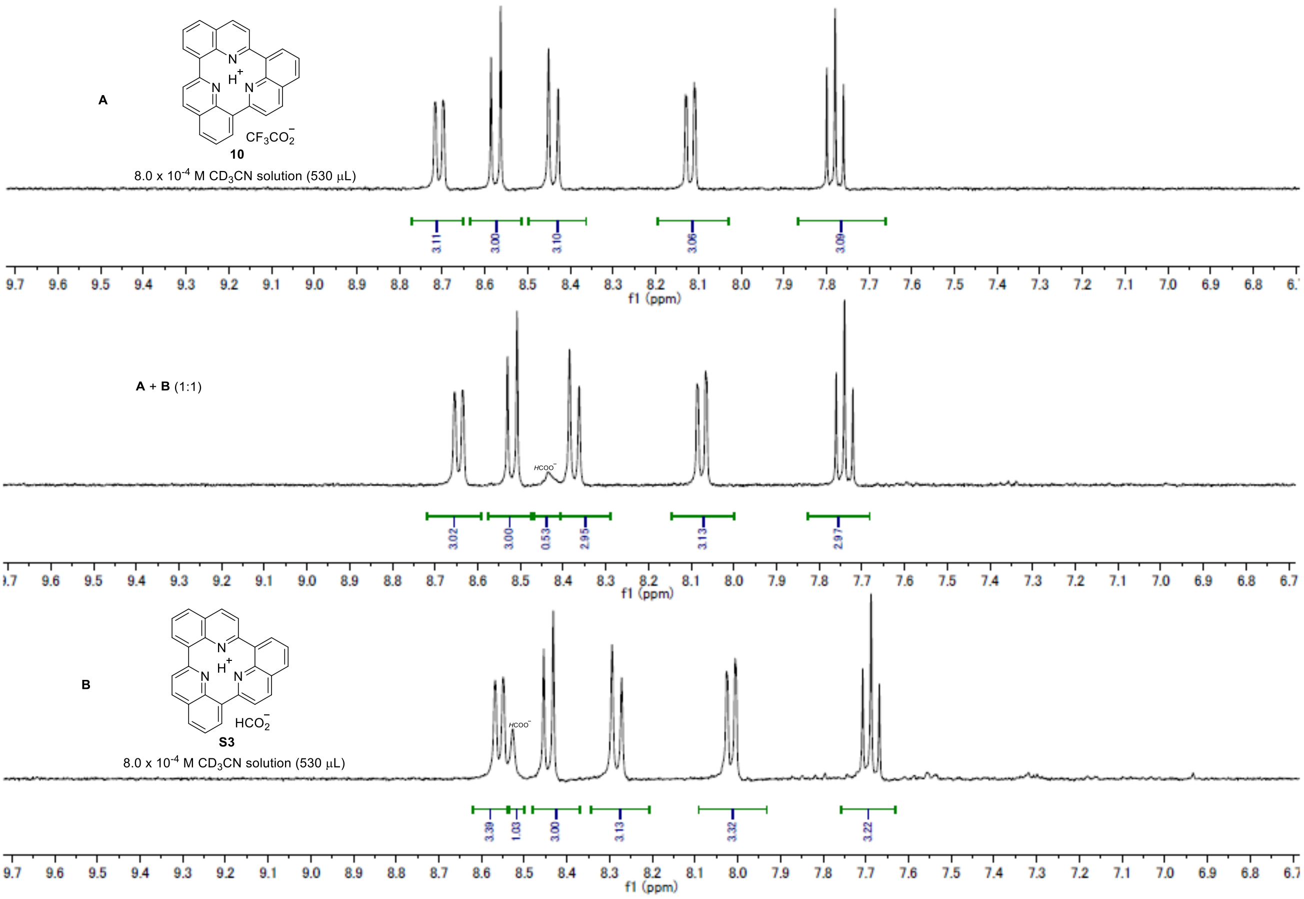

Supplementary Fig. 2 Comparison of ^{1}H NMR spectra (6.7 ppm to 9.7 ppm) of TQ•TFA synthesized from DQ-Im•TFA **9** (A, top), TQ•HCO_2H **S3** synthesized from **S2** (B, bottom), and 1:1 mixture of A and B (middle).

mM in CD_3CN) (the latter was obtained from the more reliable and reproducible protocol as outlined in Fig 2 in the main text), showed five sets of 3H peaks corresponding to protons of TQ and 0.5H of HCO_2H (Supplementary Fig. 1,2). This result indicates that both TQ•TFA and TQ•HCO_2H share the identical core structure of TQ•H^+.

2-2. Synthesis of **10**.

***tert*-Butyl (2-(8-chloroquinolin-2-yl)phenyl)carbamate** (3)

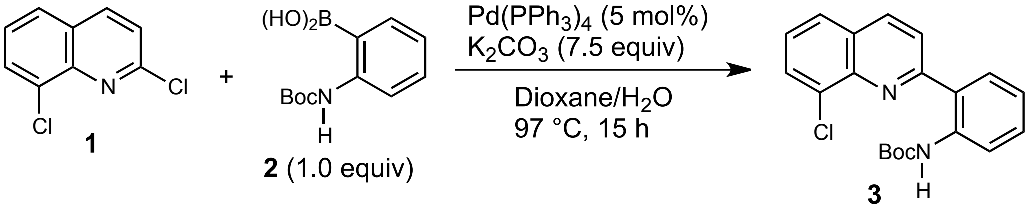

A pressure-proof glass vial capable of being sealed with a Teflon cap (for microwave experiments) was charged with 2,8-dichloroquinoline **1** (668 mg, 3.37 mol, 1.0 equiv), 2-(*t*-butoxycarbonylamino)phenylboronic acid **2** (800 mg, 3.37 mol, 1.0 equiv), K_2CO_3 (3.50 g, 25.3 mmol, 7.5 equiv), and $Pd(PPh_3)_4$ (204 mg, 0.176 mmol, 5.0 mol%) and was evacuated and back-filled with argon. To the mixture, 1,4-dioxane (12.7 mL) and H_2O (5.9 mL) were added at 0 °C, and the vial was evacuated, back-filled with argon for several times, and sealed. The resulting mixture was stirred at 85 °C for 15 h. The resulting mixture was diluted with EtOAc/H_2O and extracted three times with EtOAc. The combined organic layer was dried over Na_2SO_4, filtered, and concentrated. The residue was purified by flash column chromatography (*n*-hexane/EtOAc, 100/0 – 5.7/1) to give **3** (589 mg, 49% yield).

Orange solid; m.p.: 151–153 °C; ^{1}H NMR (600 MHz, 300 K, CD_3CN): δ 12.39 (s, 1H), 8.46 (dd, J = 8.6, 5.1 Hz, 2H), 8.14 (d, J = 8.8 Hz, 1H), 8.03 (dd, J = 8.0, 1.5 Hz, 1H), 7.93 (dd, J = 11.8, 7.8 Hz, 2H), 7.57 (t, J = 7.8 Hz, 1H), 7.48 (dd, J = 8.5, 7.2 Hz, 1H), 7.24–7.14 (m, 1H), 1.51 (s, 9H); ^{13}C NMR (150 MHz, 300 K, CD_3CN) δ 159.5, 154.3, 143.1, 140.6, 139.2, 133.2, 131.8, 131.1, 130.8, 128.9, 128.01, 127.96, 124.5, 123.1, 122.3, 120.9, 80.5, 28.6; IR (KBr): $\tilde{\nu}$= 3435, 3180, 2968, 1724, 1605,

1579, 1521, 1454, 1244, 1231, 1158, 1048, 1023, 838, 763, 756 cm^{-1}; HRMS (ESI) *m/z* calculated for $C_{20}H_{19}O_2N_2ClNa$ [M+Na]$^+$: 377.1027, found: 377.1027.

tert-Butyl (2-(8-methyl-[2,8'-biquinolin]-2'-yl)phenyl)carbamate (6)

*Note that the palladium catalyzed borylation/Suzuki–Miyaura cross-coupling reported by G. A. Molander, et al.[2-3] was utilized for the synthesis of compound **6**.*

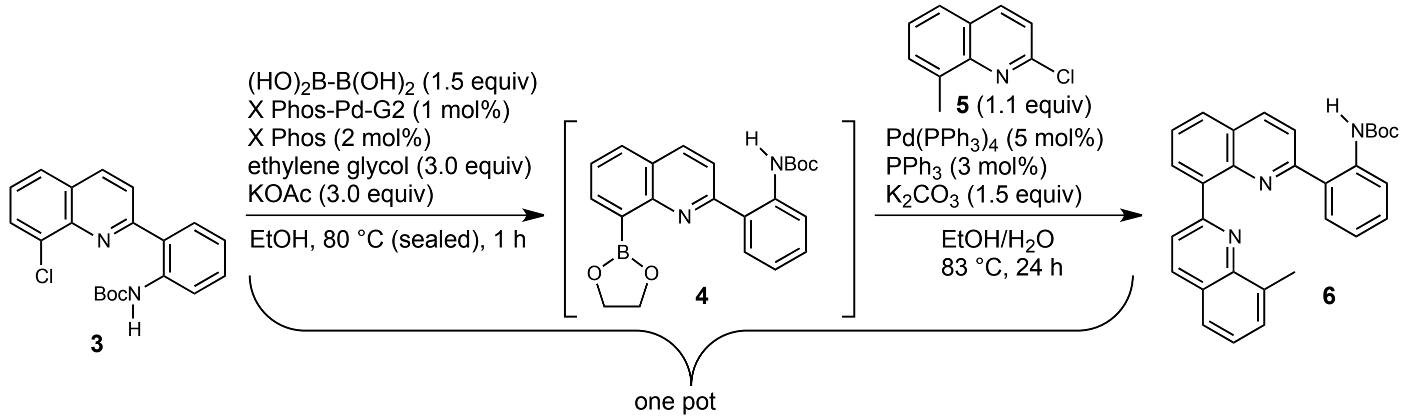

To an oven dried pressure-proof glass vial capable of being sealed with a Teflon cap (for microwave experiments) were added XPhos Pd G2 (9.97 mg, 0.0127 mmol, 1.0 mol%), XPhos (12.1 mg, 0.0253 mmol, 2.0 mol%), tetrahydroxydiboron (170 mg, 1.90 mmol, 1.5 equiv), KOAc (373 mg, 3.80 mmol, 3.0 equiv), and *tert*-butyl (2-(8-chloroquinolin-2-yl)phenyl)carbamate **3** (450 mg, 1.27 mmol, 1.0 equiv). The vial was sealed, evacuated, and filled with argon (four times). EtOH (12.7 mL) was added at 0 °C via syringe followed by the addition of ethylene glycol (212 μL 3.80 mmol, 3.0 equiv). The resulting mixture was stirred at 80 °C for 1 h. After cooling the mixture to room temperature and removing the seal, K_2CO_3 (263 mg, 1.90 mmol, 1.50 equiv), PPh_3 (9.97 mg, 0.0380 mmol, 3.0 mol%), and $Pd(PPh_3)_4$ (73.2 mg, 0.0633 mmol, 5.0 mol%) were added. After adding H_2O (3.68 mL) via a syringe, the mixture was left stand for 5 min. After subsequent addition of 2-chloro-8-methylquinoline **5** (248 mg, 1.39 mmol, 1.1 equiv), the vial was sealed, evacuated, and back-filled with argon (four times). The resulting mixture was stirred at 83 °C for 24 h. The resulting mixture was diluted with EtOAc/H_2O and extracted three times with EtOAc. The organic layer was dried over Na_2SO_4, filtered, and concentrated. The residue was purified by flash column chromatography (*n*-hexane/EtOAc, 100/0 – 3/1) to give **6** (385mg, 66% yield).

Pale orange solid; m.p.: 225–227 °C; ^{1}H NMR (400 MHz, 298 K, d_6-acetone): δ 10.91 (s, 1H), 8.65 (d, *J* = 8.8 Hz, 1H), 8.50 (dd, *J* = 7.2, 1.5 Hz, 1H), 8.40 (d, *J* = 8.6 Hz, 1H), 8.36 (d, *J* = 8.6 Hz, 1H), 8.31 (dd, *J* = 8.4, 1.2 Hz, 1H), 8.17 (dd, *J* = 8.1, 1.5 Hz, 1H), 8.08 (d, *J* = 8.7 Hz, 1H), 7.94–7.80 (m, 3H), 7.68–7.61 (m, 1H), 7.52 (dd, *J* = 8.2, 7.0 Hz, 1H), 7.47–7.39 (m, 1H), 7.20 (ddd, *J* = 7.9, 7.3, 1.3 Hz, 1H), 2.85 (s, 3H), 0.84 (s, 9H); ^{13}C NMR (100 MHz, 298 K, d_6-acetone) δ 159.0, 156.7, 153.5, 148.7, 144.8, 139.64, 139.57, 139.1, 138.4, 136.1, 134.0, 131.1, 130.9, 130.1, 130.0, 128.4, 128.0, 127.9, 127.2, 127.0, 126.6, 125.3, 123.2, 122.4, 121.1, 79.5, 27.8, 18.2; IR (KBr): $\tilde{\nu}$ = 3437, 3219, 2975, 1715, 1605, 1583, 1521, 1507, 1449, 1422, 1243, 1160, 1046, 1023, 835, 764 cm^{-1}; HRMS (ESI) *m/z* calculated for $C_{30}H_{28}O_2N_3$ [M+H]$^+$: 462.2176, found: 462.2170.

tert-Butyl (2-(8-(dibromomethyl)-[2,8'-biquinolin]-2'-yl)phenyl)carbamate (8)

*Note that the compound **8** was synthesized with reference to the procedure for the synthesis of quinoline-8-caraldehyde reported by G. Dong, et al.[5]*

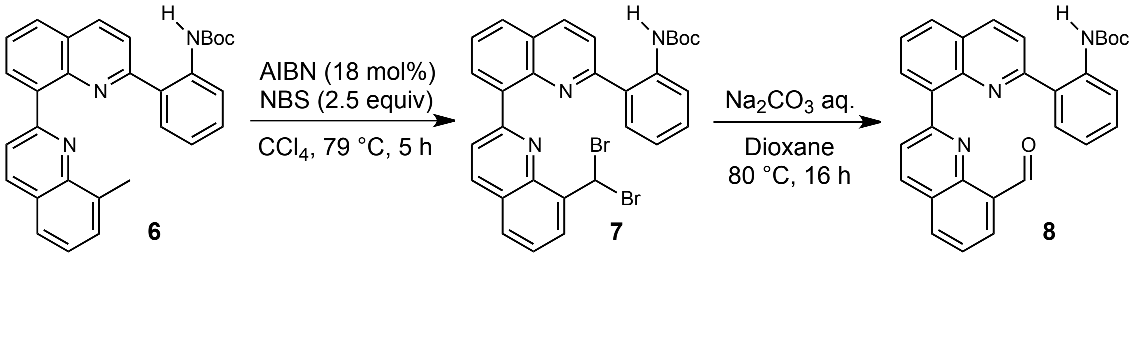

To a pressure-proof glass vial capable of being sealed with a Teflon cap (for microwave experiments) were added *tert*-butyl (2-(8-methyl-[2,8'-biquinolin]-2'-yl)phenyl)carbamate **6** (40.5 mg, 0.0877 mmol, 1.0 equiv), *N*-bromosuccinimide (38.7 mg, 0.218 mmol, 2.5 equiv), azobisisobutyronitrile (AIBN, 2.55 mg, 0.0155 mmol, 0.18 equiv), and carbon tetrachloride (2.0 mL). The vial was flushed with argon and sealed. The resulting mixture was stirred at 77 °C for 5 h. The resulting mixture was dissolved in CH_2Cl_2 and subsequently washed with 1N NaOH (aq.), H_2O, and brine. The organic layer was dried over Na_2SO_4, filtered, and concentrated to afford almost pure crude *tert*-butyl (2-(8-(dibromomethyl)-[2,8'-biquinolin]-2'-yl)phenyl)carbamate **7**, which was used in the next reaction without further purification. To the solution of crude **7** in degassed dioxane (9.0 mL) in a round-bottomed flask, degassed 10% Na_2CO_3 aq. (10.0 mL) was added. The flask was then equipped with a reflux condenser fitted with a three-way stopcock opened to an argon-filled balloon. The resulting mixture was stirred at 80 °C for 16 h under argon. The reaction mixture was diluted with EtOAc/H_2O and extracted three times with EtOAc. The combined organic layer was dried over Na_2SO_4, filtered, and concentrated to afford almost pure crude *tert*-butyl (2-(8-(dibromomethyl)-[2,8'-biquinolin]-2'-yl)phenyl)carbamate **8**, which was used in the next reaction without further purification. An analytically pure sample was obtained after purification by flash column chromatography (*n*-hexane/EtOAc, 4/1) and characterized as follows.

Pale yellow solid; m.p.: 191–193 °C; ^{1}H NMR (400 MHz, 298 K, CD_3CN): 11.55 (s, 1H), 10.78 (s, 1H), 8.56 (dt, *J* = 8.8, 2.7 Hz, 1H), 8.51–8.40 (m, 3H), 8.30 (ddt, *J* = 13.6, 7.3, 1.7 Hz, 2H), 8.22–8.08 (m, 2H), 8.00 (dt, *J* = 8.8, 2.3 Hz, 1H), 7.93–7.68 (m, 3H), 7.43 (ddd, *J* = 8.6, 7.3, 1.5 Hz, 1H), 7.28–7.12 (m, 1H), 0.81 (s, 9H); ^{13}C NMR (100 MHz, 298 K, CD_3CN) δ 193.5, 159.3, 159.2, 153.7, 148.9, 144.7, 139.6, 138.9, 138.7, 136.5, 135.4, 134.0, 132.8, 131.3, 131.2, 130.8, 129.5, 128.7, 128.0 (2C), 127.30, 127.26, 126.5, 123.5, 122.7, 121.3, 79.8, 27.8; IR (KBr): $\tilde{\nu}$= 3446, 2971, 2925, 1721, 1688, 1607, 1582, 1557, 1520, 1508, 1447, 1426, 1243, 1158, 1045, 1021, 839, 766 cm^{-1}; HRMS (ESI) *m*/*z* calculated for $C_{30}H_{26}O_3N_3$ [M+H]$^+$: 476.1969, found: 476.1965.

DQ-Im•TFA (9)

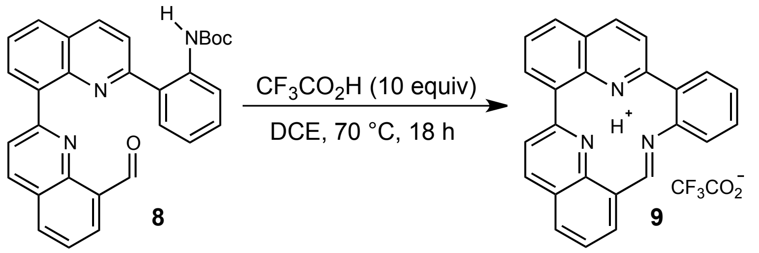

To a round-bottomed flask containing the crude *tert*-butyl (2-(8-(dibromomethyl)-[2,8'-biquinolin]-2'-yl)phenyl)carbamate **8** were added 1,2-dichloroethane (7.42 mL) and trifluoroacetic acid (TFA, 67.2 μL, 0.877 mmol, 10 equiv). The flask was then equipped with a reflux condenser fitted with a three-way stopcock opened to an argon-filled balloon. The reaction mixture was stirred at 70 °C for 18 h under argon. The resulting mixture was concentrated and dissolved in water (16.4 mL). The aqueous solution was lyophilized to afford almost pure crude DQ-Im•TFA **9**, which was used in the next reaction without further purification. An analytically pure sample was obtained by swirling powdered crude **9** in 1,2-dichloroethane followed by the collection of remaining solid material and characterized as follows.

Red solid; m.p.: 215–217 °C (decomp.); ^{1}H NMR (400 MHz, 300 K, CD_3CN): 20.88 (s, 1H), 8.39 (s, 1H), 8.31 (dd, *J* = 9.0, 5.8 Hz, 1H), 8.24–8.15 (m, 1H), 8.07 (d, *J* = 8.8 Hz, 1H), 8.02–7.94 (m, 1H), 7.88 (dt, *J* = 8.5, 2.0 Hz, 1H), 7.83– 7.71 (m, 3H), 7.63 (dd, *J* = 7.2, 1.5 Hz, 1H), 7.52–7.39 (m, 3H), 7.31 (ddd, *J* = 8.2, 7.2, 1.2 Hz, 1H), 7.20 (dd, *J* = 8.1, 1.4 Hz, 1H); ^{13}C NMR (150 MHz, 298 K, CD_3OD) δ 159.7, 155.4, 154.0, 146.9, 144.9, 142.0, 140.7, 140.0, 138.7, 135.9, 135.6, 134.4, 134.1, 131.7, 130.6, 130.1, 129.8, 129.4, 129.3, 128.5, 125.4, 124.9, 121.1, 119.6, 118.4; ^{19}F NMR (376 MHz, 298 K, CD_3OD): δ –77.2 (s); IR (KBr): $\tilde{\nu}$= 3443, 1683, 1625, 1593, 1203, 1186, 1129, 843, 800, 755, 723 cm^{-1}; HRMS (ESI) *m*/*z* calculated for $C_{25}H_{16}N_3$ [M+H]$^+$: 358.1339, found: 358.1336.

TQ•TFA (10)

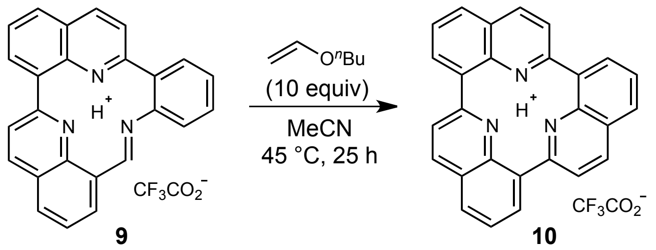

To a round-bottomed flask containing the crude DQ-Im•TFA **9** were added CH_3CN (8.35 mL) and butyl vinyl ether (BVE, 114 μL, 0.877 mmol, 10 equiv). The reaction mixture was stirred at 45 °C for 25 h. The resulting mixture was concentrated and dissolved in water, and washed with DCM (eight times). The aqueous layer was lyophilized to afford **10** in pure form (19.9 mg, 46% yield over 4 steps from compound **6**)

Brown solid; 207–209 °C (decomp.); ^{1}H NMR (400 MHz, 298 K, CD_3OD): δ 20.20 (s, 1H, this proton located at the center of TQ, which is subject to the proton exchange in CD_3OD, but observable for a week at room temperature), 7.33 (d, J = 8.3 Hz, 6H), 7.08 (d, J = 7.6 Hz, 3H), 7.02 (d, J = 9.2 Hz, 3H), 6.91–6.66 (m, 3H); ^{13}C NMR (150 MHz, 298 K, CD_3OD) δ 153.1, 140.9, 140.3, 134.1, 133.9, 128.7, 128.0, 124.9, 119.0; ^{19}F NMR (376 MHz, 298 K, CD_3OD): δ –76.6 (s); IR (KBr): $\tilde{\nu}$ = 3427, 3081, 2922, 1686, 1618, 1597, 1566, 1506, 1423, 1289, 1200, 1172, 1121, 837, 800, 753, 719 cm^{-1}; HRMS (ESI) *m*/*z* calculated for $C_{27}H_{16}N_3$ [M+H]$^+$: 382.1339, found: 382.1335.

2-3. Synthesis of 2-hydroxylethyl-TQ•TFA ***12***.

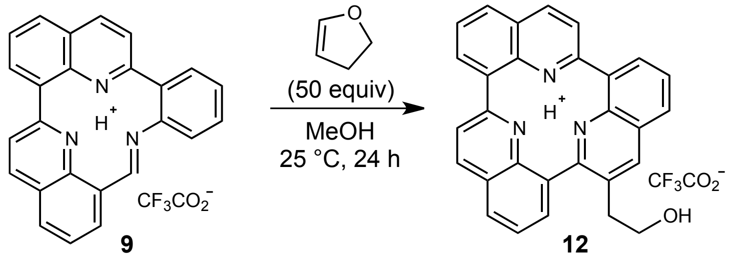

2-Hydroxylethyl-TQ•TFA (12)

To a round-bottomed flask were added DQ-Im•TFA **9** (7.9 mg, 0.0168 mmol, 1.0 equiv), MeOH (1.93 mL), and 2,3-dihydrofuran (63.6 μL, 0.840 mmol, 50 equiv). The reaction mixture was stirred at 25 °C for 24 h. After removal of the volatiles, the resulting residue was dissolved in water and washed with CH_2Cl_2 (eight times). The aqueous layer was lyophilized. Purification was performed by reverse phase preparative HPLC [A: 0.1% TFA in H_2O, B: 0.1% TFA in CH_3CN] using a Shiseido CAPCELL PAK MGII C18 column (ϕ 20 mm x 250 mm) with a gradient of [A 75%/B 25%] to [A 5%/B 95%] in 35 min. The fractions corresponding to the peak at t_R = 13.6 min were collected and lyophilized to give the **12** (3.1 mg, 34% yield).

Yellow amorphous solid; ^{1}H NMR (400 MHz, 300 K, CD_3CN): δ 21.48 (s, 1H), 8.56 (s, 1H), 8.49 (d, J = 8.5 Hz, 2H), 8.37 (ddd, J = 14.7, 12.5, 8.2 Hz, 3H), 8.22 (dd, J = 9.0, 4.3 Hz, 2H), 8.00 (ddd, J = 12.3, 8.1, 1.3 Hz, 2H), 7.87 (d, J = 8.0 Hz, 1H), 7.72 (t, J = 7.8 Hz, 1H), 7.59 (dt, J = 19.6, 7.8 Hz, 2H), 3.98 (t, J = 6.3 Hz, 2H), 3.33 (t, J = 6.3 Hz, 2H); ^{13}C NMR (150 MHz, 298 K, CD_3OD) δ 156.5, 154.8, 154.6, 142.9, 142.62, 142.60, 141.8, 141.54, 141.51, 138.0, 135.0, 134.34, 134.29, 134.2, 133.6, 132.7, 129.8, 129.5, 129.3, 129.1 (2C), 129.0, 128.8, 127.0, 125.7, 120.6, 120.5, 62.8, 37.4; ^{19}F NMR (376 MHz, 298 K, CD_3OD): δ –76.9 (s); IR (KBr): $\tilde{\nu}$= 3435, 3127, 1681, 1398, 1211, 1182, 1129, 840, 802, 724 cm^{-1}; HRMS (ESI) *m*/*z* calculated for $C_{29}H_{20}ON_3$ [M+H]$^+$: 426.1601, found: 426.1598.

2-4. Reaction of DQ-Im •TFA ***9*** *with TBS ketene acetal.*

Ketene acetal adduct (S4)

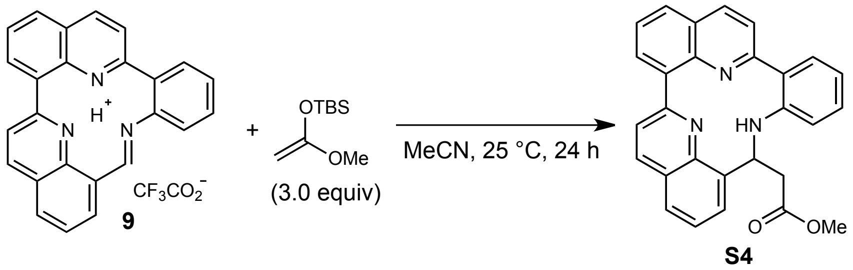

To a flask were added DQ-Im•TFA **9** (3.0 mg, 0.00636 mmol, 1.0 equiv), CH_3CN (0.50 mL), and *tert*-butyl((1-methoxyvinyl)oxy)dimethylsilane (3.6 mg, 0.0191 mmol). The reaction mixture was stirred at 25 °C for 24 h. After removal of volatiles, the resulting residue was purified by flash column chromatography (*n*-hexane/EtOAc, 100/0 – 4/1) to give **S4** (2.2 mg, 81% yield).

Yellow solid; m.p.: 206–208 °C; ^{1}H NMR (400 MHz, 298 K, CD_3CN) δ 13.82 (d, *J* = 10.1 Hz, 1H), 8.53 (dd, *J* = 7.4, 1.4 Hz, 1H), 8.49 (d, *J* = 8.7 Hz, 1H), 8.42 (d, *J* = 8.9 Hz, 1H), 8.20 (dd, *J* = 10.1, 8.9 Hz, 2H), 8.09 (ddd, *J* = 7.5, 5.9, 1.5 Hz, 2H), 7.95 (dd, *J* = 8.1, 1.4 Hz, 1H), 7.82 (dd, *J* = 7.1, 1.5 Hz, 1H), 7.78 (dd, *J* = 8.1, 7.3 Hz, 1H), 7.65 – 7.54 (m, 1H), 7.39 (dddd, *J* = 8.5, 7.0, 1.6, 0.6 Hz, 1H), 7.27 – 7.18 (m, 1H), 6.74 (ddd, *J* = 8.1, 7.0, 1.2 Hz, 1H), 5.86 – 5.67 (m, 1H), 3.28 (s, 3H), 3.04 – 2.78 (m, 2H); ^{13}C NMR (150 MHz, 298 K, CD_3CN): δ 172.5, 159.2, 155.4, 149.2, 146.1, 144.6, 140.3, 138.3, 137.7, 135.0, 132.2, 131.9, 130.9, 130.5, 130.4, 128.4, 128.3, 128.0, 127.4, 126.7, 121.9, 120.7, 120.4, 115.5, 112.5, 56.5, 51.8, 43.9; IR (KBr): $\tilde{\nu}$ = 3448, 2957, 2928, 1724, 1604, 1594, 1567, 1505, 1440, 1281, 1237, 1129, 835, 744; HRMS (ESI) *m*/*z* calculated for $C_{28}H_{22}O_2N_3$ [M+H]$^+$: 432.1707, found: 432.1703.

*2-5. Synthesis of **13**•TFA.*

1,8-Bis(dimethylamino)naphthalene•TFA (**13•TFA**)[6]

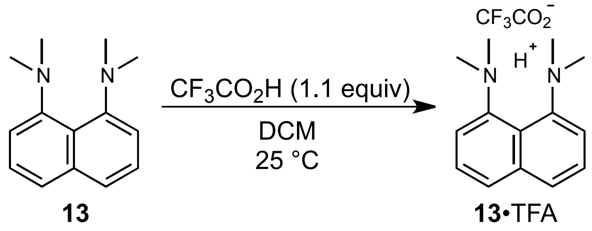

To a flask were added 1,8-bis(dimethylamino)naphthalene (**13**) (50.0 mg, 0.233 mmol, 1.0 equiv), CH_2Cl_2 (0.61 mL), and trifluoroacetic acid (19.7 μL, 0.257 mmol, 1.1 equiv). The reaction mixture was stirred for 10 min at 25 °C. The resulting mixture was concentrated and dried in vacuo to give **13**•TFA (76.1 mg, 99% yield).

White solid; m.p.: 153–155 °C; ^{1}H NMR (400 MHz, 298 K, CD_3CN): δ 18.63 (s, 1H), 8.04 (dd, *J* = 8.4, 1.0 Hz, 2H), 7.91 (dd, *J* = 7.6, 1.1 Hz, 2H), 7.70 (dd, *J* = 8.3, 7.6 Hz, 2H), 3.12 (d, *J* = 2.6 Hz, 12H); ^{13}C NMR (100 MHz, 298 K, CD_3OD) δ 145.6, 137.1, 130.6, 128.2, 122.7, 120.4, 46.7; ^{19}F NMR (376 MHz, 298 K, CD_3OD): δ –77.0 (s); IR (KBr): $\tilde{\nu}$ = 3456, 2317, 1685, 1472, 1193, 1129, 1030, 1009, 841, 802, 781, 712, 598 cm^{-1}; HRMS (ESI) *m*/*z* calculated for $C_{14}H_{19}N_2$ [M+H]$^+$: 215.1543, found: 215.1540.

*2-6. Synthesis of **S5**•TFA.*

2,8'-Biquinoline (S5)

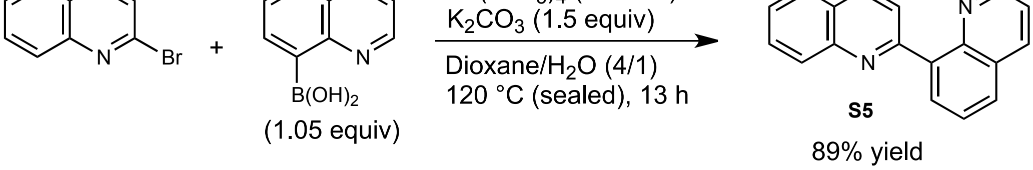

A pressure-proof glass vial capable of being sealed with a Teflon cap (for microwave experiments) was charged with 2-bromoquinoline (241 mg, 1.16 mol, 1.0 equiv), quinoline-8-boronic acid (211 mg, 1.22 mol, 1.1 equiv), K_2CO_3 (241 mg, 1.74 mmol, 1.5 equiv), and $Pd(PPh_3)_4$ (67.0 mg, 0.0580 mmol, 5.0 mol%) and was evacuated and back-filled with argon. After adding 1,4-dioxane (4.0 mL) and H_2O (1.0 mL), and the vial was evacuated and back-filled with argon (several times), and sealed. The resulting mixture was stirred at 120 °C for 14 h. After cooling to room temperature, reaction

mixture was diluted with EtOAc/H_2O and extracted with EtOAc (three times). The combined organic layer was dried over Na_2SO_4, filtered, and concentrated. The resulting residue was purified by flash column chromatography (*n*-hexane/EtOAc, 100/0 – 3/1) to give **S5** (264 mg, 89% yield)

White solid; m.p.: 122–124 °C; ^{1}H NMR (400 MHz, 298 K, $CDCl_3$): δ 8.96 (dd, *J* = 4.2, 1.8 Hz, 1H), 8.33–8.17 (m, 4H), 8.12 (d, *J* = 8.5 Hz, 1H), 7.94 (dd, *J* = 8.2, 1.6 Hz, 1H), 7.89 (dd, *J* = 8.1, 1.4 Hz, 1H), 7.80–7.64 (m, 2H), 7.56 (ddd, *J* = 8.1, 6.9, 1.2 Hz, 1H), 7.45 (dd, *J* = 8.3, 4.2 Hz, 1H); ^{13}C NMR (100 MHz, 298 K, $CDCl_3$) δ; 158.4, 150.5, 148.6, 146.2, 139.7, 136.5, 135.0, 131.6, 129.8, 129.4, 129.2, 128.8, 127.7, 127.5, 126.8, 126.5, 125.1, 121.3; IR (KBr): $\tilde{\nu}$ = 3437, 3037, 1601, 1497, 1417, 1390, 1296, 1187, 1125, 938, 839, 821, 794, 753 cm^{-1}; HRMS (ESI) *m*/*z* calculated for $C_{18}H_{13}N_2$ [M+H]$^+$: 257.1073, found: 257.1069.

2,8'-biquinoline•TFA (S5•TFA)

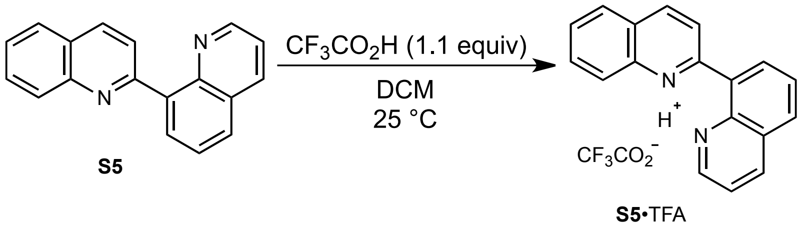

To a round-bottomed flask were added 2,8'-biquinoline (59.8 mg, 0.233 mmol, 1.0 equiv), CH_2Cl_2 (0.61 mL), and TFA (19.7 μL, 0.257 mmol, 1.1 equiv). The reaction mixture was stirred at 25 °C for 10 min. The resulting mixture was concentrated to give **S5**•TFA (86.0 mg, >99 % yield).

Yellow solid; m.p.: 43–45 °C; ^{1}H NMR (400 MHz, 298 K, $CDCl_3$): δ 11.92 (br s, 1H), 9.11 (dd, *J* = 4.4, 1.8 Hz, 1H), 8.61 (dd, *J* = 8.7, 0.8 Hz, 1H), 8.55 (dt, *J* = 8.6, 0.9 Hz, 1H), 8.47 (dd, *J* = 7.3, 1.4 Hz, 1H), 8.39 (dd, *J* = 8.3, 1.8 Hz, 1H), 8.35 (d, *J* = 8.7 Hz, 1H), 8.09 (dd, *J* = 8.3, 1.4 Hz, 1H), 8.02 (dd, *J* = 8.1, 1.3 Hz, 1H), 7.93 (ddd, *J* = 8.5, 7.0, 1.4 Hz, 1H), 7.84–7.69 (m, 2H), 7.61 (dd, *J* = 8.3, 4.4 Hz, 1H); ^{13}C NMR (100 MHz, 298 K, $CDCl_3$) δ; 155.8, 150.6, 144.7, 142.5, 140.6, 138.3, 133.3, 132.8, 132.0, 131.0, 129.0, 128.9, 128.0, 127.6, 127.2, 125.2, 124.2, 122.3; ^{19}F NMR (376 MHz, 298 K, $CDCl_3$): δ –75.8 (s); IR (KBr): $\tilde{\nu}$ = 3435, 1695, 1595, 1524, 1409, 1308, 1198, 1174, 1123, 829, 800, 760, 718 cm^{-1}; HRMS (ESI) *m*/*z* calculated for $C_{18}H_{13}N_2$ [M+H]$^+$: 257.1073, found: 257.1068.

3. Crystal Structures

*3-1. Crystal structure of compound **6.***

A crystal of **6** was obtained by vapor diffusion of hexane into an ethyl acetate solution of **6**. All measurements were made on a Rigaku XtaLAB P200 diffractometer using multi-layer mirror monochromated Cu-Kα radiation. All non-hydrogen atoms were refined anisotropically, and all hydrogenatoms were refined using the riding model. Refined structure and crystallographic parameters are summarized in Supplementary Fig. 3 and Supplementary Table 1. CCDC1909996 contains the supplementary crystallographic data for **6**.

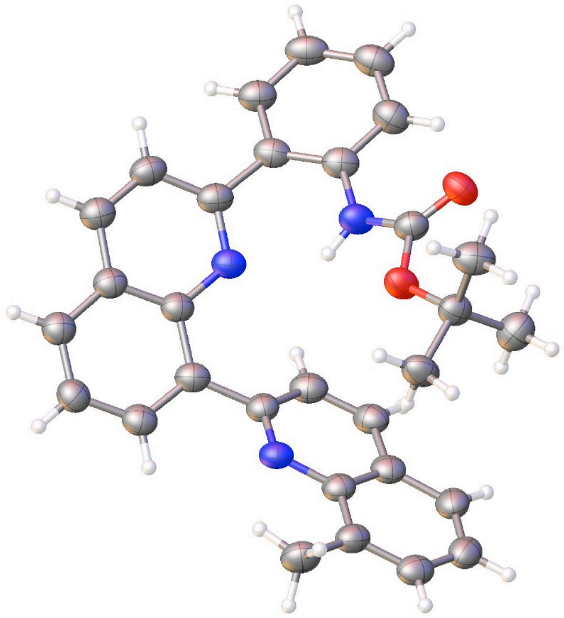

Supplementary Fig. 3 Structure of **6**. [ORTEP plot (50% ellipsoids)], Color code; gray: C, white: H, blue: N, red: O.

Supplementary Table 1. Selected crystal data of 6

Empirical Formula	$C_{30}H_{27}N_3O_2$
Formula Weight	461.56
Temperature/°C	–180.0
Crystal Color, Habit	colorless, prism
Crystal Dimensions	0.100 x 0.100 x 0.050 mm
Crystal System	monoclinic
Cell constants	
a	29.0781(11) Å
b	9.5238(4) Å
c	18.8230(7) Å
V	4935.6(4) $Å^3$
Space Group	C2/c (#15)
Z value	8
D_{calc}	1.242 g/cm^3
F_{000}	1952.00
No. of Reflections Measured	Total: 26984
	Unique: 4479 (R_{int} = 0.0642)
R_1 (I>2.00σ(I))	0.0750
R (All reflections)	0.1080
wR_2 (All reflections)	0.2380

*3-2. Crystal structure of compound **S4.***

A single-crystal was grown by leaving a concentrated solution of **S4** in CH_3CN at room temperature. All measurements were made on a Rigaku XtaLAB P200 diffractometer using multi-layer mirror monochromated Cu-Kα radiation. All non-hydrogen atoms were refined anisotropically, and all hydrogenatoms were refined using the riding model. Refined structure and crystallographic parameters are summarized in Supplementary Fig. 4 and Supplementary Table 2. CCDC 1909994 contains the supplementary crystallographic data for **S4**.

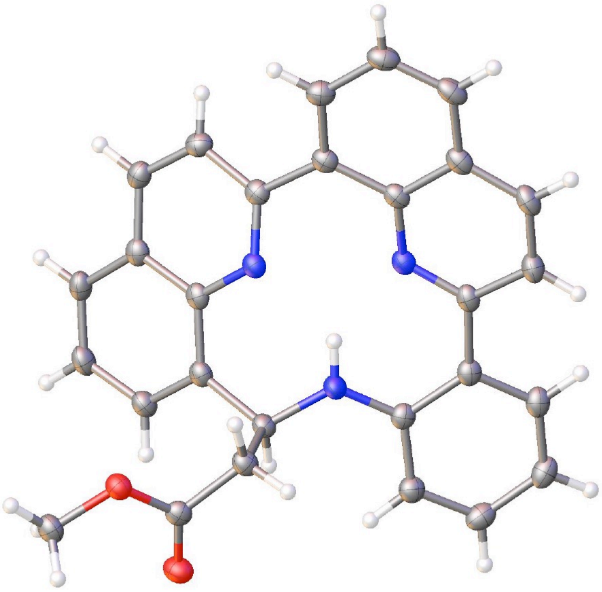

Supplementary Fig. 4 Structure of **S4**. [ORTEP plot (50% ellipsoids)], color code; gray: C, white: H, blue: N, red: O.

Supplementary Table 2. Selected crystal data of S4

Empirical Formula	$C_{28}H_{21}N_3O_2$
Formula Weight	431.49
Temperature/°C	–180.0
Crystal Color, Habit	yellow, plate
Crystal Dimensions	0.200 x 0.200 x 0.080 mm
Crystal System	monoclinic
Cell constants	
a	21.451(2) Å
b	7.67214(5) Å
c	24.7559(6) Å
V	4054.5(4) Å^3
Space Group	I2/a (#15)
Z value	8
D_{calc}	1.414 g/cm^3
F_{000}	1808.00
No. of Reflections Measured	Total: 35678
	Unique: 4184 (R_{int} = 0.0891)
R_1 (I>2.00σ(I))	0.0446
R (All reflections)	0.0461
wR_2 (All reflections)	0.1223

4. ¹H NMR Monitoring of Triquinoline Formation

*4-1. Reaction of DQ-Im•TFA **9** with butyl vinyl ether (BVE).*

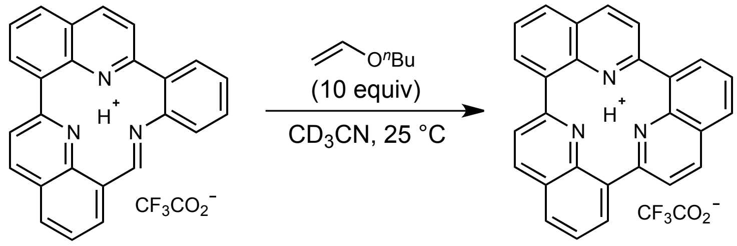

9 **10**

Butyl vinyl ether (BVE, 0.55 μL, 4.24 μmol, 10 equiv) was added to a CD_3CN (530 μL) solution of DQ-Im•TFA **9** (0.20 mg, 0.424 μmol, 1.0 equiv) in a 5 mm NMR tube. After being sealed with a Teflon cap, the nmr tube was inverted several times, and ¹H NMR analysis was performed. The nmr sample was left stand at 25 °C and monitored by ¹H NMR at the times indicated below (Supplementary Fig. 5). After 24 h, the peak derived from BVE (6.5 ppm) disappeared, presumably because DQ-Im•TFA **9** promoted hydrolysis to give acetaldehyde.

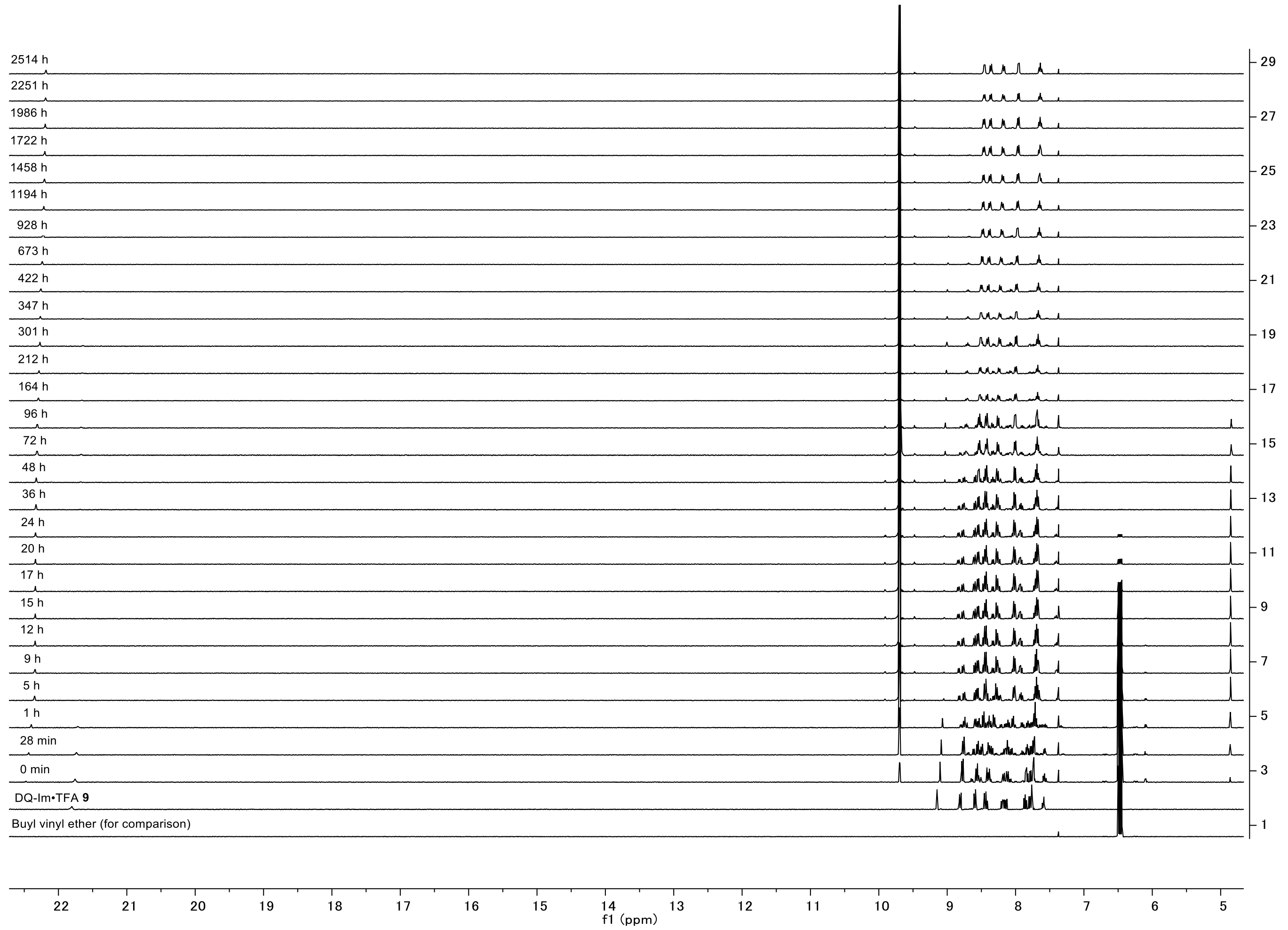

Supplementary Fig. 5 ¹H NMR monitoring of TQ•TFA **10** formation; note that the times at which the spectra were taken are shown on the left.

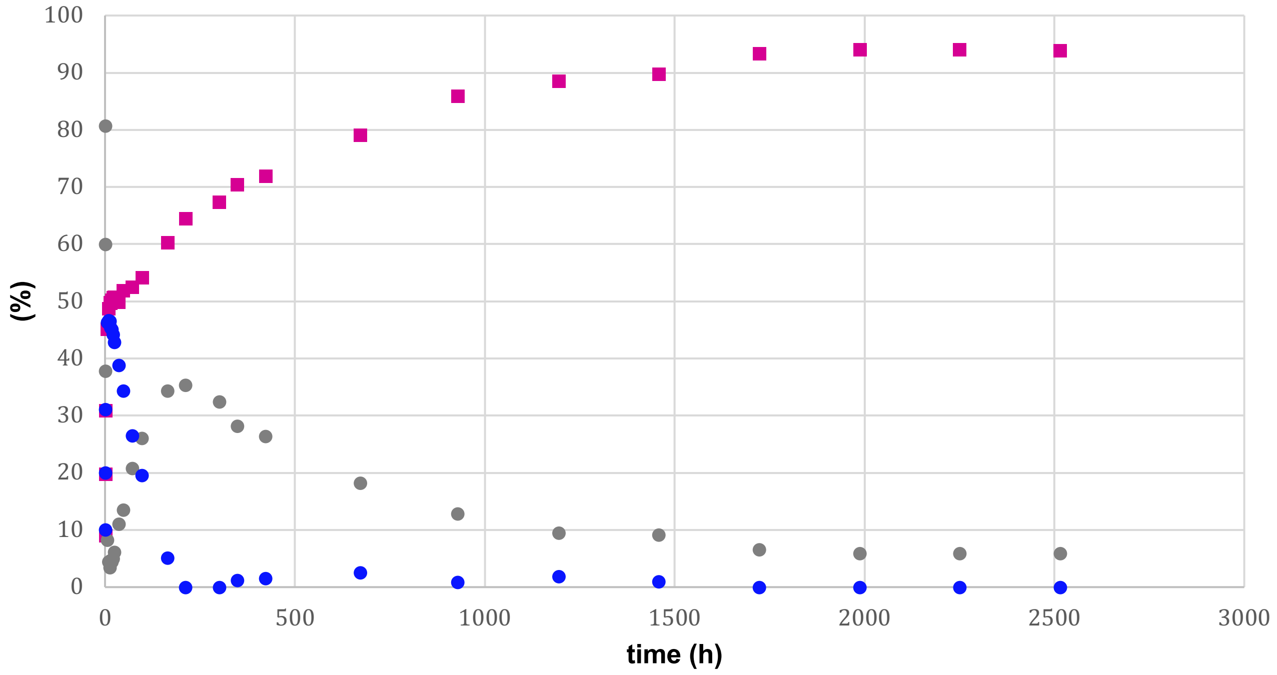

Supplementary Fig. 6 Kinetic profile of TQ•TFA **10** formation (gray; DQ-Im▪TFA **9**, blue; DQ-Am▪TFA **11**, red; TQ▪TFA **10**).

<u>*4-2. Amine formation via the reduction of DQ-Im•TFA **9** and oxidation of DQ-Am•TFA **11** into DQ-Im•TFA **9** under air.*</u>

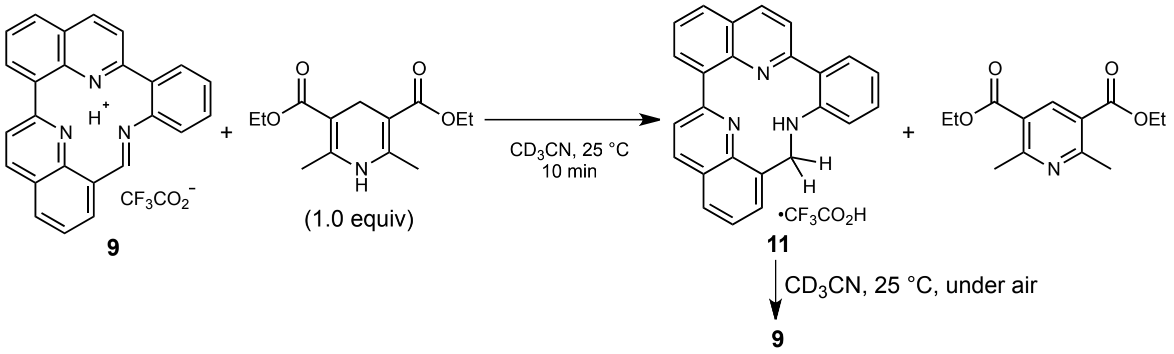

DQ-Im•TFA **9** (0.20 mg, 0.424 μmol, 1.0 equiv), diethyl 1,4-dihydro-2,6-dimethyl-3,5-pyridinedicarboxylate (Hantzsch ester, 0.11 mg, 0.424 μmol, 1.0 equiv), and CD_3CN (530 μL) were added to a 5 mm NMR tube. After being sealed with a Teflon cap, the nmr tube was inverted several times and was then left stand at 25 °C for 10 min. The nmr tube was placed into an NMR spectrometer, and the ^1H NMR analysis was performed, confirming the quantitative formation of DQ-Am•TFA **11** (Supplemenary Fig. 7, spectrum at 10 min). The nmr sample was left stand at 25 °C and monitored by ^1H NMR at the times indicated. The kinetic profile of re-oxidation of DQ-Am•TFA **11** to DQ-Im•TFA **9** is given in Supplementary Fig. 8). The HRMS of the resulting solution also indicated the presence of DQ-Am (ESI, *m*/*z* calculated for $C_{25}H_{18}N_3$ $[M+H]^+$: 360.1495, found: 360.1491). DQ-Am•TFA is not sufficiently stable for isolation in an analytically pure form.

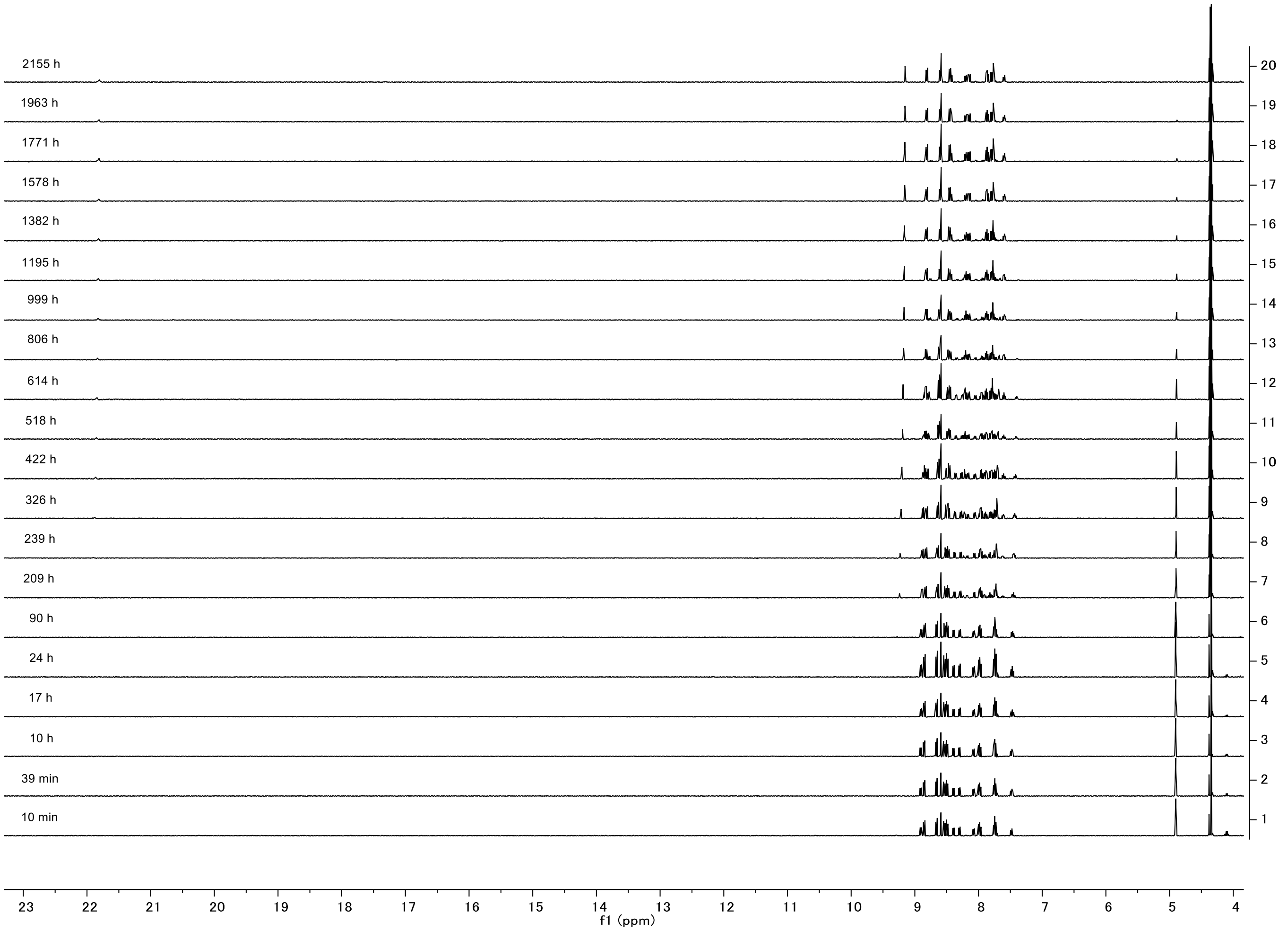

Supplementary Fig. 7 ^{1}H NMR monitoring of oxidation of DQ-Am•TFA **11** into DQ-Im•TFA **9**; note that the times at which the spectra were taken are shown on the left.

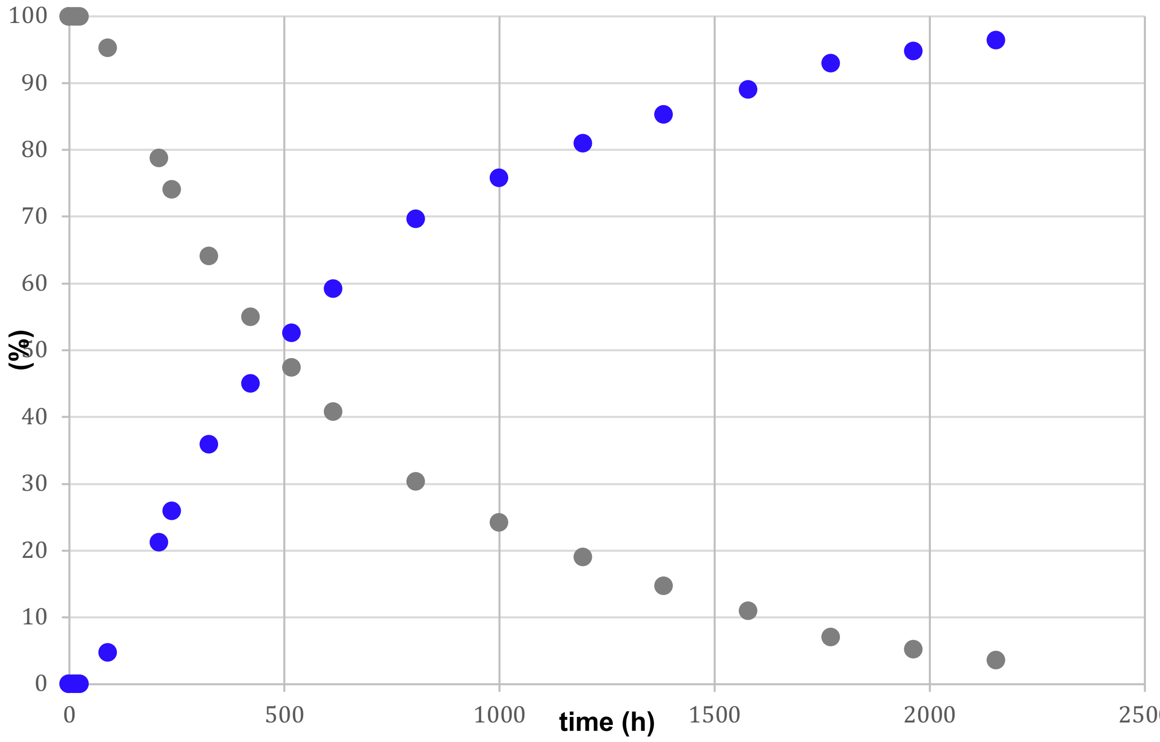

Supplementary Fig. 8 Kinetic profile of oxidation of DQ-Am•TFA **11** into DQ-Im•TFA **9** (blue; DQ-Im•TFA **9**, gray; DQ-Am•TFA **11**).

*4-3. Reaction of DQ-Im•TFA **9** with butyl vinyl ether (BVE) in a glove box.*

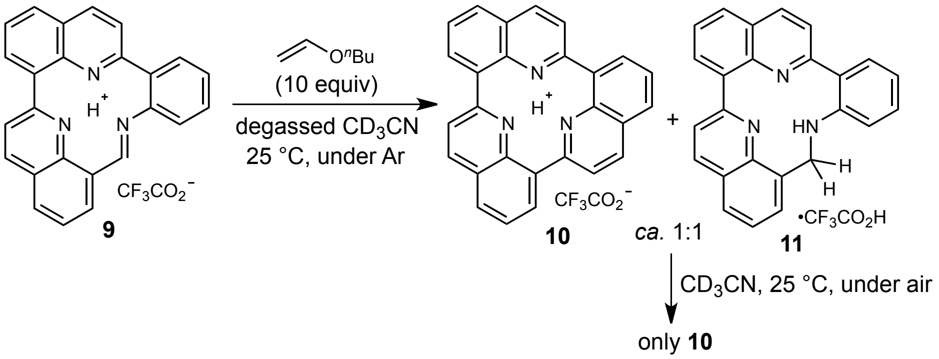

In a glove box, butyl vinyl ether (BVE, 0.55 μL, 4.24 μmol, 10 equiv) was added to a degassed CD_3CN (530 μL) solution of DQ-Im•TFA **9** (0.20 mg, 0.424 μmol, 1.0 equiv) in a 5 mm NMR tube. After being sealed with a Teflon cap, the nmr tube was inverted several times and left stand at 25 °C for 25 h. The nmr tube was taken from the glove box, and placed into an NMR spectrometer. The ^1H NMR analysis indicated formation of *ca.* 1:1 mixture of TQ•TFA **10** and DQ-Am•TFA **11** (Supplementary Fig. 9a,10,11). The presence of both DQ-Am and TQ in the nmr sample was also confirmed by ESI-MS ([DQ-Am + H]$^+$: 360.12, [TQ + H]$^+$: 382.10). The nmr tube was unsealed and occasionally shaken to keep air saturation of the solution. After being left stand at 25 °C for 14 days. the nmr sample was analyzed by ^1H NMR. As a result, the quantitative formation of TQ•TFA **10** was confirmed (Supplementary Fig. 9b). Magnified ^1H NMR spectra and ESI-MS spectrum are given in Supplemenatry Fig 10,11.

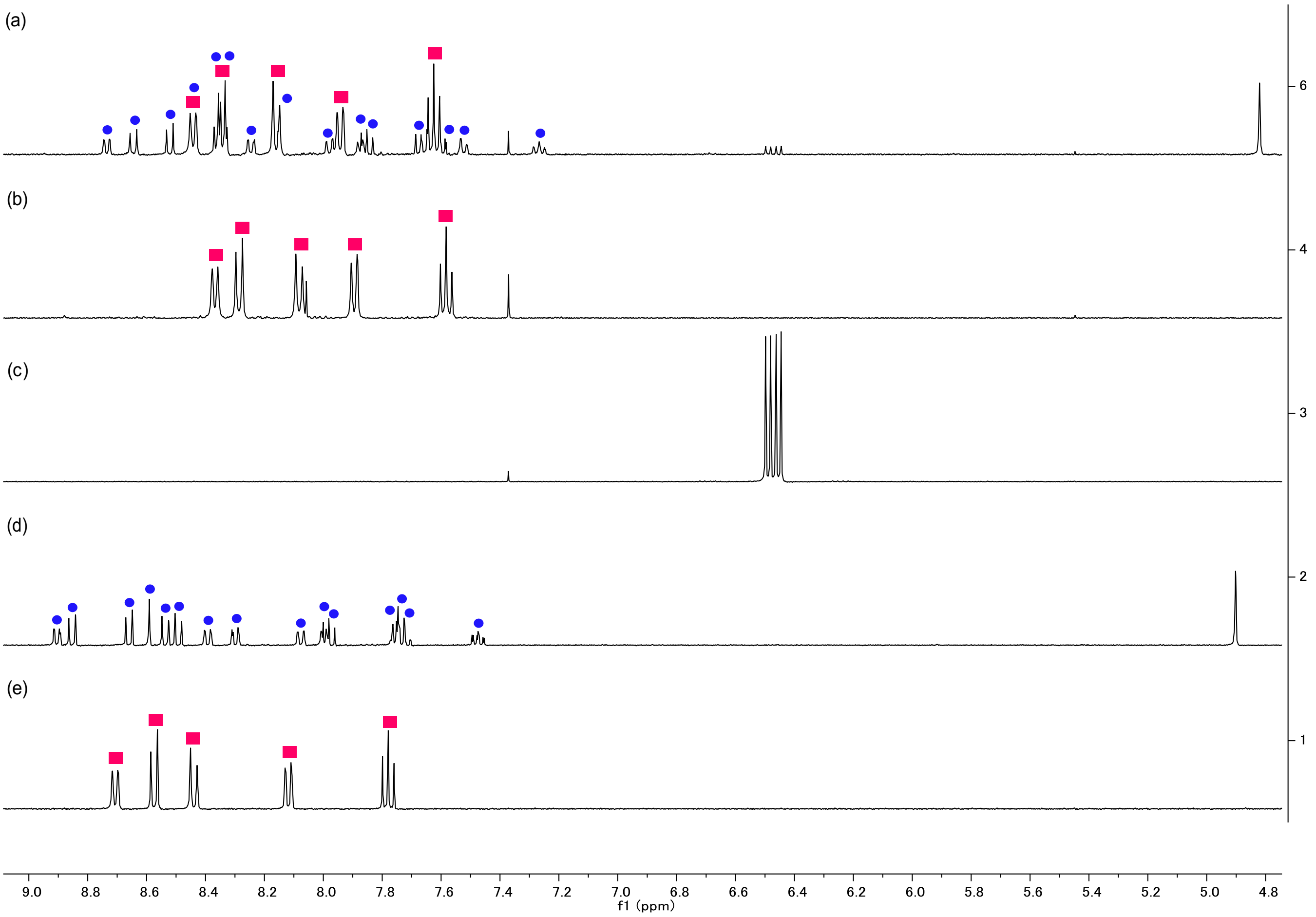

Supplementary Fig. 9 ^1H NMR spectra (CD_3CN) (a) after 25 h in a glove box, (b) after 14 days under air, (c) butyl vinyl ether (BVE, for comparison), (d) DQ-Am•TFA **11** (for comparison), (e) TQ•TFA **10** (for comparison).

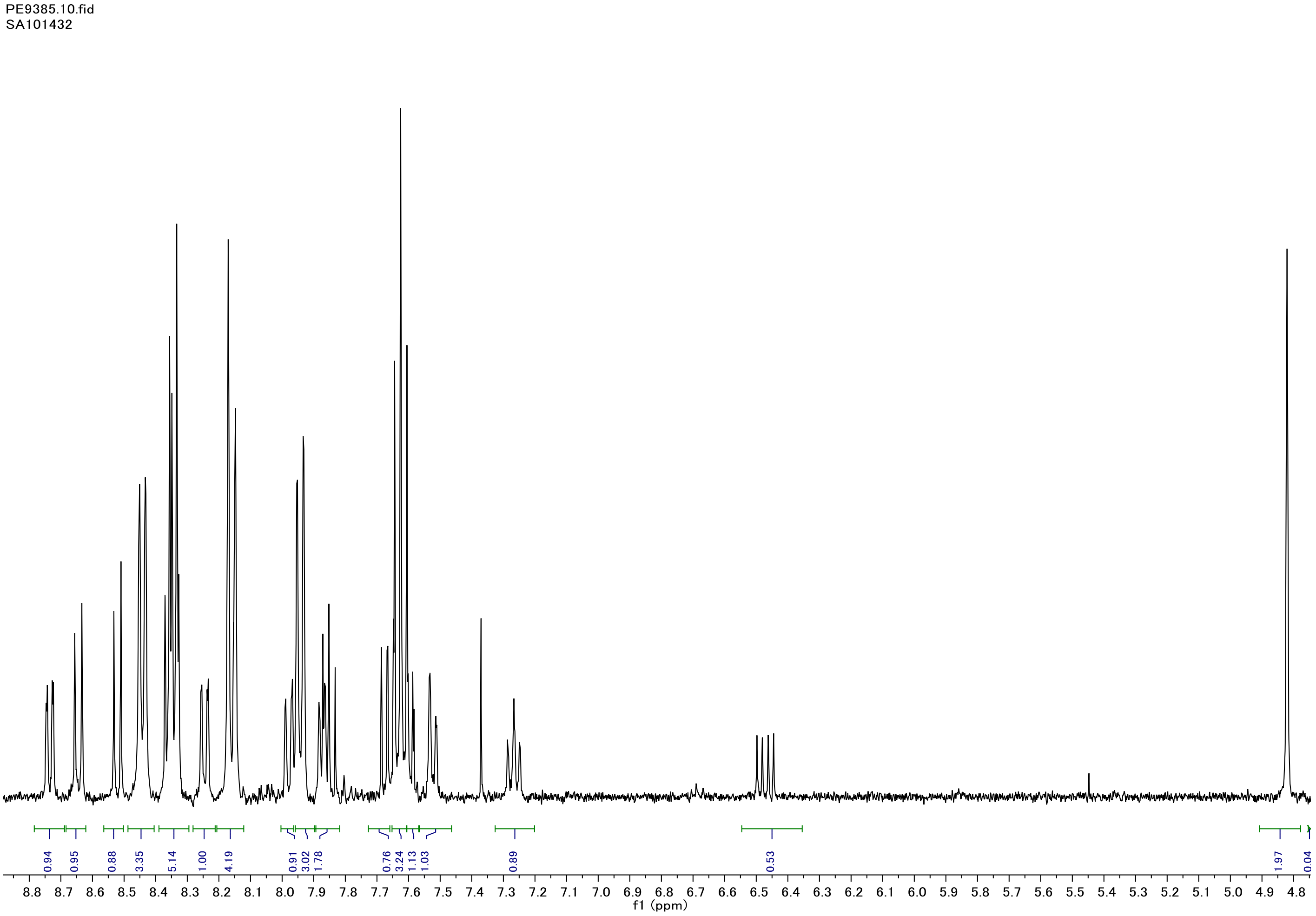

Supplementary Fig. 10 Magnified ^{1}H NMR spectrum (CD_3CN) with integration (after 25 h in a glove box showing the 1:1 mixture of TQ•TFA **10** and DQ-Am•TFA **11**).

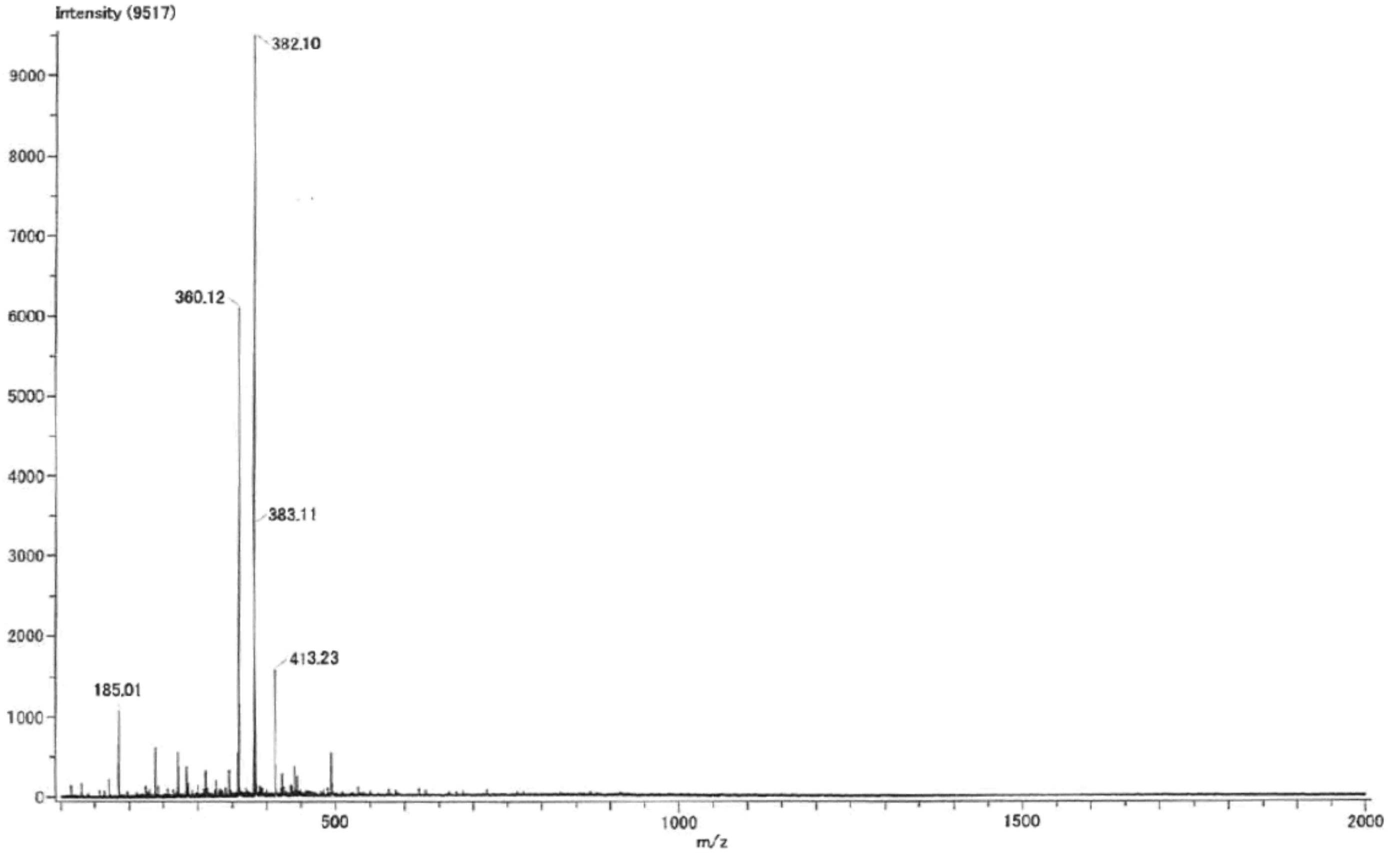

Supplementary Fig. 11 ESI-MS spectrum (after 25 h in a glove box showing the 1:1 mixture of TQ•TFA **10** and DQ-Am•TFA **11**).

4-4. Reaction of DQ-Im•TFA **9** with acetaldehyde.

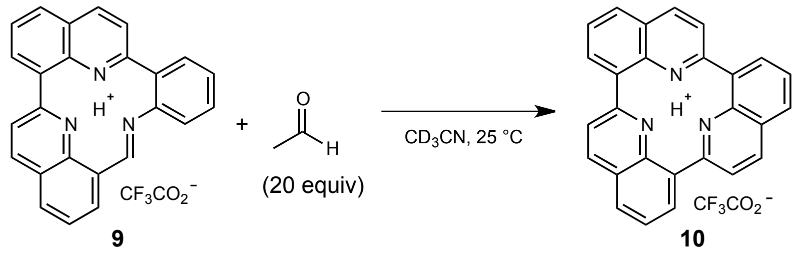

Acetaldehyde (0.48 μL, 8.48 μmol, 20 equiv) was added to a CD_3CN (530 μL) solution of DQ-Im•TFA **9** (0.20 mg, 0.424 μmol, 1.0 equiv) in a 5 mm NMR tube. After being sealed with a Teflon cap, the nmr tube was inverted several times, and ^1H NMR analysis was performed. The nmr sample was left stand and monitored by ^1H NMR at the times indicated below.

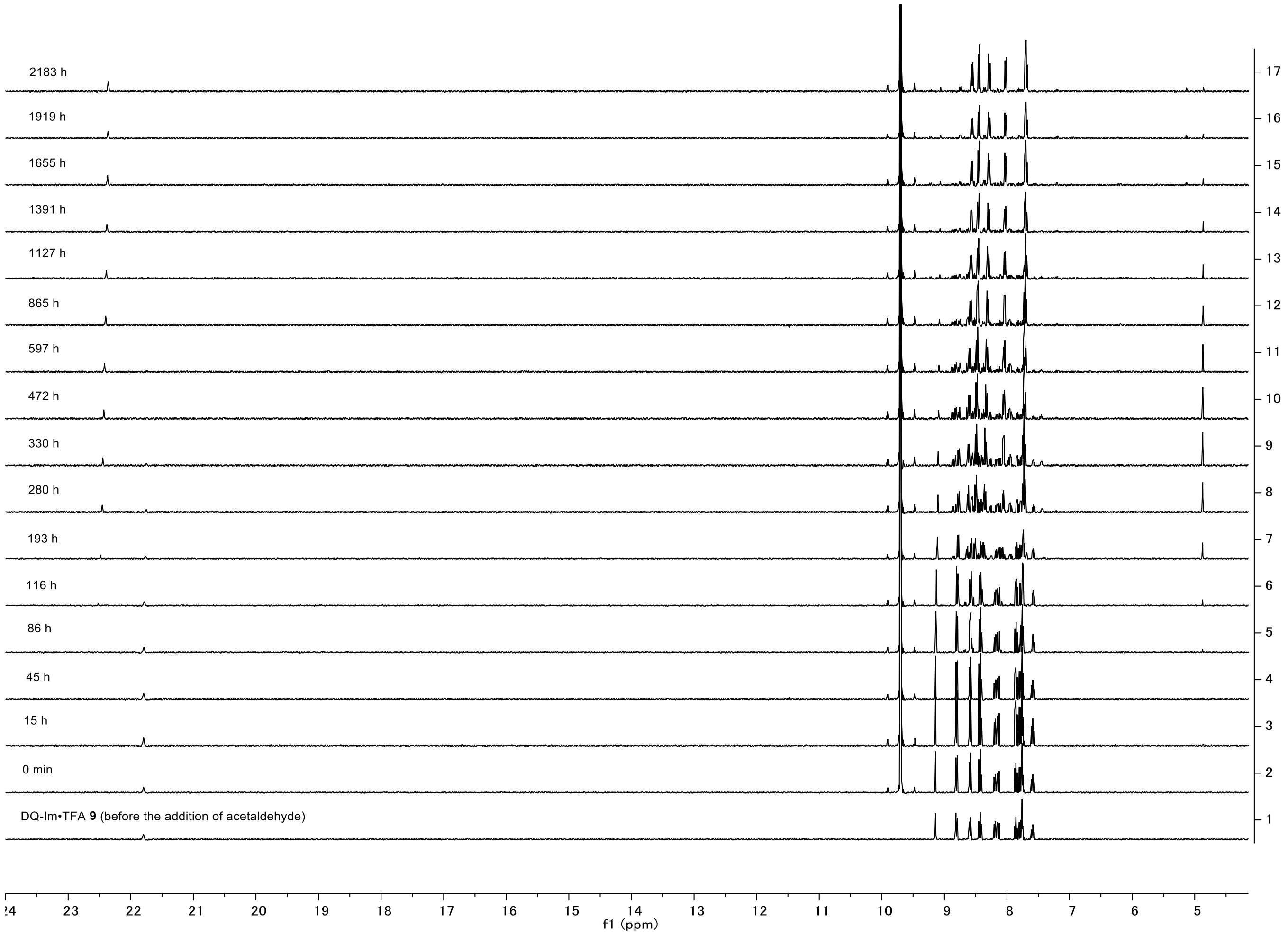

Supplementary Fig. 12 ^1H NMR monitoring of reaction of DQ-Im•TFA **9** with acetaldehyde; note that the times at which the spectra were taken are shown on the left.

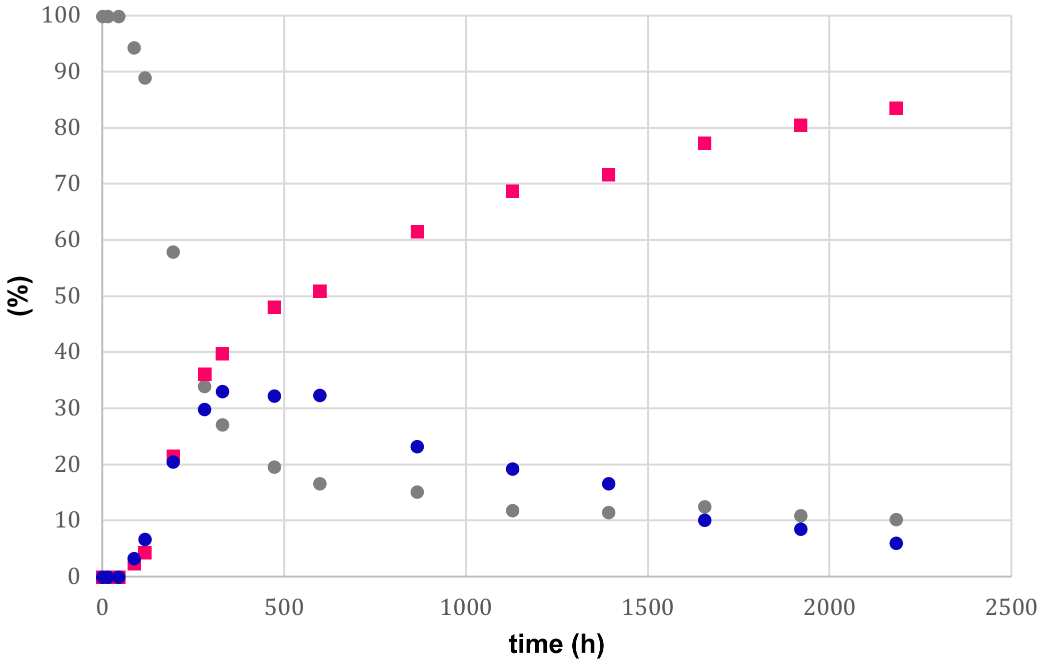

Supplementary Fig. 13. Kinetic profile of reaction of DQ-Im•TFA **9** with acetaldehyde (gray; DQ-Im･TFA **9**, blue; DQ-Am･TFA **11**, red; TQ•TFA **10**).

5. Measurement of H^+/D^+ Exchange Rate

*5-1. H^+/D^+ exchange rate in TQ•TFA **10** in the absence of TFA.*

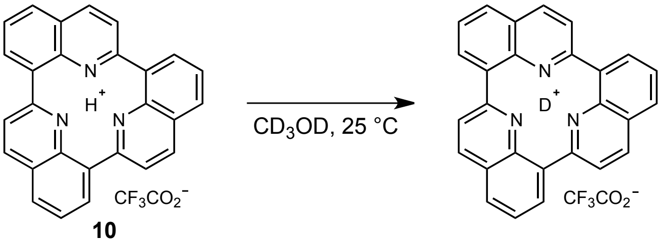

TQ•TFA **10** (0.51 mg, 1.03 μmol, 1.0 equiv) and CD_3OD (530 μL) were added to a 5 mm NMR tube (mole ratio of **10**/CD_3OD is 1/12676). After being sealed with a Teflon cap, the nmr tube was inverted several times, and ^1H NMR analysis was performed. The nmr sample was left stand and monitored by ^1H NMR at the times indicated below. By assuming pseudo first order rate dependency, rate constant k was determined to be $k = 3.88 \times 10^{-6}$ (s^{-1}) (Supplementray Fig. 17), which corresponds to half life of 49.6 h.

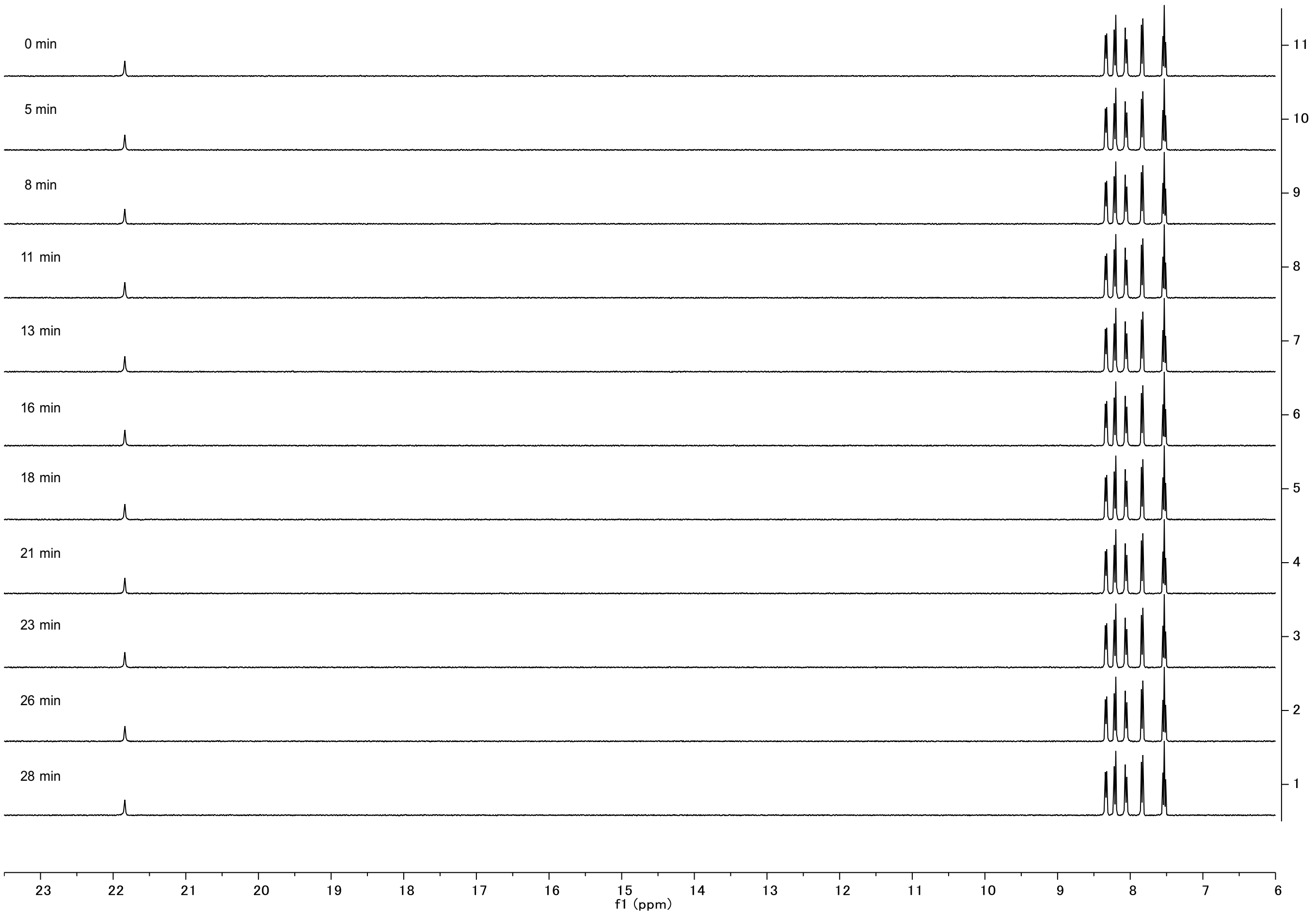

Supplementary Fig. 14 ^1H NMR monitoring of H^+/D^+ exchange in TQ•TFA **10** (0–28 min); note that the times at which the spectra were taken are shown on the left.

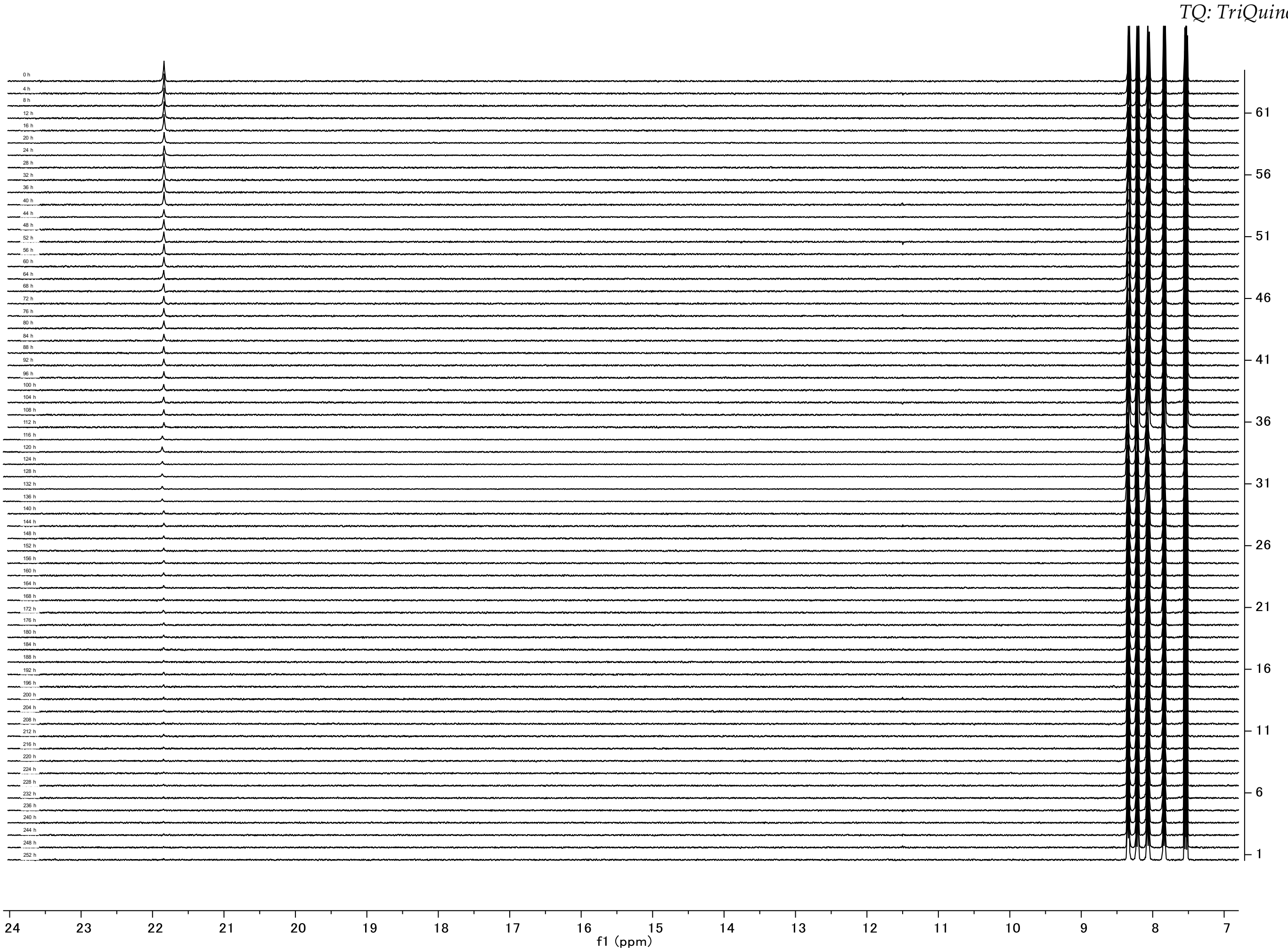

Supplementary Fig. 15 ^{1}H NMR monitoring of H^{+}/D^{+} exchange in TQ•TFA **10** (0–252 h); note that the times at which the spectra were taken are shown on the left.

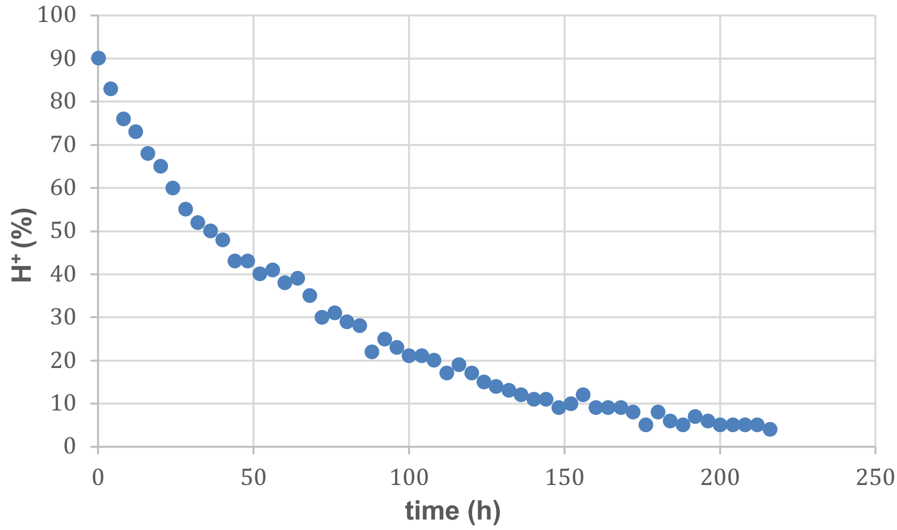

Supplementary Fig. 16 Kinetic profile of H^{+}/D^{+} exchange in TQ•TFA **10**.

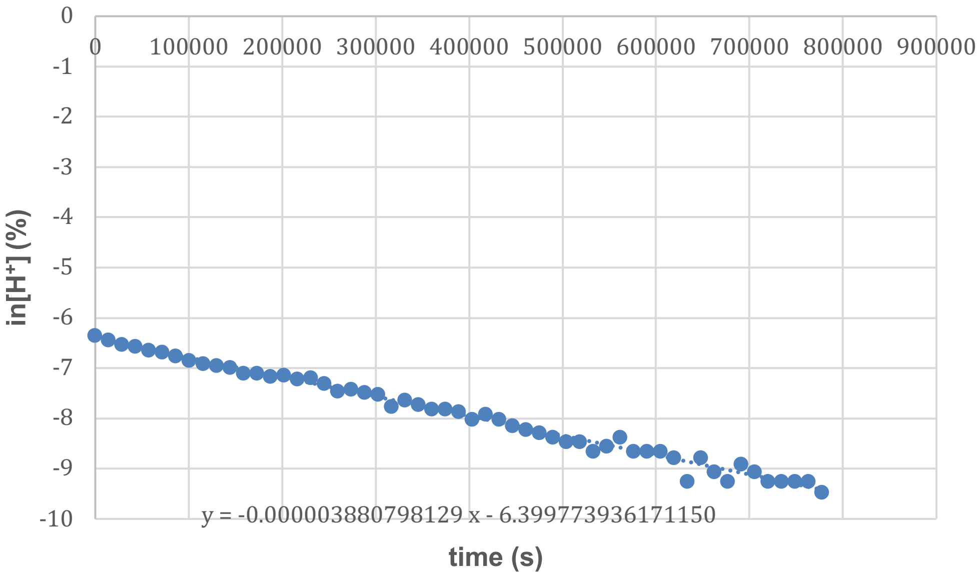

Supplementary Fig. 17 Time vs ln[H$^+$] plot of H$^+$/D$^+$ exchange in TQ•TFA **10.**

*5-2. H$^+$/D$^+$ exchange rate in TQ•TFA **10** in the presence of 3.3 ppm of CF_3COOD.*

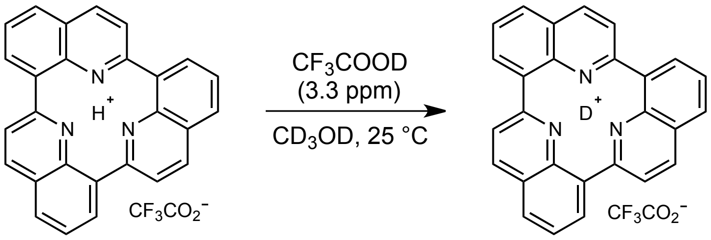

0.02%(v/v) CF_3COOD in CD_3OD (8.8 μL) was added to a CD_3OD (521.2 μL) solution of TQ•TFA **10** (0.51 mg, 1.03 μmol, 1.0 equiv) in a 5 mm NMR tube. After being sealed with a Teflon cap, the nmr tube was inverted several times, and ^{1}H NMR analysis was performed. The nmr sample was left stand and monitored by ^{1}H NMR at the times indicated below. By assuming pseudo first order rate dependency, rate constant k was determined to be $k = 6.61 \times 10^{-4}$ (s^{-1}) (Supplementray Fig. 20), which corresponds to half life of 17.5 min.

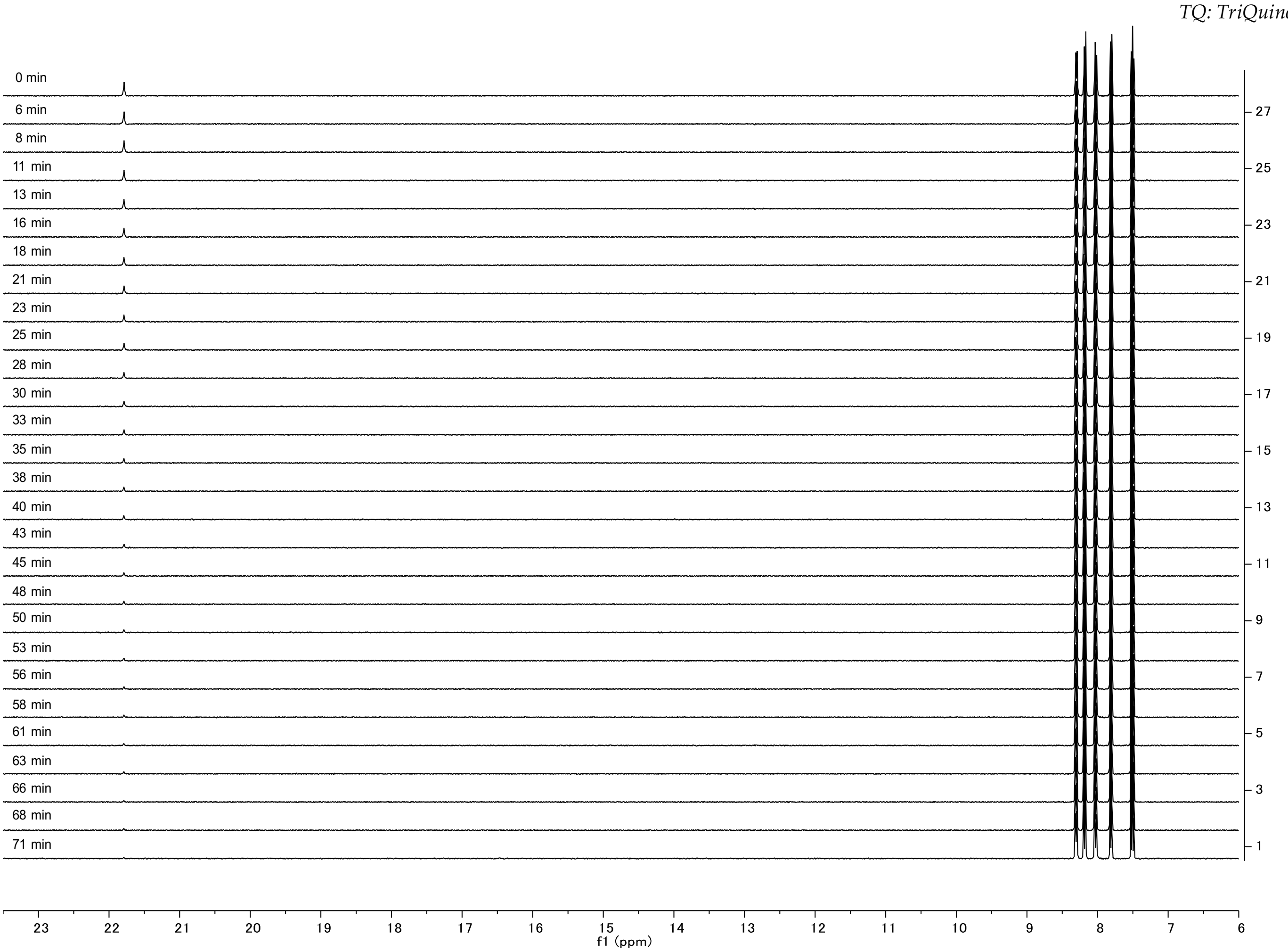

Supplementary Fig. 18 ^{1}H NMR monitoring of H^{+}/D^{+} exchange in TQ•TFA **10** in the presence of TFA (3.3 ppm); note that the times at which the spectra were taken are shown on the left.

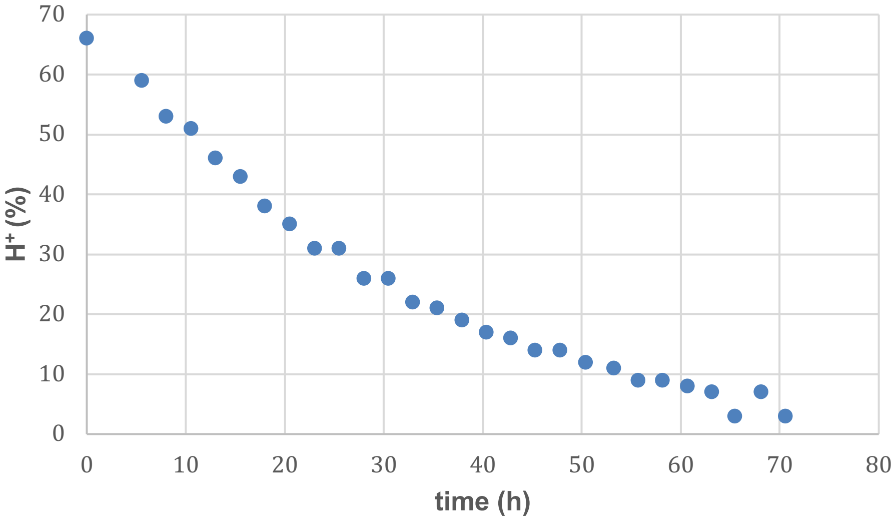

Supplementary Fig. 19 Kinetic profile of H^{+}/D^{+} exchange in TQ•TFA **10** in the presence of CF_3COOD (3.3 ppm)

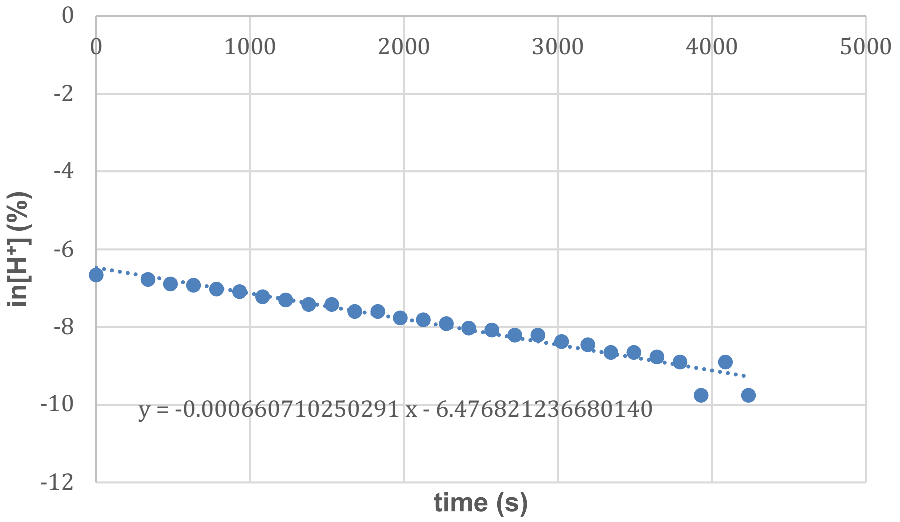

Supplementary Fig. 20 Time vs ln[H⁺] plot of H⁺/D⁺ exchange in TQ▪TFA **10** in the presence of CF_3COOD (3.3 ppm)

*5-2. H⁺/D⁺ exchange rate in TQ•TFA **10** in the presence of 6.7 ppm of CF_3COOD.*

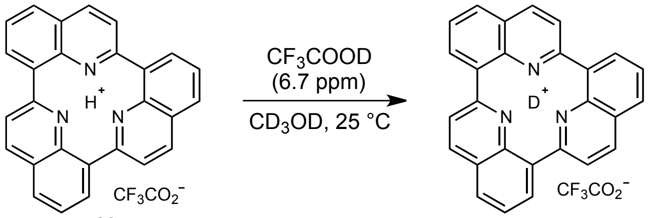

10

0.02%(v/v) CF_3COOD in CD_3OD (17.7 μL) was added to a CD_3OD (512.3 μL) solution of TQ•TFA **10** (0.51 mg, 1.03 μmol, 1.0 equiv) in a 5 mm NMR tube. After being sealed with a Teflon cap, the nmr tube was inverted several times, and ^{1}H NMR analysis was performed. The nmr sample was left stand and monitored by ^{1}H NMR at the times indicated below. By assuming pseudo first order rate dependency, rate constant k was determined to be $k = 1.15 \times 10^{-3}$ (s^{-1}) (Supplementray Fig. 23), which corresponds to half life of 10.0 min.

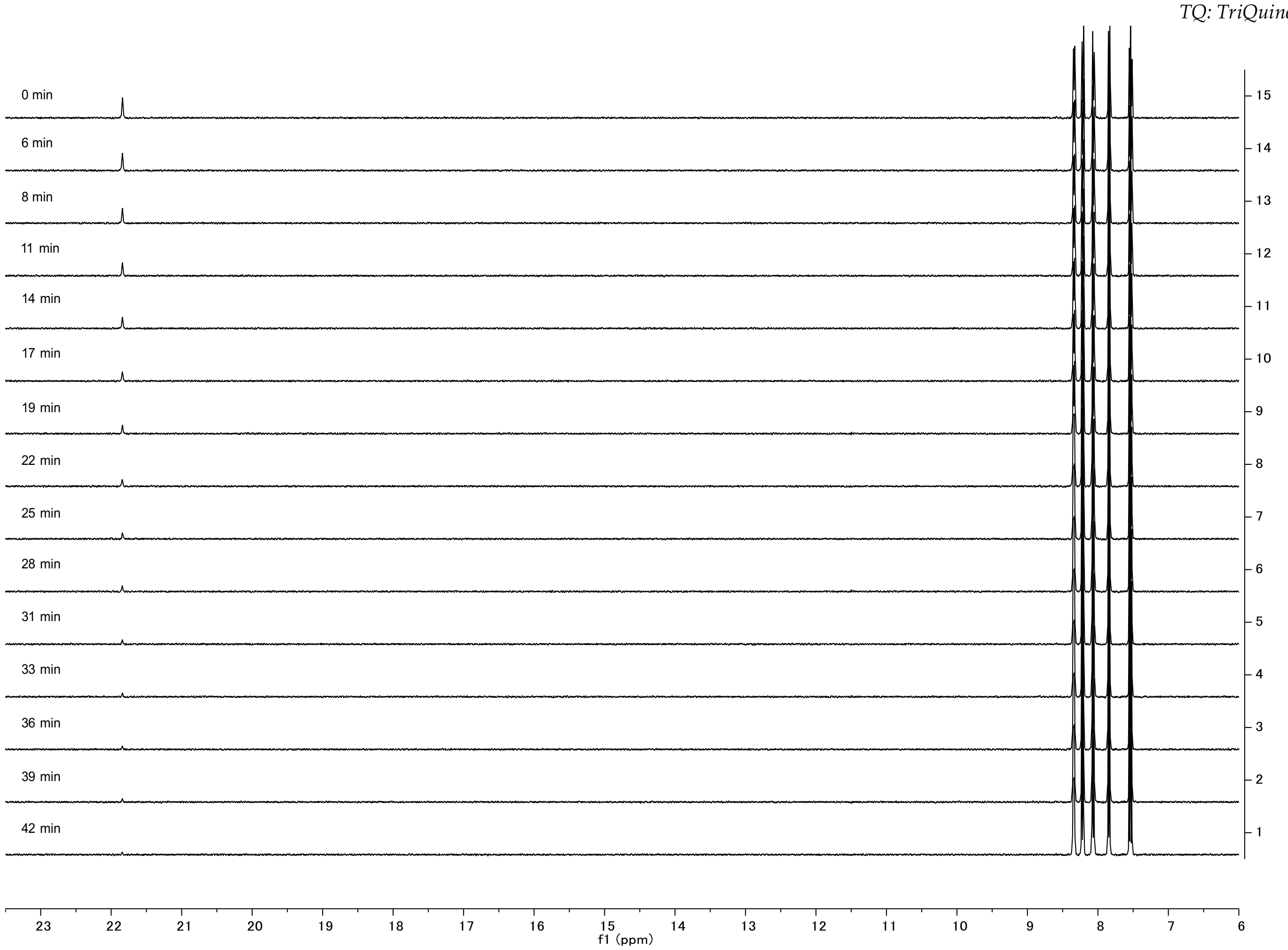

Supplementary Fig. 21 ^{1}H NMR monitoring of H^{+}/D^{+} exchange in TQ•TFA **10** in the presence of CF_3COOD (6.7 ppm); note that the times at which the spectra were taken are shown on the left.

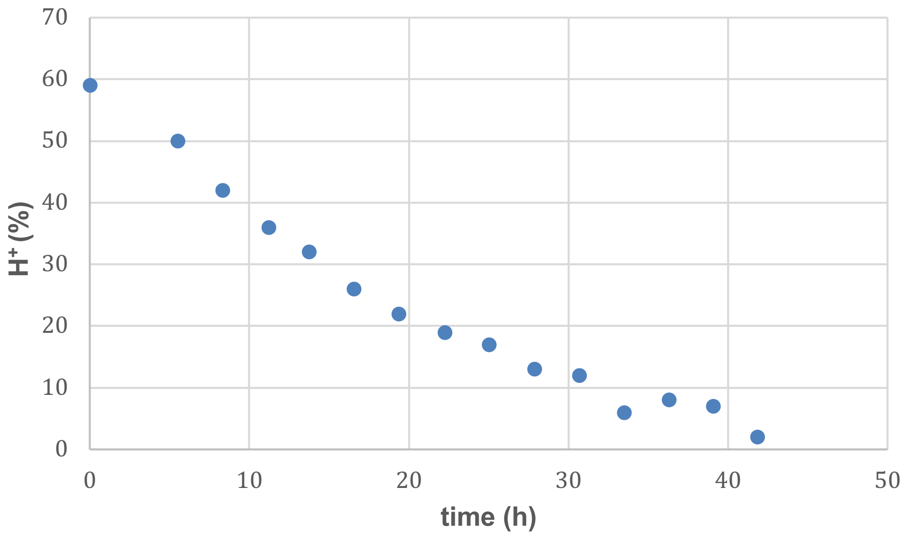

Supplementary Fig. 22 Kinetic profile of H^{+}/D^{+} exchange in TQ•TFA **10** in the presence of CF_3COOD (6.7 ppm).

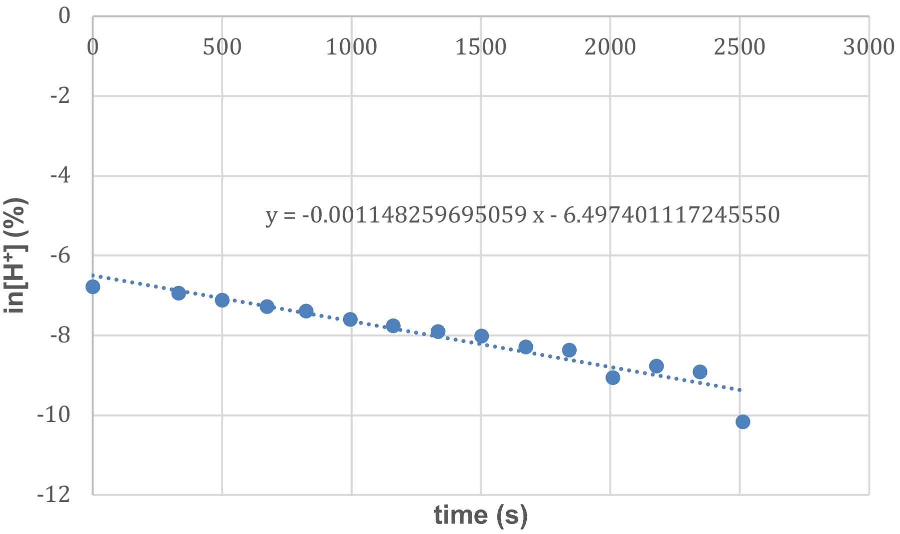

Supplementary Fig. 23 Time vs ln[H+] plot of H+/D+ exchange in TQ•TFA **10** in the presence of CF_3COOD (6.7 ppm).

<u>*5-3. H+/D+ exchange rate in TQ•TFA **10** in the presence of 10 ppm of CF_3COOD.*</u>

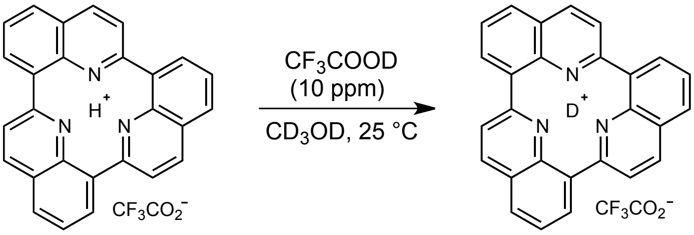

10

0.02%(v/v) CF_3COOD in CD_3OD (26.5 µL) was added to a CD_3OD (503.5 µL) solution of TQ•TFA **10** (0.51 mg, 1.03 µmol, 1.0 equiv) in a 5 mm NMR tube. After being sealed with a Teflon cap, the nmr tube was inverted several times, and ^{1}H NMR analysis was performed. The nmr sample was left stand and monitored by ^{1}H NMR at the times indicated below. By assuming pseudo first order rate dependency, rate constant *k* was determined to be $k = 2.52 \times 10^{-3}$ (s^{-1}) (Supplementray Fig. 26), which corresponds to half life of 4.6 min.

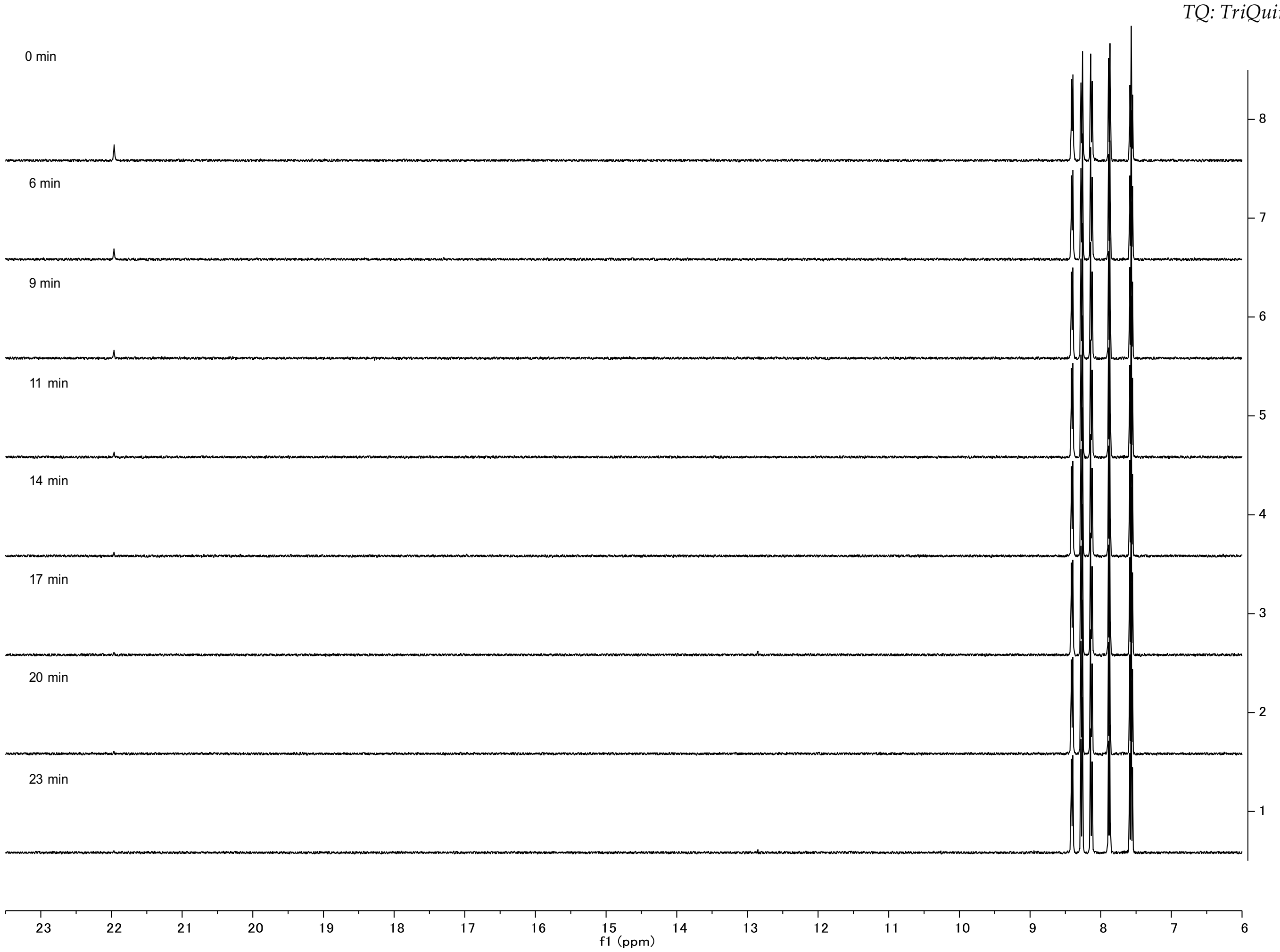

Supplementary Fig. 24 ^{1}H NMR monitoring of H^{+}/D^{+} exchange in TQ•TFA **10** in the presence of CF_3COOD (10 ppm); note that the times at which the spectra were taken are shown on the left.

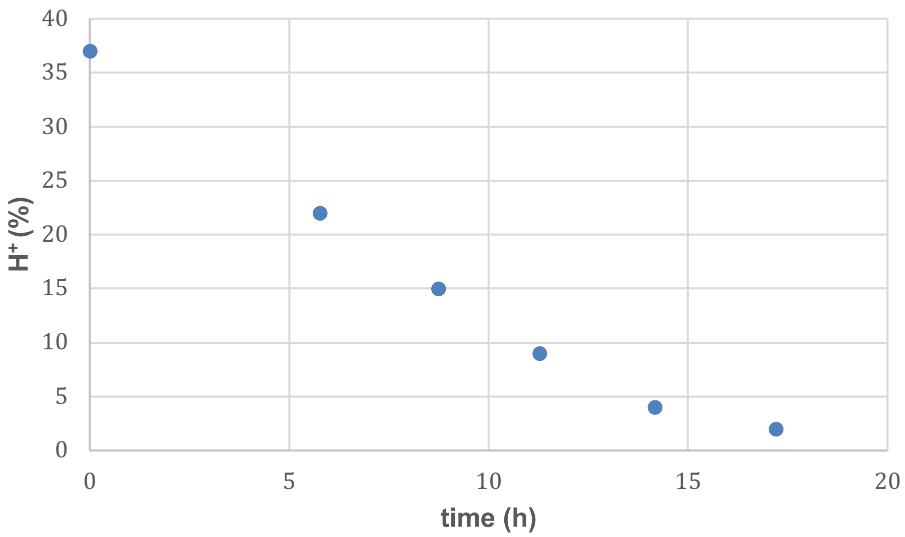

Supplementary Fig. 25 Kinetic profile of H^{+}/D^{+} exchange in TQ•TFA **10** in the presence of CF_3COOD (10 ppm).

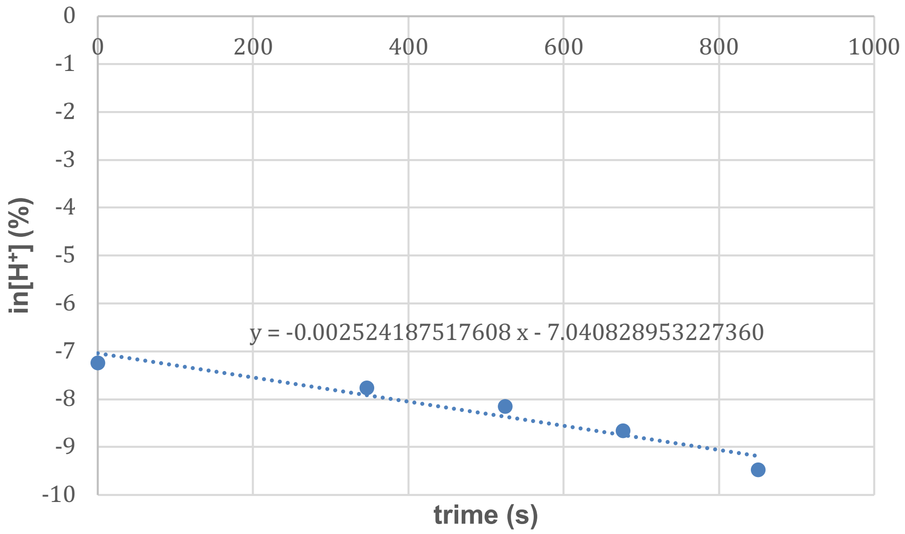

Supplementary Fig. 26 Time vs ln[H+] plot of H+/D+ exchange in TQ•TFA **10** in the presence of CF_3COOD (10 ppm).

From the collective data above, H+/D+ exchange is highly sensitive to the presence of tiny amount of CF_3COOD. Rate constant and concentration of acid are in linear relationship (Supplementary Fig. 27), indicating that TQ•H+ can be used to probe the tiny amount of acid in the media of interest.

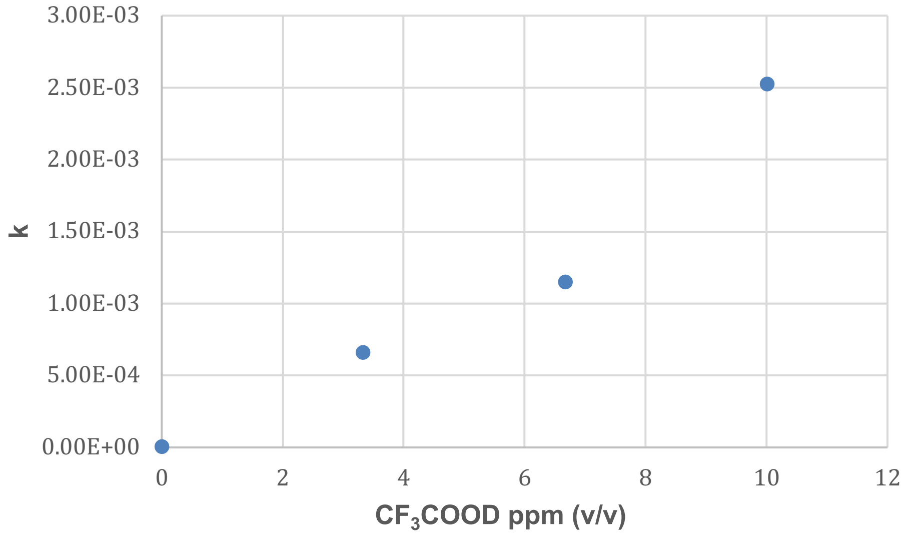

Supplementary Fig. 27 Effect of CF_3COOD in kinetic constant

5-4. H+/D+ exchange rate in ***13*** *•TFA.*

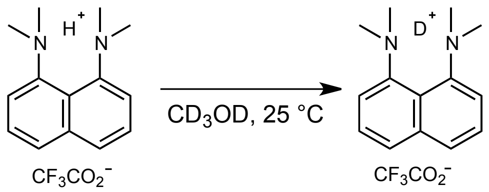

13•CF_3CO_2H **13**•CF_3CO_2D

13•TFA (0.34 mg, 1.03 μmol, 1.0 equiv) and CD_3OD (530 μL) were added to a 5 mm NMR tube. After being sealed with a Teflon cap, the nmr tube was inverted several times, and ^1H NMR analysis was performed. The nmr sample was left stand and monitored by ^1H NMR at the times indicated below.

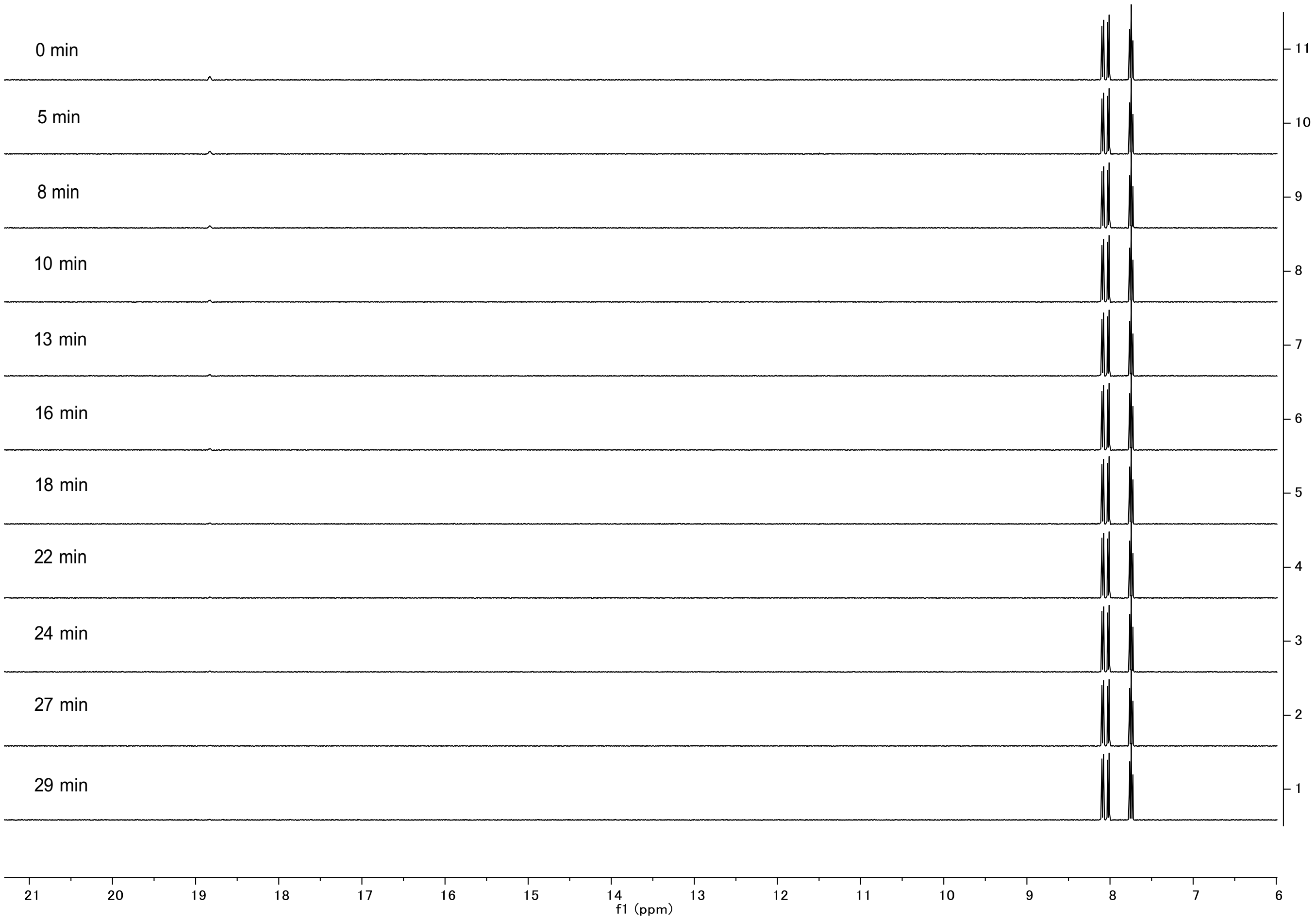

Supplementary Fig. 28 ^{1}H NMR monitoring of H^+/D^+ Exchange in **13**•TFA; note that the times at which the spectra were taken are shown on the left.

<u>*5-5. H^+/D^+ exchange rate in DQ-Im •TFA **9** or **S5**•TFA.*</u>

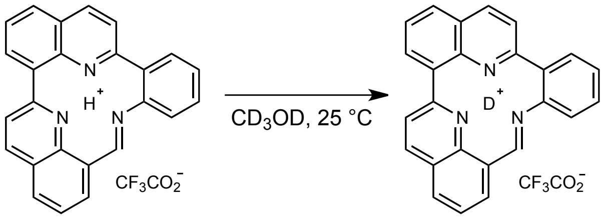

9

DQ-Im•TFA **9** (0.49 mg, 1.03 μmol, 1.0 equiv) and CD_3OD (530 μL) were added to a 5 mm NMR tube. After being sealed with a Teflon cap, the nmr tube was inverted several times and was immediately placed into an NMR spectrometer to perform ^{1}H NMR analysis (Supplementary Fig. 29a,b).

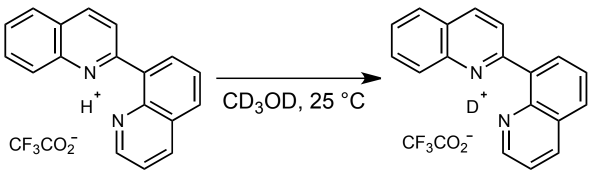

S5• CF_3CO_2H **S5**• CF_3CO_2D

2,8'-biquinoline•TFA (**S5**•TFA) (0.38 mg, 1.03 μmol, 1.0 equiv) and CD_3OD (530 μL) were added to a 5 mm NMR tube. After being sealed with a Teflon cap, the nmr tube was inverted several times and was immediately placed into an NMR spectrometer to perform ^{1}H NMR analysis (Supplementary Fig. 29c,d).

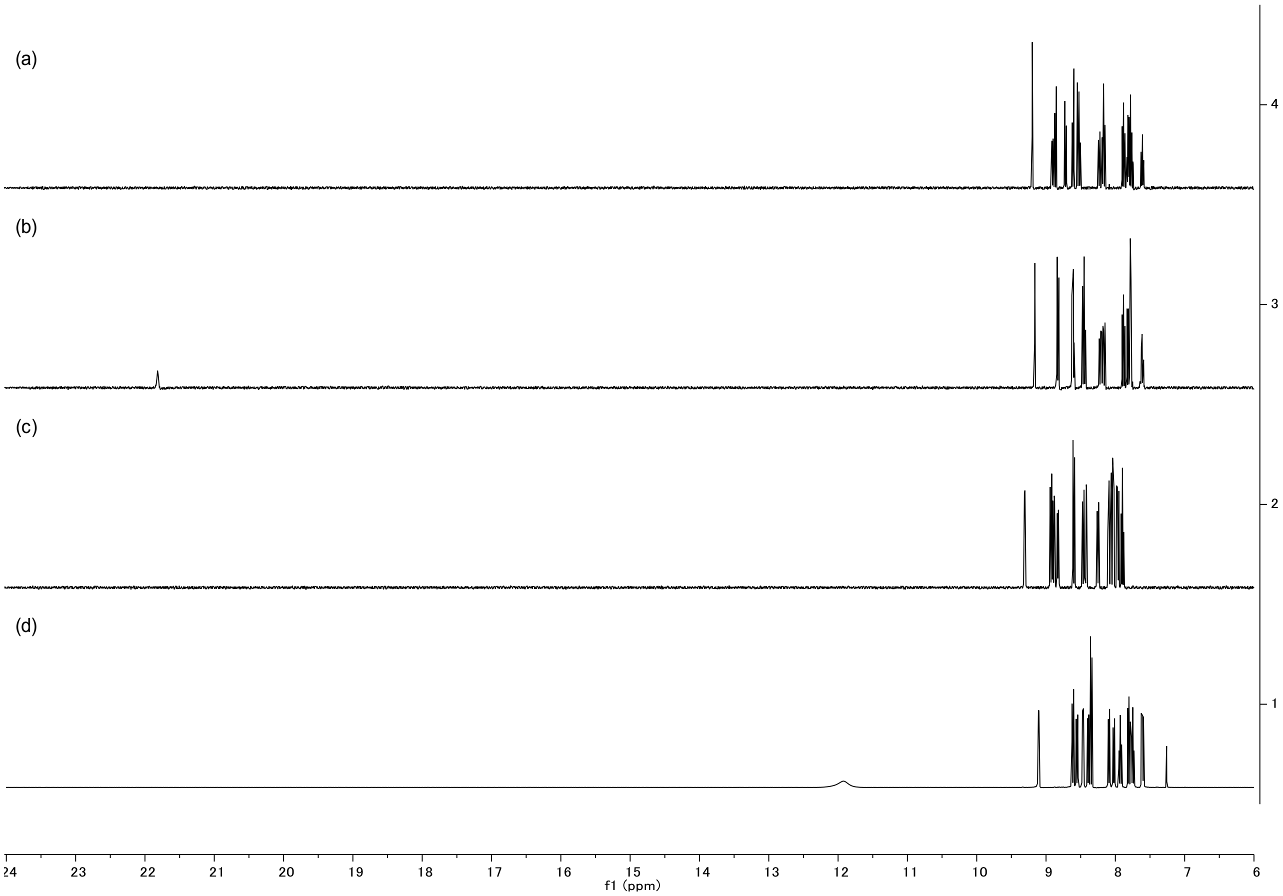

Supplementary Fig. 29 ^{1}H NMR spectra (a) DQ-Im•TFA **9** in CD_3OD (after *ca.* 5 min of CD_3OD addition), (b) DQ-Im•TFA **9** in CD_3CN (for comparison), (c) 2,8'-biquinoline•TFA **S5•TFA** in CD_3OD (after *ca.* 5 min of CD_3OD addition), (d) 2,8'-biquinoline▪TFA (**S5**•TFA) in $CDCl_3$ (for comparison).

According to supplementary Fig. 29, DQ-Im•TFA **9** and **S5**•TFA released proton significantly faster than TQ•TFA.

6. ESI-TOF-MS Spectra of Supramolecular Complexes

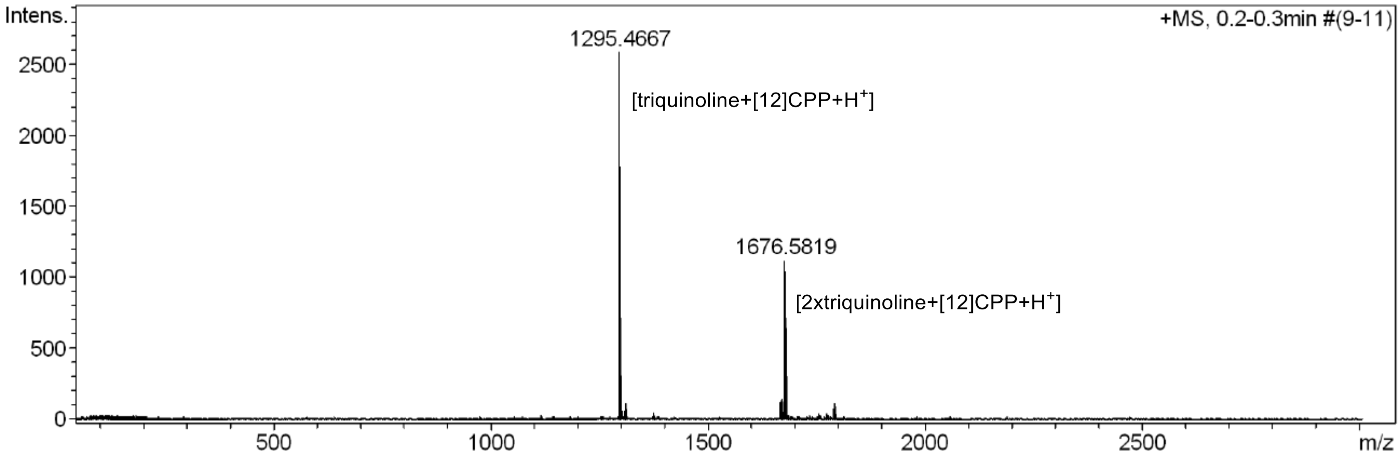

Supplementary Fig. 30 ESI-TOF-MS spectrum of the mixture of [12]CPP and TQ•TFA **10** in acetonitrile/DMSO.

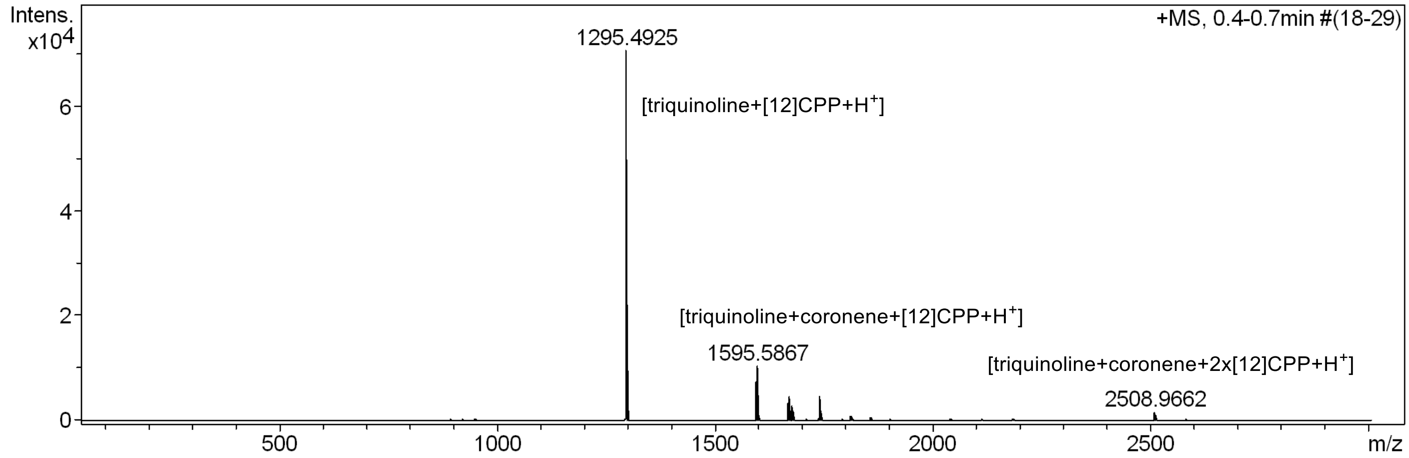

Supplementary Fig. 31 ESI-TOF-MS spectrum of the mixture of [12]CPP, TQ•TFA **10**, and coronene in acetonitrile/DMSO.

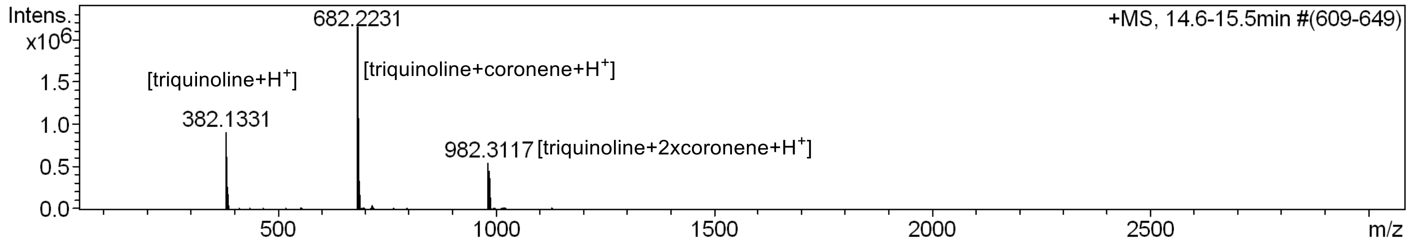

Supplementary Fig. 32 ESI-TOF-MS spectrum of the mixture of TQ•TFA **10** and coronene in acetonitrile/DMSO.

7. Determination of Association Constants from Titration Experiments

Non-linear regression analysis of ^{1}H NMR titration data was conducted by using bindfit web-based program (http://app.supramolecular.org/bindfit/) developed P. Thordarson, *et al.*[7-9]

*7-1. Complexation of TQ•TFA **10** and [12]CPP.*

To a d_6-DMSO solution of [12]CPP (1.84 x 10^{-4} M, 530 μL) was incrementally added a d_6-DMSO solution of TQ•TFA **10** (106.6 x 10^{-4} M) and analyzed by ^{1}H NMR (300K). The obtained data was fitted to 1:2 binding model, indicating that the formation of 1:1 inclusion complex [12]CPP⊃TQ•H$^+$ **15** and 1:2 inclusion complex [12]CPP⊃(TQ•H$^+$)$_2$ **16** with association constants of K_1 = (1.20 ± 0.035) x 10^3 M^{-1} and K_2 = (1.07 ± 0.011) x 10^3 M^{-1}, respectively (Supplementary Fig. 33–36).

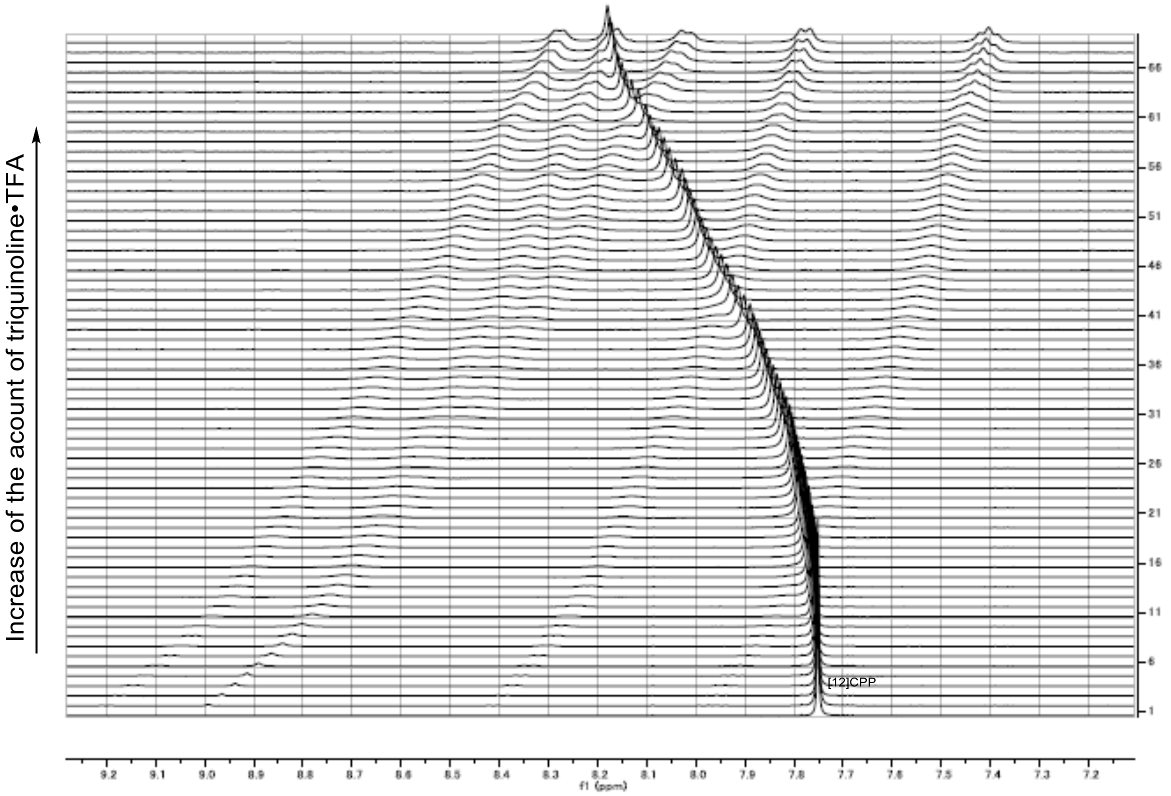

Supplementary Fig. 33 ^{1}H NMR titration of TQ•TFA **10** and [12]CPP in d_6-DMSO.

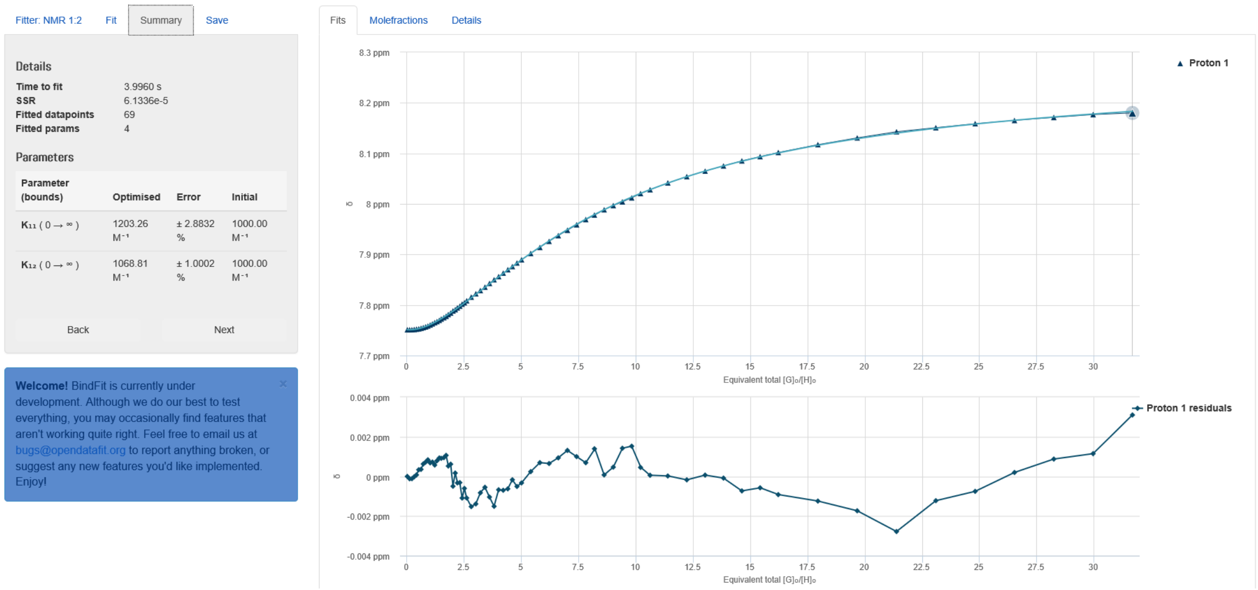

Supplementary Fig. 34 Non-linear regression analysis of ^{1}H NMR titration data.

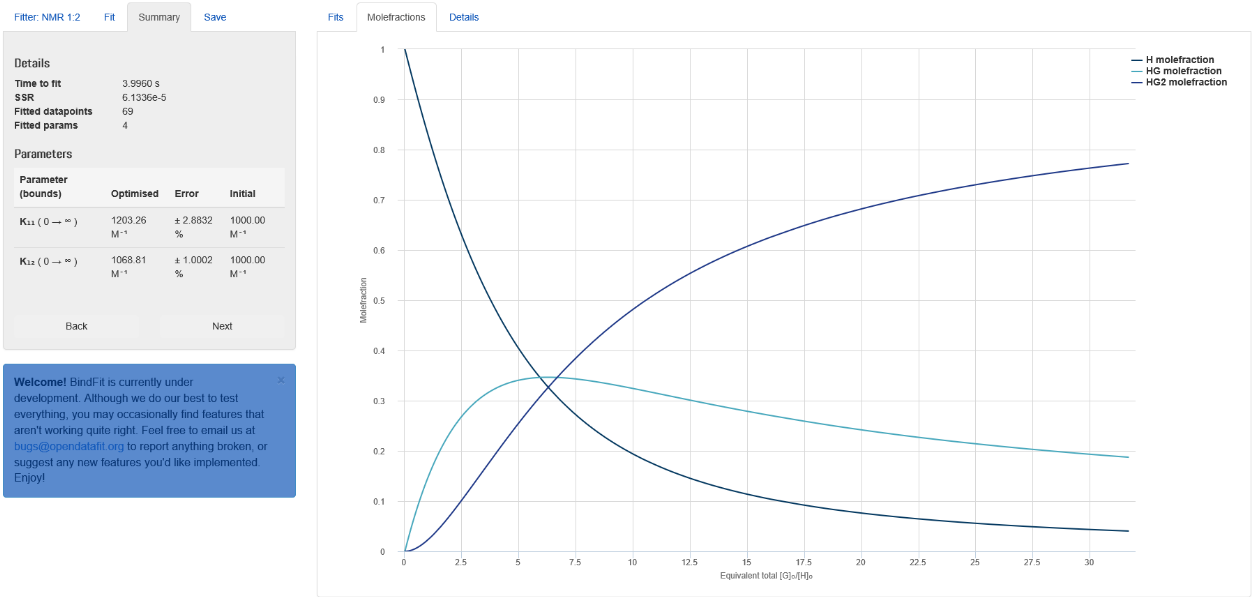

Supplementary Fig. 35 Simulated molefraction of 1:1 inclusion complex [12]CPP⊃TQ•H^+ **15** and 1:2 inclusion complex [12]CPP⊃$(TQ•H^+)_2$ **16** as a function of added amount of TQ•TFA **10** based on the obtained association constants.

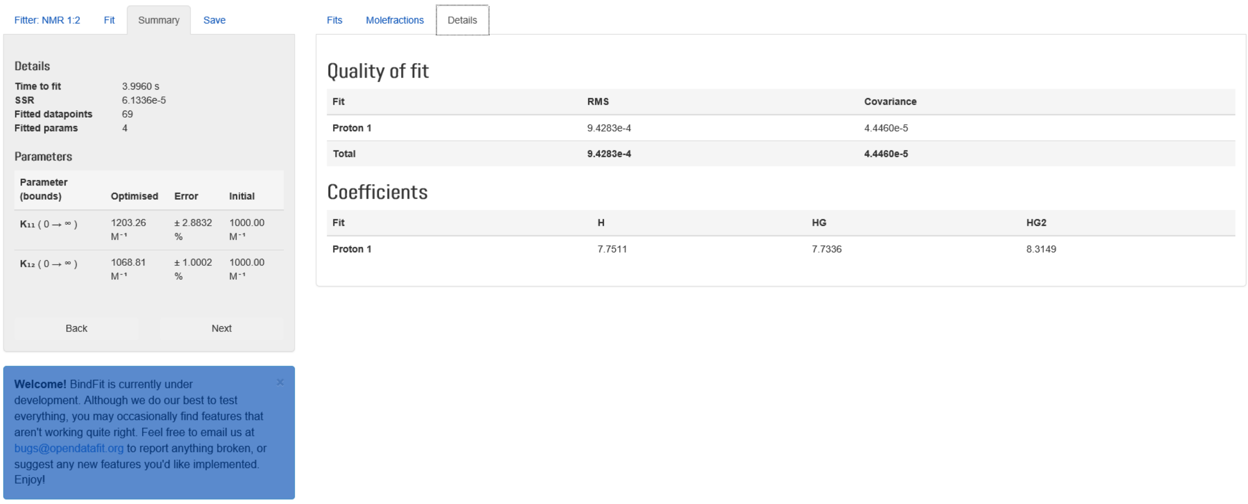

Supplementary Fig. 36 Quality of the fitting.

*7-2. Complexation of TQ•TFA **10** and coronene.*

To a d_6-DMSO solution of coronene (1.84 x 10^{-4} M, 530 μL) was incrementally added a d_6-DMSO solution of TQ•TFA **10** (146.6 x10^{-4} M) and analyzed by ^{1}H NMR (300K). The obtained data was fitted to 1:2 binding model, indicating that the formation of 1:1 complex coronene/TQ•H^+ **14** and 1:2 complex coronene/$(TQ•H^+)_2$ with association constants of K_1 = (1.62 ± 0.009) x 10^3 M^{-1} and K_2 = (0.11 ± 0.001) x 10^3 M^{-1}, respectively (Supplementary Fig. 37–40).

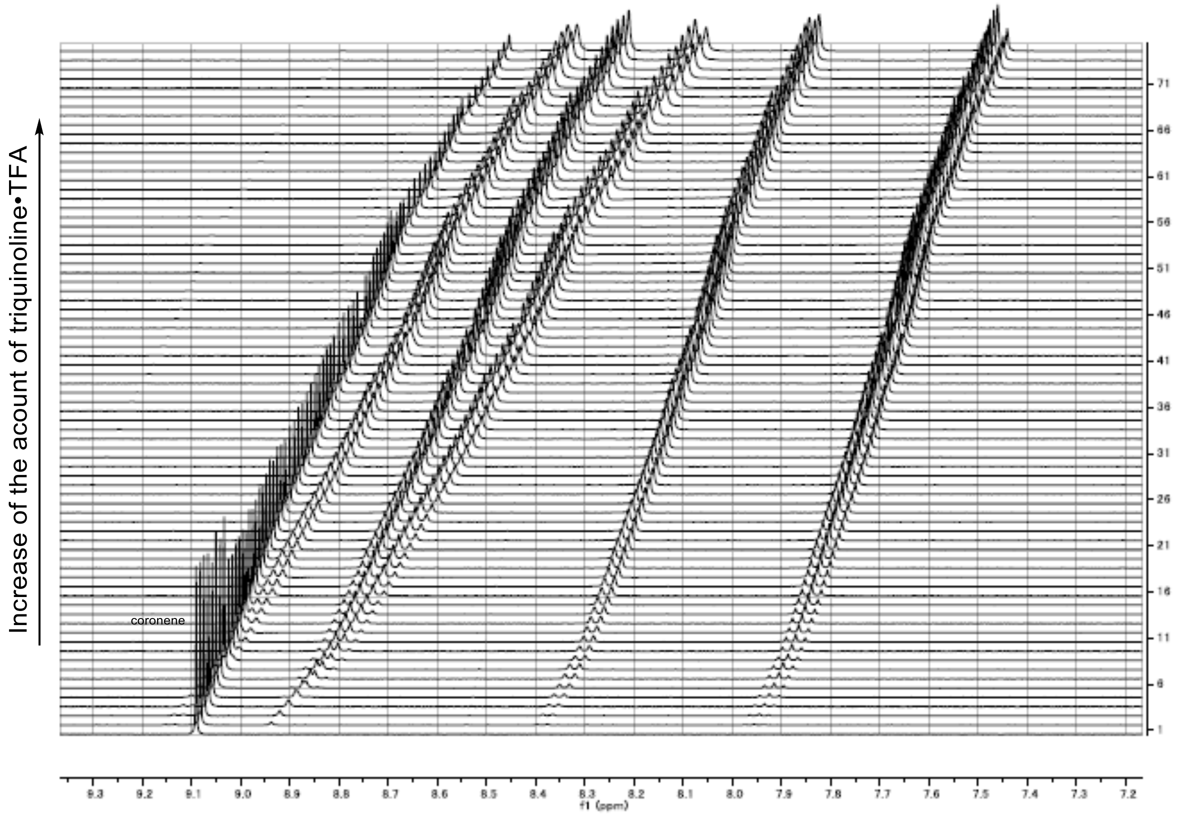

Supplementary Fig. 37 ^{1}H NMR titration of TQ•TFA **10** and coronene in *d*6-DMSO.

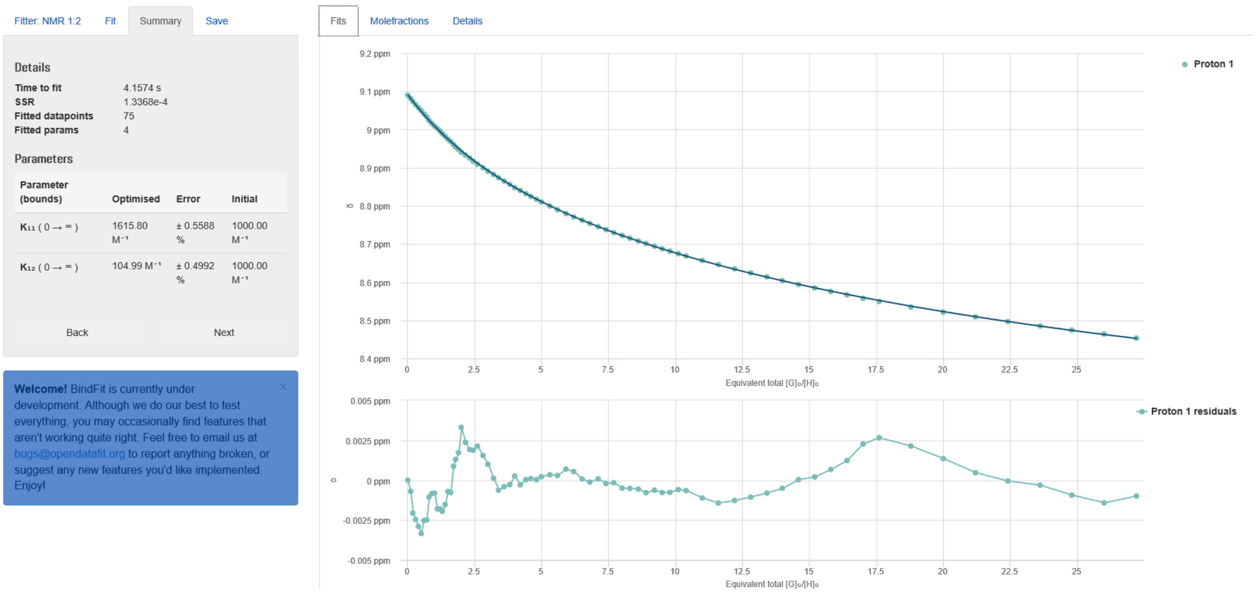

Supplementary Fig. 38 Non-linear regression analysis of ^{1}H NMR titration data.

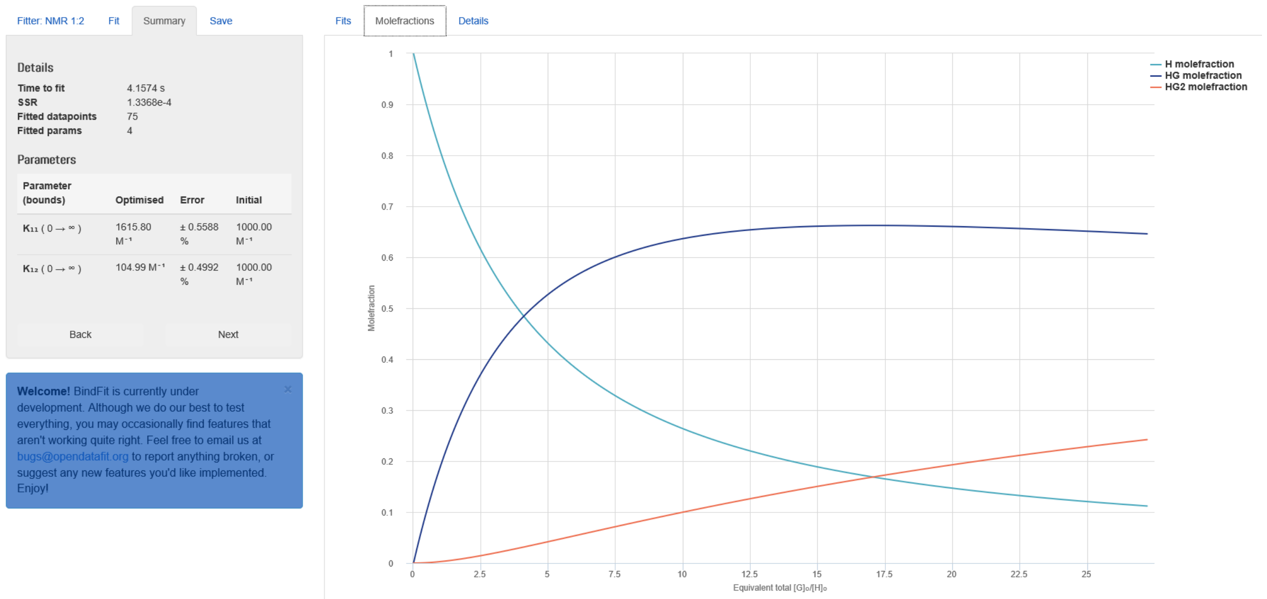

Supplementary Fig. 39 Simulated molefraction of 1:1 complex coronen/TQ•H$^+$ **14** and 1:2 complex coronene/(TQ•H$^+$)$_2$ as a function of added amount of TQ•TFA **10** based on the obtained association constants.

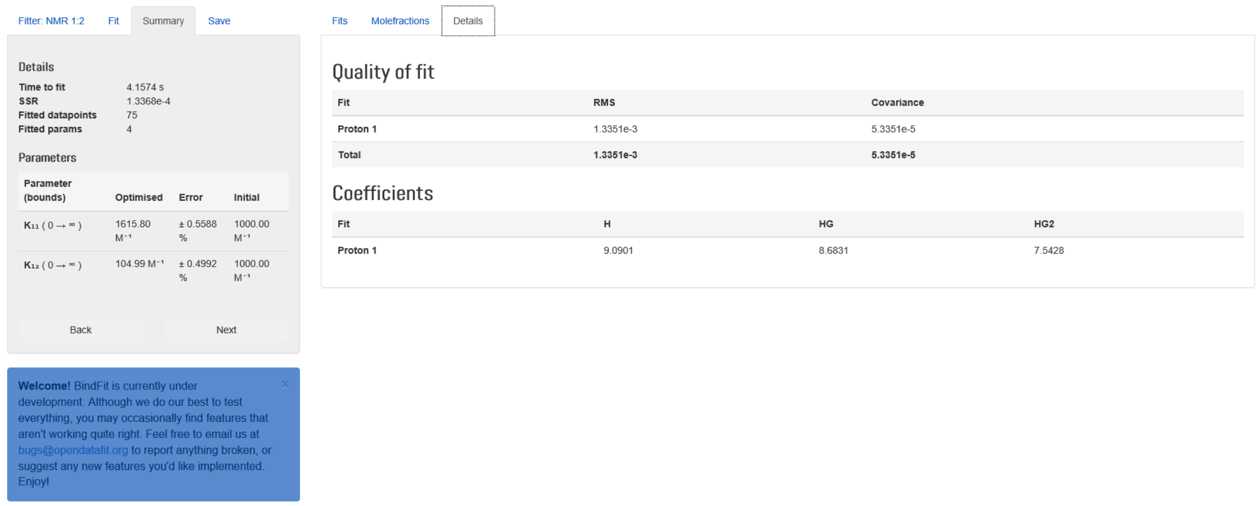

Supplementary Fig. 40 Quality of the fitting.

8. ^{1}H NMR Spectra of Mixtures Containing CPP

8-1. ^{1}H NMR spectrum of a mixture of DQ-Im•TFA **9** and [12]CPP.

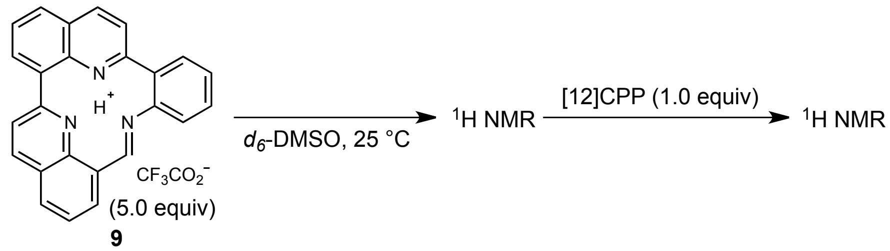

DQ-Im•TFA **9** (0.26 mg, 0.548 μmol, 5.0 equiv) and *d*6-DMSO (596 μL) were added to a 5 mm NMR tube. After being sealed with an nmr tube Teflon cap, the nmr tube was inverted several times and was placed into an NMR spectrometer to perform ^{1}H NMR analysis. To the solution in the NMR tube was then added [12]CPP (0.10 mg, 0.11 μmol, 1.0 equiv), and again the ^{1}H NMR analysis was performed. According to the absence of chemical shift change, [12]CPP and DQ-Im•TFA **9** did not form an inclusion complex (Supplementary Fig. 41).

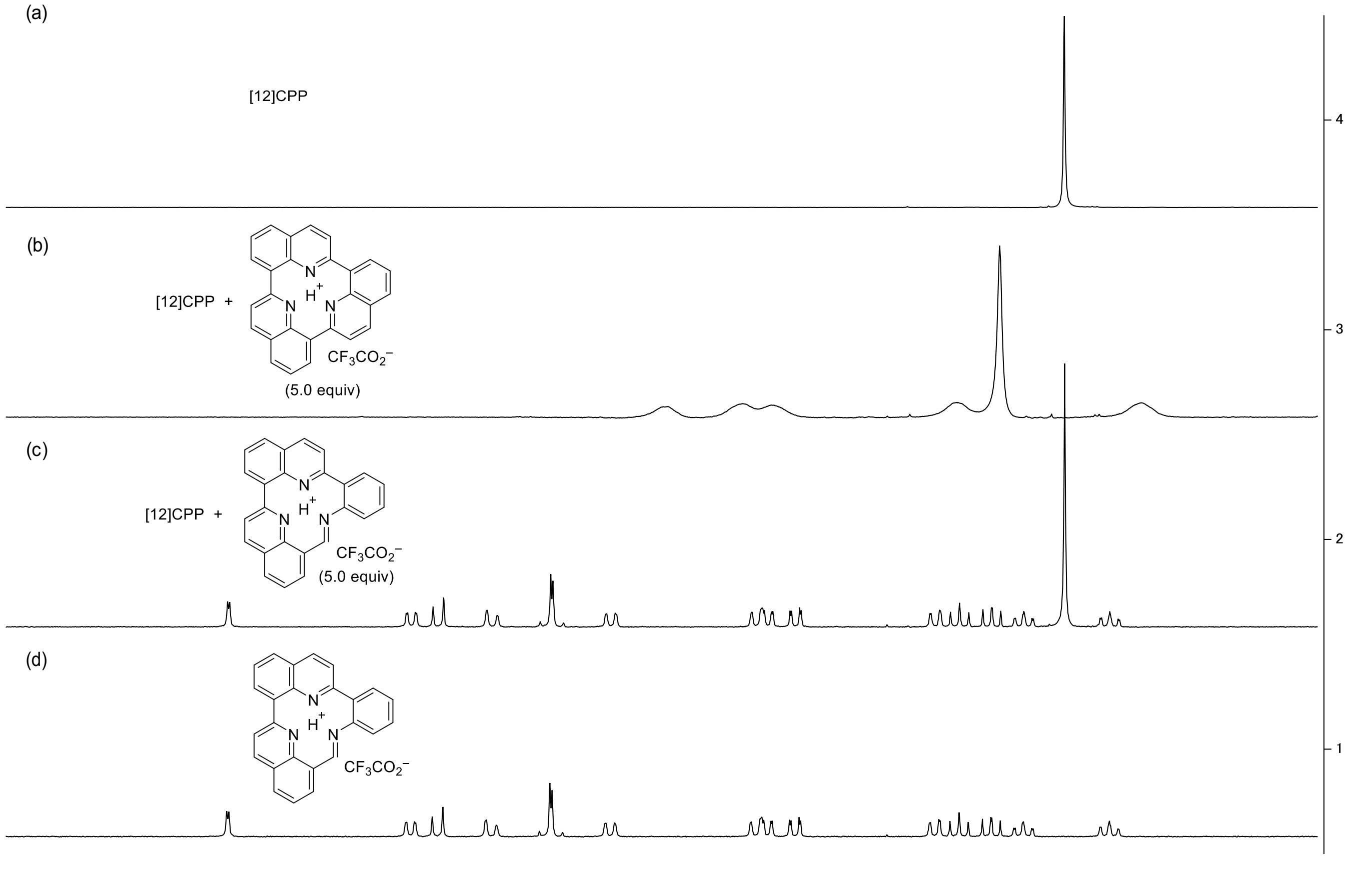

Supplementary Fig. 41 ^{1}H NMR Spectra (*d*6-DMSO) (a) [12]CPP (for comparison), (b) [12]CPP + TQ•TFA **10** (5.0 equiv) (for comparison), (c) [12]CPP + DQ-Im•TFA **9** (5.0 equiv), and (d) DQ-Im•TFA **9** (before the addition of [12]CPP).

*8-2. ¹H NMR spectrum of a mixture of **S5**•TFA and [12]CPP.*

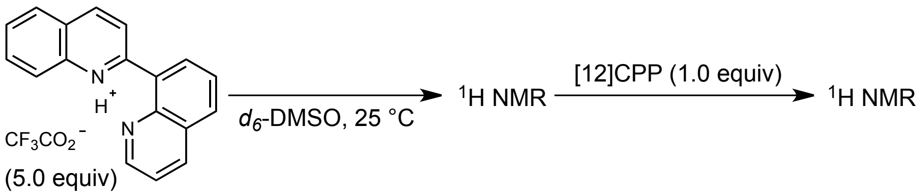

S5•TFA

2,8'-Biquinoline•TFA (**S5**•TFA) (0.20 mg, 0.548 μmol, 5.0 equiv) and d_6-DMSO (596 μL) were added to a 5 mm NMR tube. After being sealed with a Teflon cap, the nmr tube was inverted several times and was placed into an NMR spectrometer to perform ¹H NMR analysis. To the solution in the NMR tube was then added [12]CPP (0.10 mg, 0.11 μmol, 1.0 equiv), and again the ¹H NMR analysis was performed. According to the absence of chemical shift change, [12]CPP and **S5**•TFA did not form an inclusion complex (Supplementary Fig. 42).

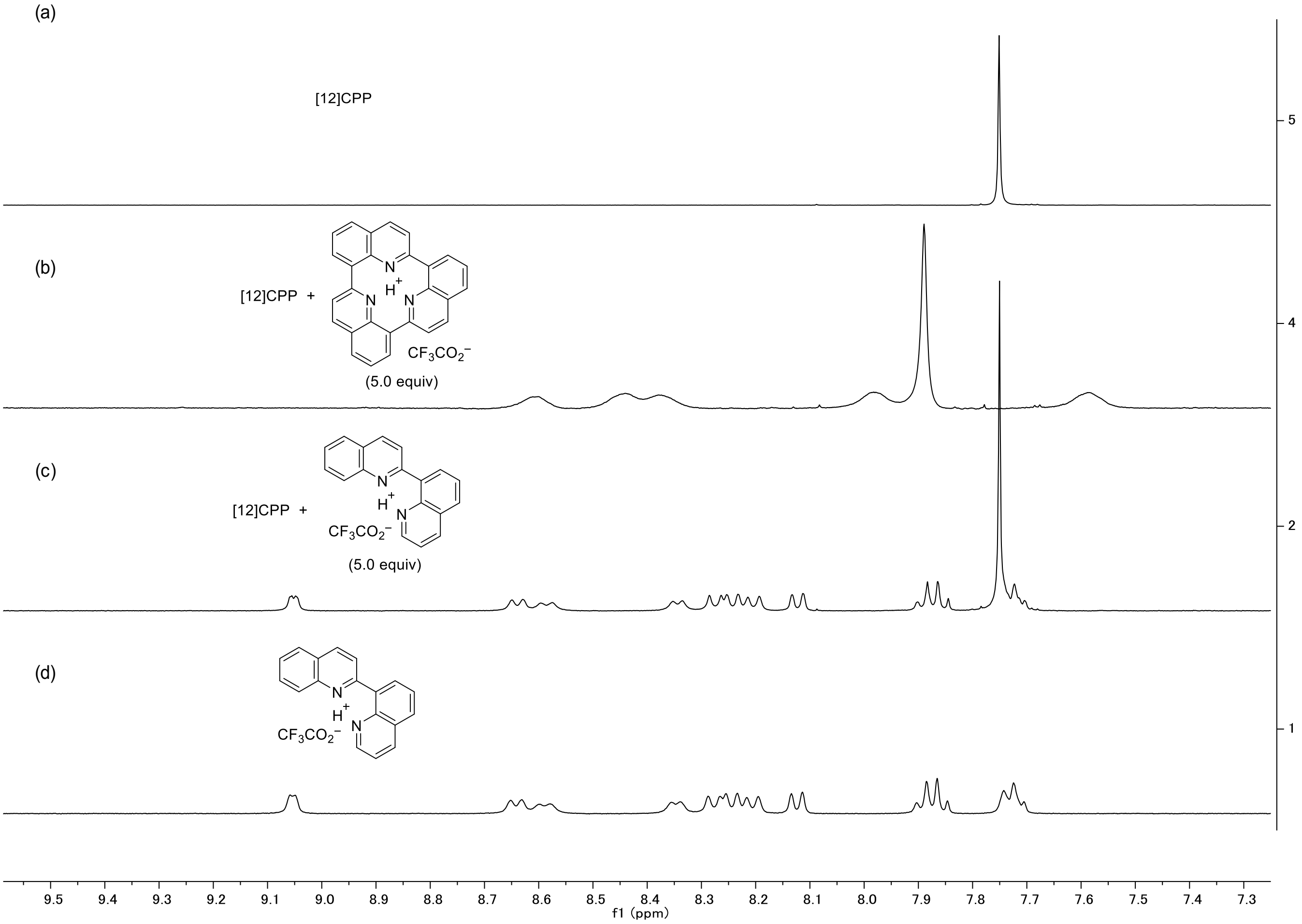

Supplementary Fig. 42 ¹H NMR Spectra (d_6-DMSO) (a) [12]CPP (for comparison), (b) [12]CPP + TQ•TFA **10** (5.0 equiv) (for comparison), (c) [12]CPP + 2,8'-biquinoline•TFA (**S5**•TFA) (5.0 equiv), and (d) 2,8'-biquinoline•TFA (**S5**•TFA) (before the addition of [12]CPP).

8-3. ^{1}H NMR spectrum of a mixture of 2-hydroxylethyl-TQ **12** and [12]CPP.

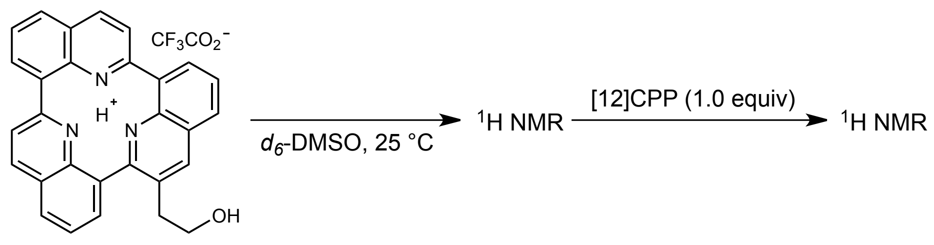

2-Hydroxylethyl-TQ•TFA **12** (0.30 mg, 0.548 μmol, 5.0 equiv) and *d*6-DMSO (596 μL) were added to a 5 mm NMR tube. After being sealed with a Teflon cap, the nmr tube was inverted several times and was placed into an NMR spectrometer to perform ^{1}H NMR analysis. To the solution in the NMR tube was then added [12]CPP (0.10 mg, 0.110 μmol, 1.0 equiv), and again the ^{1}H NMR analysis was performed. According to the absence of chemical shift change, [12]CPP and 2-hydroxyethyl-TQ•TFA **12** did not form an inclusion complex (Supplementary Fig. 43).

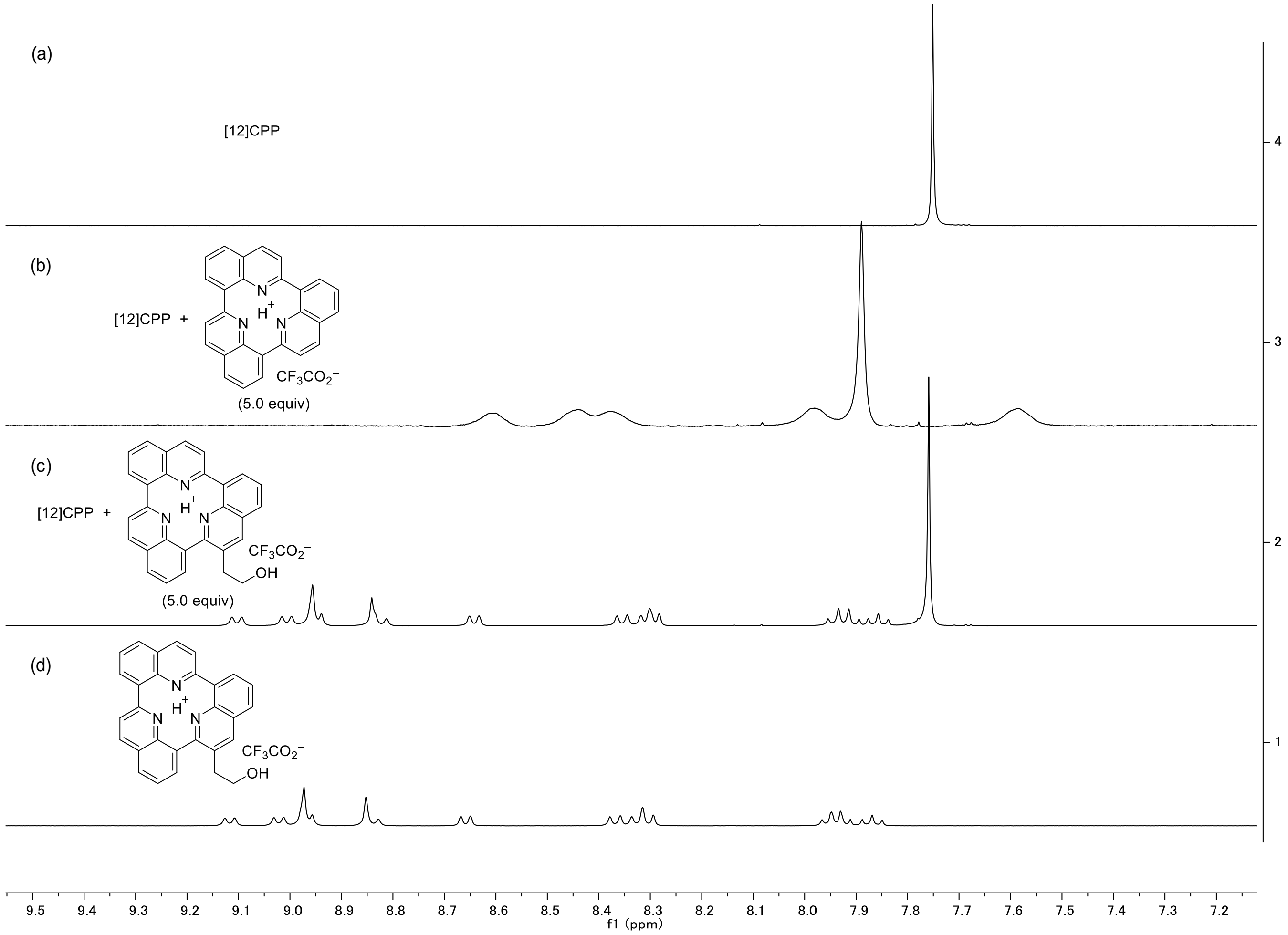

Supplementary Fig. 43 ^{1}H NMR Spectra (*d*6-DMSO) (a) [12]CPP (for comparison), (b) [12]CPP + TQ•TFA **10** (5.0 equiv) (for comparison), (c) [12]CPP + 2-hydroxylethyl-TQ•TFA **12** (5.0 equiv), and (d) 2-hydroxylethyl-TQ•TFA **12** (before the addition of [12]CPP).

*8-4. ¹H NMR spectrum of a mixture of TQ•TFA **10** and [10]CPP.*

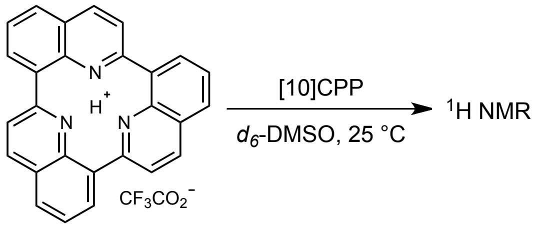

To a d_6-DMSO solution of TQ•TFA **10** (9.16 x 10^{-4} M, 530 μL) was incrementally added [10]CPP and analyzed by ^{1}H NMR (300K). According to the absence of chemical shift change, [10]CPP and TQ•TFA **10** did not form an inclusion complex (Supplementary Fig. 44).

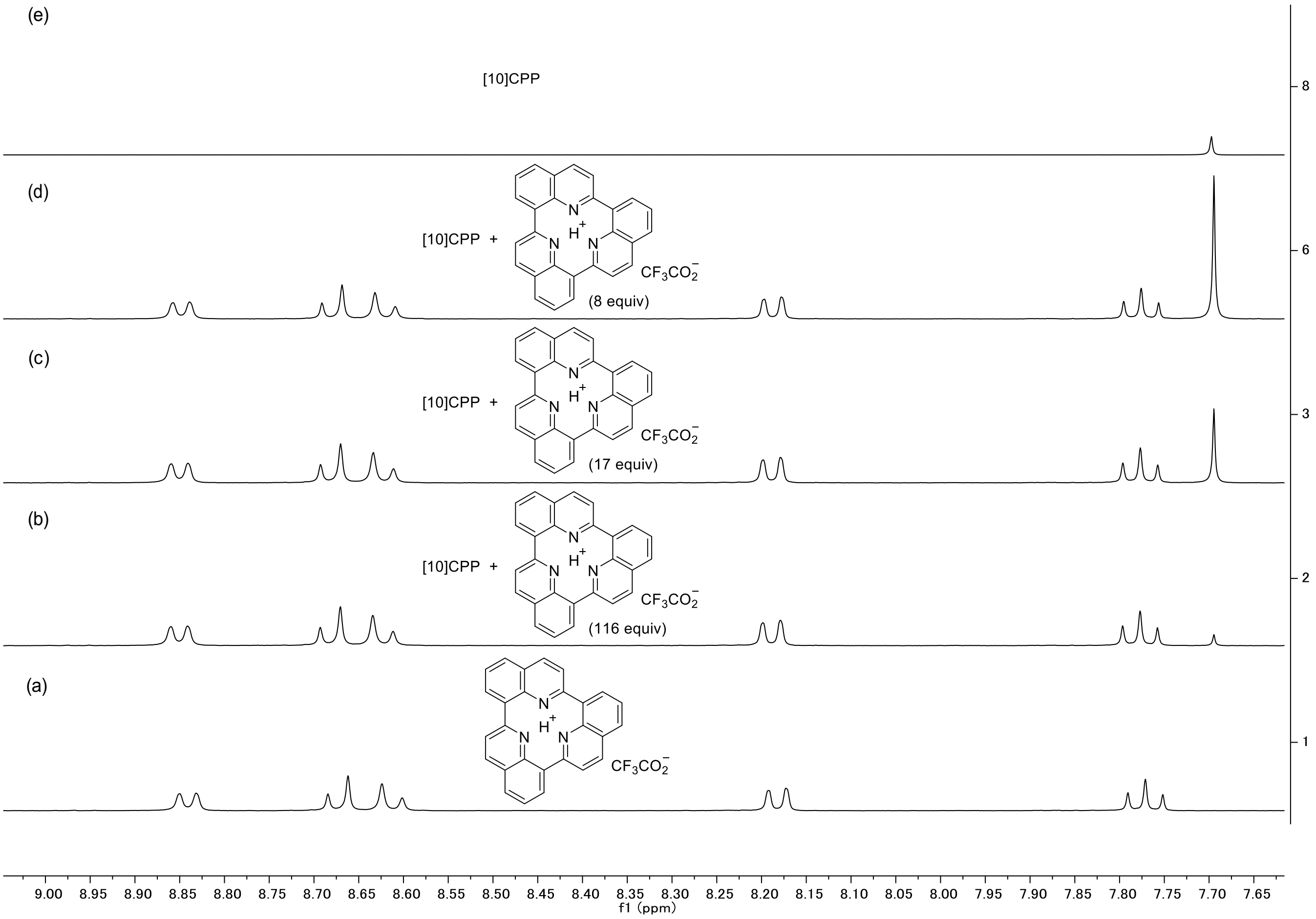

Supplementary Fig. 44 ^{1}H NMR Spectra (d_6-DMSO) (a) TQ•TFA **10** (before the addition of [10]CPP), (b) [10]CPP + TQ•TFA **10** (116 equiv), (c) [10]CPP + TQ•TFA **10** (17 equiv), (d) [10]CPP + TQ•TFA **10** (8 equiv), and (e) [10]CPP (for comparison).

8-5. ^{1}H NMR spectrum of a mixture of coronene and [12]CPP.

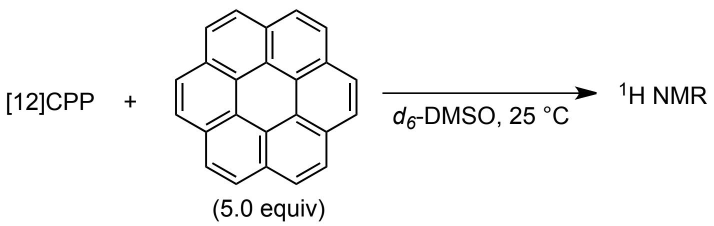

[12]CPP (0.30 mg, 0.333 μmol, 1.0 equiv) and d_6-DMSO (530 μL) were added to a 5 mm NMR tube. After being sealed with a Teflon cap, the nmr tube was inverted several times and was placed into an NMR spectrometer to perform ^{1}H NMR analysis. To the solution in the NMR tube was then added coronene (0.10 mg, 0.333 mmol, 1.0 equiv), and again the ^{1}H NMR analysis was performed. According to the absence of chemical shift change, [10]CPP and TQ•TFA **10** did not form an inclusion complex (Supplementary Fig. 45).

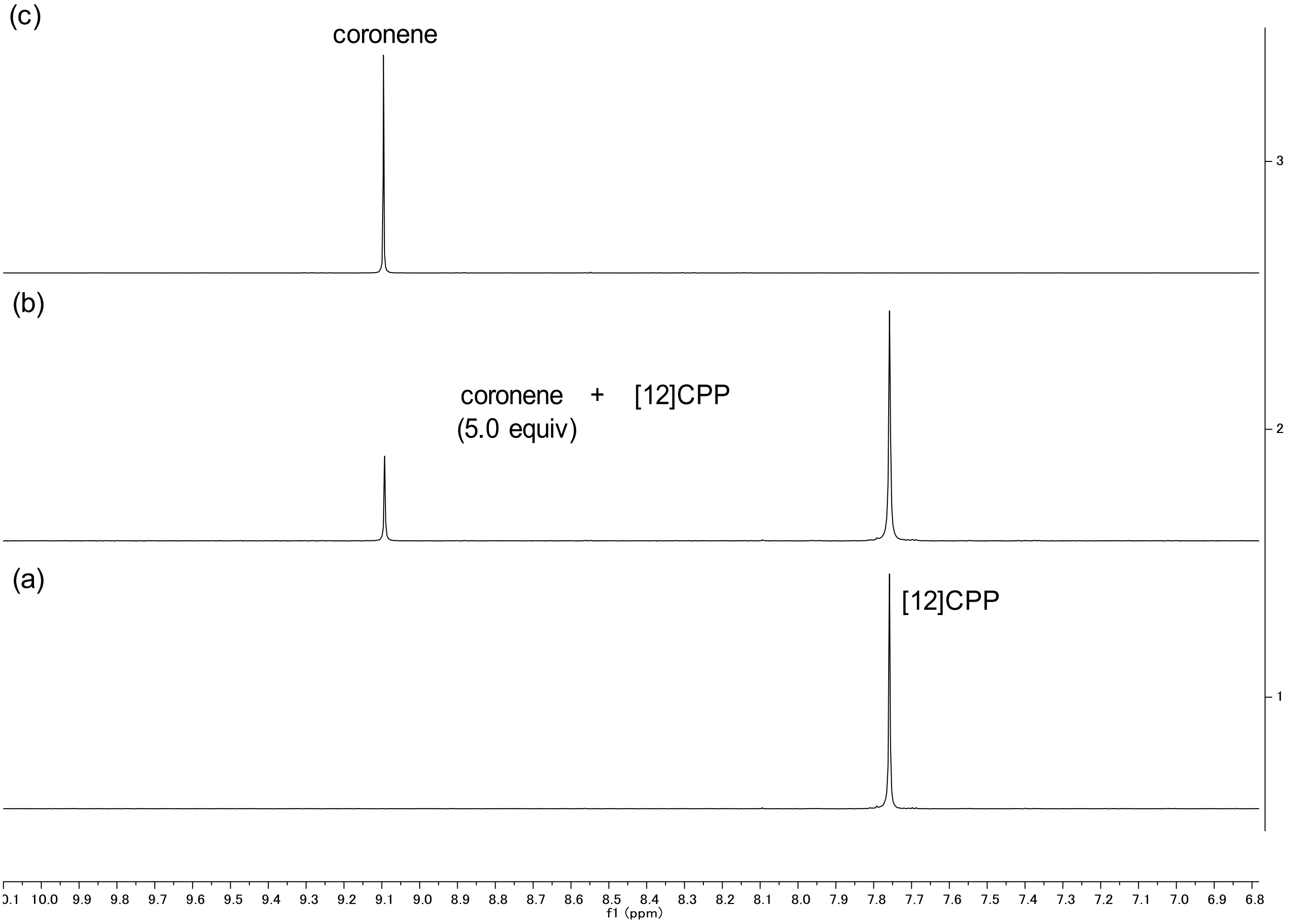

Supplementary Fig. 45 ^{1}H NMR Spectra (d_6-DMSO): (a) [12]CPP (before the addition of coronene), (b) [12]CPP + coronene (5.0 equiv), (c) coronene (for comparison).

9. Fluorescence Quenching Experiment

*9-1. Addition of TQ•TFA **10** to [12]CPP.*

Fluorescence spectrum of [12]CPP in DMSO (0.20 μM, 2 mL) was first measured (excitation at λ = 370 nm), and then to the solution was added a DMSO solution of TQ•TFA **10** (2.0 x 10^2 μM) incrementally. The changes in the fluorescent intensity were shown in Supplementary Fig. 46. Concentration-dependent decrease in fluorescent intensity corresponds to intermolecular interaction of 12CPP and TQ•TFA **10**.

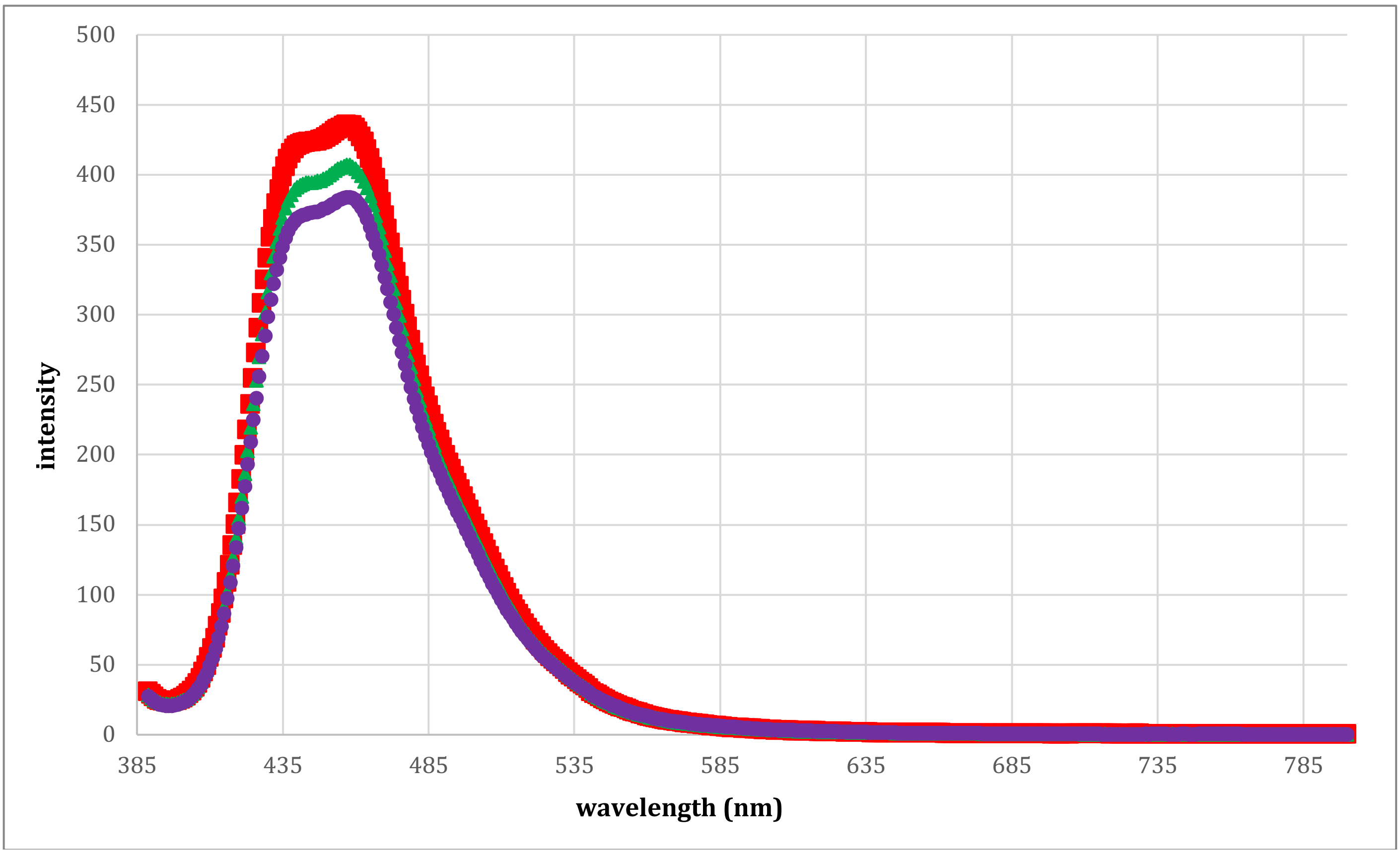

Supplementary Fig. 46 Fluorescence spectra of DMSO solutions of [12]CPP in the absence or presence of TQ•TFA **10**; (red) [12]CPP only, (green) [12]CPP + TQ•TFA **10** (2.5 equiv), and (purple) [12]CPP + TQ•TFA **10** (5.0 equiv).

*9-2. Addition of DQ-Im·TFA **9** to [12]CPP.*

Fluorescence spectrum of [12]CPP in DMSO (0.20 μM, 2 mL) was first measured (excitation at λ = 370 nm), and then to the solution was added a DMSO solution of DQ-Im•TFA **9** (2.0 x 10^2 μM) incrementally. The changes in the fluorescent intensity were shown in Supplementary Fig. 47. Almost superimposed spectra indicate the absence of intermolecular interaction (very weak if any).

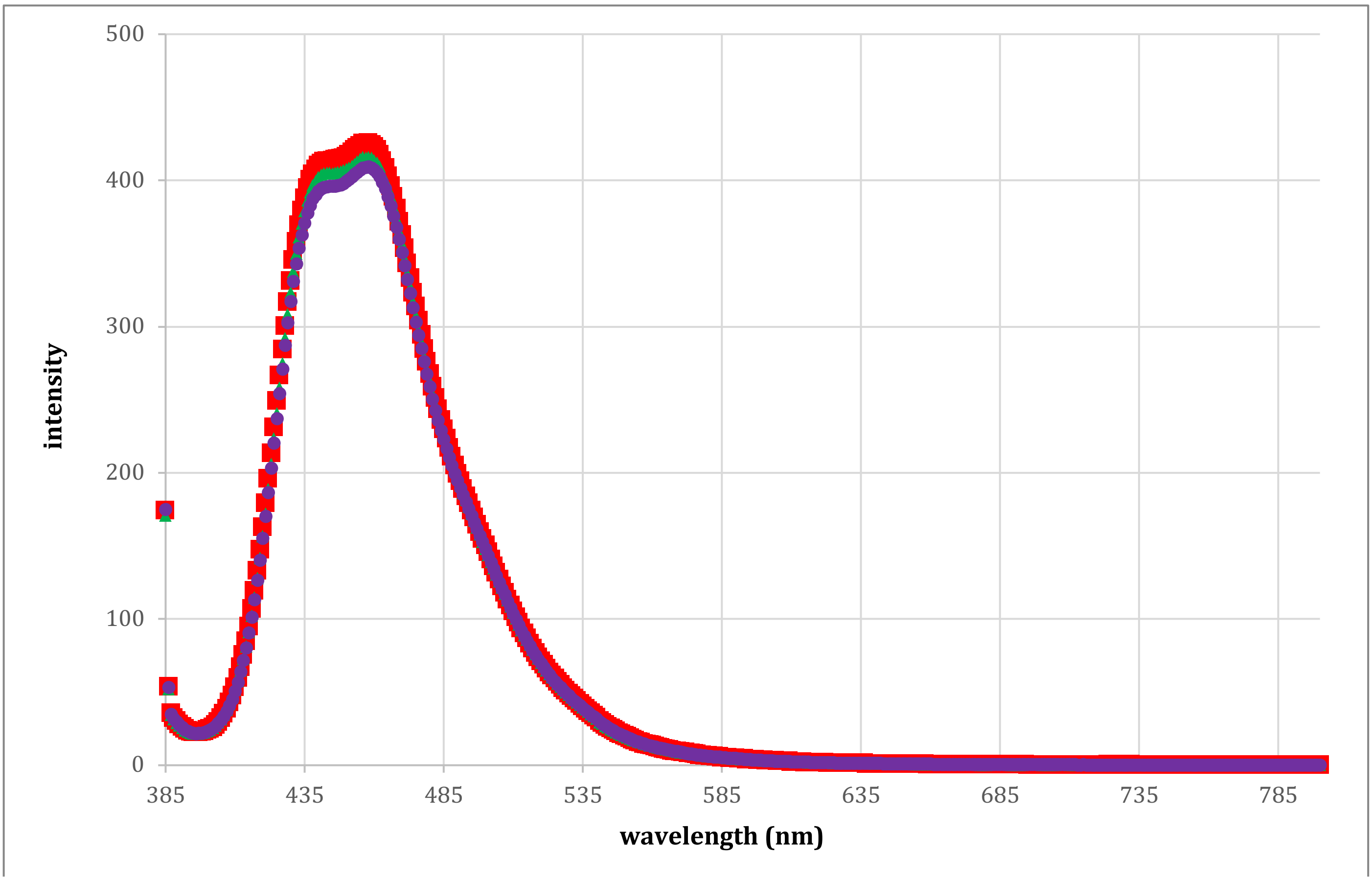

Supplementary Fig. 47 Fluorescence spectra of DMSO solution of [12]CPP in the absence or presence of DQ-Im•TFA **9**; (Rred) [12]CPP only, (green) [12]CPP + DQ-Im•TFA **9** (2.5 equiv), and (purple) [12]CPP + DQ-Im•TFA **9** (5.0 equiv).

*9-3. Addition of 2,8′-biquinoline **S5**•TFA to [12]CPP.*

Fluorescence spectrum of [12]CPP in DMSO (0.20 μM, 2 mL) was first measured (excitation at λ = 370 nm), and then to the solution was added a DMSO solution of 2,8′-biquinoline **S5**•TFA (2.0 x 10^2 μM) incrementally. The changes in the fluorescent intensity were shown in Supplementary Fig. 48. Superimposed spectra indicate the absence of intermolecular interaction.

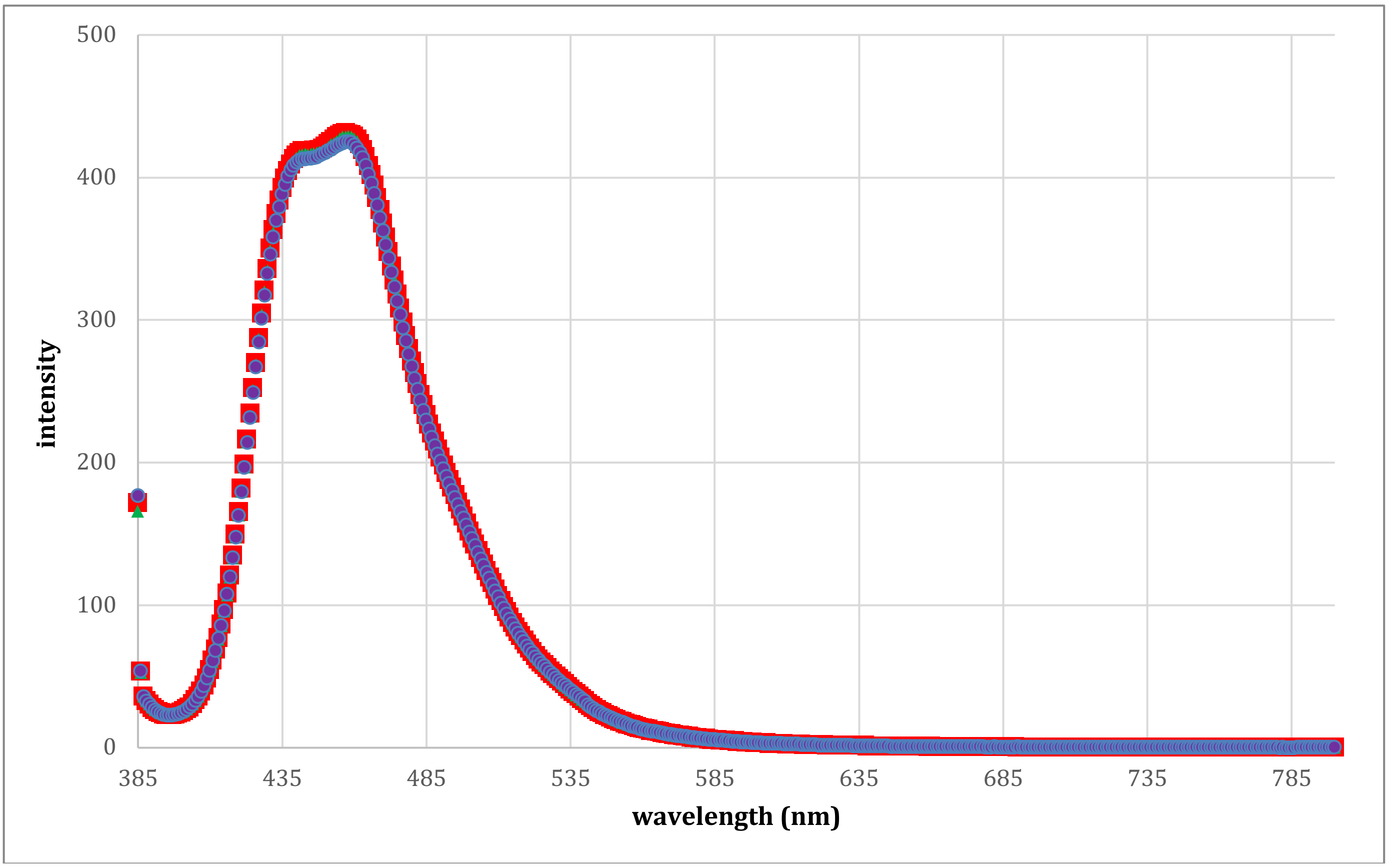

Supplementary Fig. 48 Fluorescence spectra of DMSO solution of [12]CPP in the absence or presence of **S5**•TFA; (red) [12]CPP only, (green) [12]CPP + **S5**•TFA (2.5 equiv), and (purple) [12]CPP + **S5**▪TFA (5.0 equiv).

*9-4. Addition of 2-hydroxylethyl-TQ•TFA **12** to [12]CPP.*

Fluorescence spectrum of [12]CPP in DMSO (0.20 μM, 2 mL) was first measured (excitation at λ = 370 nm), and then to the solution was added a DMSO solution of 2-hydroxylethyl-TQ•TFA **12** (2.0 x 10^2 μM) incrementally. The changes in the fluorescent intensity were shown in Supplementary Fig. 49. Based on NMR experiments that indicate no intermolecular interaction, the hydroxyl group of **12** might affect the fluorescent intensity of 12CPP.

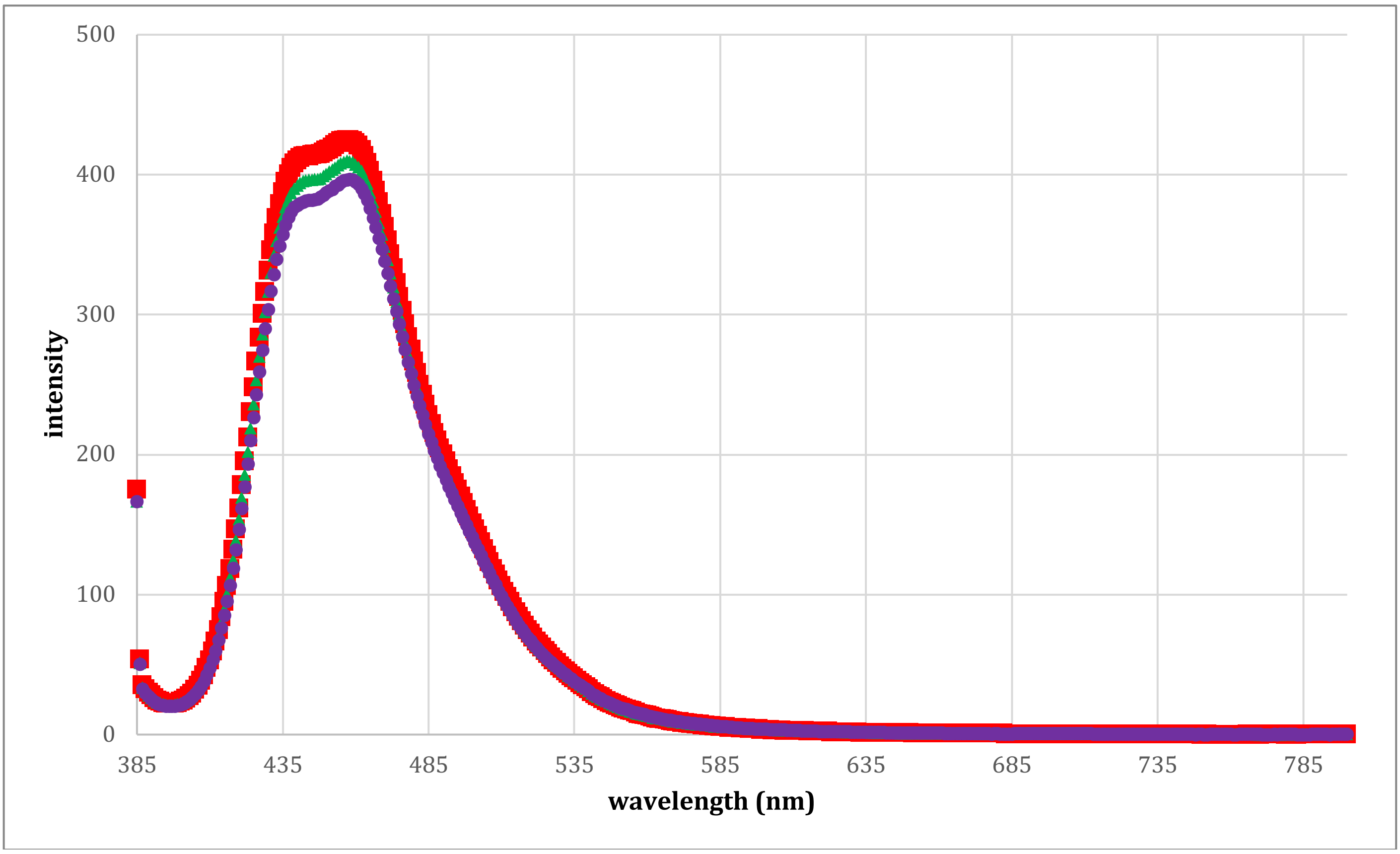

Supplementary Fig. 49 Fluorescence spectra of DMSO solution of [12]CPP in the absence or presence of 2-hydroxylethyl-TQ•TFA **12**; (red) [12]CPP only, (green) [12]CPP + 2-hydroxylethyl-TQ•TFA **12** (2.5 equiv), and (purple) [12]CPP + 2-hydroxylethyl-TQ•TFA **12** (5.0 equiv).

10 Computational Study on Non-Stop Povarov Reaction of Diquinoline-Imine (DQ-Im)

10-1. General.

In terms of accuracy and computational cost, reactants were reasonably simplified (from DQ-Im•TFA **9** and *n*-butyl vinyl ether (BVE) to DQ-Im•HCl and methyl vinyl ether (MVE), respectively) and all the geometry optimization were carried out at B3LYP-D3/6-31G(d) level of theory. To implement the possible non-covalent interactions, correction with empirical dispersion was included.

10-2. Reaction profile.

Full energy landscape from DQ-Im•HCl and methyl vinyl ether (MVE) is shown in Supplementary Fig. 50. Sigle-point energies on each geometry were further evaluated at the ωB97XD/def2-TZVPP/SMD(acetonitrile) level of theory. Gibbs free energies (G) are given relative to starting materials. Stepwise formal cycloaddition of MVE is more energy-demanding (**TS1**, **TS2**: $\Delta G^‡$ = 20.5, 20.2 kcal mol^{-1}) that the following elimination (**TS3**, **TS4**: $\Delta G^‡$ = 14.7, 12.5 kcal mol^{-1}) and hydride transfer (TS: $\Delta G^‡$ = 7.1 kcal mol^{-1}) steps. In the initial addition step of MVE to DQ-Im•HCl, other three transition states (**TS1-a**, **TS1-b**, and **TS1-c**) were located (Supplementary Fig. 51). **TS1** and **TS1-c**, in which chloride anion is located close to imine C–H, are more likely pathway and similar in energy. Both of them lead to **INT1** in supplementary Fig. 50 and subsequent reaction proceeds as delineated.

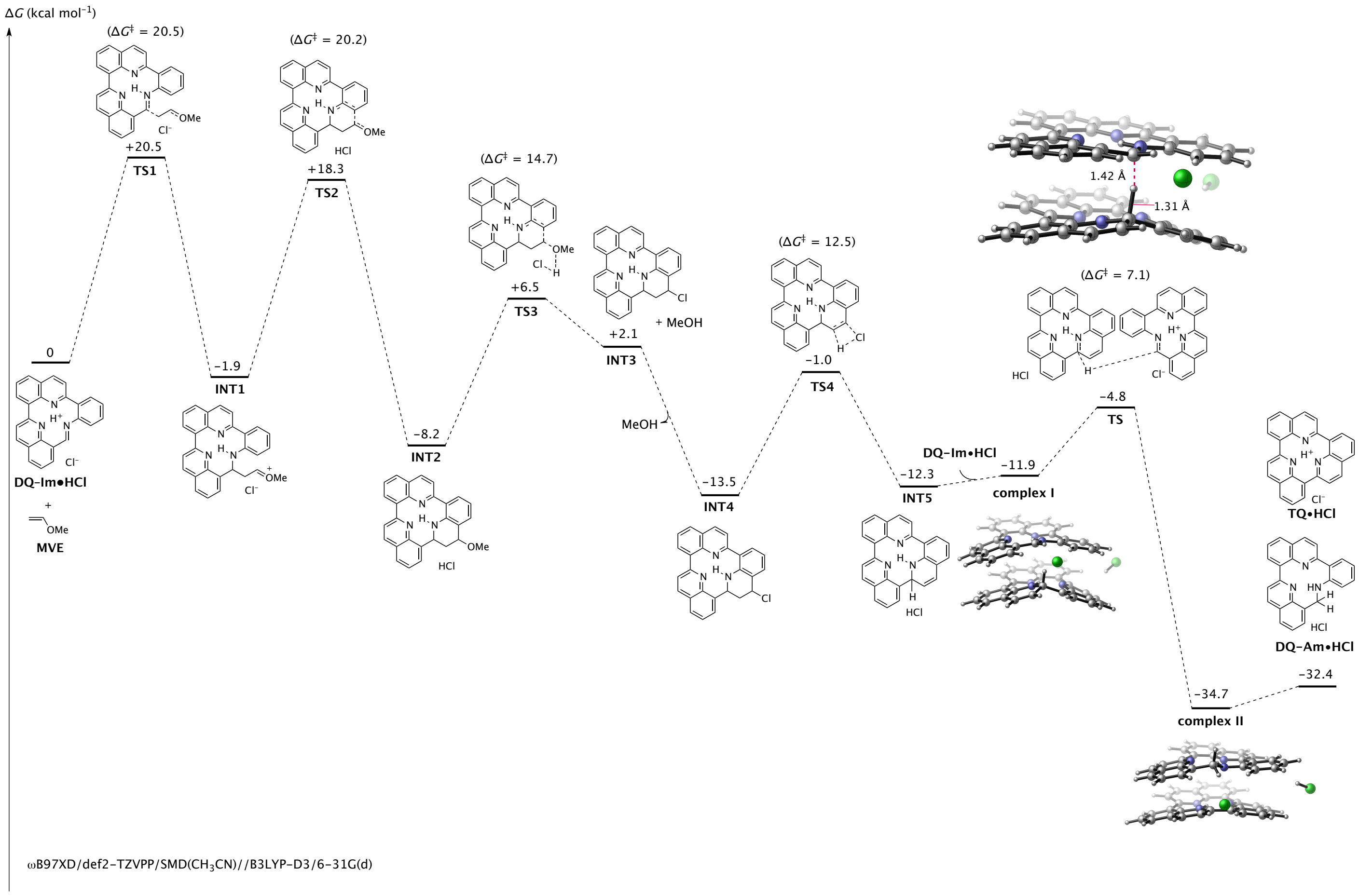

Supplementary Fig. 50 Reaction profile of non-stop Povarov reaction of DQ-Im•HCl and methyl vinyl ether (MVE) affording TQ•HCl.

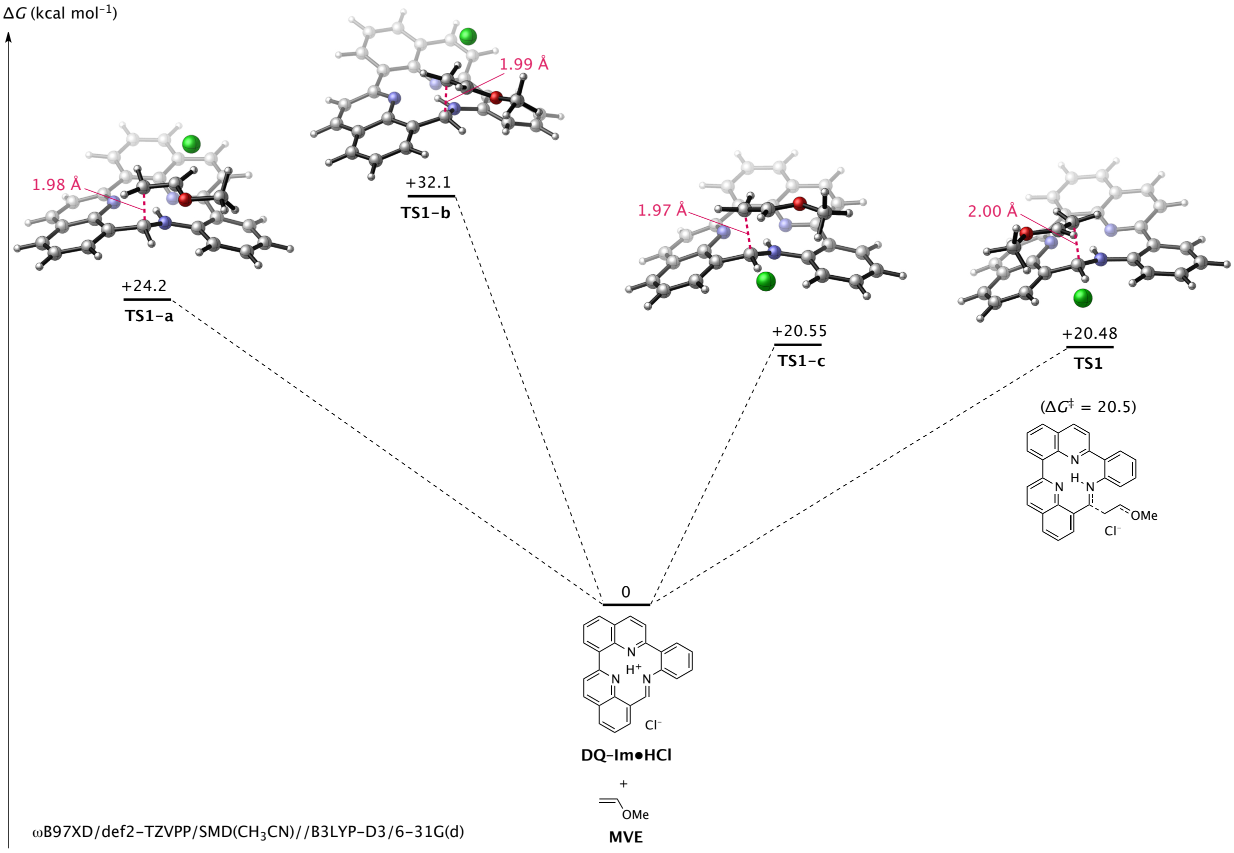

Supplementary Fig. 51 Located transition states for the initial MVE addition to DQ-Im•HCl.

<u>*10-3. Formation of π-complex I and II.*</u>

Counterpoise corrected interaction energies of complexes **I** and **II** were calculated at the ωB97XD/def2-TZVPP level in the gas phase (Supplementary Fig 52 and 53).

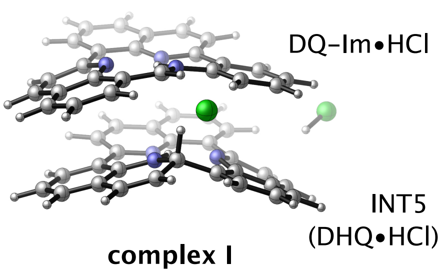

counterpoise corrected energy =	–3251.053221118559 hartree
BSSE energy =	0.003100975241 hartree
sum of fragments =	–3250.992688443432 hartree
complexation energy =	–39.93 kcal mol⁻¹ (raw)
complexation energy =	–37.98 kcal/mole (corrected)

$\Delta E_{\mathrm{deform}}$ (DQ-Im•HCl) = 1.49 kcal mol^{-1} ; $\Delta E_{\mathrm{deform}}$ (**INT5**) = 4.84 kcal mol^{-1}

ΔE_{ZPE} = 1.44 kcal mol^{-1}

ΔE_{bind} = $\Delta E_{\mathrm{deform}}$ (DQ-Im•HCl) + $\Delta E_{\mathrm{deform}}$ (**INT5**) + corrected $\Delta E_{\mathrm{complexation}}$ + ΔE_{ZPE} = –30.21 kcal mol^{-1}

Supplementary Fig. 52 Non-covalent association energy of DQ-Im•HCl and DHQ•HCl (**INT5**) into complex **I**.

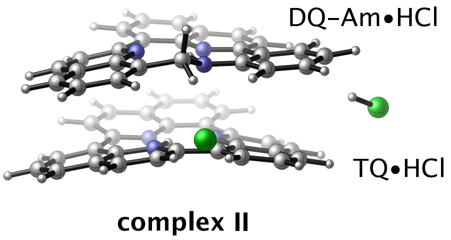

counterpoise corrected energy =	–3251.082090196266 hartree
BSSE energy =	0.003840917591 hartree
sum of fragments =	–3251.014582172565 hartree
complexation energy =	–44.77 kcal mol⁻¹ (raw)
complexation energy =	–42.36 kcal mol⁻¹ (corrected)

$\Delta E_{\mathrm{deform}}$ (DQ-Am•HCl) = 4.40 kcal mol^{-1} ; $\Delta E_{\mathrm{deform}}$ (TQ•HCl) = 1.62 kcal mol^{-1}

ΔE_{ZPE} = 1.87 kcal mol^{-1}

ΔE_{bind} = $\Delta E_{\mathrm{deform}}$ (DQ-Am•HCl) + $\Delta E_{\mathrm{deform}}$ (TQ•HCl) + corrected $\Delta E_{\mathrm{complexation}}$ + ΔE_{ZPE} = –34.48 kcal mol^{-1}

Supplementary Fig. 53 Non-covalent association energy of DQ-Am•HCl and TQ•HCl into complex **II**.

10-4. Distortion/interation analysis along IRC of TS (hydride transfer).

The intrinsic reaction coordinate from TS, involving hydride transfer of complex **I** from dihydroquinoline•HCl (DHQ•HCl) to DQ-Im•HCl (Fig. 2c, supplementary Fig. 50), was followed every 0.20 (for forward) or 0.10 (for backward) Bohr (stepsize = 20 or 10 for the Gaussian option, respectively). The IRC calculation was performed at the B3LYP-D3/6-31G(d) level of theory. At the designated steps were selected for analysis. Single point energy calculations were performed at the ωB97XD/def2-TZVPP/SMD(acetonitrile) level. The collected data are summarized in Supplementary Table 3 and Supplementary Fig. 54. During the hydride transfer, i.e. from complex I to step 10 in which newly formed C_{imine}–H bond is in a typical C_{sp3}–H bond length, the vertical distance between DQ-Im and DHQ is almost identical (longest: 3.290 Å, shortest: 3.270 Å, Δ = 0.020 Å), which corresponds to 2.0% of the traveling distance of hydride.

Supplementary Table 3 Energy and structural data. Energies are given in kcal mol^{-1}.

step	ΔE_{total}	ΔE_{dist}	ΔE_{int}	DHQ•HCl ΔE_{dist}	DHQ•HCl C–H (Å)	DQ-Im•HCl ΔE_{dist}	DQ-Im•HCl C–H (Å)	[b]distance between DHQ and DQ-Im (Å)	traveling distance of hydride[c] (Å)
**I**[a]					1.077		2.160	3.277	
–20	3.682	2.118	1.564	0.024	1.123	2.094	1.832	3.290	0.172
–15	4.512	2.609	1.904	0.257	1.128	2.351	1.776	3.276	0.202
–10	5.683	3.578	2.105	0.836	1.138	2.742	1.706	3.268	0.242
–5	7.408	6.271	1.137	2.535	1.159	3.736	1.612	3.268	0.299
-4	7.905	7.690	0.215	3.336	1.171	4.354	1.586	3.269	0.318
–3	8.460	9.748	-1.288	4.466	1.187	5.282	1.558	3.268	0.341
–2	9.066	12.927	-3.861	5.901	1.218	7.026	1.519	3.269	0.376
–1	9.521	17.036	-7.515	7.551	1.257	9.485	1.474	3.269	0.418
TS	9.569	22.136	-12.567	8.939	1.311	13.197	1.416	3.270	0.474
1	7.833	33.569	-25.735	11.913	1.423	21.655	1.304	3.271	0.586
2	4.166	45.147	-40.981	15.337	1.529	29.811	1.207	3.274	0.687
3	0.371	55.137	-54.767	19.228	1.611	35.909	1.146	3.277	0.759
4	-2.773	62.731	-65.504	22.691	1.666	40.04	1.129	3.280	0.795
5	-5.570	69.538	-75.107	25.789	1.727	43.748	1.111	3.282	0.834
10	-14.246	86.793	-101.039	35.536	2.019	51.257	1.097	3.289	0.987

[a]**I** denotes complex **I**. [b]Vertical distance of DHQ plane and DQ-Im plane, defined by the distance of hydrogens located at the center of each molecule. [c]With respect to complex I, the averaged value of elongated C–H bond length of DHQ and shortened C_{imine}–H distance of DQ-Im.

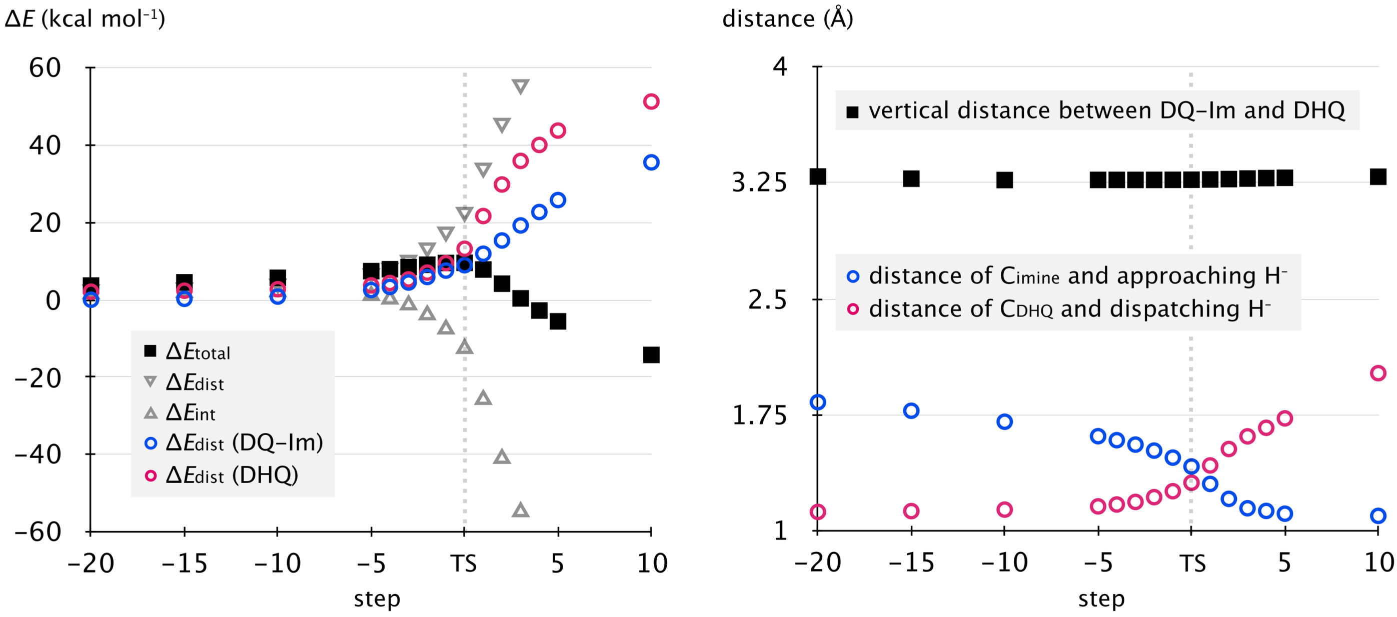

Supplementary Fig. 54 Distortion/interaction energies in the hydride transfer reaction and structural analysis.

11. Homodesmotic Reaction of TQ

All calculations were performed at the ωB97XD/def2-TZVPP//B3LYP-D3/6-31G(d,p) level of theory. Opened structures gave local minima with no imaginary frequency. Cartesian geometry of the optimized structures is provided in Section 18. Homodesmotic reaction of TQ and TQ•H+ with three biphenyl molecules showed large energetic disparity (Supplementary Fig. 55).

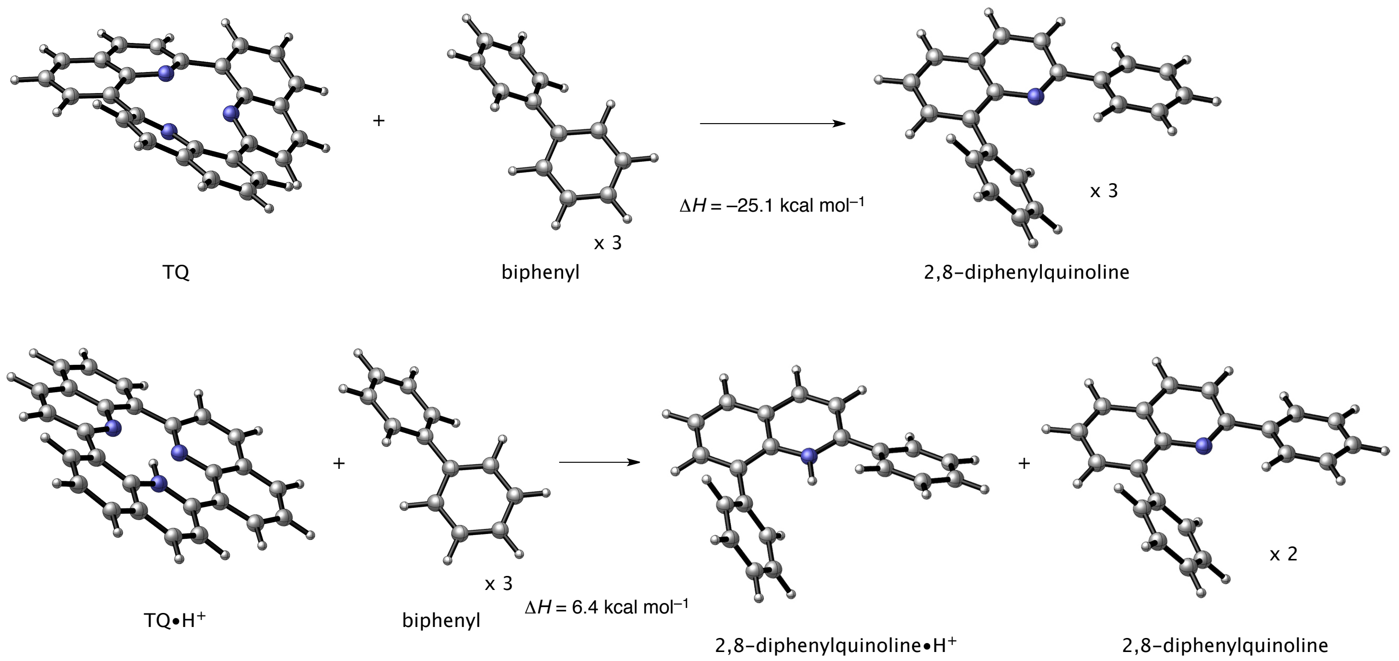

Supplementary Fig. 55 Homodesmotic reaction of TQ and TQ•H⁺ to 2,8-diphenyl quinoline fragments.

Another homodesmotic reactions to opened-TQ with diphenyl endcaps were calculated, resulting in similar energetic disparity (Supplementary Fig 56). These opened forms may contain conformational factors and homodesmotic reactions considered above are more reliable. For opened-TQ•H+, bidentate coordination to proton is unlikely due to structural constraint.

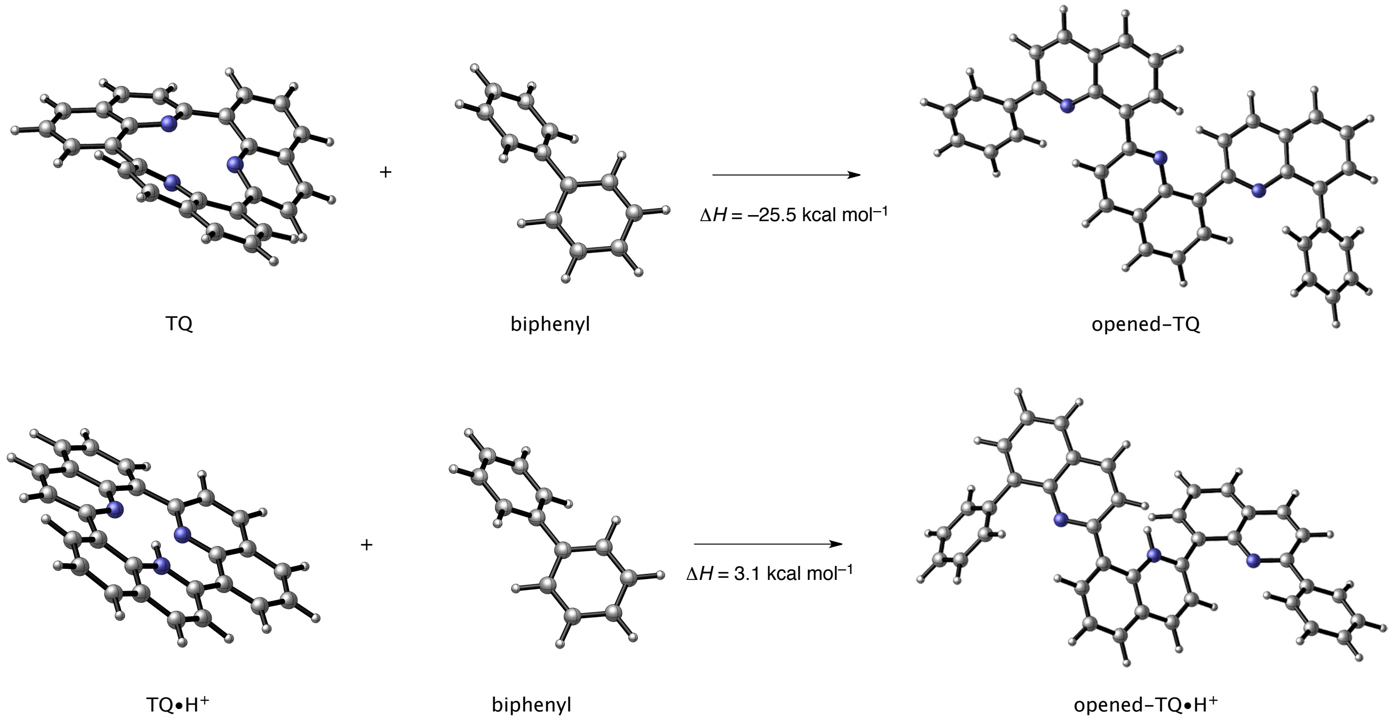

Supplementary Fig. 56 Homodesmotic reaction of TQ and TQ•H⁺ to opened-TQ with diphenyl endcaps.

12. Proton Affinity of TQ

Proton affinity (PA) is defined by the negative of the molar enthalpy change ($-\Delta H$) of the reaction: TQ + H+ → $TQ\bullet H^+$ at 298.15 K in gas phase.[10] By assuming the ideal gas behavior, $\Delta H = \Delta E - RT$. The required thermodynamic properties were obtained from the electronic structure calculations at the B3LYP/6-311+G(2df,p)//B3LYP/6-31G(d) level of theory. The energy of a nonlinear polyatomic molecules can be approximated as

$E(T) = E_{ele} + E_{rot} + E_{vib} + E_{trans}$, $\quad E_{vib} = ZPE + E'_{vib}(T)$

where ZPE is the zero point energy and $E'_{vib}(T)$ is the temperature dependent portion vibrational energy. Proton does not possess rotational kinetic energy. The change from TQ to $TQ\bullet H^+$ does not bring about fundamental change in shape, thereby the change of rotational energy ΔE_{rot} here can be neglected. $E'_{vib}(T)$ is generally significantly smaller than ZPE and here only ΔZPE is considered. The change of translational energy E_{trans} of proton corresponds to 3RT/2.

Taken together, proton affinity (PA) at the standard state (298.15 K, 1.00 atm)

$PA = -\Delta H = -\Delta E + RT = -\Delta E_{ele} - ZPE + 5RT/2$

$= -[\{(-1202.631165) - (-1202.177383)\} + (0.360412 - 0.345692)] \times 627.50955 + 5 \times 1.987 \times 10^{-3} \times 298.15 / 2$

$= 276.9967$ kcal mol^{-1}

Cartesian geometry of the optimized structures is provided in Section 17.

13. Conformational Analysis of $TQ\bullet H^+$ and $DQ\text{-}Im\bullet H^+$

Conformational flexibility of $TQ\bullet H^+$ and $DQ\text{-}Im\bullet H^+$ was evaluated by molecular dynamics (MD) simulations. Initial structures were obtained by geometry optimization at the B3LYP-D3/6-31G(d,p) level of theory. All ab initio MD calculations were performed by atom-centered density matrix propagation (ADMP) method at 300 K. Calculated trajectories were saved each 0.5 femtosecond (fs) over 5000 fs, giving 10000 structures. The superimposed MD structures of TQ•H+ and DQ-Im•H+ (structures every 100 steps of MD simulation) are shown in Supplementary Fig. 57. Larger structural displacement was observed for $DQ\text{-}Im\bullet H^+$ compared with $TQ\bullet H^+$, both in entire molecular framework and the central proton.

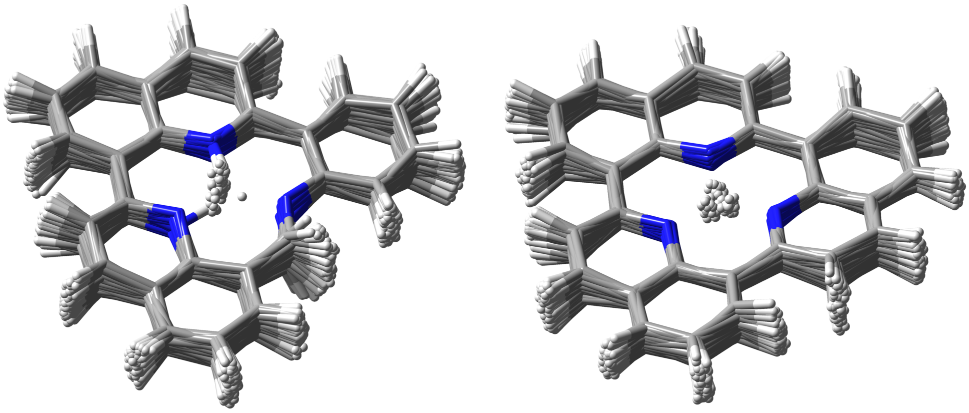

Supplementary Fig. 57 MD simulation of $TQ\bullet H^+$ and $DQ\text{-}Im\bullet H^+$.

14. DFT Calculations of Supramolecular Complexes of TQ

14-1. Inclusion complex TQ•H+ and [12]CPP.

In order to determine suitable calculation method, geometry optimization of [12]CPP⊃TQ•H+ **15** was performed using several DFT functionals with 6-31G(d,p) basis set followed by single point energy calculation with 6-311+G(d,p) basis set and SMD(DMSO) option. Each calculation gave similar structures albeit with large disparity in complexation enthalpy and Gibbs free energy (Supplementary Table 4). Generally, functionals with dispersion correction provides favorable complexation energy except for M06-2X functional. Given that empirically determined association constant K_1 = 1.203 x 10^3 mol^{-1} corresponds to ΔG = –4.23 kcal mol^{-1} (section 7-1), B3LYP-D3/6-31G(d) was taken as the most suitable optimization method. Further evaluation for the method of single point energy calculation identified that ωB97XD/def2-TZVPP/SMD(DMSO)//B3LYP-D3/6-31G(d) provided the ΔG = –7.94 kcal mol^{-1}, which was closest to the empirical value and this method was used for other supramolecular complexes in this study (Supplementary Fig. 58).

Geometry optimization and single point energy calculation of [12]CPP⊃(TQ•H+)2 **16** were performed at the identical level of theory (ωB97XD/def2-TZVPP/SMD(DMSO)//B3LYP-D3/6-31G(d)) (Supplementary Fig. 58). Large negative ΔG is deviated from the empirically determined association constant (K_2), likely due to overestimation of plane-to-plane interaction.

Supplementary Table 4 Energies obtained from different functionals. Energies are given in kcal mol^{-1}.

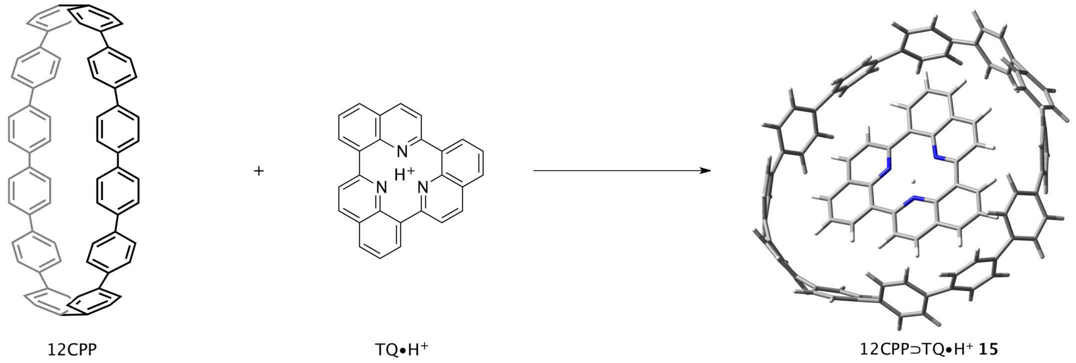

	B3LYP	B3LYP-D3	B3LYP-D3BJ	LC-BLYP	BMK	ωB97	ωB97XD	LC-ωPBE	M06-2X
ΔH	10.0	–26.9	–27.9	–4.8	3.3	–14.1	–28.0	3.4	–16.9
ΔG	24.9	–9.4	–10.9	11.9	22.0	3.1	–11.0	19.7	0.37

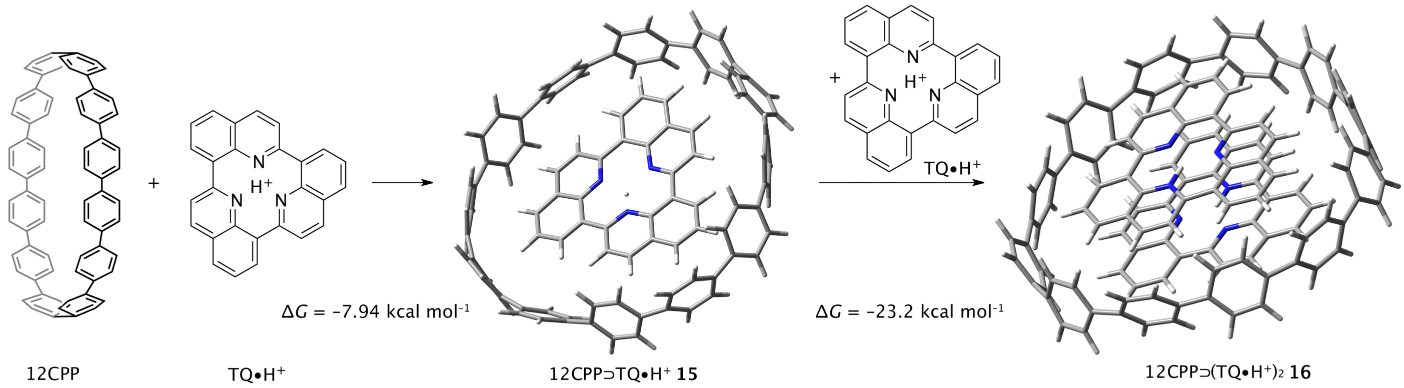

Supplementary Fig. 58 Optimized structure and complexation energy of [12]CPP⊃(TQ•H+)2 **16** calculated at the ωB97XD/def2-TZVPP/SMD(DMSO)//B3LYP-D3/6-31G(d) level of theory

Counterpoise corrected interaction energies of complexes **15** and **16** were calculated at the ωB97XD/def2-TZVPP level in the gas phase (Supplementary Fig. 59 and 60). In contrast to small deformation energy $\Delta E_{\mathrm{deform}}$ of TQ•H$^+$ for both complexes, that of [12]CPP is significantly higher in particular for complex 16 containing two molecules of TQ•H$^+$.

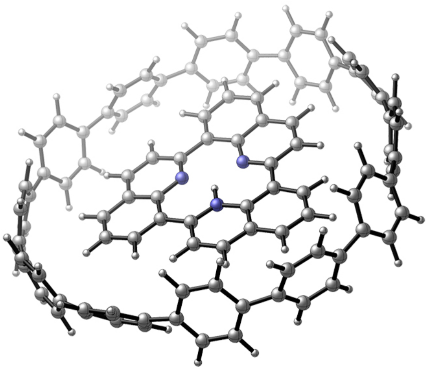

counterpoise corrected energy =	–3975.285059880880 hartree
BSSE energy =	0.001432096185 hartree
sum of fragments =	–3975.207964387086 hartree
complexation energy =	–49.28 kcal mol^{-1} (raw)
complexation energy =	–48.38 kcal mol^{-1} (corrected)

$\Delta E_{\mathrm{deform}}$ (TQ•H$^+$) = 0.56 kcal mol^{-1} ; $\Delta E_{\mathrm{deform}}$ ([12]CPP) = 1.27 kcal mol^{-1}

ΔE_{ZPE} = 2.21 kcal mol^{-1}

ΔE_{bind} = $\Delta E_{\mathrm{deform}}$ (TQ•H$^+$) + $\Delta E_{\mathrm{deform}}$ ([12]CPP) + corrected $\Delta E_{\mathrm{complexation}}$ + ΔE_{ZPE}
= –44.34 kcal mol^{-1}

Supplementary Fig. 59 Non-covalent association energy of TQ•H$^+$ and [12]CPP into inclusion complex [12]CPP⊃TQ•H$^+$ **15**.

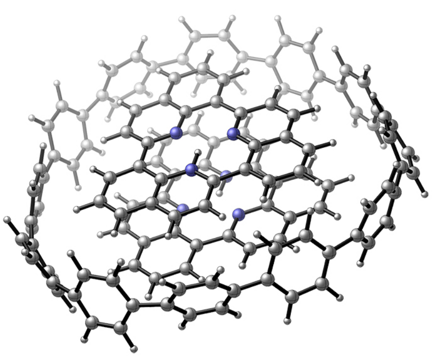

counterpoise corrected energy =	–5177.916930582436 hartree
BSSE energy =	0.003658951340 hartree
sum of fragments =	–5177.814390136555 hartree
complexation energy =	–66.64 kcal mol^{-1} (raw)
complexation energy =	–64.35 kcal mol^{-1} (corrected)

$\Delta E_{\mathrm{deform}}$ (TQ[1]•H$^+$) = 0.75 kcal mol^{-1} ; $\Delta E_{\mathrm{deform}}$ (TQ[2]•H$^+$) = 0.68 kcal mol^{-1};
$\Delta E_{\mathrm{deform}}$ ([12]CPP) = 6.14 kcal mol^{-1}; ΔE_{ZPE} = 4.05 kcal mol^{-1}

ΔE_{bind} = $\Delta E_{\mathrm{deform}}$ (TQ[1]•H$^+$) + $\Delta E_{\mathrm{deform}}$ (TQ[2]•H$^+$) + $\Delta E_{\mathrm{deform}}$ ([12]CPP)
+ corrected $\Delta E_{\mathrm{complexation}}$ + ΔE_{ZPE} = –52.73 kcal mol^{-1}

Supplementary Fig. 60 Non-covalent association energy of two molecules of TQ•H$^+$ and [12]CPP into inclusion complex [12]CPP⊃(TQ•H$^+$)$_2$ **16**.

14-2. Complex of TQ•H$^+$ and coronene.

Geometry optimization and single point energy calculation of plane-to-plane TQ•H$^+$/coronene complex **14** were performed at the ωB97XD/def2-TZVPP/SMD(DMSO)//B3LYP-D3/6-31G(d) level of theory as described above (Supplementary Fig. 61). Larger negative ΔG (–10.9 kcal mol^{-1}) compared to [12]CPP⊃TQ•H$^+$ **15** (–7.9 kcal mol^{-1}) reproduced the empirical observation (K_1: coronene complex **14** (1.62 ± 0.009) × 10^3 M^{-1}; [12]CPP complex **15**: K_1 = (1.20 ± 0.035) × 10^3 M^{-1}. Counterpoise corrected interaction energy of complexes **14** was calculated at the ωB97XD/def2-TZVPP level in the gas phase (Supplementary Fig. 62)

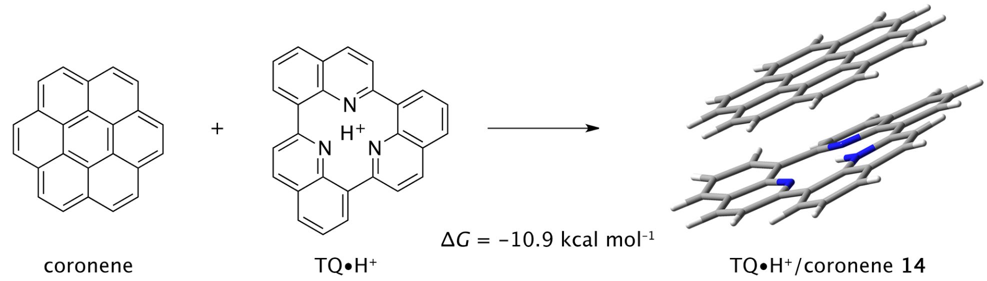

Supplementary Fig. 61 Optimized structure and complexation energy of TQ•H$^+$/coronene complex **14** calculated at the ωB97XD/def2-TZVPP/SMD(DMSO)//B3LYP-D3/6-31G(d) level of theory.

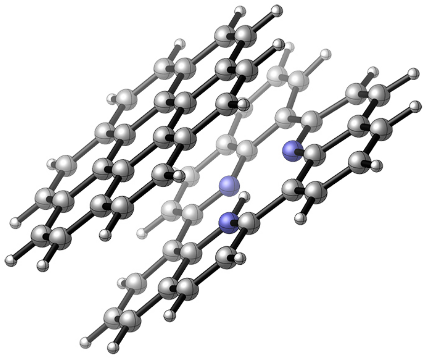

counterpoise corrected energy =	–2124.544343726800 hartree
BSSE energy =	0.001224503719 hartree
sum of fragments =	–2124.493843537497 hartree
complexation energy =	–32.46 kcal mol^{-1} (raw)
complexation energy =	–31.69 kcal mol^{-1} (corrected)

ΔE_{deform} (TQ•H$^+$) = ~0.0 kcal mol^{-1}; ΔE_{deform} (coronene) = ~0.0 kcal mol^{-1}

ΔE_{ZPE} = 0.80 kcal mol^{-1}

ΔE_{bind} = ΔE_{deform} (TQ•H$^+$) + ΔE_{deform} (coronene) + corrected $\Delta E_{\text{complexation}}$ + ΔE_{ZPE}
= –30.89 kcal mol^{-1}

Supplementary Fig. 62 Non-covalent association energy of TQ•H$^+$ and coronene into TQ•H$^+$/coronene complex **14**.

14-3. Complex of TQ•H$^+$, [12]CPP, and coronene.

Geometry optimization and single point energy calculation of inclusion complex of [12]CPP⊃(TQ•H$^+$/coronene) **17** were performed at the ωB97XD/def2-TZVPP/SMD(DMSO)//B3LYP-D3/6-31G(d) level of theory as described above (Supplementary Fig. 63). Counterpoise corrected interaction energy of complexes **17** was calculated at the ωB97XD/def2-TZVPP level in the gas phase (Supplementary Fig. 64).

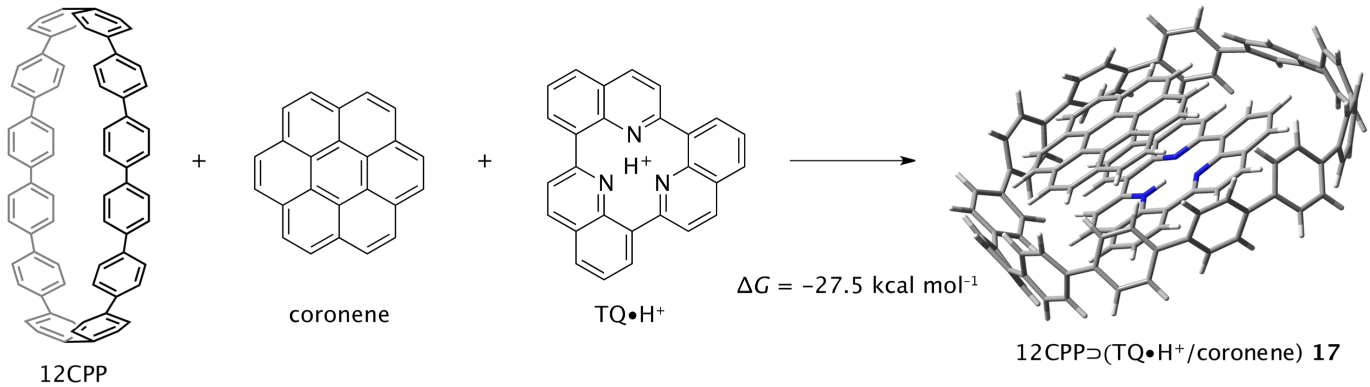

Supplementary Fig. 63 Optimized structure and complexation energy of [12]CPP⊃(TQ•H$^+$/coronene) **17** calculated at the ωB97XD/def2-TZVPP/SMD(DMSO)//B3LYP-D3/6-31G(d) level of theory.

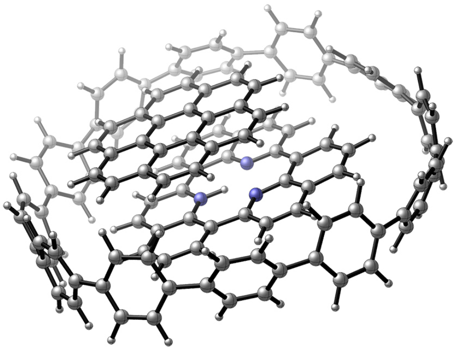

counterpoise corrected energy =	–4897.224902012853 hartree
BSSE energy =	0.003172701103 hartree
sum of fragments =	–4897.082924515382 hartree
complexation energy =	–91.08 kcal mol^{-1} (raw)
complexation energy =	–89.09 kcal mol^{-1} (corrected)

ΔE_{deform} (TQ•H$^+$) = 0.59 kcal mol^{-1}; ΔE_{deform} ([12]CPP) = 3.09 kcal mol^{-1};

ΔE_{deform} (coronene) = 0.14 kcal mol^{-1}; ΔE_{ZPE} = 3.60 kcal mol^{-1}

ΔE_{bind} = ΔE_{deform} (TQ•H$^+$) + ΔE_{deform} ([12]CPP) + ΔE_{deform} (coronene)
+ corrected $\Delta E_{\text{complexation}}$ + ΔE_{ZPE} = –81.66 kcal mol^{-1}

Supplementary Fig. 64 Non-covalent association energy of TQ•H$^+$, [12]CPP, and coronene into 12CPP⊃(TQ•H$^+$/coronene) **17**.

14-4. DFT-GIAO calculations of supramolecular complexes.

DFT-GIAO calculation was performed at the B3LYP/6-311+G(2d,p)/SMD(DMSO) level of theory on the optimized structures (B3LYP-D3/6-31G(d,p)) of complexes TQ•H^+/coronene complex **14**, [12]CPP⊃TQ•H^+ **15**, [12]CPP⊃$(TQ•H^+)_2$ **16**, and [12]CPP⊃(TQ•H^+/coronene) **17** to estimate the ^{1}H resonance of each complex (Supplementary Fig. 65). For both coronene complex **14** and [12]CPP complex **15**, empirically observed upfield shift of coronene resonances and downfield shift of [12]CPP resonances were reproduced. Enhanced upfield shift of coronene in a ternary mixture of TQ•H+/[12]CPP/coronene was also reproduced in the calculation of complex **17**.

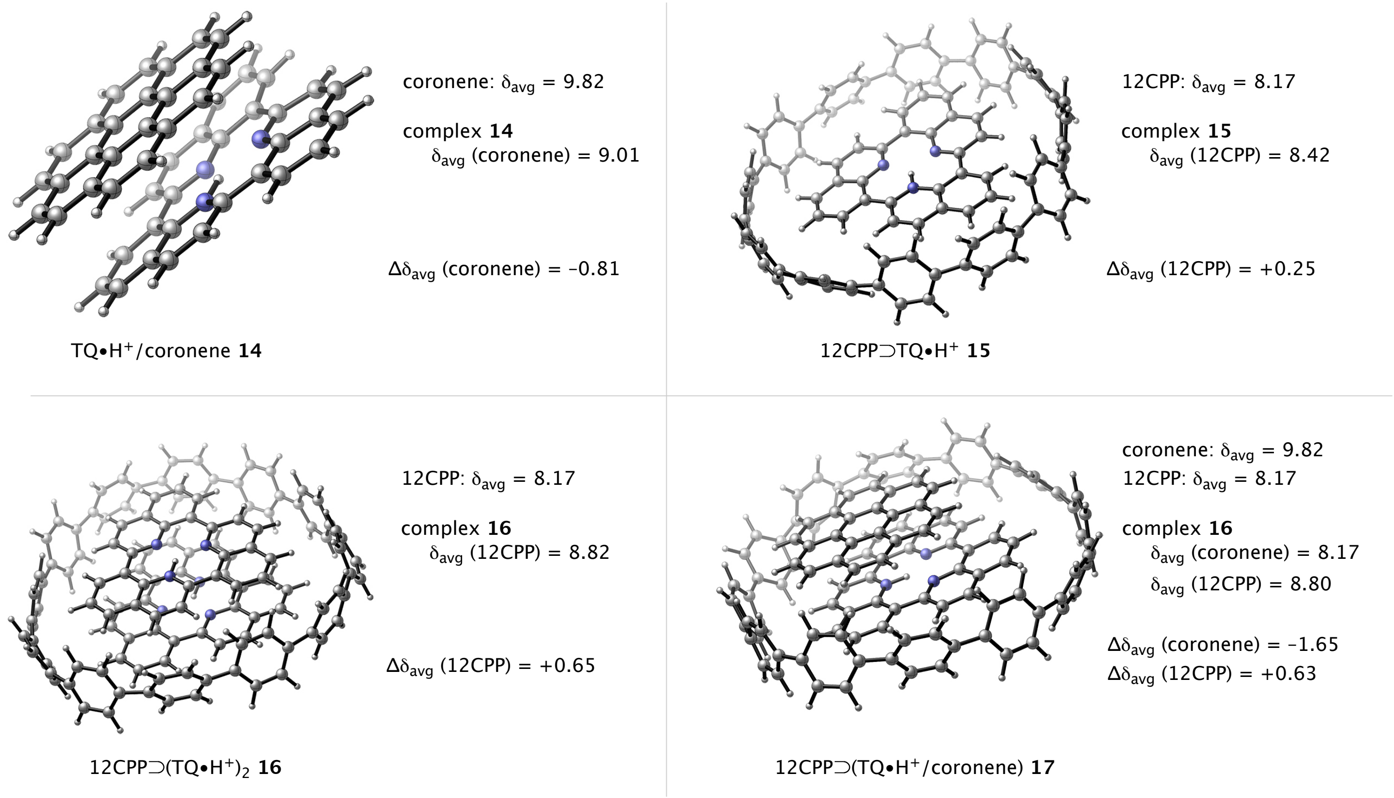

Supplementary Fig. 65 Averaged chemical shift of coronene and [12]CPP as a monomeric form and in supramolecular complexes calculated at the B3LYP/6-311+G(2d,p)/SMD(DMSO) level of theory.

14-5. SAPT calculation.

Interaction energy of inclusion complex [12]CPP⊃TQ•H^+ **15** was analyzed in terms of physically meaningful components, e.g. electrostatic (E_{elst}), exchange (E_{ex}), induction (E_{ind}), and dispersion (E_{disp}) energies by conducting the symmetry adopted perturbation theory (SAPT) calculation. SAPT0 approach was employed with jun-cc-pVTZ basis set.[11,12] The optimized geometry obtained above was used (B3LYP-D3/6-31G(d,p)). The dispersion (E_{disp}) is revealed to make the largest contribution for the complexation, which is consistent with the apparent significance of the CH–π interaction (Supplementary Fig. 66).

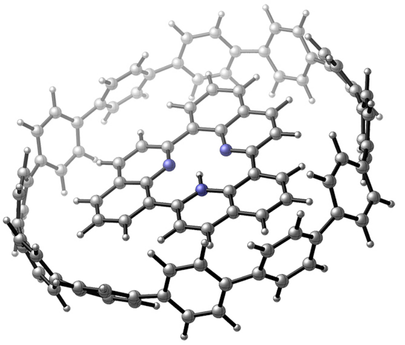

Inclusion complex [12]CPP⊃TQ•H^+ **15**

E_{elst}	–28.62 kcal mol^{-1}
E_{ex}	42.20 kcal mol^{-1}
E_{ind}	–12.69 kcal mol^{-1}
E_{disp}	–52.43 kcal mol^{-1}
SAPT0	–51.53 kcal mol^{-1}

Supplementary Fig. 66 Energy decomposition of inclusion complex [12]CPP⊃TQ•H^+ **15** by SAPT0/jun-cc-pVTZ approach.

<u>*14-6 NCIPLOT of supramolecular complexes.*</u>

Non-covalent interaction of supramolecular complexes TQ•H$^+$/coronene complex **14**, [12]CPP⊃TQ•H$^+$ **15**, [12]CPP⊃(TQ•H$^+$)$_2$ **16**, and [12]CPP⊃(TQ•H$^+$/coronene) **17** were visualized by NCIPLOT program (Supplementary Fig. 67).[13] Optimized geometry was obtained DFT calculations described above.

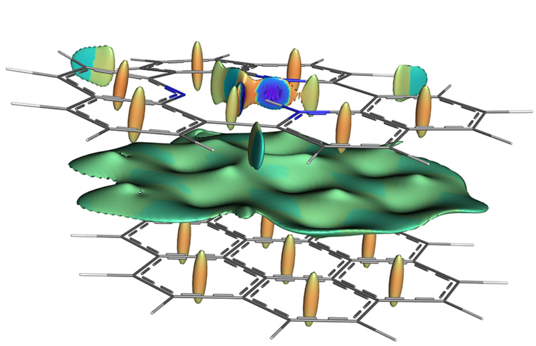

TQ•H$^+$/coronene **14**

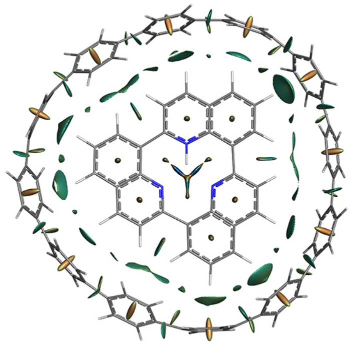

12CPP⊃TQ•H$^+$ **15**

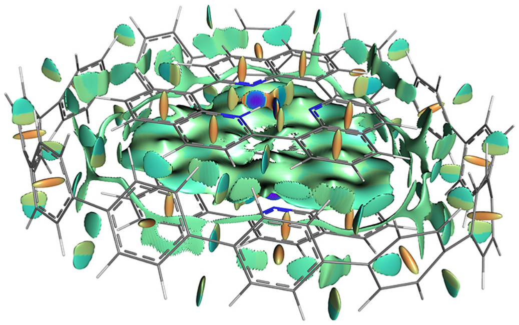

12CPP⊃(TQ•H$^+$)$_2$ **16**

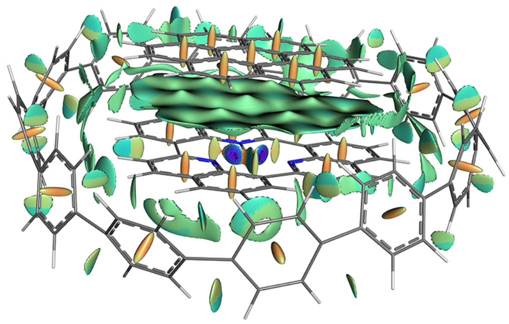

12CPP⊃(TQ•H$^+$/coronene) **17**

Supplementary Fig. 67 NCIPLOT of supramolecular plane-to-plane andedge-to-plane complexes containing TQ•H$^+$.

15. Topoisomerase I Assay

Supercoiled pBluescriptII SK(+) (500 ng) was incubated with 2 units of topoisomerase I at 37 °C for 1 h in reaction buffer [50 mM Tris-HCl (pH 7.9), 50 mM KCl, 0.1 mM EDTA, 10 mM MgCl$_2$, and 0.5 mM DTT] in the presence of varying concentrations of DQ-Im•TFA **9**, TQ•TFA **10**, or doxorubicin. Reactions were terminated by adding SDS and proteinase K (final 0.2% and 0.5 mg/ml, respectively), and the samples were incubated at 37 °C for 15 min. DNA samples were electrophoresed in a 1% agarose gel and stained by ethidium bromide after electrophoresis.

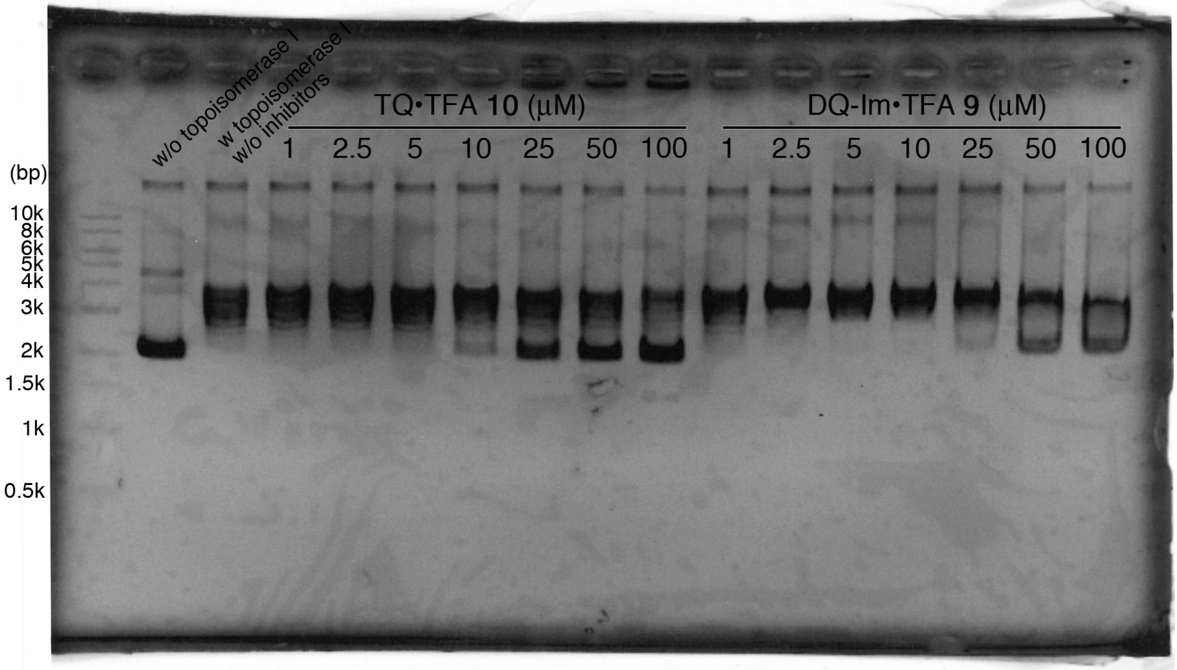

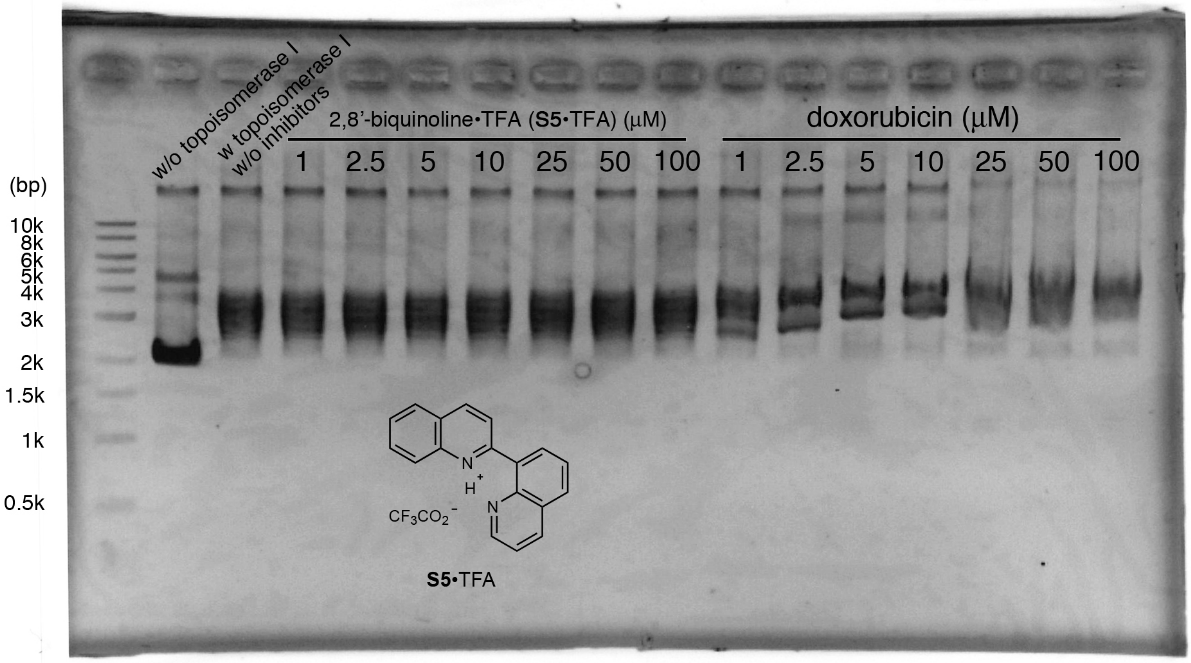

Supplementary Fig. 68 Unprocessed scan of the gel image.

16. ^{1}H, ^{13}C, and ^{19}F NMR Spectra of New Compounds

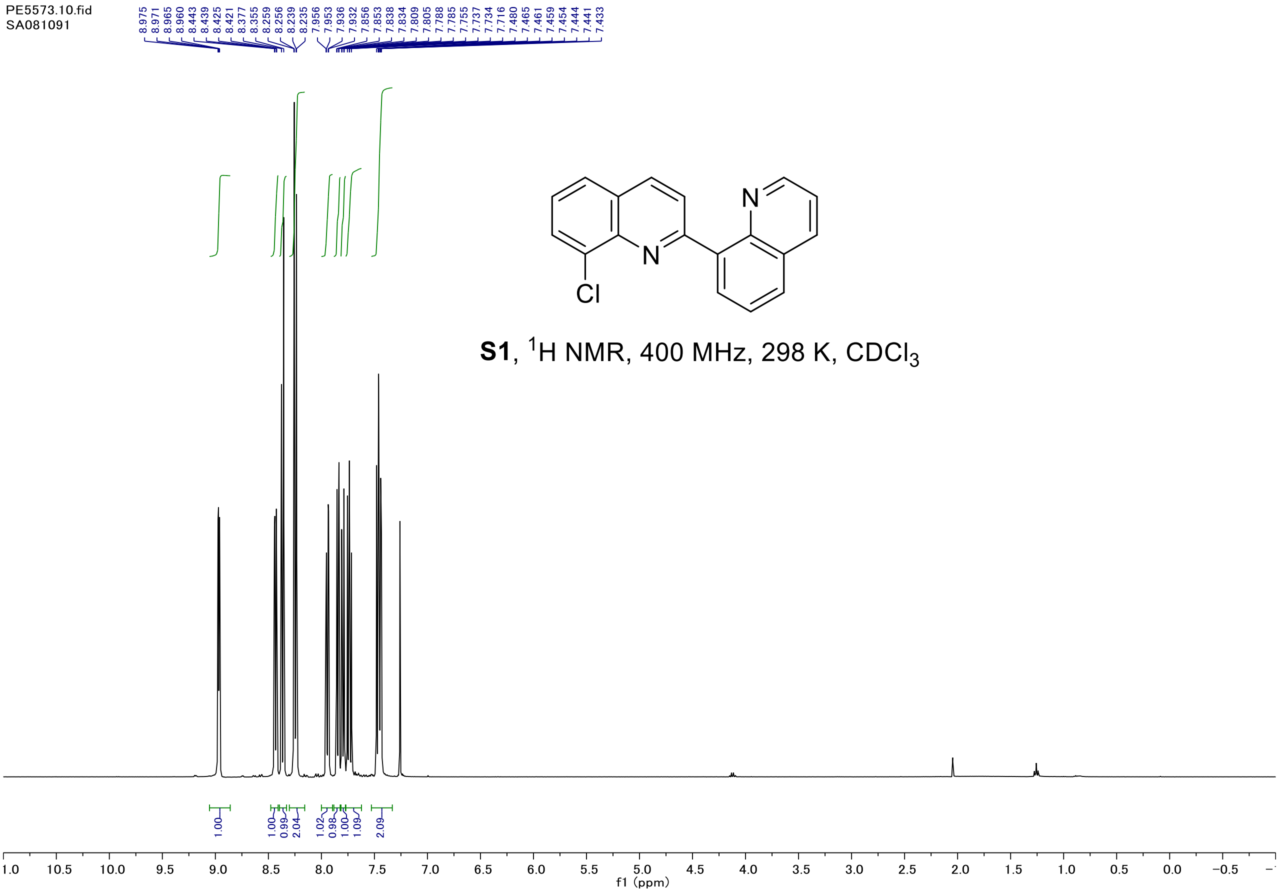

Supplementary Fig. 69 ^{1}H NMR spectrum of **S1**.

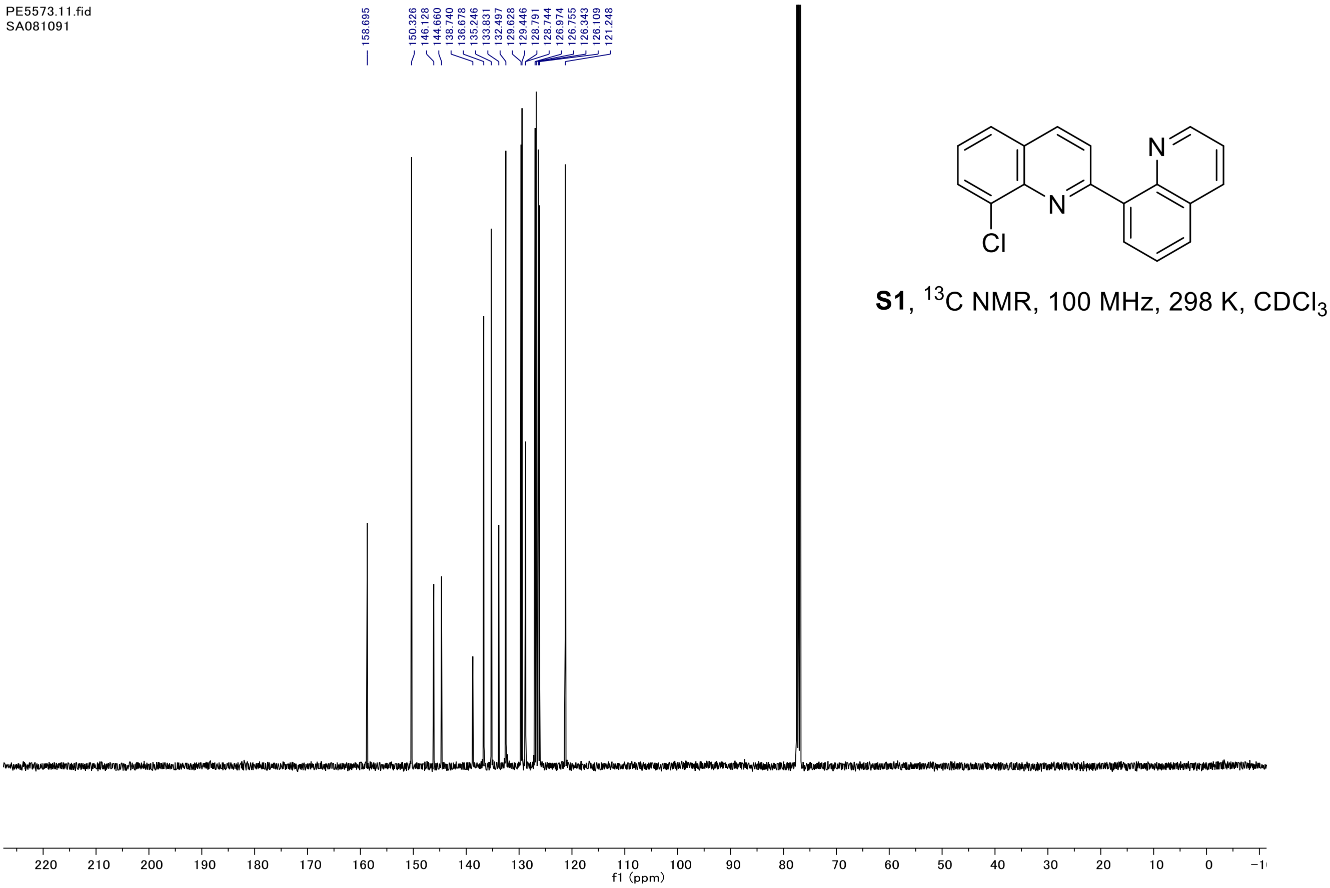

Supplementary Fig. 70 ^{13}C NMR spectrum of **S1**.

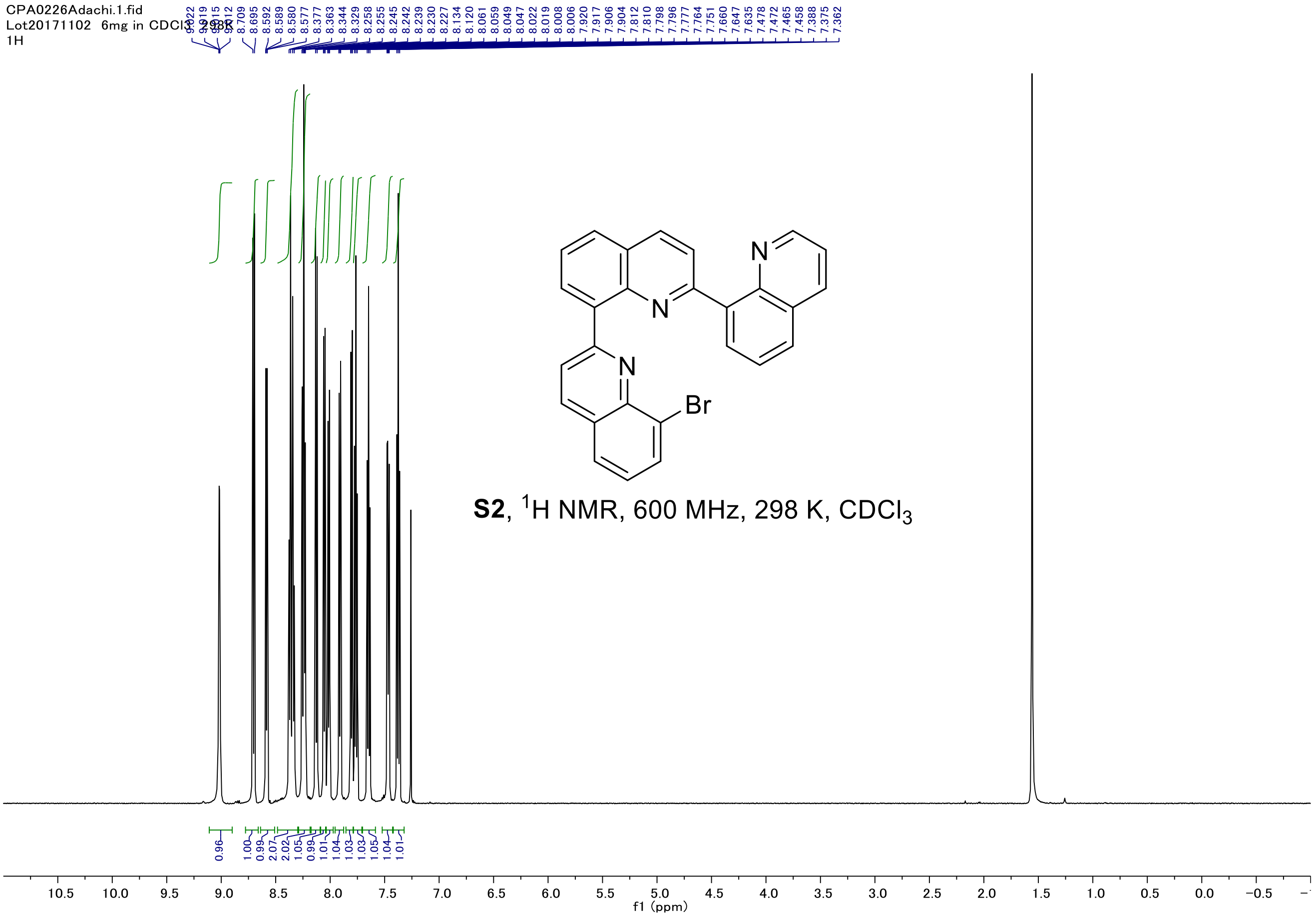

Supplementary Fig. 71 ^{1}H NMR spectrum of **S2**.

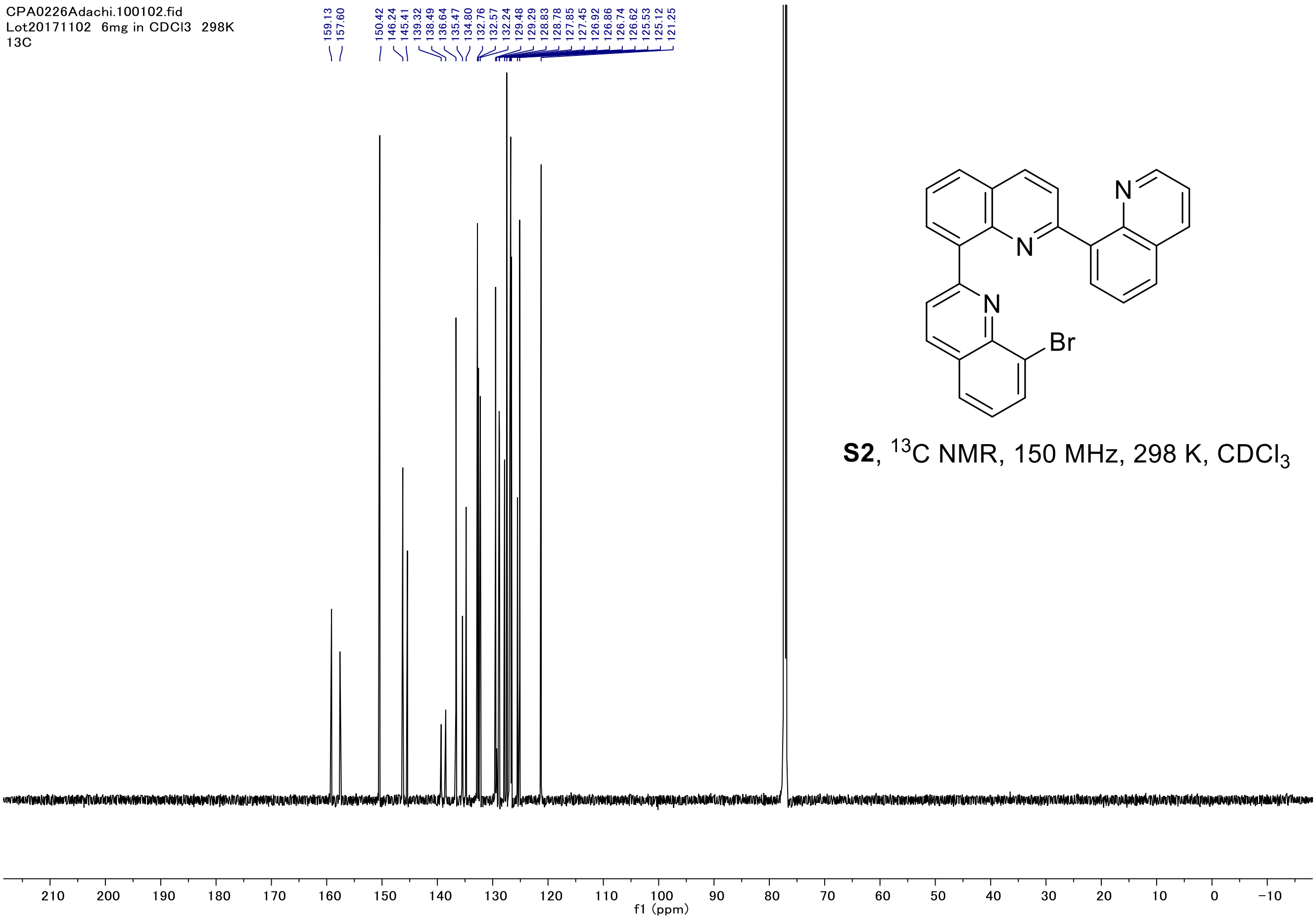

Supplementary Fig. 72 ^{13}C NMR spectrum of **S2**.

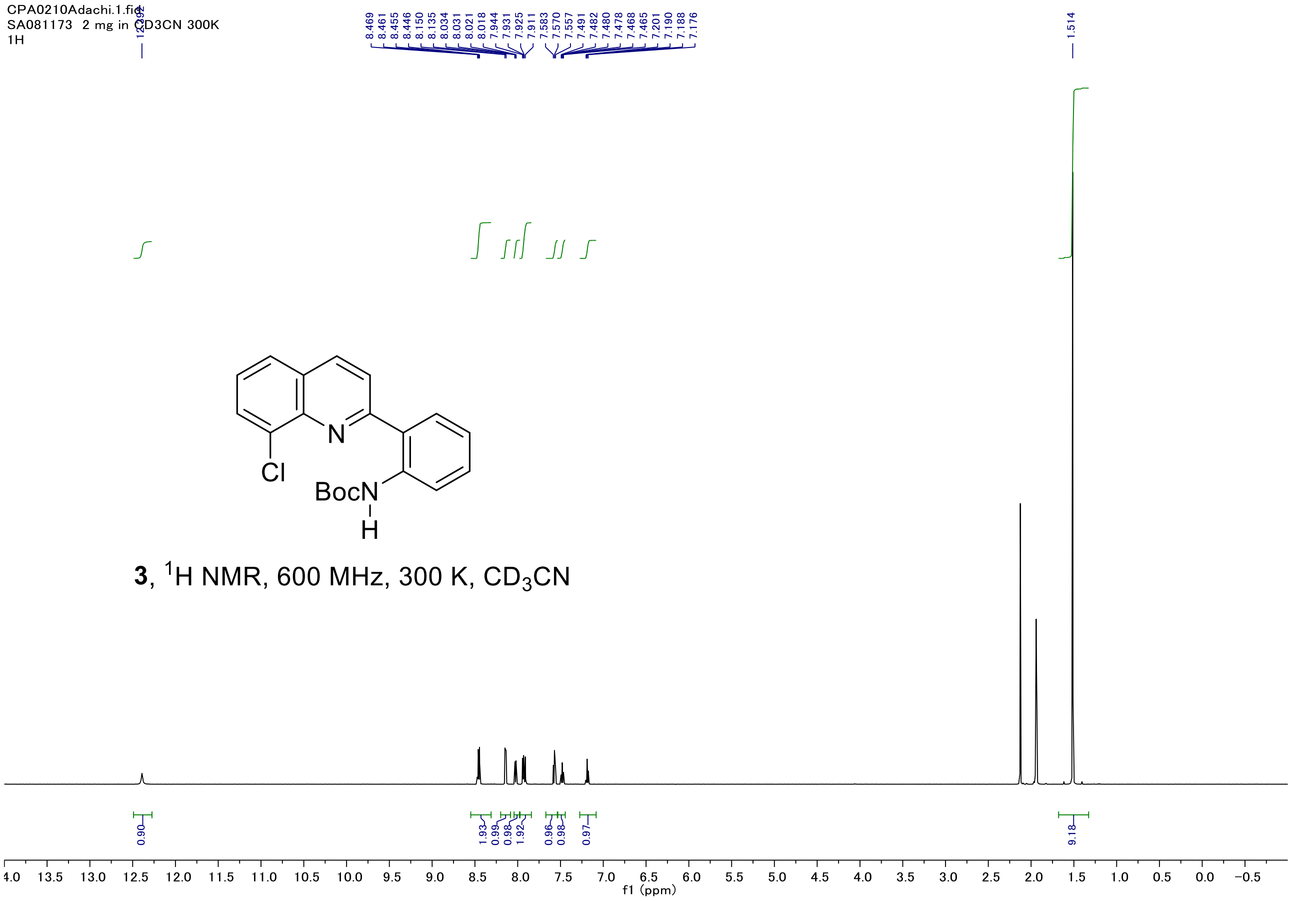

Supplementary Fig. 73 ^{1}H NMR spectrum of **S3**.

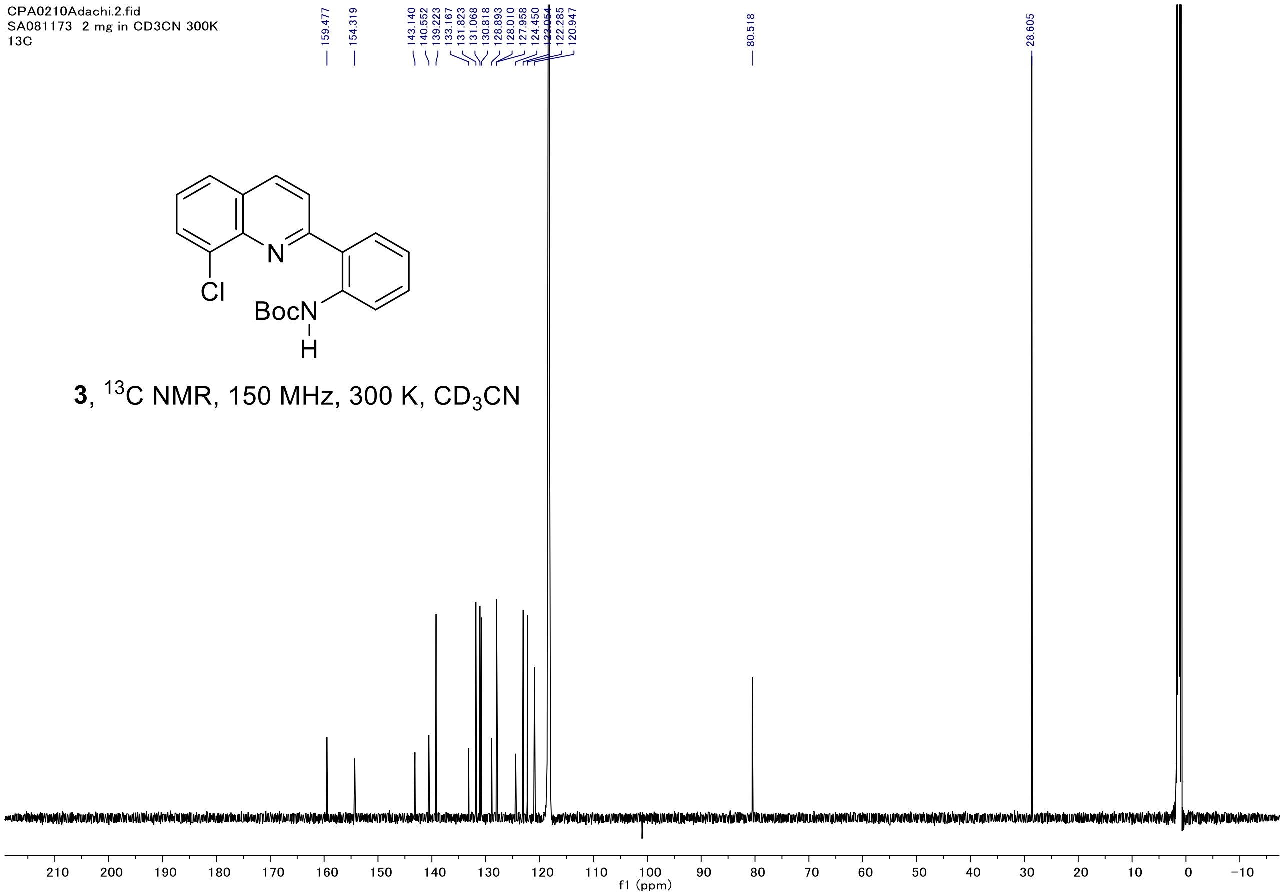

Supplementary Fig. 74 ^{13}C NMR spectrum of **S3**.

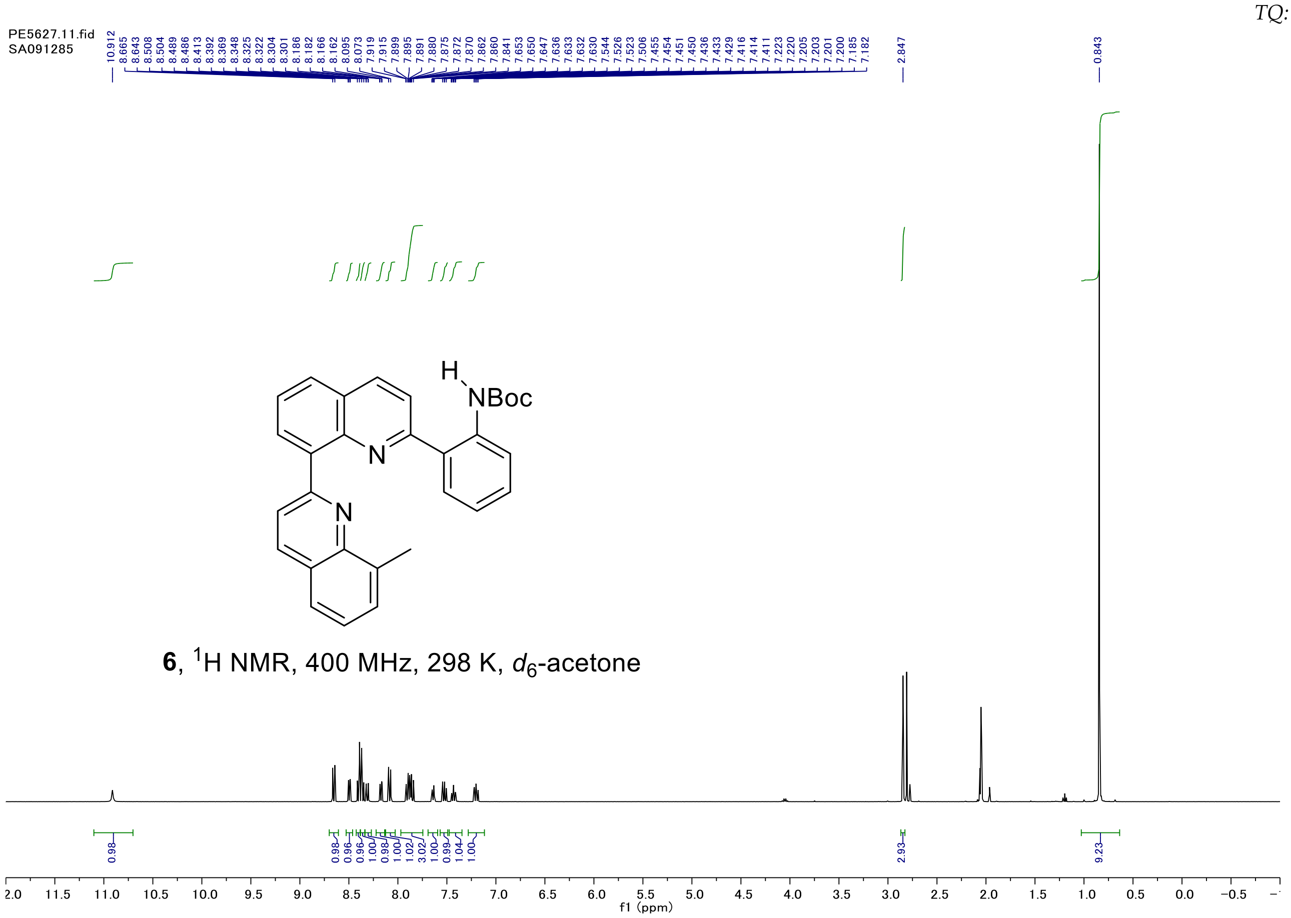

Supplementary Fig. 75 ^{1}H NMR spectrum of **6**.

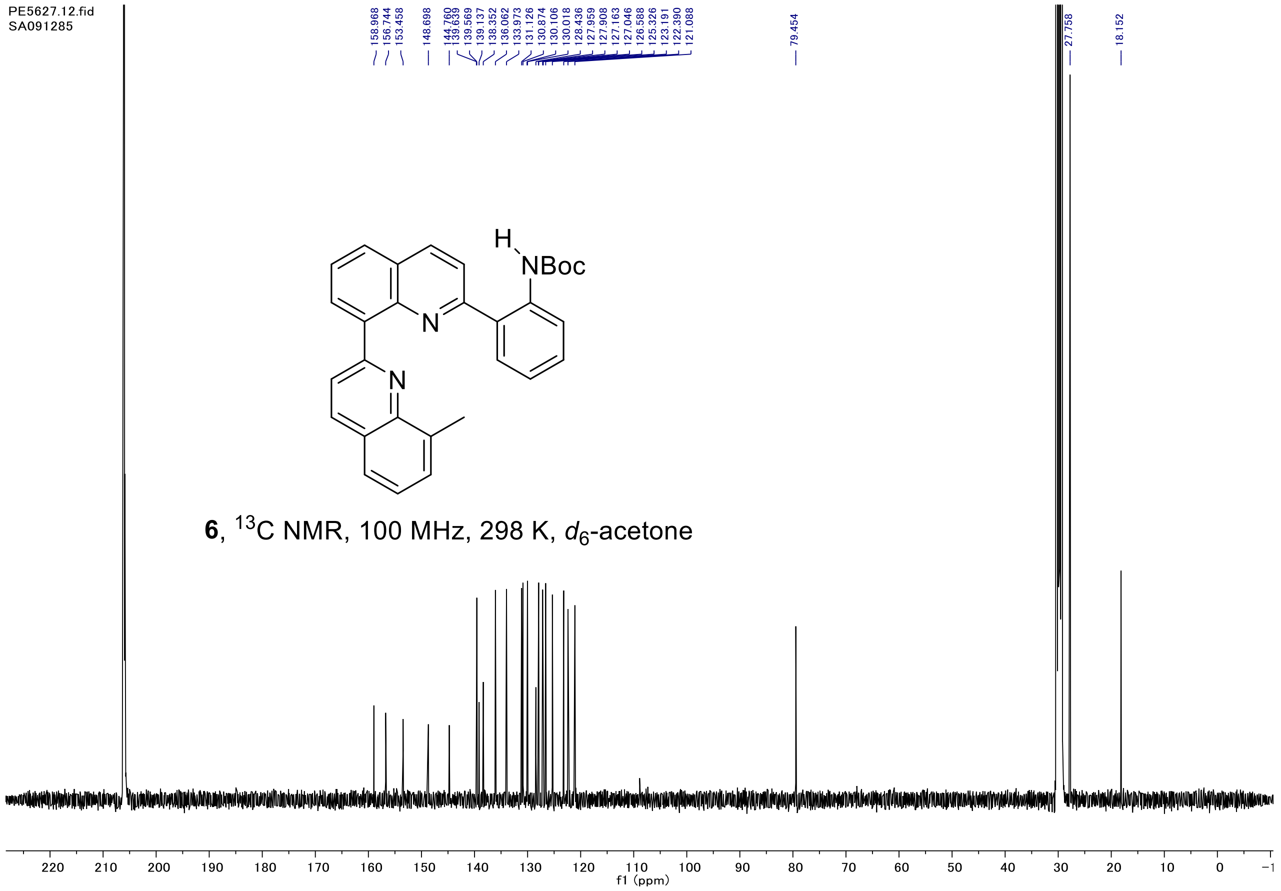

Supplementary Fig. 76 ^{13}C NMR spectrum of **6**.

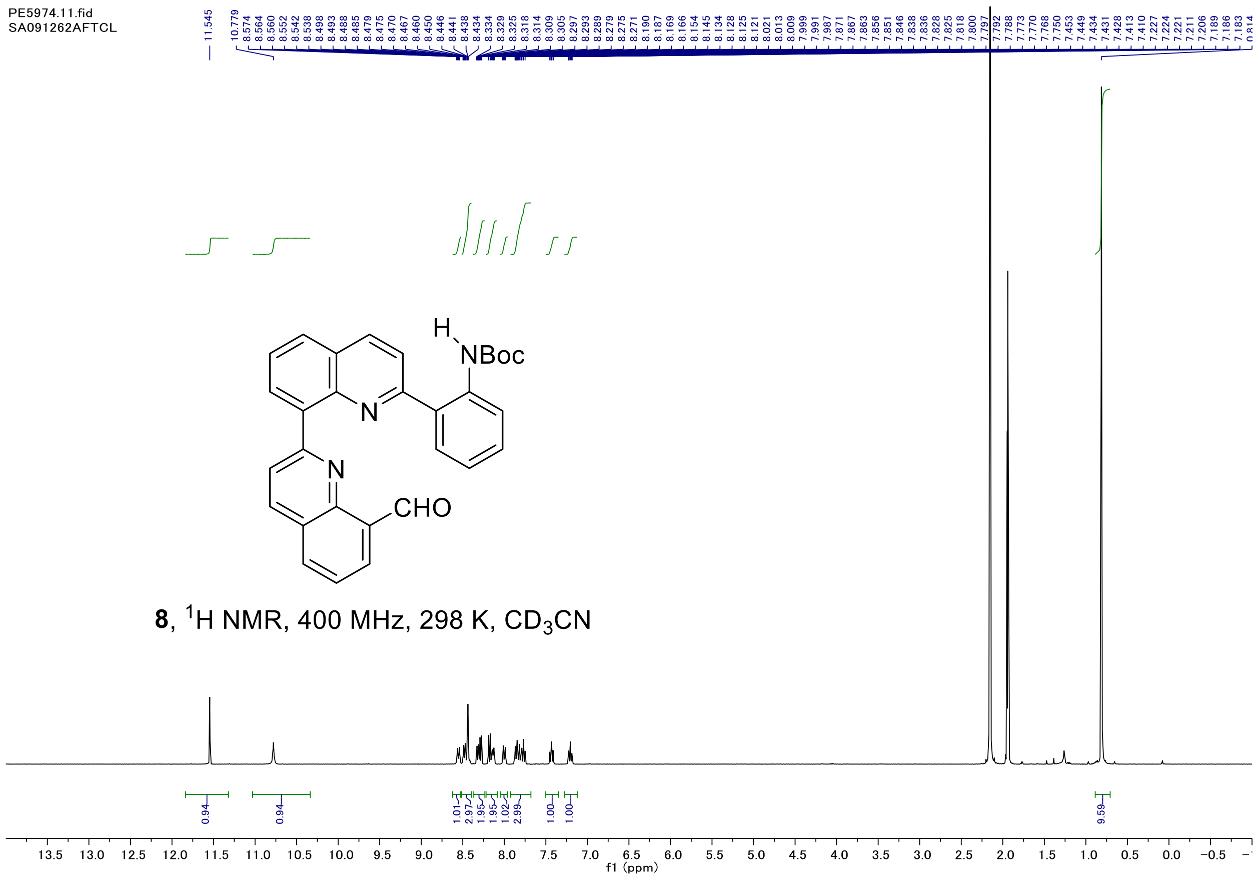

Supplementary Fig. 77 ^{1}H NMR spectrum of **8**.

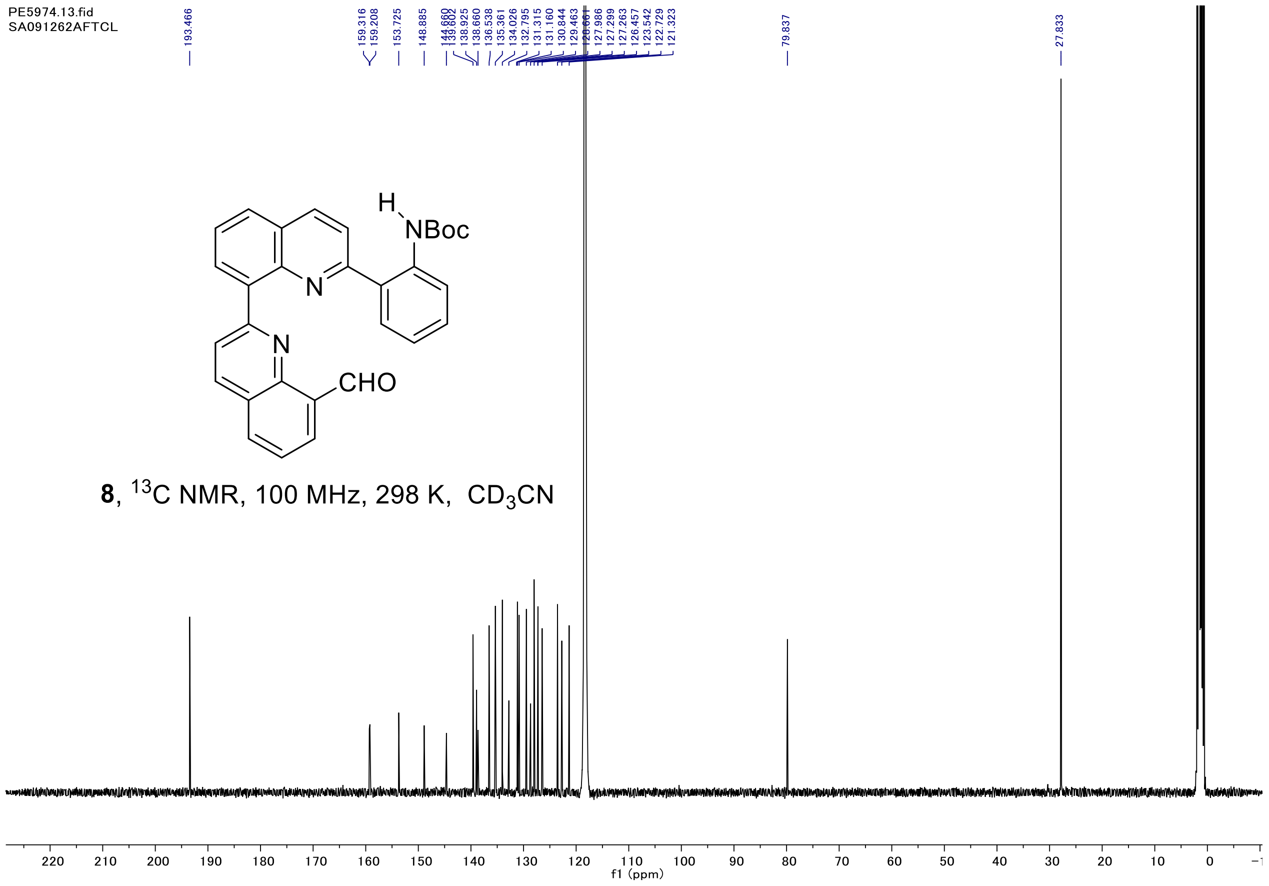

Supplementary Fig. 78 ^{13}C NMR spectrum of **8**.

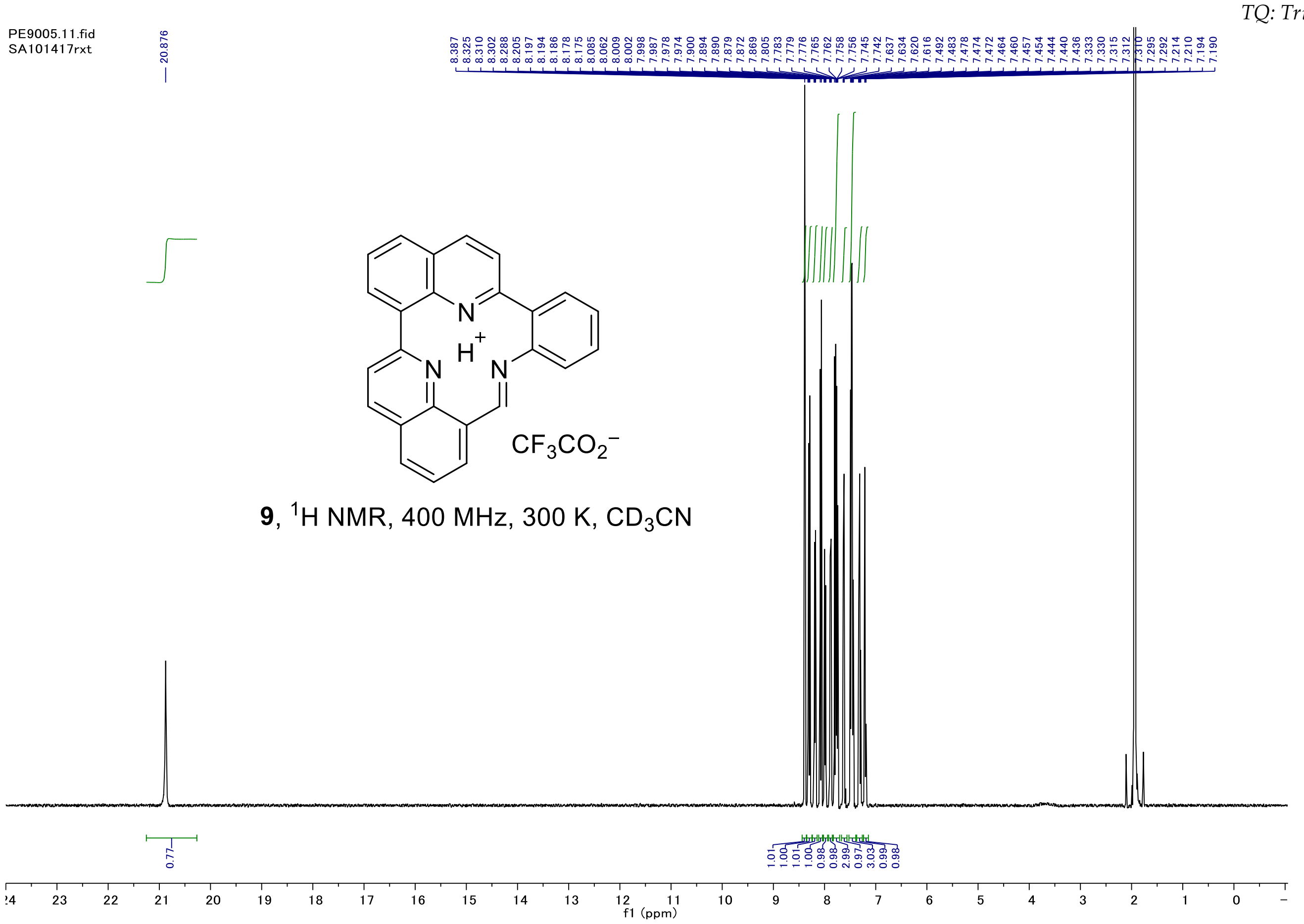

Supplementary Fig. 79 ¹H NMR spectrum of **9**.

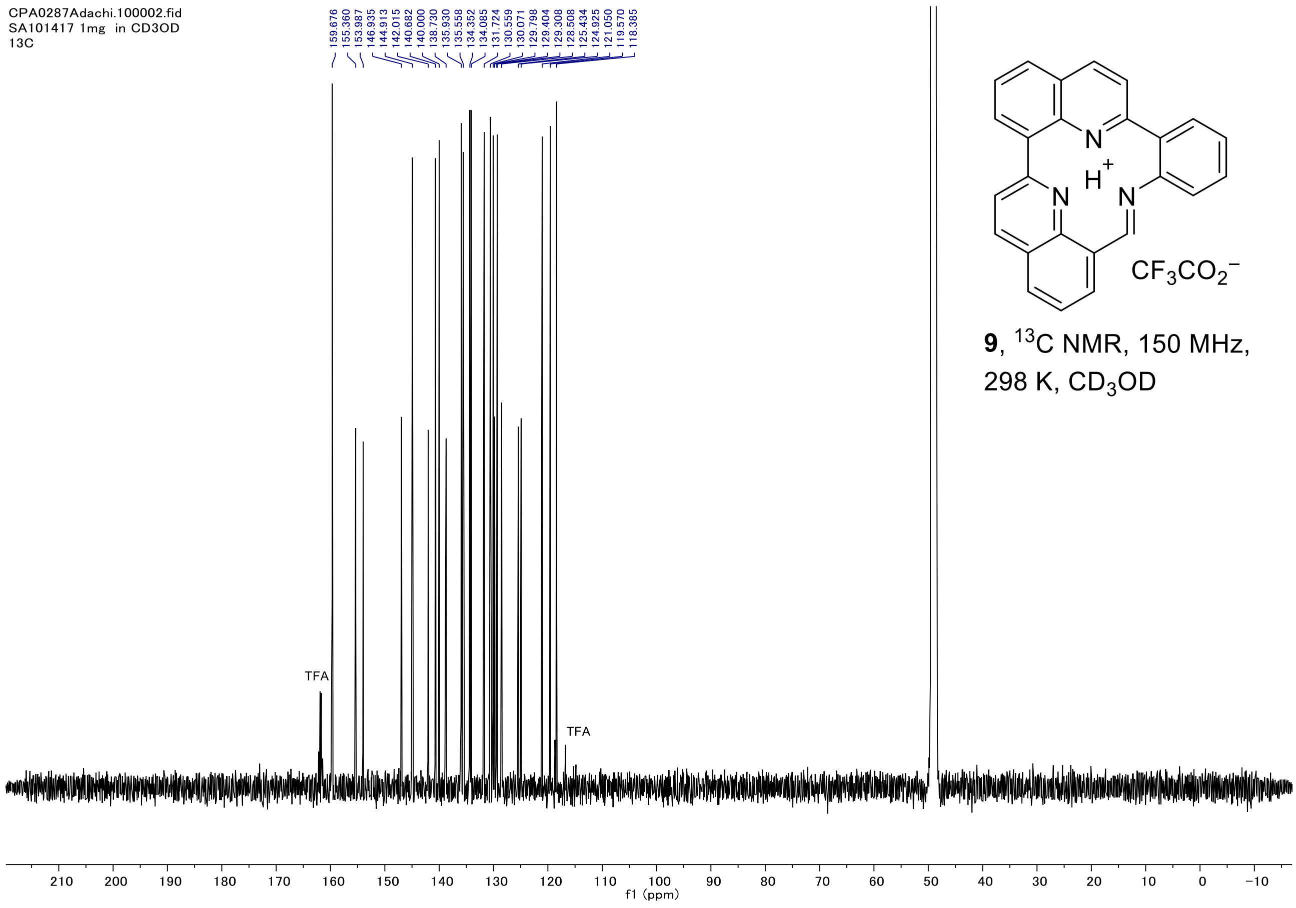

Supplementary Fig. 80 ¹³C NMR spectrum of **9**.

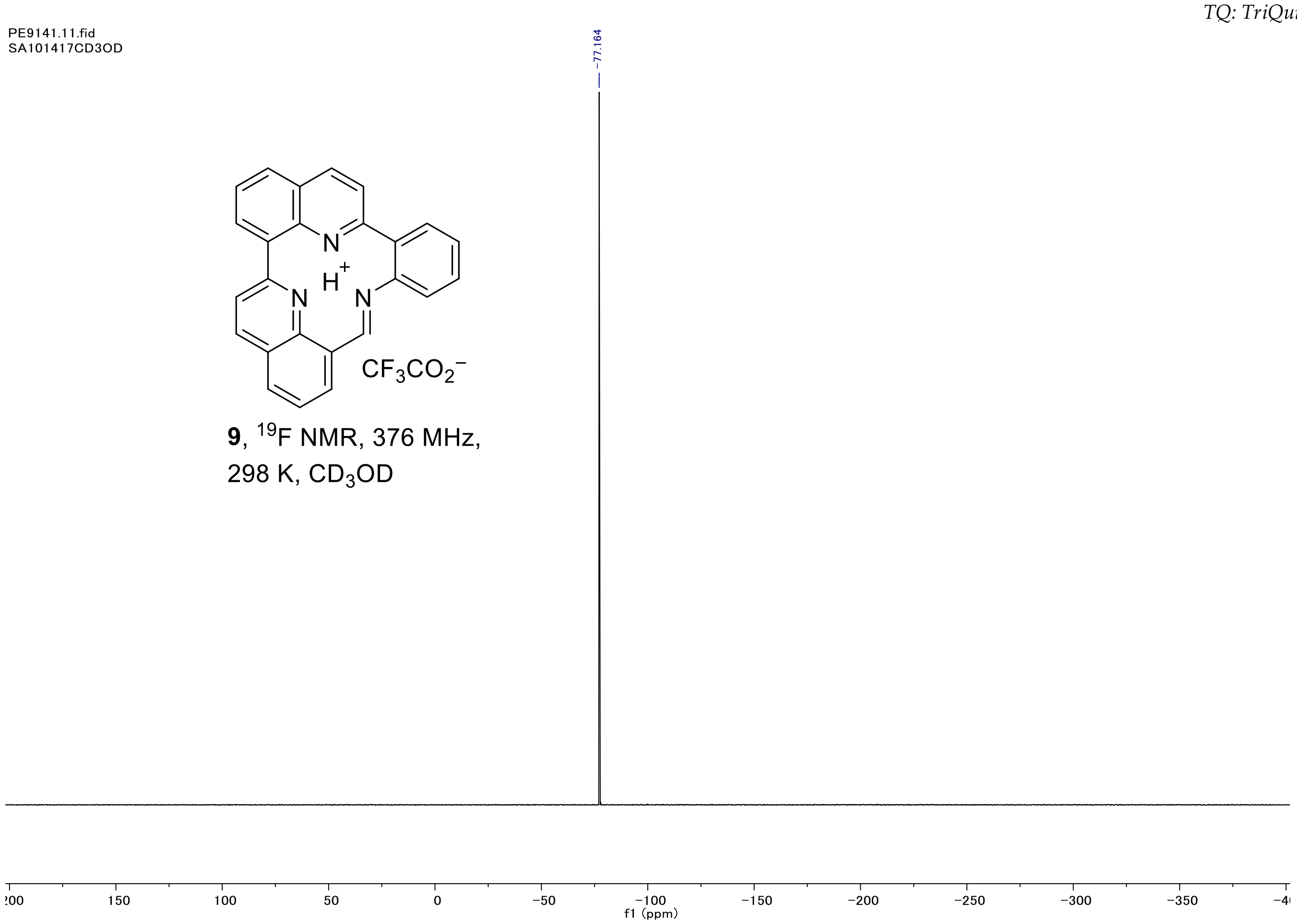

Supplementary Fig. 81 ^{19}F NMR spectrum of **9**.

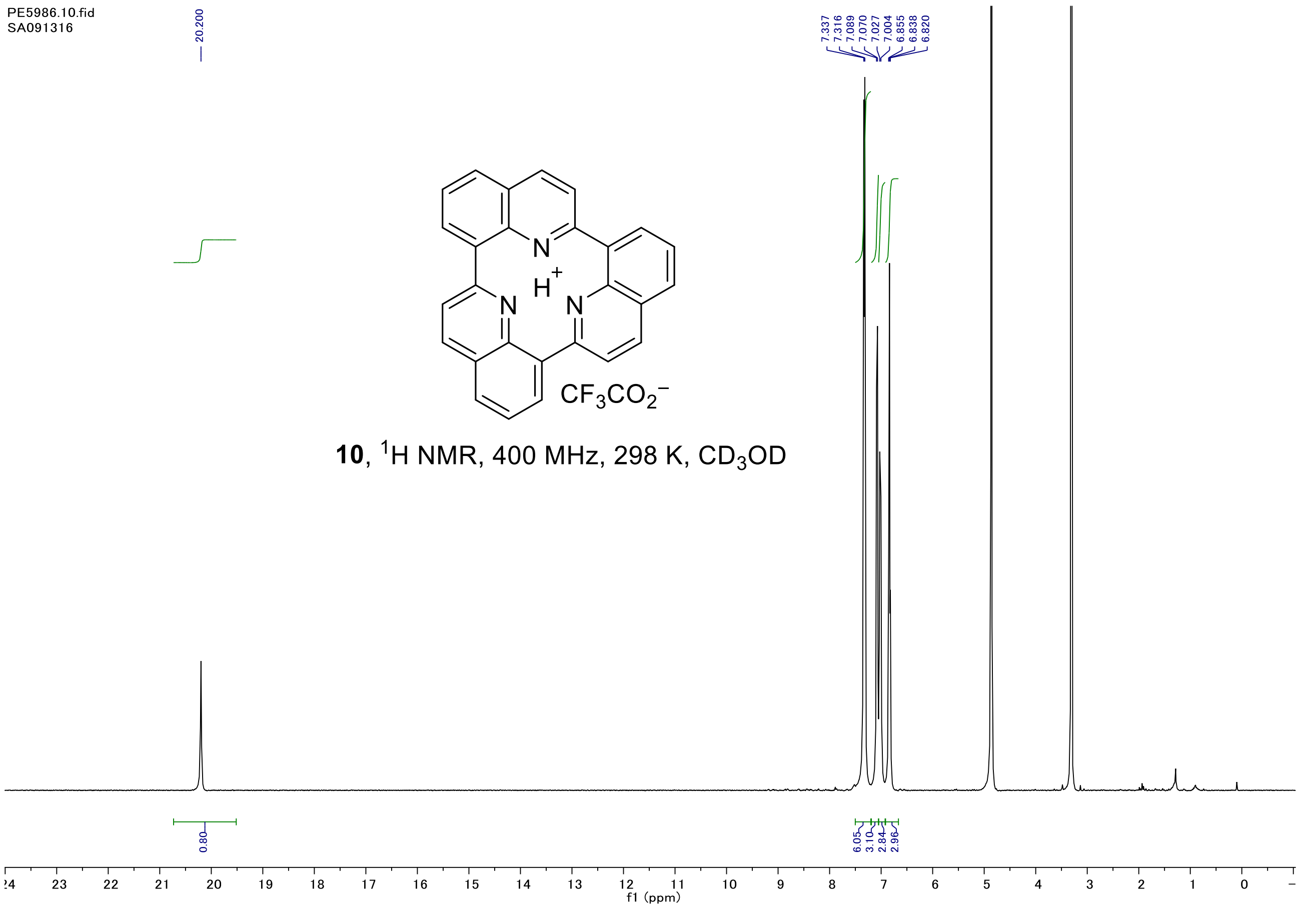

Supplementary Fig. 82 ^{1}H NMR spectrum of **10**.

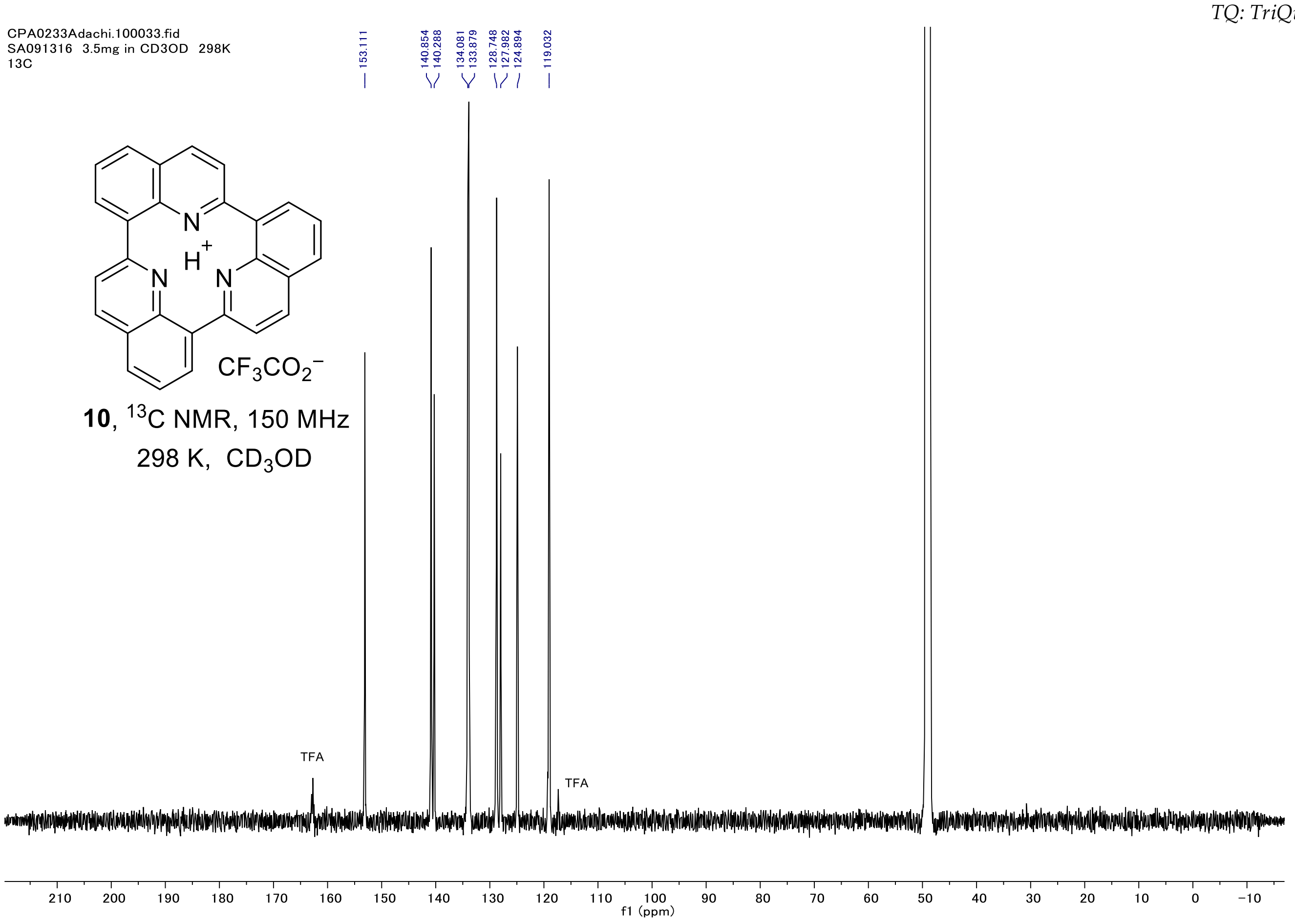

Supplementary Fig. 83 ^{13}C NMR spectrum of **10**.

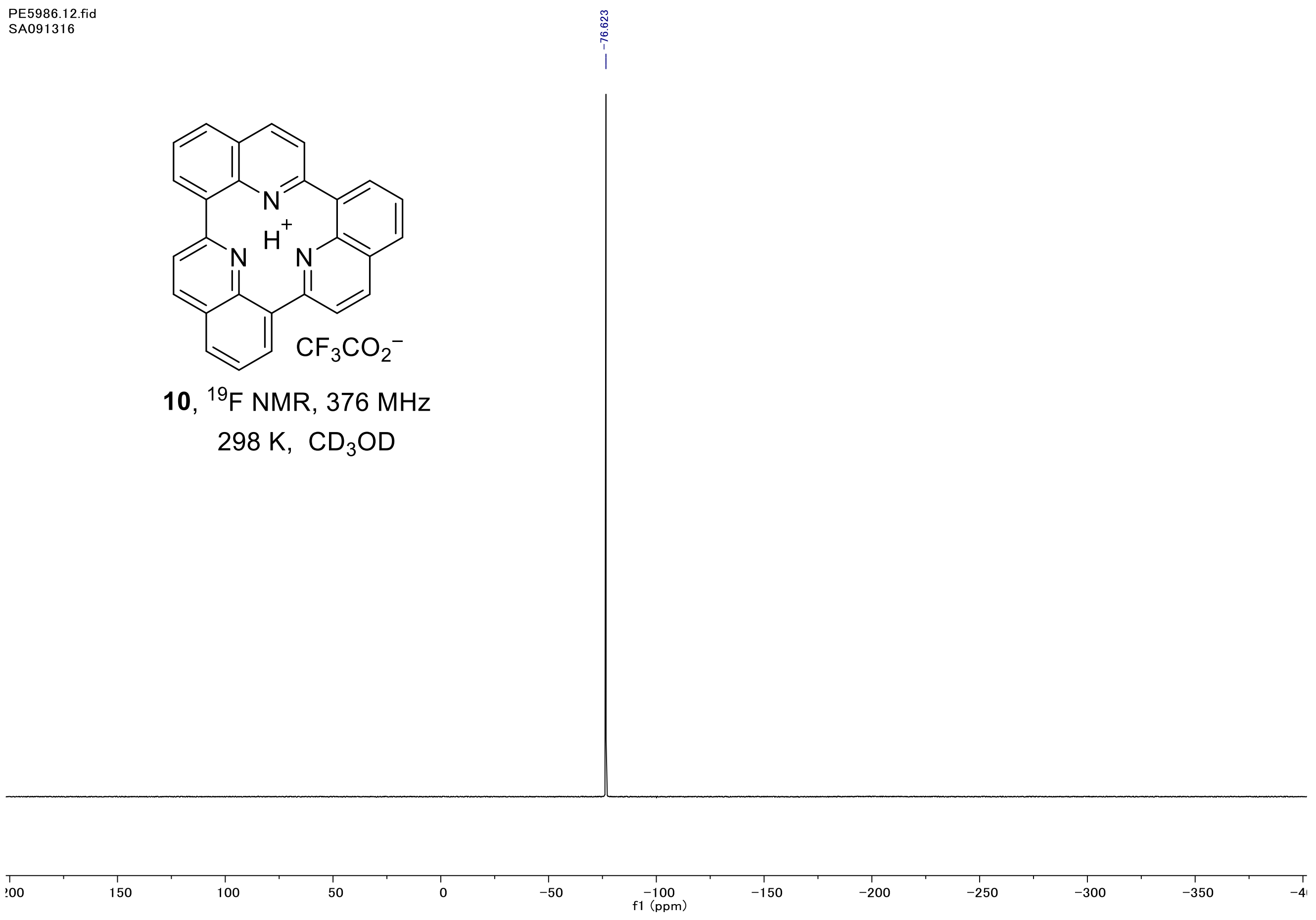

Supplementary Fig. 84 ^{19}F NMR spectrum of **10**.

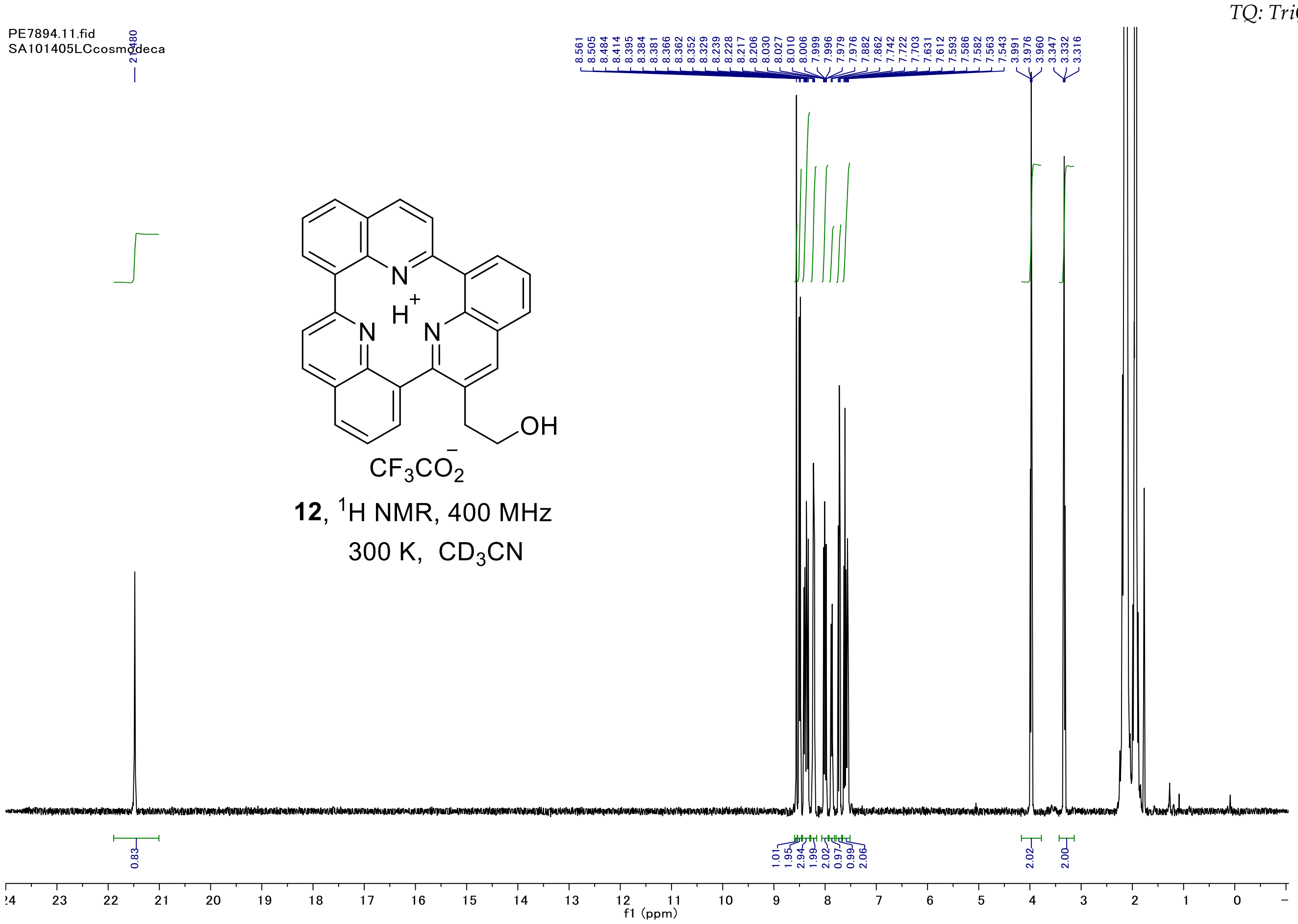

Supplementary Fig. 85 ^{1}H NMR spectrum of **12**.

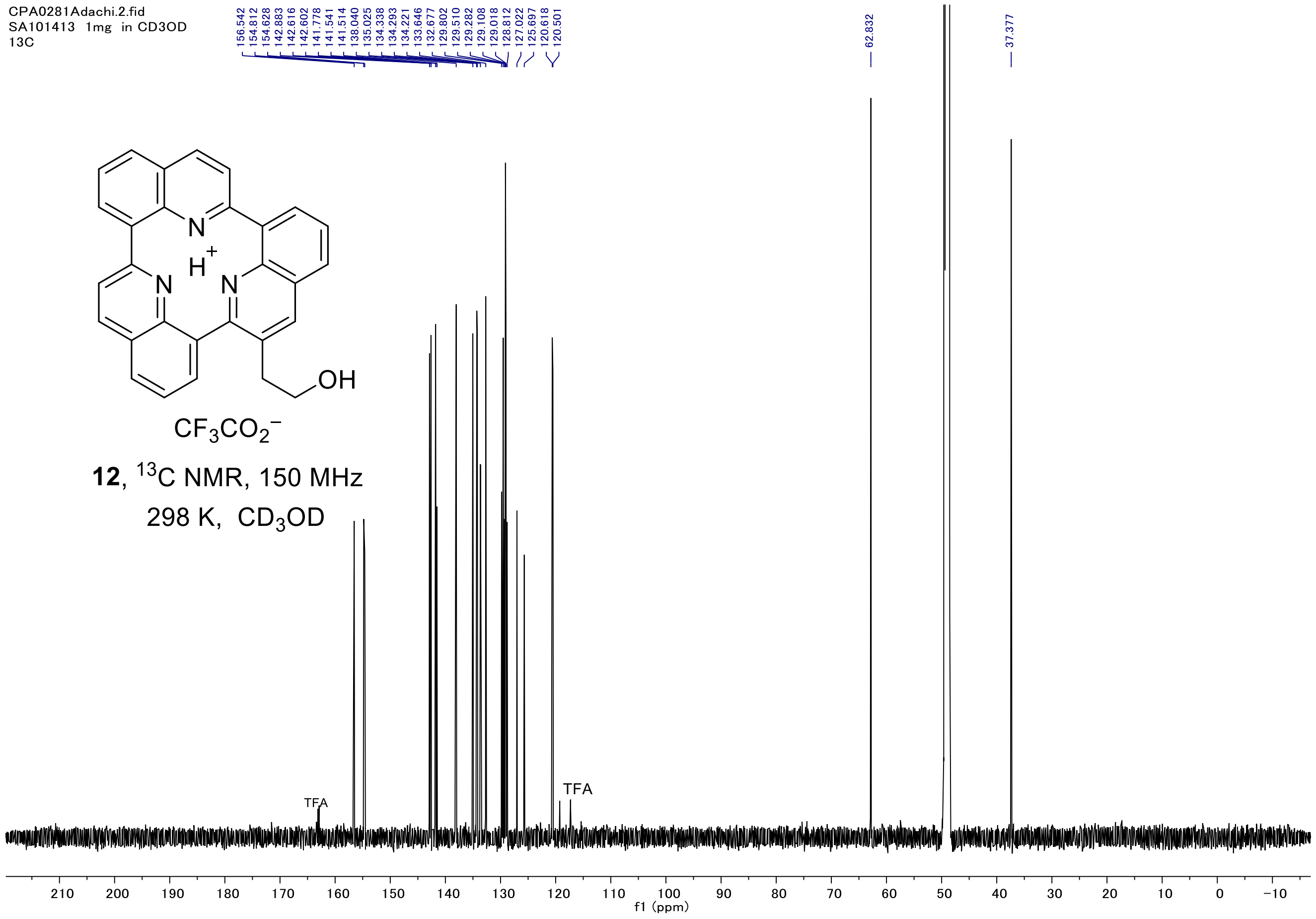

Supplementary Fig. 86 ^{13}C NMR spectrum of **12**.

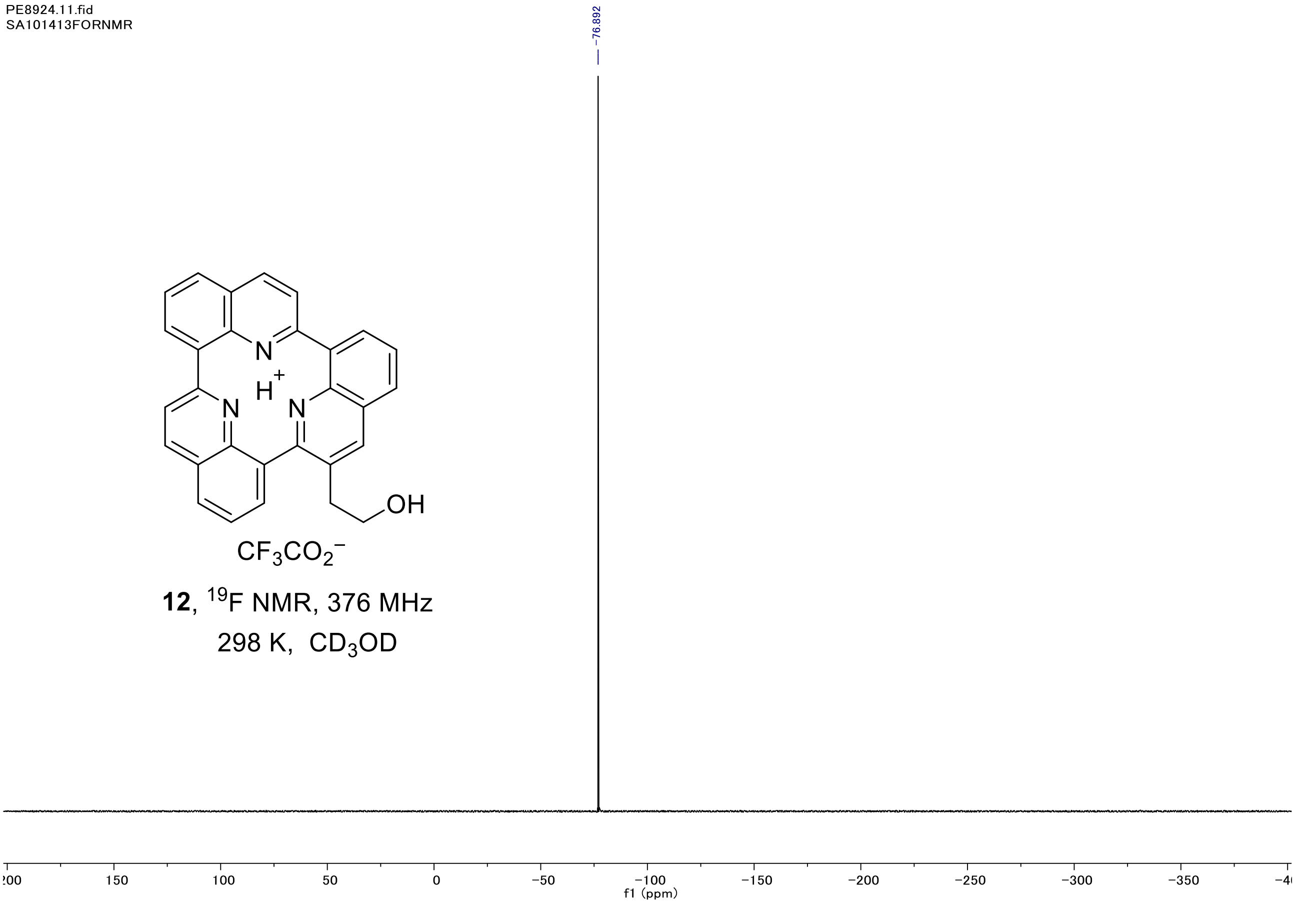

Supplementary Fig. 87 ^{19}F NMR spectrum of **12**.

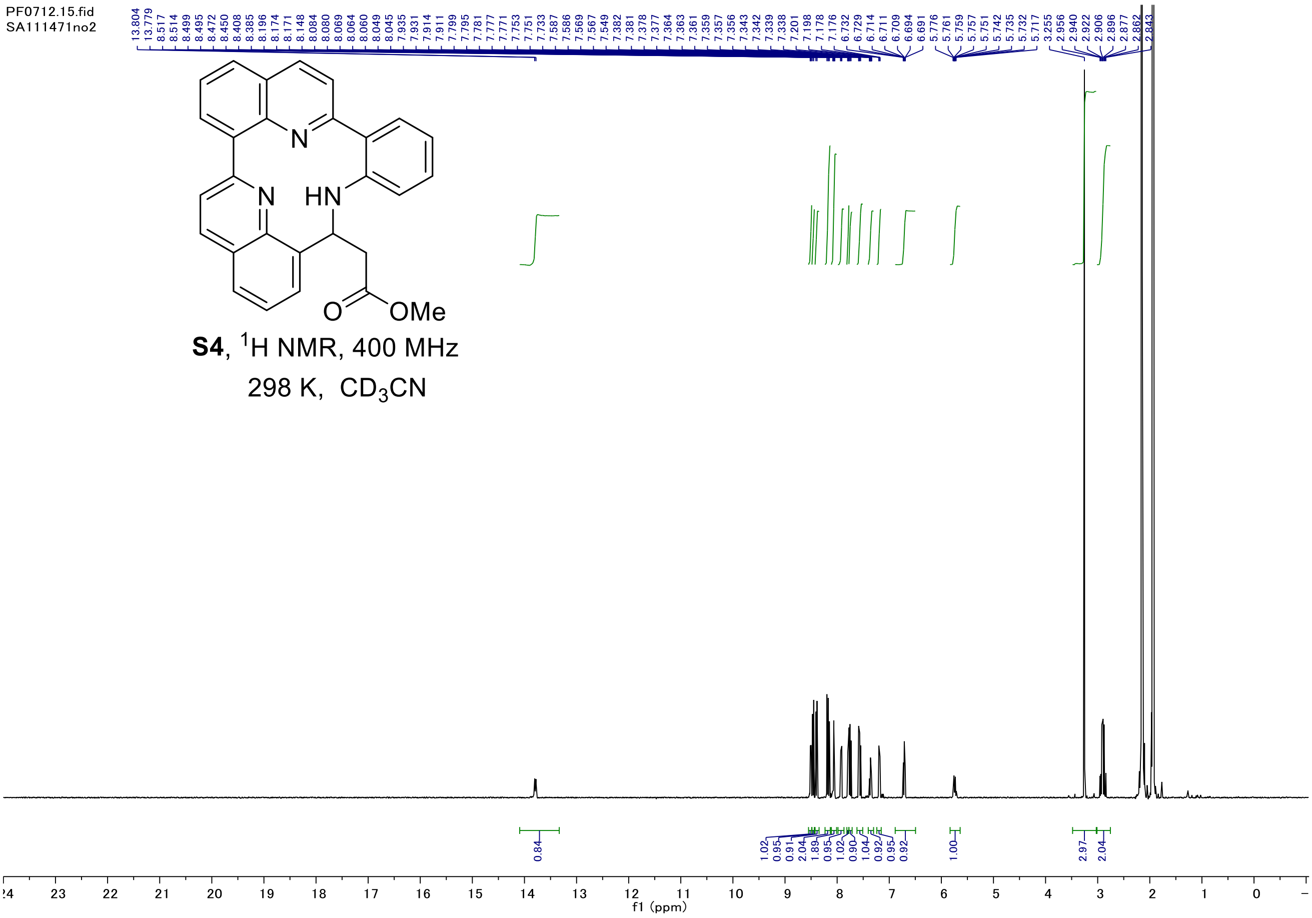

Supplementary Fig. 88 ^{1}H NMR spectrum of **S4**.

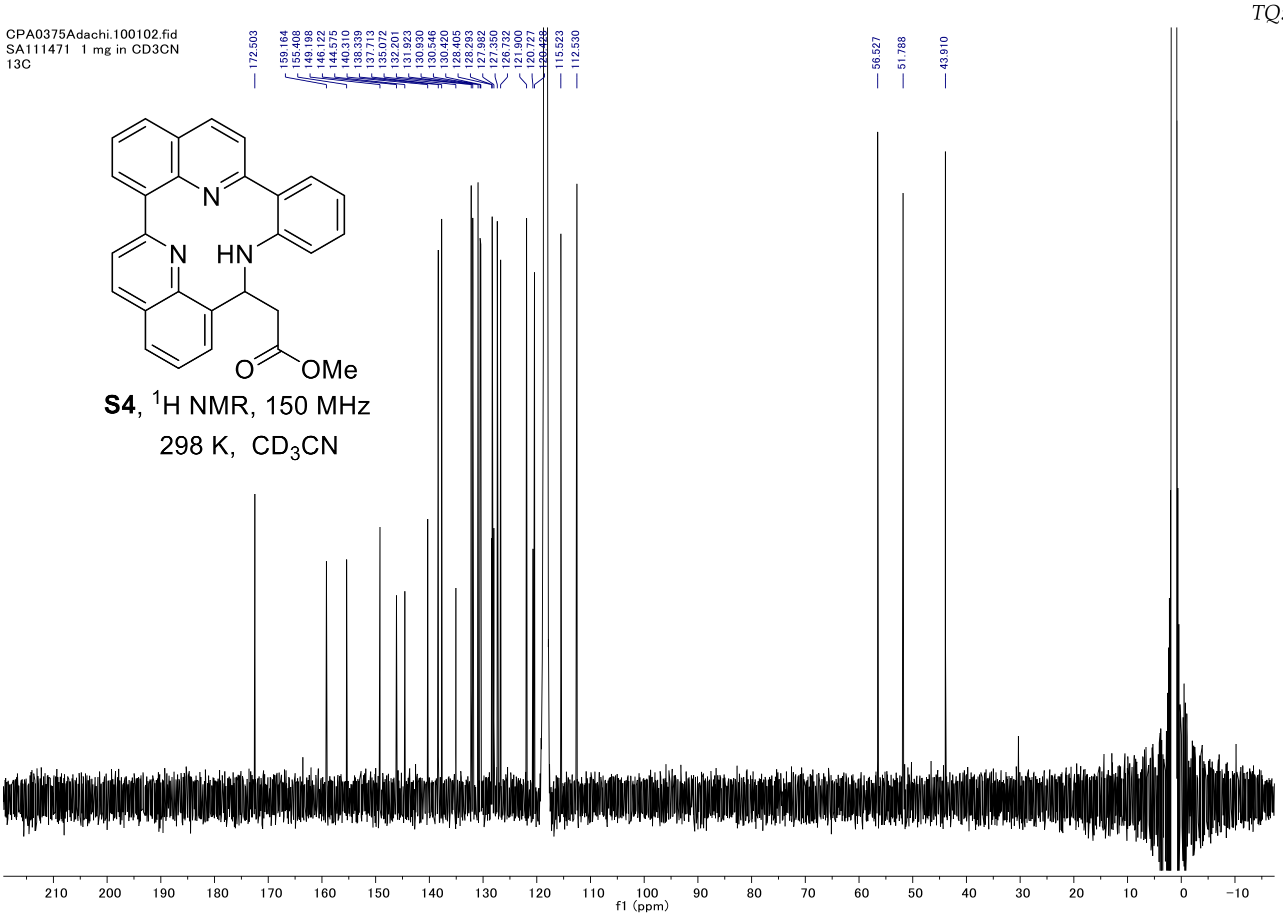

Supplementary Fig. 89 ^{13}C NMR spectrum of **S4**.

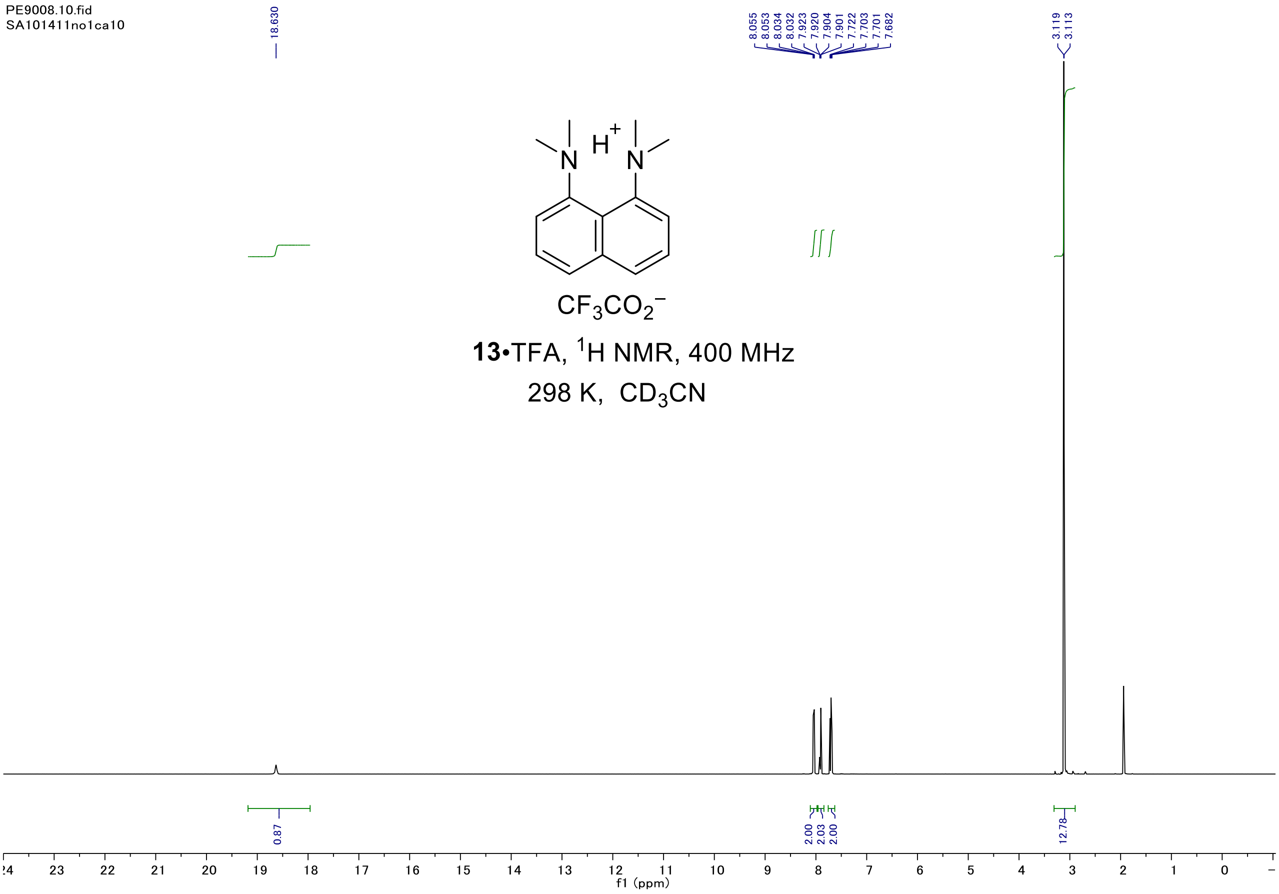

Supplementary Fig. 90 ^{1}H NMR spectrum of **13**•TFA.

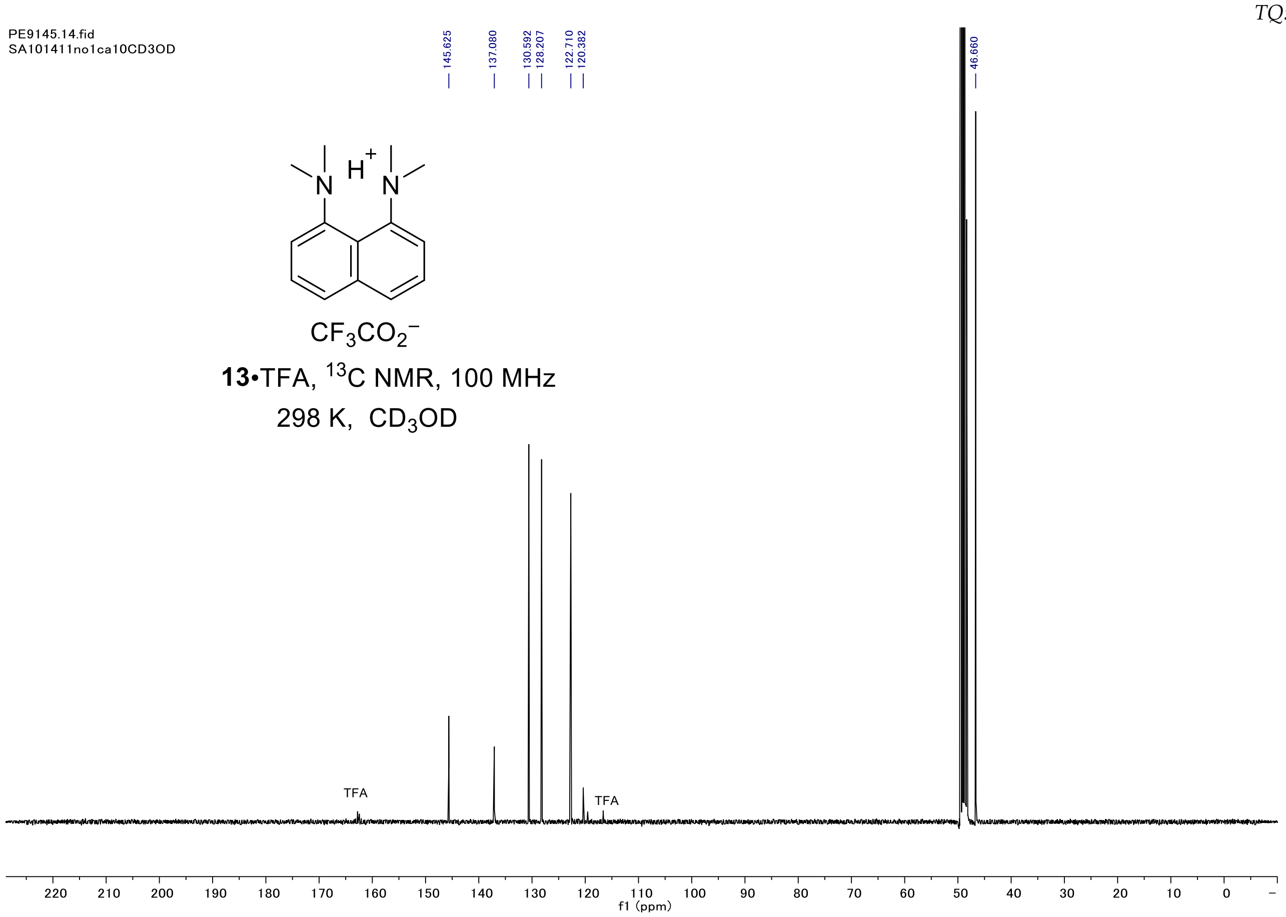

Supplementary Fig. 91 ^{13}C NMR spectrum of **13**•TFA.

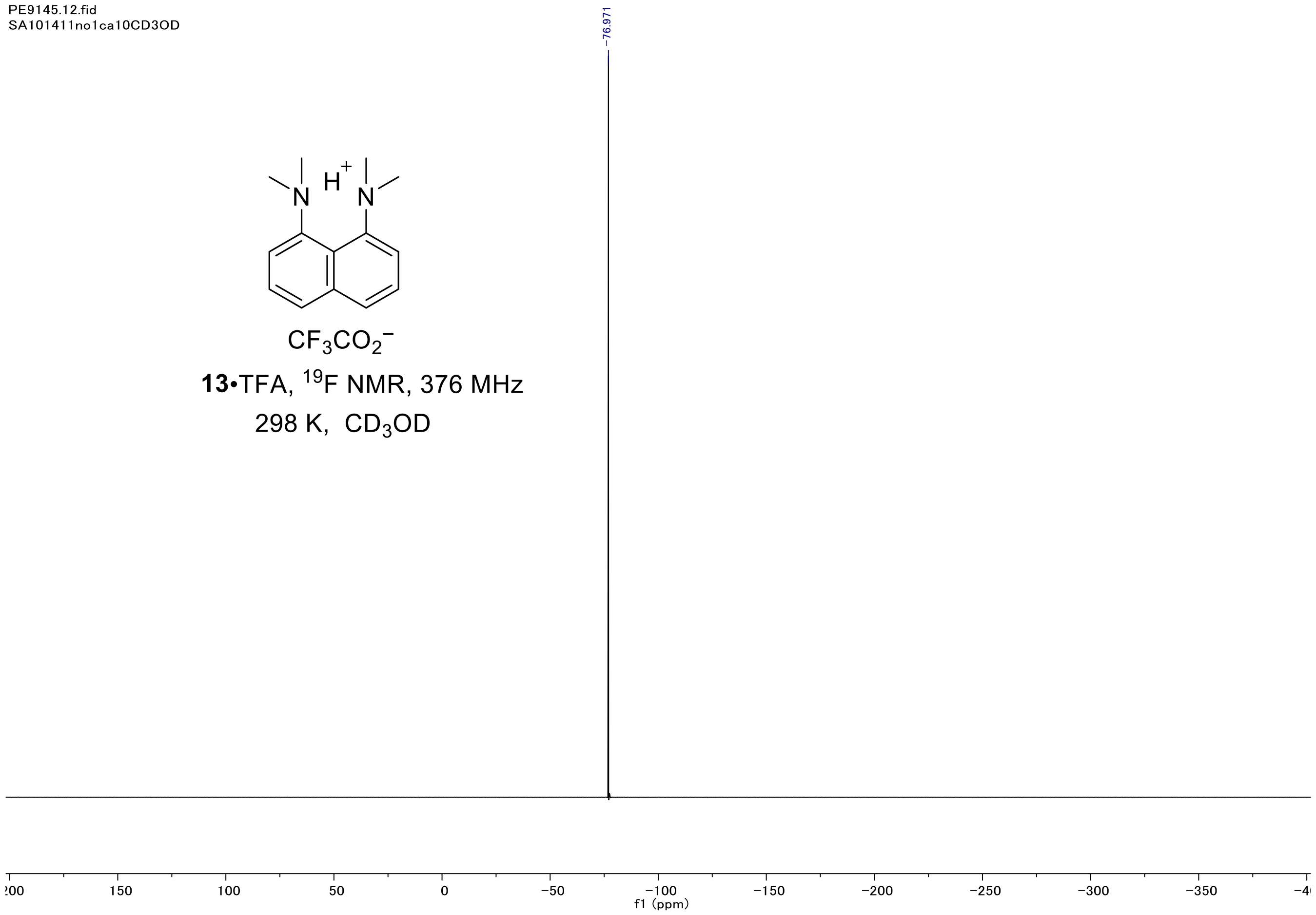

Supplementary Fig. 92 ^{19}F NMR spectrum of **13**•TFA.

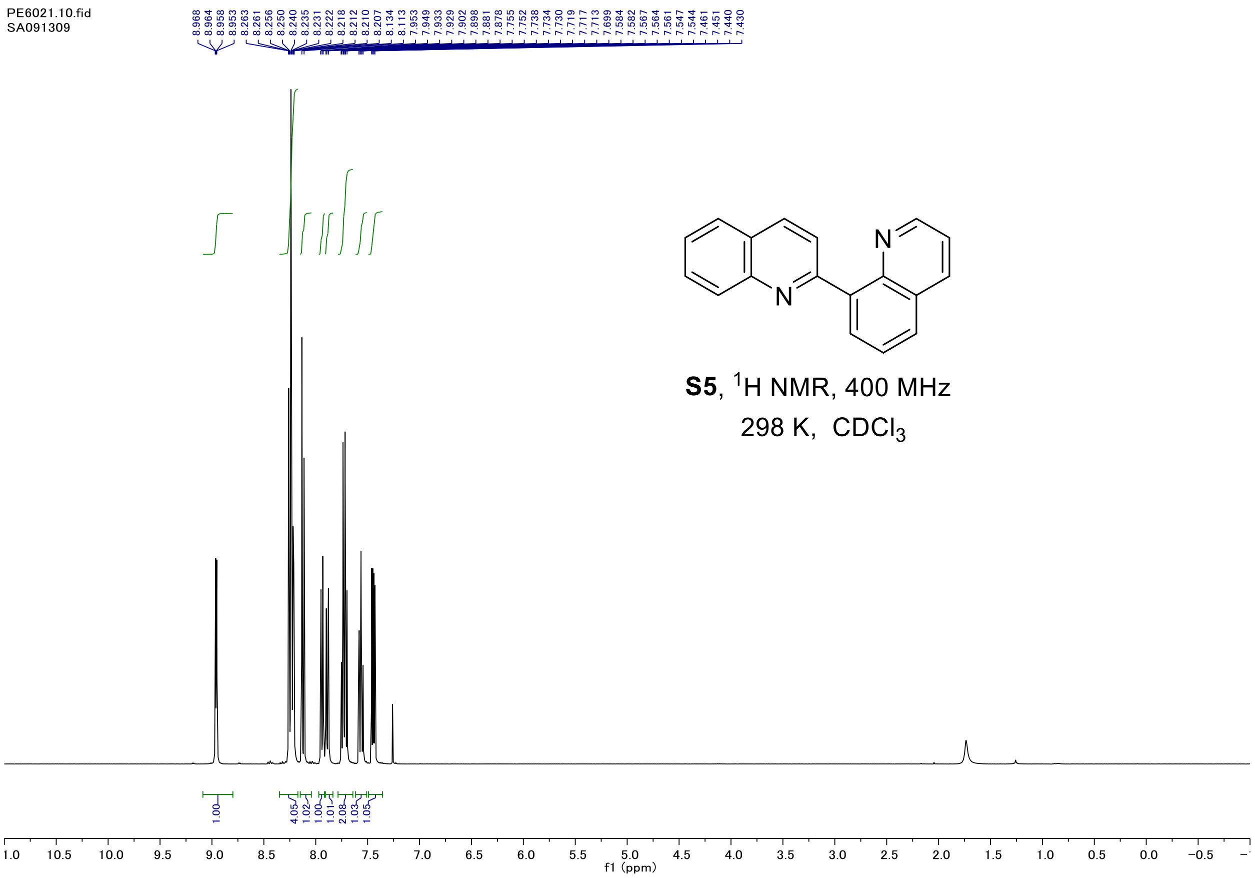

Supplementary Fig. 93 ^{1}H NMR spectrum of **S5**.

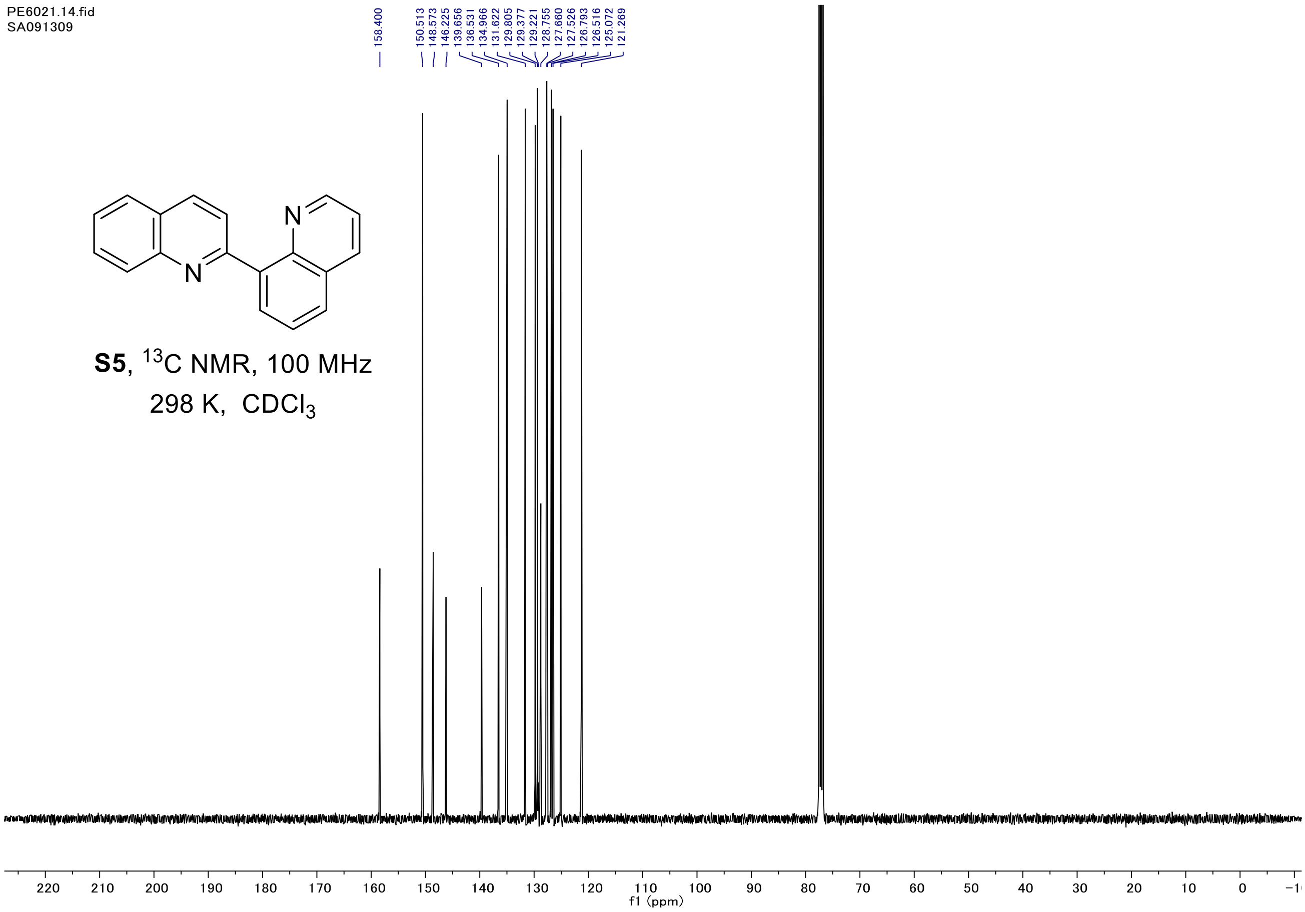

Supplementary Fig. 94 ^{13}C NMR spectrum of **S5**.

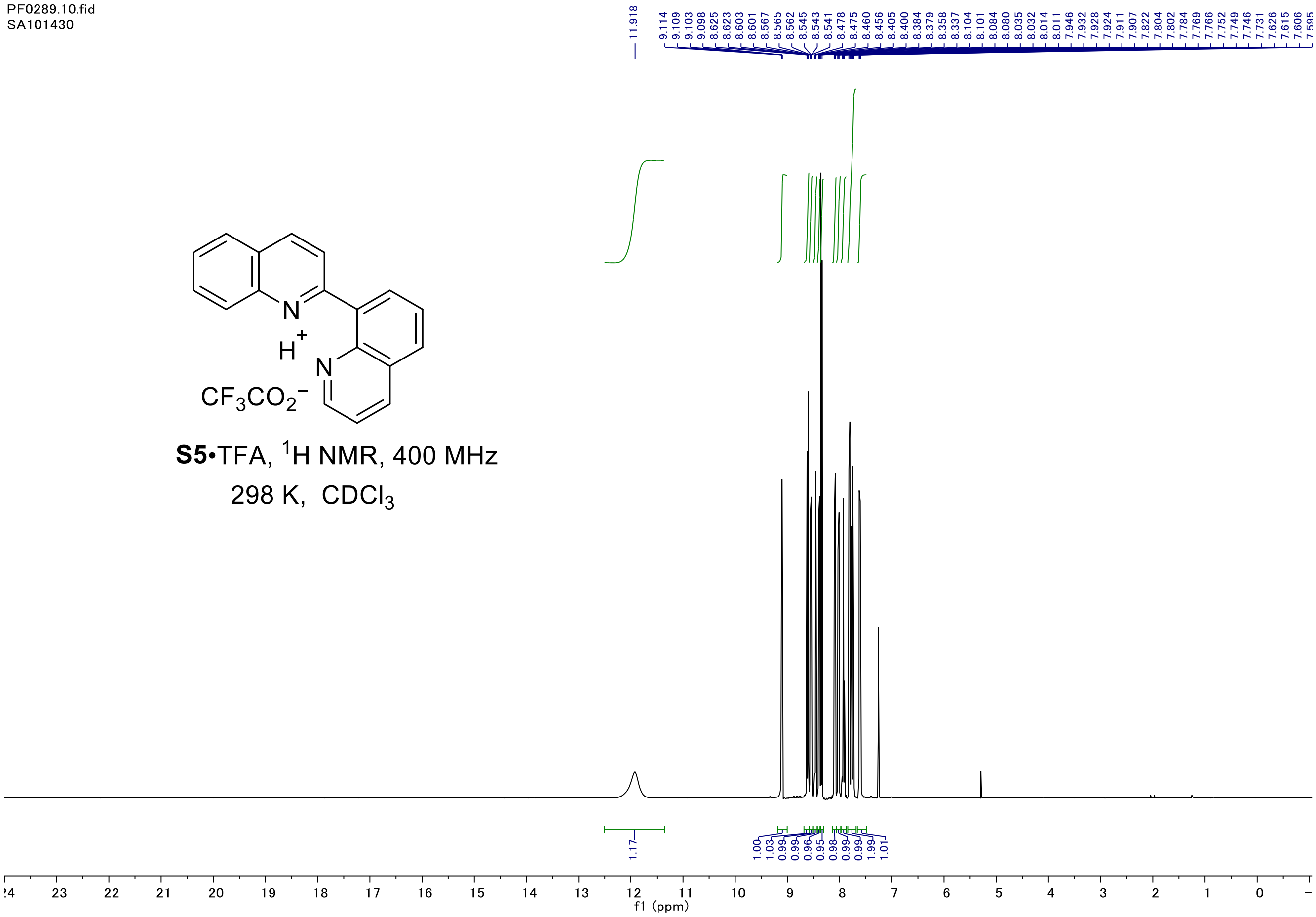

Supplementary Fig. 95 ^{1}H NMR spectrum of **S5**•TFA.

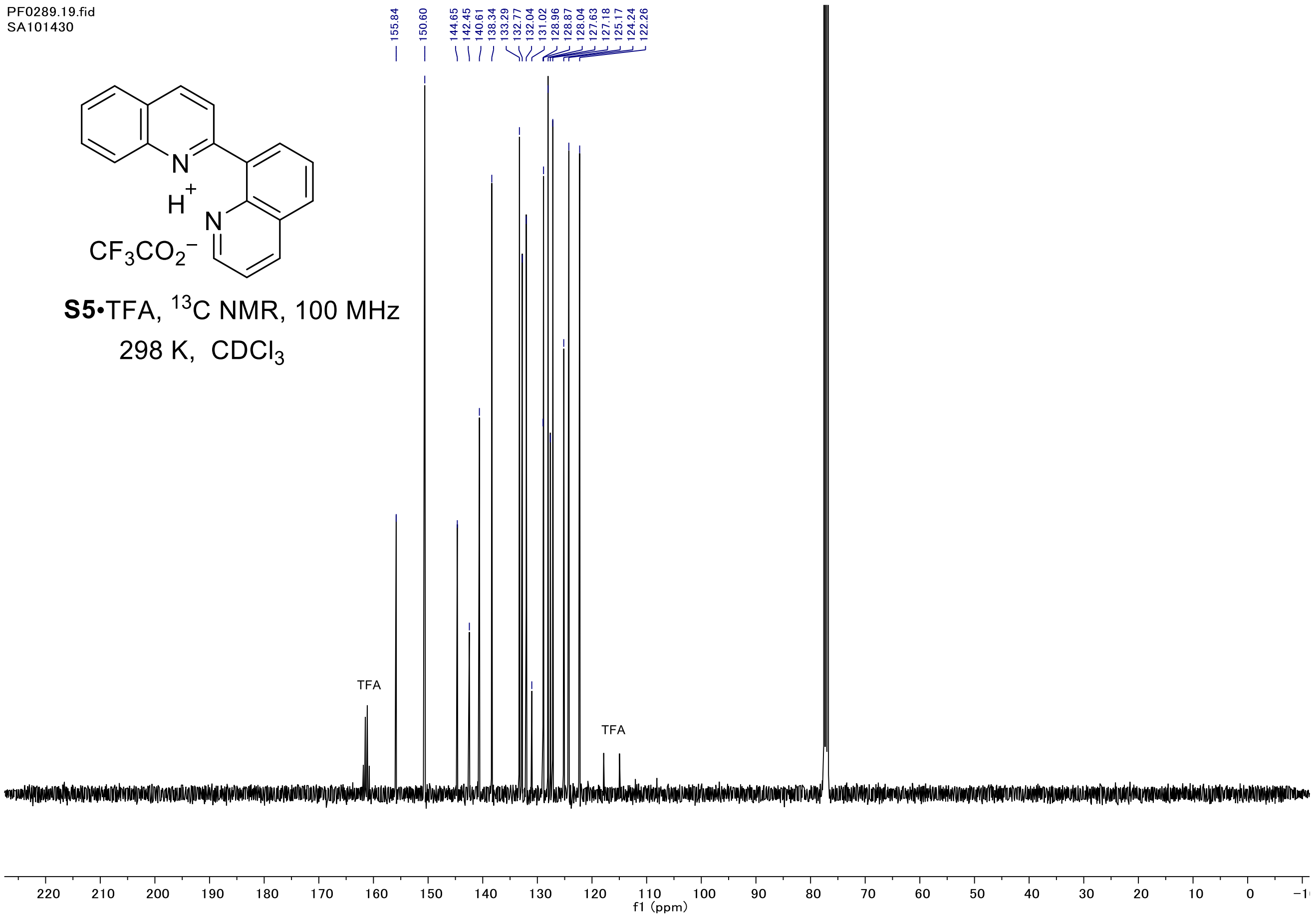

Supplementary Fig. 96 ^{13}C NMR spectrum of **S5**•TFA.

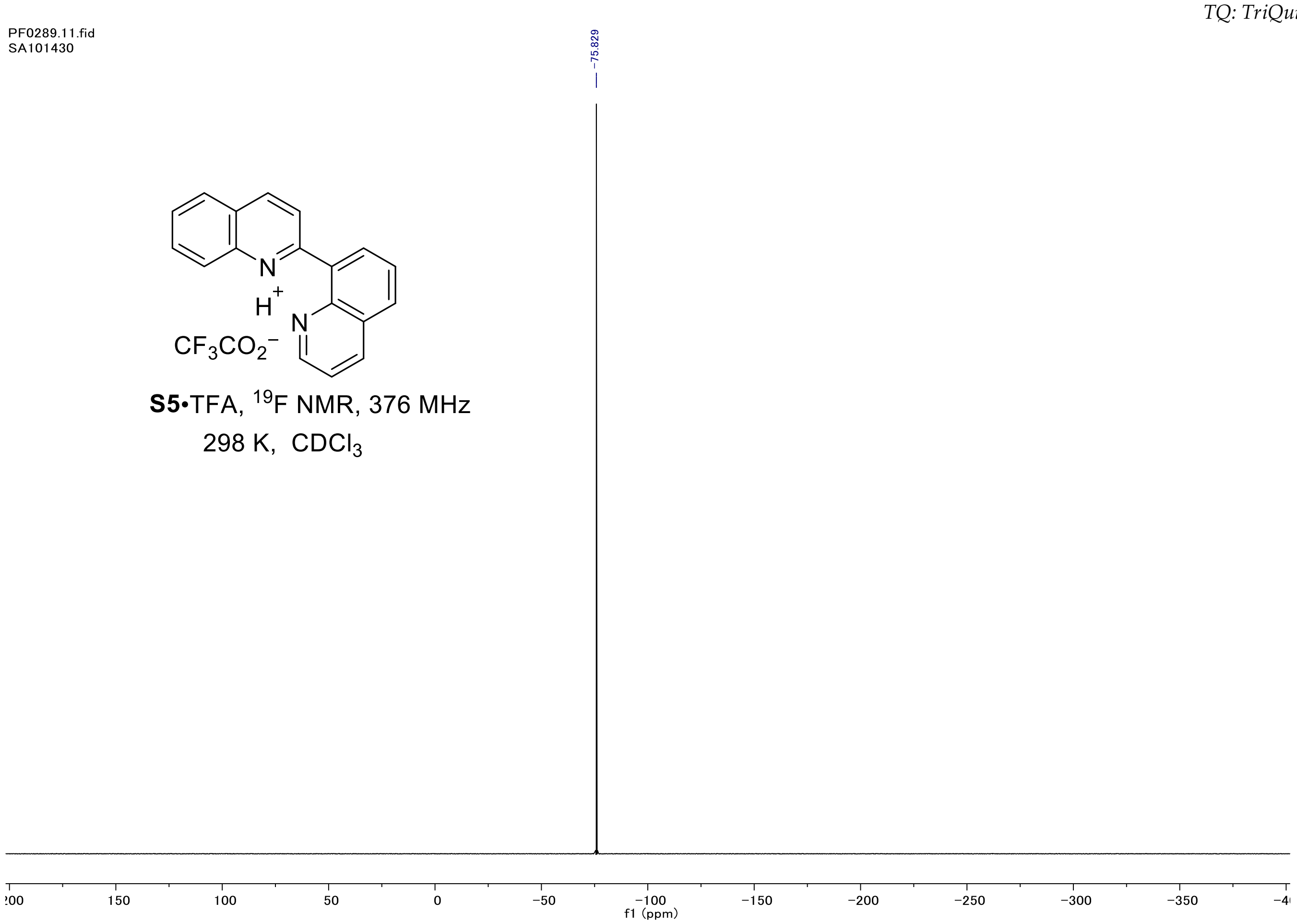

Supplementary Fig. 97 ^{19}F NMR spectrum of **S5**•TFA.

17. Cartesian Coordinates of Optimized Structure

Supplementary Table 5 Cartesian coordinates and energies of DQ-Im•HCl.

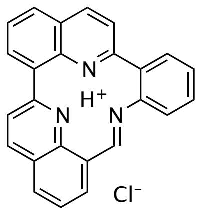

DQ–Im•HCl

B3LYP-D3/6-31G(d)
Zero-point correction= 0.347736 (Hartree/Particle)
Thermal correction to Energy= 0.368768
Thermal correction to Enthalpy= 0.369712
Thermal correction to Gibbs Free Energy= 0.297481
Sum of electronic and zero-point Energies= -1586.482107
Sum of electronic and thermal Energies= -1586.461076
C -0.03740500 4.20429500 0.36282700
C 0.05527200 2.81931300 0.13698200
C -1.17443400 2.10753100 0.00633300
C -2.40620400 2.77325800 0.06361400
C -2.44376900 4.14927000 0.26400000
C -1.25909200 4.86701000 0.42515500
H 0.86737200 4.77993300 0.52067500
H -3.40707100 4.64773900 0.31173000
H -1.28170500 5.93749500 0.60705800
C -2.15619000 -0.10756200 -0.33117700
C -2.04830900 -1.54950100 -0.26911700
C -0.80889300 -2.25076600 -0.07398100
C -3.24164000 -2.27181300 -0.29005200
C -0.82173300 -3.65366400 0.16471000
C -3.23708400 -3.67280300 -0.10947000
H -4.17839100 -1.72341600 -0.40130700
C 0.42958200 -4.24848100 0.46539400
C -2.05846500 -4.35063900 0.12895400
C 1.50628100 -2.09808100 0.16223000
H -4.17999800 -4.21016800 -0.12973000
C 1.57477200 -3.48629900 0.49450100
H -2.06356400 -5.42384000 0.30502300
H 2.51342800 -3.94578800 0.77995500
C 2.72049400 -1.23863600 0.06680500
C 2.67104400 0.20871300 0.02993500
C 3.96762300 -1.84020400 -0.05844800
C 3.88793700 0.93853700 -0.16317100
C 5.16425800 -1.10887100 -0.20475800
H 4.03874300 -2.92182400 -0.08094800
C 3.78890700 2.34820400 -0.29025200
C 5.12827500 0.26385700 -0.26352500
C 1.40620800 2.19017100 0.03270700
H 6.10597600 -1.64172400 -0.29459100
C 2.57193700 2.97371700 -0.22467900
H 4.69387400 2.92357000 -0.46998900
H 6.03721500 0.84362000 -0.40299400
H 2.50438600 4.04183800 -0.38748900
N 1.49455000 0.87289100 0.16201600
N -1.14075700 0.69560300 -0.13722000
N 0.33576400 -1.53950400 -0.10614900
H 0.47249500 -5.31039200 0.69510600
H -3.34573200 2.22363700 -0.02158700
H -3.17609800 0.29771700 -0.46133100
H -0.19465700 0.26871800 -0.01950800
Cl -5.25349400 0.65944800 -0.18433600

Supplementary Table 6 Cartesian coordinates and energies of methyl vinyl ether (MVE).

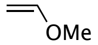

MVE

B3LYP-D3/6-31G(d)
Zero-point correction= 0.084680 (Hartree/Particle)
Thermal correction to Energy= 0.089966
Thermal correction to Enthalpy= 0.090910
Thermal correction to Gibbs Free Energy= 0.056271
Sum of electronic and zero-point Energies= -193.029535
Sum of electronic and thermal Energies= -193.024249
Sum of electronic and thermal Enthalpies= -193.023305
Sum of electronic and thermal Free Energies= -193.057945
C -1.87576700 -0.07290500 0.03631400
C -0.61512400 0.35447100 -0.03359800
H -2.11029200 -1.13093900 0.09005100
H -2.69140100 0.64034000 0.03181700
H -0.36310700 1.41544200 -0.08650200
C 1.72385500 0.10813300 0.04221600
H 2.45990700 -0.69392300 -0.04467700
H 1.89207200 0.83672100 -0.76422700
H 1.85339000 0.61087600 1.01110900
O 0.44520600 -0.50208900 -0.06339500

Supplementary Table 7 Cartesian coordinates and energies of **TS1**.

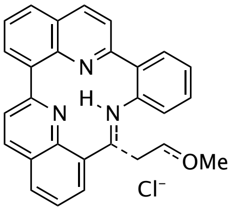

TS1

B3LYP-D3/6-31G(d)
Zero-point correction= 0.436105 (Hartree/Particle)
Thermal correction to Energy= 0.462255
Thermal correction to Enthalpy= 0.463199
Thermal correction to Gibbs Free Energy= 0.380058
Sum of electronic and zero-point Energies= -1779.524157
Sum of electronic and thermal Energies= -1779.498007
Sum of electronic and thermal Enthalpies= -1779.497063
Sum of electronic and thermal Free Energies= -1779.580204
C 3.00755700 -1.56025500 -0.88394600
C 1.75238800 -1.09103900 -0.52800800
C 0.64777500 -2.00395200 -0.53301100
C 0.83549700 -3.34579800 -0.98009900

C 2.13820700 -3.78672600 -1.32742600
C 3.19954000 -2.91060000 -1.26470500
H 3.84304900 -0.86341500 -0.89519000
C -0.33253700 -4.14352600 -1.08720100
H 2.28033200 -4.81342600 -1.65657700
H 4.19671900 -3.24251000 -1.53906000
C -1.56465800 -3.62120200 -0.77112900
C -1.65204300 -2.28688400 -0.26764400
H -0.24606000 -5.16331400 -1.45518900
H -2.45988000 -4.21347900 -0.92342800
C -2.95516200 -1.68425700 0.13081600
C -3.19215900 -0.25737500 0.13948900
C -3.99134500 -2.51463000 0.54115800
C -4.46466100 0.23071100 0.57672200
C -5.24977800 -2.02320600 0.94492300
H -3.82556800 -3.58616200 0.58747500
C -4.63040900 1.64014200 0.63604400
C -5.48610200 -0.66772800 0.96500700
C -2.36812800 1.92447300 -0.16390600
H -6.02024900 -2.72225200 1.25644000
C -3.60318100 2.48056000 0.30025700
H -6.44450700 -0.27122200 1.29141900
H -3.72489900 3.55026200 0.41518100
C -1.20347200 2.79189000 -0.49304600
C 0.14682100 2.31199200 -0.54813100
C -1.40501600 4.17095900 -0.69731600
C 1.20900400 3.22573900 -0.69713600
C -0.35451500 5.05988300 -0.88273400
H -2.41718500 4.55944100 -0.72741900
C 3.15195700 0.30833100 2.02678700
C 0.95852600 4.58164600 -0.85736600
C 1.59786900 0.35286900 -0.20761900
H -0.55733300 6.11474200 -1.04166900
C 1.78579600 0.41871400 1.77742400
H 1.79587100 5.26254500 -0.98066800
H 1.15163700 -0.43304400 2.00442700
N 0.39602400 0.94533600 -0.46894400
N -0.56397700 -1.54547500 -0.14043800
N -2.22223400 0.60693300 -0.25152500
H -5.57884900 2.04271100 0.98426500
H -0.44406400 0.35360500 -0.38944500
H 1.35647700 1.39932500 1.96058600
H 2.23791400 2.87431100 -0.70352600
H 2.49043400 0.93345200 -0.43607200
Cl 4.72624100 1.57696400 -0.27210000
O 3.70801900 -0.88070500 2.08476400
C 5.15687500 -0.92283000 2.11339500
H 5.41843300 -1.97638900 2.01483600
H 5.54343700 -0.32568500 1.28100800
H 5.51551500 -0.53400300 3.07195800
H 3.80196600 1.17679400 2.08497000

Supplementary Table 8 Cartesian coordinates and energies of **TS-1a**.

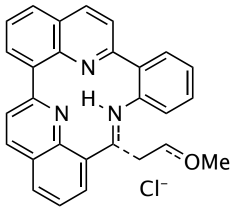

TS1-a

B3LYP-D3/6-31G(d)
Zero-point correction= 0.434795 (Hartree/Particle)
Thermal correction to Energy= 0.461130
Thermal correction to Enthalpy= 0.462074
Thermal correction to Gibbs Free Energy= 0.378392
Sum of electronic and zero-point Energies= -1779.501763
Sum of electronic and thermal Energies= -1779.475428
Sum of electronic and thermal Enthalpies= -1779.474484
Sum of electronic and thermal Free Energies= -1779.558166

C 0.68315300 -4.05980100 -0.43958800
C 0.28601000 -2.74071100 -0.28982600
C -1.11862600 -2.45526500 -0.24588400
C -2.06193800 -3.51055200 -0.43443900
C -1.60463900 -4.84303700 -0.57789300
C -0.25234600 -5.11073600 -0.56715600
H 1.74489800 -4.29465900 -0.47445500
C -3.42847800 -3.12864600 -0.50409900
H -2.32939900 -5.64329500 -0.70638300
H 0.10510600 -6.12983800 -0.68099000
C -3.78689200 -1.80881100 -0.38829900
C -2.77943200 -0.82329800 -0.13721100
H -4.18305600 -3.89198900 -0.67911200
H -4.82420200 -1.51543800 -0.50337500
C -3.11755900 0.60883800 0.06702800
C -2.16782900 1.67176600 -0.16300500
C -4.36963800 0.94574000 0.56742300
C -2.53329000 3.01010800 0.18343700
C -4.73723000 2.27559800 0.85509700
H -5.08356400 0.16002100 0.79385300
C -1.53464800 4.01088800 0.05456300
C -3.82896100 3.29399400 0.67534600
C -0.00902200 2.33250200 -0.75114500
H -5.72753100 2.48248600 1.25004100
C -0.27616500 3.68018300 -0.36563500
H -4.08408800 4.32016500 0.92896000
H 0.51352000 4.42083800 -0.36097700
C 1.35384000 1.92467700 -1.19167400
C 1.85230000 0.59076300 -1.03649600
C 2.22701500 2.88506800 -1.71331400
C 3.20319700 0.32614500 -1.32223900
C 3.55740700 2.60477900 -2.02761700
H 1.85703400 3.89049400 -1.88408500
C 2.98733300 -0.87444800 1.74388200
C 4.04888600 1.32169400 -1.81086700
C 1.33697600 -1.67667900 -0.24667100
H 4.19693400 3.38408200 -2.43077800
C 1.88817900 -1.72131600 1.64949200

H 2.81386400 0.19579900 1.92887900
H 5.08341400 1.07926000 -2.04016600
H 2.06822800 -2.78914000 1.74949700
N 0.99234800 -0.42277100 -0.64058400
N -1.50758000 -1.17533800 -0.05966000
N -0.94757700 1.40198300 -0.68755700
H -1.77078200 5.03093300 0.34774300
H 0.01967100 -0.12340000 -0.45060400
H 0.98724500 -1.29974400 2.09081700
H 3.59696200 -0.67625800 -1.19603400
H 2.28376100 -2.03783500 -0.64086000
O 4.20867400 -1.33441800 1.52717700
C 5.25491400 -0.34054700 1.52437200
H 6.19093900 -0.88742900 1.40757900
H 5.10077200 0.34784300 0.68705300
H 5.25327000 0.21339400 2.46760200
Cl 0.96012700 1.49873900 2.19102800

Supplementary Table 9 Cartesian coordinates and energies of **TS-1b**.

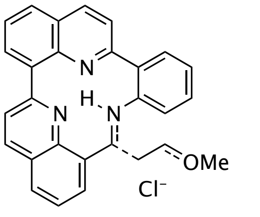

TS1-b

B3LYP-D3/6-31G(d)
Zero-point correction= 0.432796 (Hartree/Particle)
Thermal correction to Energy= 0.459264
Thermal correction to Enthalpy= 0.460208
Thermal correction to Gibbs Free Energy= 0.375379
Sum of electronic and zero-point Energies= -1779.487778
Sum of electronic and thermal Energies= -1779.461310
Sum of electronic and thermal Enthalpies= -1779.460366
Sum of electronic and thermal Free Energies= -1779.545195
C -2.31346800 3.17483900 -0.64646000
C -1.37892700 2.18122500 -0.40829000
C 0.00644000 2.54806700 -0.37159900
C 0.39419700 3.89155100 -0.66130600
C -0.60048200 4.87032500 -0.90323500
C -1.93258700 4.51498800 -0.88259000
H -3.37009500 2.91580500 -0.66259200
C 1.79077900 4.14572500 -0.71848300
H -0.30045700 5.89452900 -1.11107800
H -2.69991500 5.26076000 -1.06902500
C 2.69024100 3.13133800 -0.50137200
C 2.21164200 1.82666800 -0.16008200
H 2.13702800 5.14700500 -0.96418200
H 3.75392300 3.31455200 -0.60541200
C 3.13856500 0.70416800 0.13810400
C 2.75884300 -0.67704400 -0.04080400
C 4.39644900 0.97538000 0.66173800
C 3.66576300 -1.70305400 0.37042500
C 5.30080200 -0.04442900 1.02247100
H 4.68720000 2.00520300 0.84560100
C 3.21262100 -3.04578300 0.27214000
C 4.93874300 -1.36587800 0.88769900
C 1.12785200 -2.24209500 -0.63056500
H 6.26978800 0.22238900 1.43407200
C 1.95334500 -3.32017900 -0.18754600
H 5.61292300 -2.16265000 1.19300300
H 1.57420500 -4.33466800 -0.17508400
C -0.25723900 -2.49045000 -1.11323800
C -1.30792600 -1.52960100 -0.98405100
C -0.58087500 -3.74264100 -1.65529400
C -2.62210300 -1.90258700 -1.30880100
C -1.87948300 -4.08724300 -2.01803400
H 0.21217200 -4.46816200 -1.80597400
C -3.66874500 0.67118000 1.80942900
C -2.90563700 -3.16446900 -1.81803800
C -1.85078000 0.77090500 -0.25104900
H -2.08738700 -5.06734200 -2.43670100
C -2.30747100 0.73897400 1.68082800
H -3.93217000 -3.42211400 -2.06521700
H -1.84425500 1.59857700 2.15953200
N -0.99855100 -0.23368300 -0.57545400
N 0.91362000 1.58836300 -0.08735700
N 1.55803200 -0.99105800 -0.58373000
H 3.86500800 -3.84714000 0.61099300
H 0.00323700 -0.06722200 -0.38691700
H -1.69999400 -0.23024100 1.81265500
H -3.43668700 -1.20795400 -1.14286200
H -2.86224700 0.62809400 -0.61721900
Cl -0.48797200 -1.67579900 2.24432900
O -4.35323400 -0.31050200 1.20393300
C -5.73662700 -0.44493100 1.54229800
H -5.84553200 -0.79938300 2.57311900
H -6.26717800 0.50835400 1.41940700
H -6.14816300 -1.18578600 0.85585400
H -4.27532500 1.41494200 2.33298900

Supplementary Table 10 Cartesian coordinates and energies of **TS-1c**.

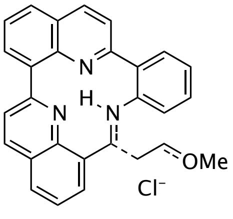

TS1-c

B3LYP-D3/6-31G(d)
Zero-point correction= 0.435856 (Hartree/Particle)
Thermal correction to Energy= 0.462171
Thermal correction to Enthalpy= 0.463115
Thermal correction to Gibbs Free Energy= 0.379338
Sum of electronic and zero-point Energies= -1779.521589
Sum of electronic and thermal Energies= -1779.495275
Sum of electronic and thermal Enthalpies= -1779.494330
Sum of electronic and thermal Free Energies= -1779.578108

C -1.83699400 3.33442900 -0.43797900
C -0.97838700 2.27413800 -0.20141500
C 0.43306400 2.50860900 -0.20620100
C 0.93862500 3.79908100 -0.54343700
C 0.02631300 4.85911000 -0.78019100
C -1.33162600 4.62845400 -0.71308000
H -2.91065000 3.15379900 -0.42688000
C 2.34927300 3.91238900 -0.65435100
H 0.41161000 5.84596600 -1.02523500
H -2.03072600 5.43866200 -0.89824800
C 3.15402200 2.81604500 -0.44964700
C 2.56276700 1.57497700 -0.05928800
H 2.78406000 4.86854100 -0.93635000
H 4.22497900 2.89028200 -0.60313400
C 3.38383900 0.36127200 0.20634200
C 2.86783200 -0.97839300 0.03606700
C 4.68828100 0.50155800 0.66269100
C 3.70389100 -2.09175300 0.36548300
C 5.51356500 -0.60379900 0.95746400
H 5.08559400 1.49708400 0.83499700
C 3.12909700 -3.38636400 0.25184600
C 5.02905300 -1.88449700 0.81624600
C 1.06625500 -2.38580000 -0.51150800
H 6.52476400 -0.43376400 1.31512000
C 1.82983700 -3.54231500 -0.15250800
H 5.64669800 -2.74576100 1.05936000
H 1.38402400 -4.52928300 -0.17283600
C -0.35459900 -2.49447300 -0.93892000
C -1.28832300 -1.41178100 -0.84708700
C -0.83700200 -3.72926300 -1.41726000
C -2.63982800 -1.62596000 -1.17869600
C -2.16853400 -3.92848200 -1.75405600
H -0.14126600 -4.55119400 -1.55079000
C -2.76388300 0.06042000 2.17742400
C -3.07259000 -2.86926400 -1.61620100
C -1.56225100 0.91429700 -0.00528400
H -2.49755300 -4.89407700 -2.12640600
C -1.61117300 0.81888000 1.95942300
H -4.12082600 -3.00317300 -1.86924400
H -1.67802400 1.85419400 2.27719800
N -0.83624100 -0.15106200 -0.46475400
N 1.25208100 1.46900600 0.07984600
N 1.60873100 -1.17626800 -0.42753600
H 3.72964200 -4.25247400 0.52040400
H 0.18666500 -0.07284800 -0.38353600
H -0.64517500 0.34381300 2.09864600
H -3.35843900 -0.81618200 -1.08158500
H -2.63488300 0.88823600 -0.19886300
Cl -4.91327300 0.96801500 0.14437600
O -2.70719800 -1.24423900 2.07300800
C -3.97113200 -1.95148900 1.97650900
H -4.41840400 -2.02779500 2.97227000
H -4.62859500 -1.41108800 1.28717600
H -3.71894600 -2.93945300 1.59280400
H -3.73967400 0.52229100 2.30947200

Supplementary Table 11 Cartesian coordinates and energies of **INT1**.

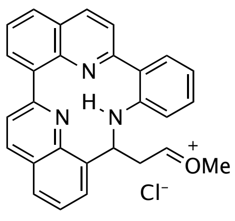

INT1

B3LYP-D3/6-31G(d)

Zero-point correction=	0.439185 (Hartree/Particle)
Thermal correction to Energy=	0.464664
Thermal correction to Enthalpy=	0.465608
Thermal correction to Gibbs Free Energy=	0.383383
Sum of electronic and zero-point Energies=	-1779.558044
Sum of electronic and thermal Energies=	-1779.532565
Sum of electronic and thermal Enthalpies=	-1779.531621
Sum of electronic and thermal Free Energies=	-1779.613846

C 3.23394200 -0.75280200 -1.41324000
C 1.99260000 -0.56458300 -0.84113300
C 1.09153500 -1.67513300 -0.80134800
C 1.45838900 -2.91912400 -1.40131600
C 2.75272800 -3.06867100 -1.95788600
C 3.62471700 -2.00285900 -1.95177200
H 3.93394200 0.07764300 -1.44395000
C 0.45781900 -3.92531000 -1.43941500
H 3.03522100 -4.02073300 -2.40118200
H 4.61602600 -2.10686400 -2.38428500
C -0.79726700 -3.67585400 -0.93956700
C -1.06546900 -2.41557900 -0.32052300
H 0.68541600 -4.88105200 -1.90645800
H -1.58335400 -4.41679700 -1.03717000
C -2.41243100 -2.09421500 0.22694300
C -2.94057900 -0.74963100 0.24316500
C -3.19972000 -3.11241700 0.75092300
C -4.23577300 -0.52357900 0.80751700
C -4.48274700 -2.87997200 1.28648300
H -2.80496600 -4.12369300 0.78087000
C -4.68953800 0.82340300 0.85585300
C -4.99541100 -1.60164500 1.31678900
C -2.62522700 1.54160700 -0.18758000
H -5.05330600 -3.71234700 1.68817900
C -3.90196500 1.84255300 0.39566600
H -5.97696700 -1.40344900 1.74129200
H -4.23336500 2.86791600 0.50389500
C -1.70740900 2.61726800 -0.62828300
C -0.28415100 2.41728500 -0.76716400
C -2.21014600 3.91962000 -0.82972200
C 0.53516900 3.55772700 -0.95518600
C -1.39307300 5.01296800 -1.07284200
H -3.28373200 4.07740100 -0.80471800
C 2.97984900 0.63172500 1.92781500
C -0.00561300 4.82344300 -1.10535400
C 1.59288000 0.80355300 -0.29517000
H -1.82430000 5.99741300 -1.22657400
C 1.64488100 0.89247200 1.25402500

H 0.65795500 5.66942800 -1.26713500
H 0.93110100 0.16958600 1.66463800
N 0.24899600 1.15647700 -0.74391200
N -0.12587000 -1.49115000 -0.23125400
N -2.21743800 0.27721500 -0.26992800
H -5.66158900 1.03422900 1.29641000
H -0.44260100 0.42294700 -0.56710200
H 1.31321300 1.89712200 1.53632100
H 1.61080000 3.42999200 -1.01462700
H 2.30966400 1.52390200 -0.69445800
Cl 4.29048100 1.82362100 1.21827600
O 3.35964100 -0.67057000 1.76771400
C 4.47675800 -1.09320600 2.54347900
H 4.56525800 -2.17004400 2.38954800
H 5.39216200 -0.59198500 2.20915400
H 4.31815600 -0.88423600 3.61147100
H 2.97165000 0.93455600 2.98221100

Supplementary Table 12 Cartesian coordinates and energies of **TS2**.

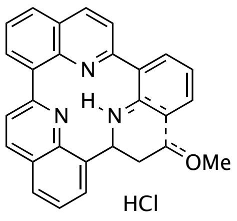

TS2

B3LYP-D3/6-31G(d)
Zero-point correction= 0.437942 (Hartree/Particle)
Thermal correction to Energy= 0.463208
Thermal correction to Enthalpy= 0.464152
Thermal correction to Gibbs Free Energy= 0.382976
Sum of electronic and zero-point Energies= -1779.516325
Sum of electronic and thermal Energies= -1779.491059
Sum of electronic and thermal Enthalpies= -1779.490115
Sum of electronic and thermal Free Energies= -1779.571291
C -1.61945700 3.55413100 -0.31612200
C -0.84810300 2.41197100 -0.18726500
C 0.56829000 2.55279700 -0.04334800
C 1.15919600 3.85389000 -0.10168500
C 0.33080300 4.99575300 -0.22026300
C -1.03564100 4.84288500 -0.31256500
H -2.69726300 3.44469800 -0.43262500
C 2.57675300 3.90426400 -0.07461800
H 0.78710600 5.98236000 -0.25008900
H -1.67758800 5.71355400 -0.41018300
C 3.31663900 2.75062100 0.00663700
C 2.64665800 1.49049300 0.12050200
H 3.07274600 4.86919400 -0.15156200
H 4.39750800 2.80486900 -0.04392300
C 3.41604900 0.21350200 0.22850600
C 2.86238900 -1.09073100 -0.07357200
C 4.74477200 0.25822600 0.63867200
C 3.70986700 -2.24374800 0.00141000
C 5.56648900 -0.88408700 0.71841000
H 5.17851300 1.20716900 0.93372400
C 3.12590300 -3.49862200 -0.31804600
C 5.06346200 -2.12163100 0.38994100
C 0.99421800 -2.41256700 -0.62325000
H 6.59674800 -0.77588500 1.04438600
C 1.79491300 -3.59816200 -0.62212700
H 5.68462800 -3.01249200 0.44142300
H 1.37292700 -4.56752400 -0.85661500
C -0.48234100 -2.44755900 -0.80270100
C -1.29899300 -1.24774800 -0.80421600
C -1.15817000 -3.66379600 -0.87719600
C -2.72842300 -1.36272600 -0.81403800
C -2.55959500 -3.77782200 -0.94739000
H -0.58983200 -4.58718600 -0.86387800
C -3.02888300 -0.48769000 1.23943300
C -3.33426500 -2.63583900 -0.92486300
C -1.57221700 1.07762200 -0.22456600
H -3.01228500 -4.76151400 -1.02602700
C -2.12096800 0.68631400 1.20152600
H -4.41673600 -2.68648200 -1.00880300
H -2.74285400 1.53209900 1.51750000
N -0.76611000 -0.01917300 -0.70628300
N 1.32643500 1.43652300 0.11041300
N 1.55561400 -1.22988500 -0.40295100
H 3.75371100 -4.38654300 -0.30485800
H 0.24236100 0.02512000 -0.49836600
H -1.27873500 0.53671300 1.88306700
H -3.34166000 -0.49917900 -1.08383800
H -2.46149300 1.21840300 -0.84544200
Cl -4.92374300 1.39624900 -0.50259300
O -2.73838000 -1.40822400 2.13989300
C -3.75828600 -2.39004100 2.40763900
H -3.41264900 -2.95261500 3.27510100
H -4.71093300 -1.89694100 2.62578600
H -3.86238400 -3.04899400 1.54230500
H -4.06908500 -0.31971600 0.94052200

Supplementary Table 13 Cartesian coordinates and energies of **INT2**.

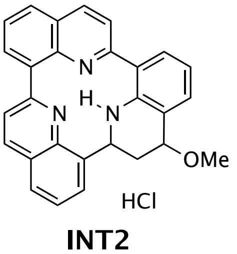

INT2

B3LYP-D3/6-31G(d)
Zero-point correction= 0.434978 (Hartree/Particle)
Thermal correction to Energy= 0.461176
Thermal correction to Enthalpy= 0.462120
Thermal correction to Gibbs Free Energy= 0.377564
Sum of electronic and zero-point Energies= -1779.559305
Sum of electronic and thermal Energies= -1779.533108
Sum of electronic and thermal Enthalpies= -1779.532163
Sum of electronic and thermal Free Energies= -1779.616719

C 0.18053500 4.15312100 0.12426700
C -0.12626200 2.81231500 0.27371600
C -1.51510100 2.44160600 0.18477400
C -2.50274000 3.43362400 -0.11702600
C -2.12668900 4.79159400 -0.24949900
C -0.80443400 5.14090800 -0.11818400
H 1.21179900 4.48185700 0.17850800
C -3.83421100 2.98017400 -0.30566600
H -2.88773300 5.53760400 -0.46541800
H -0.49716200 6.17768800 -0.22269600
C -4.13255300 1.64427500 -0.21747700
C -3.09750300 0.72163100 0.12712300
H -4.60644000 3.70358400 -0.55746000
H -5.13440300 1.29018600 -0.43361800
C -3.38049700 -0.73831100 0.21130600
C -2.39051800 -1.73691500 -0.11279500
C -4.64274200 -1.17430400 0.59694500
C -2.74456500 -3.12092000 -0.03628900
C -4.98338500 -2.54048000 0.66578200
H -5.39199000 -0.44294400 0.88498700
C -1.72038700 -4.06370700 -0.32973700
C -4.04945900 -3.50331000 0.34965600
C -0.17673100 -2.24103400 -0.70948000
H -5.98292700 -2.82589300 0.98057400
C -0.45538000 -3.64613000 -0.64320600
H -4.29682300 -4.56080400 0.40688800
H 0.33228700 -4.37455000 -0.79374000
C 1.19176800 -1.73146200 -0.93641800
C 1.60275400 -0.42112400 -0.49380000
C 2.17029500 -2.57120600 -1.50125500
C 2.99474200 -0.11466200 -0.47475000
C 3.51230400 -2.21423900 -1.57086700
H 1.86876700 -3.53441500 -1.90064100
C 3.42168000 1.14824900 0.24066400
C 3.91806500 -0.98417500 -1.02421600
C 0.98701100 1.77876600 0.54841200
H 4.23522300 -2.87748800 -2.03538000
C 2.37276600 2.25614600 0.08755500
H 3.52628700 0.90961900 1.31506000
H 4.96467600 -0.69976300 -1.01512500
H 2.68777800 3.12623000 0.67015000
N 0.69484000 0.49543200 -0.07784600
N -1.86316100 1.14028400 0.35131400
N -1.14328900 -1.35840700 -0.47959300
H -1.94981200 -5.12498300 -0.26533000
H -0.28166800 0.18902100 -0.04977100
H 2.33250500 2.54832400 -0.96756700
H 3.39694900 -2.87516000 0.58525200
H 1.01973300 1.63903700 1.64415100
O 4.68568400 1.56806400 -0.26226700
C 5.45083600 2.33231100 0.64892800
H 6.40246500 2.54885500 0.15613000
H 4.96881700 3.28687600 0.91045300
H 5.64803400 1.77677500 1.57962500
Cl 3.31834300 -3.14010500 1.86030600

Supplementary Table 14 Cartesian coordinates and energies of **TS3**.

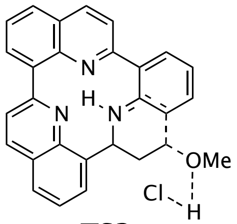

TS3

B3LYP-D3/6-31G(d)

Zero-point correction=	0.436877 (Hartree/Particle)
Thermal correction to Energy=	0.462440
Thermal correction to Enthalpy=	0.463384
Thermal correction to Gibbs Free Energy=	0.380274
Sum of electronic and zero-point Energies=	-1779.527907
Sum of electronic and thermal Energies=	-1779.502344
Sum of electronic and thermal Enthalpies=	-1779.501400
Sum of electronic and thermal Free Energies=	-1779.584510

C -0.82967500 3.73521000 -0.58299100
C -0.20695600 2.49866700 -0.58698900
C 1.19482500 2.46478400 -0.26779400
C 1.86454500 3.66618000 0.12805200
C 1.17456800 4.90136300 0.11553000
C -0.14861600 4.93129400 -0.25348400
H -1.88162100 3.81689900 -0.83063900
C 3.21243800 3.53833200 0.55171200
H 1.69913400 5.80881000 0.40391400
H -0.69346000 5.87055400 -0.27298400
C 3.82424100 2.31129900 0.56341500
C 3.10912100 1.16915900 0.08585000
H 3.74324000 4.42296300 0.89555600
H 4.83041800 2.20982300 0.95299500
C 3.75815200 -0.17155500 0.04985300
C 3.01840500 -1.40975300 0.11790200
C 5.14254700 -0.25175900 -0.05926200
C 3.73394300 -2.64906900 0.09520200
C 5.83751100 -1.47740500 -0.07414000
H 5.72101400 0.65997900 -0.16631800
C 2.96145300 -3.84083600 0.13057100
C 5.14469100 -2.66416100 0.00926600
C 0.94422000 -2.51200800 0.16479700
H 6.91974300 -1.47554200 -0.16317900
C 1.59442700 -3.78833600 0.14645500
H 5.66493600 -3.61871000 -0.00804900
H 1.02757200 -4.71026000 0.12061300
C -0.53044600 -2.37815400 0.07603600
C -1.18428800 -1.11508000 -0.19987100
C -1.34769000 -3.50863900 0.15101300
C -2.60368000 -1.11406500 -0.45833200
C -2.73274300 -3.50062300 -0.08467700
H -0.89533800 -4.46369700 0.39551100
C -3.26806100 0.11001500 -0.62411500
C -3.35556600 -2.31768100 -0.41128000
C -1.00744100 1.23316500 -0.94233900
H -3.29838000 -4.42295800 -0.00735000
C -2.50913800 1.40413000 -0.65339000

H -4.29077800 0.09264600 -0.99428100
H -4.42995800 -2.25678300 -0.57785600
H -2.96772000 2.06993700 -1.38985700
N -0.50613400 0.04179800 -0.25034500
N 1.85614500 1.28100100 -0.32324400
N 1.66468000 -1.39642100 0.18906600
H 3.47413300 -4.79971900 0.11545600
H 0.51798700 -0.02905900 -0.16134100
H -2.62728800 1.88272900 0.32436800
H -0.88021800 1.05470300 -2.02534900
O -4.25430600 0.25461200 1.28636500
C -4.79742000 1.51146200 1.68029900
H -5.43665600 1.37879500 2.56266000
H -3.97207100 2.17935900 1.94955000
H -5.40172900 1.95687100 0.87975900
Cl -6.51730500 -0.79664800 -0.21841400
H -5.05739900 -0.26147100 0.90685700

Supplementary Table 15 Cartesian coordinates and energies of **INT3**.

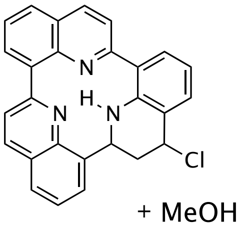

INT3

B3LYP-D3/6-31G(d)

Zero-point correction=	0.436835 (Hartree/Particle)
Thermal correction to Energy=	0.463426
Thermal correction to Enthalpy=	0.464370
Thermal correction to Gibbs Free Energy=	0.378950
Sum of electronic and zero-point Energies=	-1779.528355
Sum of electronic and thermal Energies=	-1779.501764
Sum of electronic and thermal Enthalpies=	-1779.500820
Sum of electronic and thermal Free Energies=	-1779.586240

C -0.90176500 3.69364400 -0.60186100
C -0.24591000 2.47500700 -0.62575800
C 1.15009100 2.46756700 -0.28194700
C 1.78366500 3.67618000 0.14944900
C 1.06205900 4.89347700 0.15336600
C -0.25633800 4.89827400 -0.23341100
H -1.95189100 3.75337300 -0.86304200
C 3.12791700 3.57313500 0.59047300
H 1.55877900 5.80715200 0.46980900
H -0.82507900 5.82335400 -0.23806600
C 3.76874300 2.36053700 0.59063200
C 3.08720700 1.21038500 0.08623800
H 3.63222700 4.46378400 0.95768900
H 4.77143700 2.27505300 0.99300600
C 3.76418700 -0.11644800 0.05016200
C 3.04754200 -1.36735500 0.11814700
C 5.14980200 -0.17137100 -0.05216900
C 3.78285700 -2.59491100 0.10110500
C 5.86574900 -1.38553500 -0.06306000
H 5.71211800 0.75093500 -0.15593500
C 3.03026500 -3.79950000 0.13586100
C 5.19431000 -2.58456700 0.01934500
C 0.99388500 -2.50381600 0.16856300
H 6.94811100 -1.36487500 -0.14766500
C 1.66183800 -3.76962900 0.15090900
H 5.73203600 -3.52935400 0.00545600
H 1.10682200 -4.69899700 0.12016600
C -0.48283700 -2.39078700 0.08626400
C -1.14960400 -1.14765800 -0.25642300
C -1.29149300 -3.51352600 0.23979400
C -2.57944600 -1.17314200 -0.50854100
C -2.68786200 -3.53039900 0.02939400
H -0.82769400 -4.44892700 0.53590200
C -3.23823700 0.01560600 -0.75322100
C -3.32614600 -2.38435500 -0.36734500
C -1.00676000 1.20149100 -1.03348000
H -3.24073200 -4.45116700 0.18130200
C -2.52106200 1.32208200 -0.79037100
H -4.30879200 -0.01261800 -0.96325600
H -4.40382500 -2.33403100 -0.52287000
H -2.98046000 1.96894600 -1.54373500
N -0.48572500 0.00178500 -0.36509900
N 1.83946300 1.29980100 -0.34505700
N 1.69376300 -1.37615000 0.18581300
H 3.55874600 -4.74967300 0.11966600
H 0.53871300 -0.03925700 -0.23839600
H -2.70914700 1.79121200 0.18397500
H -0.83912900 1.05040400 -2.11434200
O -4.29463400 0.29900300 1.45586900
C -4.89303800 1.54523500 1.77143800
H -5.41082400 1.50595900 2.74188800
H -4.10262900 2.30452800 1.84563500
H -5.62050400 1.84718300 1.00601000
Cl -6.44804700 -0.86649900 -0.27332800
H -5.03462300 -0.25028300 1.05550100

Supplementary Table 16 Cartesian coordinates and energies of **INT4**.

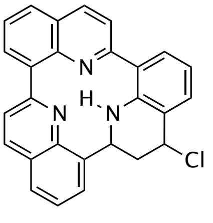

INT4

B3LYP-D3/6-31G(d)

Zero-point correction=	0.384938 (Hartree/Particle)
Thermal correction to Energy=	0.406397
Thermal correction to Enthalpy=	0.407341
Thermal correction to Gibbs Free Energy=	0.335190
Sum of electronic and zero-point Energies=	-1663.879349
Sum of electronic and thermal Energies=	-1663.857891
Sum of electronic and thermal Enthalpies=	-1663.856946
Sum of electronic and thermal Free Energies=	-1663.929097

C 1.63394100 3.60326700 0.10286900
C 0.91604300 2.43631400 0.29669600
C -0.50856400 2.49995500 0.09996000
C -1.11478600 3.71624700 -0.35111900
C -0.32765300 4.87941700 -0.52471800
C 1.02420700 4.82006500 -0.28725200
H 2.70924000 3.60871800 0.23656400
C -2.50354600 3.67435700 -0.63954900
H -0.80187100 5.80061800 -0.85459600
H 1.64486200 5.70172200 -0.41976200
C -3.20705800 2.50545300 -0.49955300
C -2.53804300 1.34571000 0.00019600
H -2.99271300 4.57334800 -1.00773100
H -4.25050000 2.45600000 -0.79002400
C -3.26860700 0.05710900 0.15324000
C -2.61981900 -1.22415700 0.00987200
C -4.63147600 0.06969300 0.42878500
C -3.40034300 -2.41630200 0.13894300
C -5.39140900 -1.11063200 0.55158000
H -5.13258500 1.02032700 0.58415600
C -2.70720300 -3.65290500 0.02230000
C -4.78637000 -2.33989000 0.40332100
C -0.63439000 -2.44343200 -0.29807500
H -6.45234800 -1.04198500 0.77362400
C -1.35370800 -3.67950100 -0.17358900
H -5.35857000 -3.25949000 0.50209700
H -0.83308600 -4.62898100 -0.19343600
C 0.84033800 -2.41381100 -0.39935200
C 1.61357500 -1.24333800 -0.05763600
C 1.53806000 -3.58816800 -0.74231700
C 3.02690200 -1.38073100 0.08976000
C 2.91871300 -3.67896100 -0.68247700
H 0.97717800 -4.45425700 -1.07828000
C 3.76511800 -0.20260500 0.68604900
C 3.65097600 -2.57837700 -0.22539000
C 1.62753900 1.13569200 0.72533200
H 3.42477400 -4.59603600 -0.96681400
C 3.11868200 1.13924200 0.35332100
H 3.83585400 -0.32028700 1.77235100
H 4.72927000 -2.65008300 -0.12795200
H 3.65594700 1.92317500 0.89122300
N 1.00653700 -0.04120600 0.13628100
N -1.25353000 1.38578300 0.31225000
N -1.28979000 -1.28781300 -0.23869300
H -3.26683000 -4.57996900 0.12552900
H -0.01697100 -0.04419600 0.10077300
H 3.21905800 1.32923100 -0.72030500
H 1.54494100 1.07824200 1.82660200
Cl 5.53147700 -0.11127700 0.16411300

Supplementary Table 17 Cartesian coordinates and energies of **TS4**.

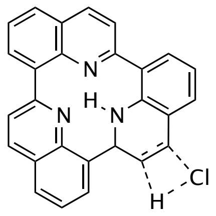

TS4

B3LYP-D3/6-31G(d)

Zero-point correction=	0.381062 (Hartree/Particle)
Thermal correction to Energy=	0.402705
Thermal correction to Enthalpy=	0.403650
Thermal correction to Gibbs Free Energy=	0.330334
Sum of electronic and zero-point Energies=	-1663.837602
Sum of electronic and thermal Energies=	-1663.815959
Sum of electronic and thermal Enthalpies=	-1663.815014
Sum of electronic and thermal Free Energies=	-1663.888330

C 2.67224500 2.67511400 0.41570000
C 1.57947500 1.85252200 0.60833500
C 0.28057500 2.36980700 0.27640100
C 0.15445900 3.68554300 -0.27099800
C 1.30520200 4.49826600 -0.42019200
C 2.53781000 3.99650200 -0.07910200
H 3.67462800 2.31086800 0.60812600
C -1.13981200 4.08607900 -0.68785000
H 1.19987100 5.50053300 -0.82797600
H 3.43175800 4.59827200 -0.21165500
C -2.20052300 3.21824900 -0.59441000
C -1.99473100 1.92938400 -0.01927300
H -1.27216500 5.07368200 -1.12344900
H -3.17266900 3.49614500 -0.98617300
C -3.11140400 0.94319200 0.04586600
C -2.89774400 -0.47790600 -0.07977600
C -4.41789100 1.38714900 0.20190300
C -4.02063900 -1.36408200 -0.05859400
C -5.52112000 0.50725400 0.22175200
H -4.60399900 2.44817900 0.33752200
C -3.74750000 -2.75537600 -0.15821100
C -5.33054700 -0.84976700 0.08760100
C -1.38758500 -2.26534800 -0.25774300
H -6.52081900 0.91100400 0.35191400
C -2.45823000 -3.21311200 -0.23995700
H -6.17245900 -1.53739500 0.10576400
H -2.26252000 -4.27889900 -0.25389400
C 0.03708900 -2.68315900 -0.24093300
C 1.09132200 -1.79239600 0.22511000
C 0.40964200 -3.97409300 -0.58555300
C 2.42229800 -2.33568100 0.43704800
C 1.72665600 -4.48153400 -0.45812400
H -0.34714400 -4.64558200 -0.97946600
C 3.39082500 -1.49772300 0.95491000
C 2.70762600 -3.68308200 0.06683700
C 1.73883700 0.43242100 1.17847500
H 1.93863600 -5.49955300 -0.76655500
C 3.18918000 -0.06967600 1.17972100

H 4.40221100 -1.86970500 1.08783900
H 3.72383800 -4.04533800 0.19281300
H 3.74918000 0.29298000 2.04712400
N 0.85578600 -0.51572400 0.47777200
N -0.80432100 1.56601100 0.43131300
N -1.63990000 -0.96441800 -0.21024800
H -4.57868100 -3.45623200 -0.13697600
H -0.11013900 -0.18076400 0.32936900
H 3.80234800 0.30222700 0.29063100
H 1.36329400 0.46905800 2.21471400
Cl 5.34004600 -0.07775600 -1.03912800

Supplementary Table 18 Cartesian coordinates and energies of **INT5**.

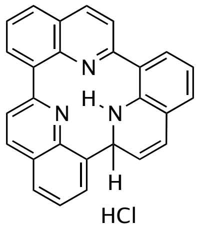

DQ–Im•HCl
INT5

B3LYP-D3/6-31G(d)
Zero-point correction= 0.347736 (Hartree/Particle)
Thermal correction to Energy= 0.368768
Thermal correction to Enthalpy= 0.369712
Thermal correction to Gibbs Free Energy= 0.297481
Sum of electronic and zero-point Energies= -1586.482107
Sum of electronic and thermal Energies= -1586.461076
Sum of electronic and thermal Enthalpies= -1586.460131
Sum of electronic and thermal Free Energies= -1586.532363
C -0.03740500 4.20429500 0.36282700
C 0.05527200 2.81931300 0.13698200
C -1.17443400 2.10753100 0.00633300
C -2.40620400 2.77325800 0.06361400
C -2.44376900 4.14927000 0.26400000
C -1.25909200 4.86701000 0.42515500
H 0.86737200 4.77993300 0.52067500
H -3.40707100 4.64773900 0.31173000
H -1.28170500 5.93749500 0.60705800
C -2.15619000 -0.10756200 -0.33117700
C -2.04830900 -1.54950100 -0.26911700
C -0.80889300 -2.25076600 -0.07398100
C -3.24164000 -2.27181300 -0.29005200
C -0.82173300 -3.65366400 0.16471000
C -3.23708400 -3.67280300 -0.10947000
H -4.17839100 -1.72341600 -0.40130700
C 0.42958200 -4.24848100 0.46539400
C -2.05846500 -4.35063900 0.12895400
C 1.50628100 -2.09808100 0.16223000
H -4.17999800 -4.21016800 -0.12973000
C 1.57477200 -3.48629900 0.49450100
H -2.06356400 -5.42384000 0.30502300
H 2.51342800 -3.94578800 0.77995500
C 2.72049400 -1.23863600 0.06680500
C 2.67104400 0.20871300 0.02993500
C 3.96762300 -1.84020400 -0.05844800
C 3.88793700 0.93853700 -0.16317100
C 5.16425800 -1.10887100 -0.20475800
H 4.03874300 -2.92182400 -0.08094800
C 3.78890700 2.34820400 -0.29025200
C 5.12827500 0.26385700 -0.26352500
C 1.40620800 2.19017100 0.03270700
H 6.10597600 -1.64172400 -0.29459100
C 2.57193700 2.97371700 -0.22467900
H 4.69387400 2.92357000 -0.46998900
H 6.03721500 0.84362000 -0.40299400
H 2.50438600 4.04183800 -0.38748900
N 1.49455000 0.87289100 0.16201600
N -1.14075700 0.69560300 -0.13722000
N 0.33576400 -1.53950400 -0.10614900
H 0.47249500 -5.31039200 0.69510600
H -3.34573200 2.22363700 -0.02158700
H -3.17609800 0.29771700 -0.46133100
H -0.19465700 0.26871800 -0.01950800
Cl -5.25349400 0.65944800 -0.18433600

Supplementary Table 19 Cartesian coordinates and energies of **complex I**.

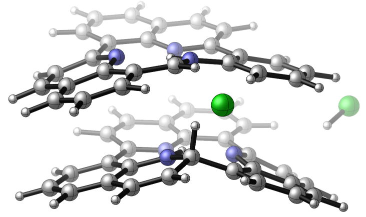

complex I

B3LYP-D3/6-31G(d)
Zero-point correction= 0.728480 (Hartree/Particle)
Thermal correction to Energy= 0.773679
Thermal correction to Enthalpy= 0.774623
Thermal correction to Gibbs Free Energy= 0.652944
Sum of electronic and zero-point Energies= -3250.408771
Sum of electronic and thermal Energies= -3250.363572
Sum of electronic and thermal Enthalpies= -3250.362628
Sum of electronic and thermal Free Energies= -3250.484307
C 3.24274000 -0.11446200 -1.90819300
C 1.85720500 -0.34110700 -1.96701800
C 1.02722700 0.80123600 -2.16606600
C 1.58318500 2.07979100 -2.31431000
C 2.96103300 2.25241200 -2.26033000
C 3.79505400 1.15460200 -2.05324800
H 3.91246400 -0.94219900 -1.70805100
H 3.37060800 3.25338400 -2.35279800
H 4.87157500 1.27990700 -1.99745600
C -1.28079300 1.59030600 -2.27088800
C -2.65896200 1.43074000 -1.87789900
C -3.20234700 0.18851800 -1.39371900
C -3.43658400 2.58796400 -1.80109300
C -4.48383400 0.17319300 -0.77724300

C -4.72973300 2.54841100 -1.23812900
H -2.99948300 3.53378100 -2.11916200
C -4.90251700 -1.06196100 -0.22647300
C -5.23489800 1.37684000 -0.71316600
C -2.84305400 -2.07632700 -0.99507300
H -5.30624300 3.46607800 -1.17793000
C -4.09027300 -2.17063100 -0.31404300
H -6.20978400 1.36081600 -0.23262900
H -4.39114300 -3.09504000 0.16490600
C -1.93459500 -3.24481400 -1.14952500
C -0.51737100 -3.11132200 -1.40551900
C -2.45308600 -4.52959200 -1.04948000
C 0.27913800 -4.29270700 -1.53686000
C -1.65441700 -5.68708600 -1.15410300
H -3.51997100 -4.66261800 -0.90471100
C 1.65530300 -4.11941800 -1.83522600
C -0.30423800 -5.57379400 -1.39256300
C 1.33990300 -1.73179200 -1.81083700
H -2.11847900 -6.66467000 -1.06234900
C 2.18540600 -2.86675400 -1.99368500
H 2.28179500 -4.99991200 -1.95377700
H 0.32381300 -6.45588800 -1.49073300
H 3.22680900 -2.75085400 -2.26512900
N 0.05622200 -1.88799800 -1.51774900
N -0.37785700 0.64517000 -2.13140200
N -2.45218100 -0.92126300 -1.51705600
H -5.85424100 -1.11654400 0.29415600
H 0.95065700 2.95959000 -2.42516000
H -0.98307100 2.58928300 -2.60598700
H -0.70501500 -0.29785400 -1.82285300
C 1.22521900 4.50229700 0.68126500
C 0.91839600 3.16433600 0.86399400
C 2.00459500 2.27369700 1.16508400
C 3.31092000 2.80582600 1.42936100
C 3.55855100 4.18878300 1.26869100
C 2.53184700 5.01113500 0.86796900
H 0.46069200 5.18726500 0.33874000
C 4.30071200 1.89006700 1.86666700
H 4.55665000 4.57846200 1.45429900
H 2.70742400 6.07069000 0.70634800
C 4.01558800 0.54752900 1.97140200
C 2.72787000 0.07930000 1.56021100
H 5.28149900 2.26832100 2.14754500
H 4.73762500 -0.13727800 2.40390500
C 2.41670400 -1.37570900 1.57033400
C 1.07871800 -1.87300200 1.77555800
C 3.43585400 -2.30716200 1.40144600
C 0.85121200 -3.28500400 1.81746400
C 3.20166400 -3.69659900 1.43854200
H 4.44740300 -1.96650600 1.20475000
C -0.49270800 -3.71096300 2.00803300
C 1.92842900 -4.18273000 1.64692800
C -1.19368300 -1.39779600 2.10645700
H 4.03363100 -4.37900400 1.29037500
C -1.50321300 -2.79699500 2.14281900
H 1.73475000 -5.25269000 1.66823700
H -2.52906600 -3.13364900 2.23476800
C -2.21839500 -0.34661800 2.23330700
C -1.99184800 1.00264600 1.78387900
C -3.46676100 -0.64813300 2.81287900
C -3.03211700 1.96968500 1.95422900
C -4.47418700 0.29476200 2.96267100
H -3.63906400 -1.65276600 3.18623900
C -2.75572400 3.32760700 1.52082200
C -4.24463500 1.60498000 2.53046300
C -0.52969100 2.68283000 0.60660400
H -5.41593100 0.02308100 3.42986900
C -1.60422900 3.68520800 0.93087200
H -3.53306500 4.07236700 1.67853700
H -5.01247700 2.36593400 2.65324700
H -1.47144000 4.68655600 0.54395000
N -0.83028900 1.38766500 1.20107200
N 1.79413000 0.93109000 1.17312500
N 0.06165900 -1.00084300 1.93689300
H -0.71021500 -4.77628300 2.00792700
H -0.05671100 0.71738400 1.22433000
H -0.56668600 2.59460300 -0.49691600
Cl -0.72839400 4.85797700 -2.19957400
Cl 6.47535100 -0.44810000 -0.28006700
H 5.47318200 0.19825100 0.24364500

Supplementary Table 20 Cartesian coordinates and energies of **TS**.

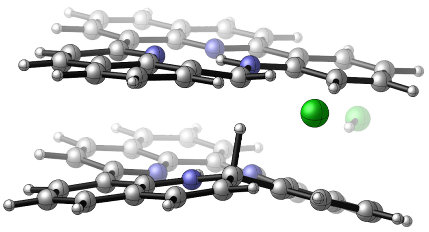

TS

B3LYP-D3/6-31G(d)

Zero-point correction= 0.723955 (Hartree/Particle)
Thermal correction to Energy= 0.768796
Thermal correction to Enthalpy= 0.769740
Thermal correction to Gibbs Free Energy= 0.648962
Sum of electronic and zero-point Energies= -3250.402641
Sum of electronic and thermal Energies= -3250.357799
Sum of electronic and thermal Enthalpies= -3250.356855
Sum of electronic and thermal Free Energies= -3250.477633

C 3.31089100 2.15926000 -1.86945500
C 2.19673500 1.29347600 -1.82510600
C 0.90252700 1.90855300 -1.80554000
C 0.79092100 3.31519300 -1.78328000
C 1.91969800 4.11778900 -1.79011300
C 3.19326600 3.54130300 -1.84337800
H 4.30696600 1.74344600 -1.94472900
H 1.79954100 5.19647600 -1.75827900
H 4.08473300 4.16098100 -1.87384900
C -1.52228200 1.56712400 -1.69990700
C -2.66601700 0.64928900 -1.79916800

C -2.55351300 -0.77645500 -1.66909100
C -3.93584100 1.21900100 -1.87433200
C -3.73273600 -1.56750500 -1.54598200
C -5.09632100 0.41906900 -1.80080600
H -4.01519200 2.30154400 -1.96111300
C -3.53473800 -2.95642300 -1.34588700
C -5.00373500 -0.94351500 -1.60857100
C -1.13576600 -2.62965500 -1.51610000
H -6.06989200 0.89614000 -1.86408400
C -2.26722900 -3.48522800 -1.32081500
H -5.89718000 -1.55516100 -1.51000600
H -2.14788900 -4.54554800 -1.13960800
C 0.25939500 -3.15671000 -1.57848200
C 1.44438000 -2.32143100 -1.66213100
C 0.45806800 -4.53530500 -1.57449800
C 2.73093500 -2.95094900 -1.71999200
C 1.72805300 -5.13869500 -1.62176300
H -0.39691700 -5.19908600 -1.54507400
C 3.86625800 -2.10434100 -1.80364500
C 2.85726900 -4.35648100 -1.68989400
C 2.41721600 -0.17702600 -1.78937200
H 1.80374700 -6.22217000 -1.61197900
C 3.72990000 -0.74458700 -1.84228100
H 4.85459000 -2.55673300 -1.83610200
H 3.84878300 -4.80101500 -1.72870800
H 4.61675700 -0.12823400 -1.89969300
N 1.35414300 -0.96993600 -1.69378200
N -0.24082100 1.11851600 -1.79937900
N -1.32035300 -1.32706500 -1.66064900
H -4.39975800 -3.59947900 -1.20376300
H -0.18708300 3.78828400 -1.73649900
H -1.74092000 2.60014300 -1.99235600
H -0.08343600 0.09744800 -1.72801700
C -0.86594100 4.22244200 1.23721600
C -0.67155500 2.84934900 1.27260800
C 0.65710600 2.36413200 1.53044100
C 1.70305500 3.29414500 1.83155300
C 1.45067500 4.68412700 1.78412400
C 0.18745800 5.13189100 1.47468500
H -1.82758300 4.63338600 0.96265400
C 2.95881300 2.74642100 2.19359000
H 2.26047900 5.37952800 1.99121100
H -0.02175400 6.19539800 1.41355200
C 3.14668400 1.38549200 2.22019700
C 2.08269500 0.52596600 1.80572900
H 3.76351700 3.41835000 2.48429400
H 4.08292100 0.97517700 2.57830900
C 2.27685900 -0.94886900 1.75263700
C 1.18571200 -1.89166200 1.81558600
C 3.56494300 -1.46347000 1.64314700
C 1.46981300 -3.29446800 1.79014800
C 3.83442500 -2.84606500 1.63263700
H 4.40469500 -0.78668400 1.52859900
C 0.35899300 -4.17866300 1.81555700
C 2.80341000 -3.75462000 1.71000000
C -1.12638400 -2.27935200 1.90050100
H 4.86226600 -3.18483600 1.54215700
C -0.92096300 -3.69455700 1.85473400
H 2.99459400 -4.82414500 1.68450200
H -1.75736300 -4.38115300 1.81844800
C -2.48002500 -1.67728300 1.92008400
C -2.71861900 -0.28912600 1.64263500
C -3.60038900 -2.48075600 2.18282700
C -4.05347500 0.20739200 1.62327000
C -4.90646800 -1.99331800 2.16153200
H -3.45261800 -3.52515900 2.43310400
C -4.24046300 1.60537300 1.33604200
C -5.12906300 -0.65319200 1.87281600
C -1.83873700 1.92103300 0.97363700
H -5.73757000 -2.65875100 2.37360100
C -3.20901200 2.43853700 1.06540700
H -5.25539300 1.99484800 1.33555100
H -6.13832900 -0.25042100 1.84646100
H -3.39069000 3.46036500 0.75709600
N -1.70063900 0.59148400 1.40345300
N 0.90495500 1.03256100 1.47797700
N -0.09128700 -1.45078500 1.89699600
H 0.54211900 -5.24948000 1.77268000
H -0.73691800 0.22387500 1.47315200
H -1.70177700 1.87878700 -0.32986400
Cl -2.76947500 4.64905600 -1.50720200
Cl 6.05813300 1.47080300 0.32772100
H 4.82210000 1.86297900 0.36474400

Supplementary Table 21 Cartesian coordinates and energies of **complex II**.

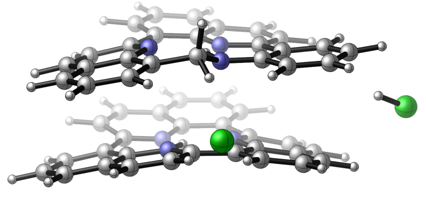

complex II

B3LYP-D3/6-31G(d)

Zero-point correction=	0.729214 (Hartree/Particle)
Thermal correction to Energy=	0.774586
Thermal correction to Enthalpy=	0.775530
Thermal correction to Gibbs Free Energy=	0.652053
Sum of electronic and zero-point Energies=	-3250.438306
Sum of electronic and thermal Energies=	-3250.392934
Sum of electronic and thermal Enthalpies=	-3250.391990
Sum of electronic and thermal Free Energies=	-3250.515467

C 4.22992300 0.20424400 -1.64212100
C 2.82903900 0.35969300 -1.56880800
C 2.31722000 1.70679900 -1.43692200
C 3.24871900 2.77448400 -1.26694300
C 4.60938300 2.55710800 -1.30989700
C 5.12010400 1.26461000 -1.52602200
H 4.64133900 -0.77918900 -1.84022800
H 5.28572600 3.39856600 -1.18475500

H 6.18639900 1.10619800 -1.66311400
C 0.41935600 3.27274200 -1.74383600
C -1.08325300 3.37419900 -1.55015200
C -1.99564400 2.27488000 -1.65423000
C -1.59740600 4.62474600 -1.24805300
C -3.39408100 2.48669000 -1.43224000
C -2.98406400 4.83768100 -1.07364400
H -0.90499100 5.44695400 -1.08638300
C -4.21782700 1.33201800 -1.43991000
C -3.87467600 3.78872500 -1.16043400
C -2.27283000 -0.03817500 -1.90442100
H -3.33954700 5.83859000 -0.84374100
C -3.67650700 0.08858500 -1.64413200
H -4.94007400 3.93979800 -1.00120000
H -4.31010400 -0.78676600 -1.56880000
C -1.65133000 -1.37506300 -2.13973000
C -0.23055800 -1.63688700 -2.02818000
C -2.47092600 -2.44667100 -2.48910400
C 0.24220500 -2.97779400 -2.20934400
C -1.98906800 -3.75305400 -2.68699200
H -3.53050200 -2.27754500 -2.64295900
C 1.63745900 -3.19672700 -2.04337400
C -0.64746500 -4.02308500 -2.53311600
C 1.95844300 -0.82379700 -1.71094000
H -2.68414500 -4.54260700 -2.95800200
C 2.48683100 -2.15700000 -1.79173500
H 2.01828900 -4.21280800 -2.11884400
H -0.25883000 -5.02966800 -2.66408100
H 3.54108000 -2.35457600 -1.64588400
N 0.64246400 -0.63380500 -1.77813400
N 0.98633000 1.96157800 -1.45392100
N -1.50345900 1.03616900 -1.91683900
H -5.28017100 1.44090800 -1.23516700
H 2.87259000 3.77210700 -1.06424500
H 0.65663700 3.55455600 -2.78478700
H 0.38679400 1.16028500 -1.67312600
C 2.16650800 2.21235400 1.78725300
C 1.23379500 1.18424200 1.65957100
C 1.71976700 -0.17457000 1.68875400
C 3.09973600 -0.42435000 1.95745000
C 3.99798700 0.65991500 2.12222500
C 3.53470500 1.94907500 2.00370200
H 1.87729900 3.25885500 1.69617800
C 3.49465300 -1.77944200 2.06034500
H 5.04308400 0.45446700 2.33333300
H 4.21179500 2.79290900 2.08399600
C 2.58156600 -2.78851600 1.86944800
C 1.23730300 -2.46092900 1.52967000
H 4.53067300 -2.00974200 2.28979400
H 2.89280700 -3.81858400 1.99200700
C 0.23731700 -3.52055200 1.21071400
C -1.18572000 -3.28241300 1.15937800
C 0.67685000 -4.79944600 0.88989800
C -2.06583700 -4.34438700 0.77377400
C -0.19837200 -5.84928300 0.54339400
H 1.73991600 -5.00772700 0.86344700
C -3.44588800 -4.03816800 0.66109400
C -1.55403200 -5.62949400 0.48044600
C -2.97433700 -1.78402700 1.35354300
H 0.21317400 -6.82586700 0.30597700
C -3.90617300 -2.77384900 0.92252300
H -2.23933100 -6.42162200 0.18979200
H -4.95332200 -2.53586600 0.77907700
C -3.39290500 -0.39024500 1.65794100
C -2.47359800 0.71287300 1.68516000
C -4.73281400 -0.09956700 1.89696400
C -2.95107700 2.03952700 1.85911200
C -5.20706300 1.20981000 2.09601600
H -5.45043200 -0.91138200 1.93818400
C -2.00444900 3.09778900 1.80429000
C -4.33074000 2.27089600 2.05690800
C -0.19579000 1.52356900 1.58784400
H -6.26571200 1.37357000 2.27236500
C -0.66421500 2.86329800 1.66147600
H -2.35831500 4.12254500 1.85962700
H -4.67636400 3.29310300 2.17846900
H 0.01939800 3.71052100 1.57537700
N -1.11760300 0.53793100 1.55839900
N 0.85788500 -1.19177000 1.46421200
N -1.68625900 -2.06142100 1.46416100
H -4.13104200 -4.81738400 0.33622000
H -0.75764500 -0.44275000 1.47884100
H 0.86703900 4.03223400 -1.09240800
Cl 1.36828900 5.45332900 0.79927300
Cl 6.73046800 -1.09313100 0.55306400
H 5.90075300 -0.20460600 0.07249700

Supplementary Table 22 Cartesian coordinates and energies of **DQ-Am**•HCl.

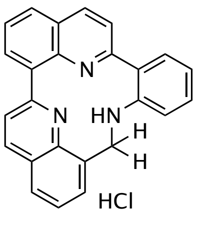

DQ–Am•HCl

B3LYP-D3/6-31G(d)
Zero-point correction= 0.365928 (Hartree/Particle)
Thermal correction to Energy= 0.388468
Thermal correction to Enthalpy= 0.389412
Thermal correction to Gibbs Free Energy= 0.312388
Sum of electronic and zero-point Energies= -1587.671797
Sum of electronic and thermal Energies= -1587.649258
Sum of electronic and thermal Enthalpies= -1587.648313
Sum of electronic and thermal Free Energies= -1587.725338
C -3.81652700 0.76106900 -0.30023900
C -2.43993500 0.53017100 -0.46853600
C -2.01672500 -0.79632100 -0.86966000
C -3.02443700 -1.75005900 -1.17514100
C -4.36793400 -1.46691700 -1.01483900

C -4.78352700 -0.21054100 -0.54344200
H -4.14377300 1.73039500 0.06234200
H -5.10421500 -2.23524700 -1.23618900
H -5.83613400 0.01209100 -0.39985200
C -0.04756400 -2.22945600 -1.57166100
C 1.21907800 -2.66805000 -0.84198200
C 2.13662200 -1.71721800 -0.28639500
C 1.50963300 -4.01036900 -0.70081000
C 3.27936700 -2.16988600 0.44337300
C 2.67411200 -4.45992700 -0.03066200
H 0.82009000 -4.74947100 -1.10251200
C 4.07085200 -1.17196400 1.07016500
C 3.54206300 -3.55871300 0.54251100
C 2.57461800 0.51965300 0.20374500
H 2.86139300 -5.52691800 0.05056700
C 3.71432700 0.15127400 0.98393700
H 4.41993600 -3.89678700 1.08784700
H 4.27467300 0.90610500 1.52474000
C 2.13989200 1.94090500 0.10571500
C 0.75229600 2.31440500 -0.03456300
C 3.08517000 2.95749500 0.16422400
C 0.40758700 3.70096500 -0.10435400
C 2.73460100 4.32181100 0.10027200
H 4.13772300 2.69913600 0.23358500
C -0.96908500 4.01293100 -0.27997600
C 1.41370300 4.69154100 -0.03018100
C -1.48713900 1.64980900 -0.29146200
H 3.51518300 5.07579200 0.14321800
C -1.90329400 3.01858200 -0.39072000
H -1.26489600 5.05696100 -0.35530800
H 1.13077900 5.73996200 -0.08935900
H -2.93875200 3.27038700 -0.58662600
N -0.20551200 1.35833800 -0.09552100
N -0.69712600 -1.09880700 -0.93527500
N 1.86178500 -0.39608400 -0.42925200
H 4.93901600 -1.47298500 1.65212700
H -2.73191400 -2.73693300 -1.51567900
H 0.21188100 -1.97536400 -2.61385000
H -0.06003700 -0.34229200 -0.66968000
H -0.72349500 -3.08709400 -1.62057000
Cl -4.02016700 -1.34113200 2.70971400
H -4.26517900 -0.87978700 1.51448800

Supplementary Table 23 Cartesian coordinates and energies of **TQ**•HCl.

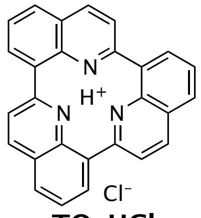

TQ•HCl

B3LYP-D3/6-31G(d)

Zero-point correction=	0.360304 (Hartree/Particle)
Thermal correction to Energy=	0.382036
Thermal correction to Enthalpy=	0.382980
Thermal correction to Gibbs Free Energy=	0.309086
Sum of electronic and zero-point Energies=	-1662.700758
Sum of electronic and thermal Energies=	-1662.679026
Sum of electronic and thermal Enthalpies=	-1662.678081
Sum of electronic and thermal Free Energies=	-1662.751975

C -3.32550300 -1.70338900 0.09469000
C -2.04677800 -1.14411800 0.09364400
C -0.91595500 -2.03416200 -0.02723100
C -1.13720000 -3.43087100 -0.22919400
C -2.45837200 -3.94694000 -0.22716400
C -3.52230200 -3.09502800 -0.04594800
H -4.23225500 -1.09325900 0.18536300
C 0.00867700 -4.23001900 -0.45256400
H -2.60955200 -5.01387500 -0.37375300
H -4.54225900 -3.46653400 -0.03369300
C 1.26567600 -3.67427700 -0.43362400
C 1.41122800 -2.28860300 -0.13288000
H -0.11898400 -5.29022900 -0.65722300
H 2.12587500 -4.29223900 -0.65922500
C 2.75840800 -1.66308700 0.01030000
C 2.98282400 -0.23491400 0.00509300
C 3.86891900 -2.47832800 0.20173100
C 4.31269500 0.26719900 0.18524300
C 5.17760000 -1.97585300 0.35388500
H 3.73890300 -3.55217800 0.26798100
C 4.47642400 1.67541900 0.23595400
C 5.40324100 -0.61998400 0.34432100
C 2.10508500 1.93784100 -0.07804300
H 5.99950200 -2.67115100 0.49526900
C 3.39556100 2.51058900 0.12868300
H 6.40336700 -0.21522500 0.47624800
H 3.53092000 3.58044200 0.22632000
C 0.87841400 2.77665800 -0.17725000
C -0.45212900 2.24327400 -0.06382000
C 0.98579700 4.15375100 -0.35946800
C -1.57878400 3.11306200 -0.06482400
C -0.12594400 5.01516000 -0.38661000
H 1.96556600 4.59531200 -0.49920000
C -2.86622700 2.53330100 0.09403900
C -1.39408000 4.50569900 -0.22367700
C -1.91597500 0.32387400 0.12982400
H 0.02797700 6.07985900 -0.53114200
C -3.04756300 1.17941600 0.19525800
H -3.73960600 3.18024700 0.12372300
H -2.26649800 5.15263900 -0.22575800
H -4.07274200 0.79142200 0.29554300
N -0.69343600 0.89494200 0.03962800
N 0.33706500 -1.53054200 0.04794500
N 1.94622100 0.62526000 -0.14907500
H 5.47259300 2.08286100 0.38999800
H 0.13482300 0.25444700 0.00945900
Cl -6.14170600 0.09740100 0.34814100

Supplementary Table 24 Cartesian coordinates and energies of T**Q**.

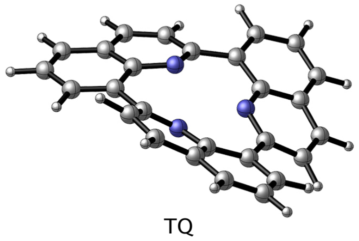

TQ

B3LYP-D3/6-31G(d,p)

Zero-point correction=	0.345535 (Hartree/Particle)
Thermal correction to Energy=	0.365028
Thermal correction to Enthalpy=	0.365972
Thermal correction to Gibbs Free Energy=	0.298621
Sum of electronic and zero-point Energies=	-1201.903818
Sum of electronic and thermal Energies=	-1201.884325
Sum of electronic and thermal Enthalpies=	-1201.883381
Sum of electronic and thermal Free Energies=	-1201.950732

C -3.70117500 -1.88168400 -0.33803400
C -2.49098900 -1.27169700 -0.04452500
C -2.46411800 0.16673400 0.07283900
C -3.66273500 0.90701900 -0.18175200
C -4.87540200 0.22954700 -0.45999900
C -4.89212600 -1.14455700 -0.52579000
H -3.73505900 -2.95875300 -0.46984600
C -3.55104200 2.32173500 -0.18268500
H -5.77773600 0.80861200 -0.63957000
H -5.81356800 -1.67165100 -0.75399900
C -2.33101200 2.91583100 0.01224200
C -1.19328600 2.09730700 0.31173800
H -4.43361600 2.92269900 -0.38737900
H -2.23408800 3.99083700 -0.08298600
C 0.16363900 2.72225000 0.41859900
C 1.37185500 1.99647700 0.08540000
C 0.29222000 4.07725700 0.69160200
C 2.58409800 2.72439800 -0.14474800
C 1.51923400 4.76787600 0.56933100
H -0.57879400 4.64409900 1.00333500
C 3.68563100 1.98418000 -0.65171000
C 2.64229400 4.11520100 0.11645800
C 2.39687600 -0.03775600 -0.40543500
H 1.55647400 5.82745600 0.80333000
C 3.59295600 0.62632800 -0.82538900
H 3.57714400 4.64834200 -0.03680000
H 4.42056200 0.07525300 -1.25760800
C 2.31466400 -1.52520200 -0.30085000
C 1.05506700 -2.21525300 -0.11985600
C 3.47913800 -2.27581900 -0.21277500
C 1.06997700 -3.59192500 0.27918700
C 3.47414100 -3.66068700 0.06463900
H 4.44158300 -1.78357700 -0.30010200
C -0.17160800 -4.17045500 0.65276300
C 2.29149800 -4.30741900 0.33574900
C -1.25242400 -2.08972800 0.09689400
H 4.41758100 -4.19563300 0.11546100
C -1.31628800 -3.41619600 0.62497200
H -0.19145500 -5.20121100 0.99776800
H 2.27945100 -5.35706400 0.61819500
H -2.25520600 -3.82049900 0.98728900
N -0.11178900 -1.55205500 -0.29582800
N -1.30224500 0.78387900 0.38978700
N 1.34832300 0.65043400 0.00302800
H 4.60020600 2.51270000 -0.90919200

Supplementary Table 25 Cartesian coordinates and energies of **biphenyl**.

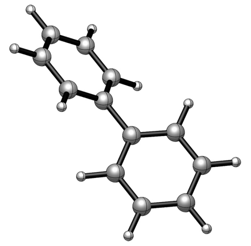

biphenyl

B3LYP-D3/6-31G(d,p)

Zero-point correction=	0.181891 (Hartree/Particle)
Thermal correction to Energy=	0.190734
Thermal correction to Enthalpy=	0.191678
Thermal correction to Gibbs Free Energy=	0.147410
Sum of electronic and zero-point Energies=	-463.155339
Sum of electronic and thermal Energies=	-463.146496
Sum of electronic and thermal Enthalpies=	-463.145552
Sum of electronic and thermal Free Energies=	-463.189820

C -1.46365200 -1.13549400 0.40503800
C -2.85756600 -1.13612300 0.40420900
C -0.74224000 0.00000000 0.00000000
C -3.56105500 0.00000000 -0.00000100
H -3.39507100 -2.02252200 0.72883400
C -1.46365100 1.13549400 -0.40503900
C -2.85756600 1.13612400 -0.40421100
H -4.64703500 0.00000100 -0.00000100
H -0.92492300 2.01416200 -0.74704700
H -3.39507000 2.02252300 -0.72883600
H -0.92492400 -2.01416200 0.74704700
C 0.74224000 0.00000000 0.00000000
C 1.46365200 -1.13549400 -0.40503800
C 1.46365100 1.13549400 0.40503900
C 2.85756600 -1.13612300 -0.40421000
H 0.92492400 -2.01416200 -0.74704700
C 2.85756600 1.13612400 0.40421100
H 0.92492300 2.01416200 0.74704700
C 3.56105500 0.00000000 0.00000100
H 3.39507100 -2.02252200 -0.72883500
H 3.39507000 2.02252300 0.72883600
H 4.64703500 0.00000100 0.00000100

Supplementary Table 26 Cartesian coordinates and energies of **2,8-diphenylquinoline**.

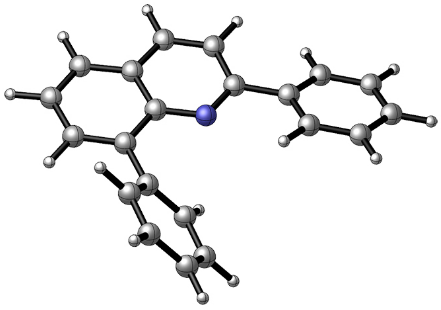

2,8-diphenylquinoline

B3LYP-D3/6-31G(d,p)

Zero-point correction= 0.297680 (Hartree/Particle)
Thermal correction to Energy= 0.313763
Thermal correction to Enthalpy= 0.314707
Thermal correction to Gibbs Free Energy= 0.252765
Sum of electronic and zero-point Energies= -863.804364
Sum of electronic and thermal Energies= -863.788281
Sum of electronic and thermal Enthalpies= -863.787337
Sum of electronic and thermal Free Energies= -863.849279

C 3.26460300 1.33007700 0.09217700
C 2.12051200 0.54588300 0.07482400
C 0.84558300 1.20972700 0.02685700
C 0.79996400 2.64162700 -0.00121500
C 2.00054200 3.39200700 0.02082200
C 3.21233000 2.74149700 0.06528500
H 4.23222600 0.84107600 0.15034200
C -0.48308200 3.24246800 -0.06798800
H 1.94753600 4.47737600 0.00373100
H 4.13803600 3.30853600 0.08920900
C -1.61030300 2.46232700 -0.09599500
C -1.47431300 1.04250100 -0.05480700
H -0.55815100 4.32625300 -0.10800100
H -2.58875300 2.92084400 -0.17539400
C 3.34710100 -2.94750500 -0.72732600
C 2.56019200 -3.73467500 0.11366600
C 1.61261200 -3.12438900 0.93890700
C 2.23823200 -0.93525900 0.08431100
C 1.44994100 -1.74079100 0.92461000
H 0.99751700 -3.72841400 1.59989500
H 0.70976900 -1.27539100 1.56316100
N -0.28511700 0.46101800 -0.00615600
C -2.65455400 0.13741100 -0.08053400
C -3.95164700 0.59245400 0.20661800
C -2.47398900 -1.22337600 -0.38644800
C -5.03715200 -0.28226700 0.17884200
C -3.55918900 -2.09403100 -0.41622900
H -1.47060400 -1.57709500 -0.59500900
C -4.84664200 -1.62799700 -0.13649200
H -3.40099500 -3.14056900 -0.66053000
H -5.69279500 -2.30851300 -0.16040800
H -4.12155300 1.62905000 0.47879900
H -6.03159900 0.08830500 0.41004100
C 3.18699600 -1.56216100 -0.74029800
H 3.78634400 -0.95703600 -1.41431500
H 2.68116400 -4.81404600 0.12445200
H 4.08125300 -3.41066100 -1.38041200

Supplementary Table 27 Cartesian coordinates and energies of **TQ** •H+.

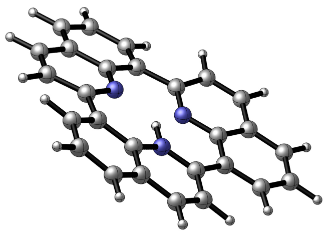

TQ•H+

B3LYP-D3/6-31G(d,p)

Zero-point correction= 0.359821 (Hartree/Particle)
Thermal correction to Energy= 0.379351
Thermal correction to Enthalpy= 0.380295
Thermal correction to Gibbs Free Energy= 0.312408
Sum of electronic and zero-point Energies= -1202.348866
Sum of electronic and thermal Energies= -1202.329336
Sum of electronic and thermal Enthalpies= -1202.328392
Sum of electronic and thermal Free Energies= -1202.396279

C 3.75330600 -1.89801100 -0.20035700
C 2.50517400 -1.27474400 -0.16939000
C 2.48432800 0.16117500 -0.03097800
C 3.71030100 0.87068400 0.14431300
C 4.94333700 0.18038700 0.11003800
C 4.95911300 -1.18454700 -0.07806800
H 3.83042700 -2.97225100 -0.30565100
C 3.61216200 2.26639800 0.37526300
H 5.86808500 0.73578100 0.23621800
H 5.89886500 -1.72450300 -0.11215200
C 2.38797800 2.88222800 0.38732500
C 1.21098400 2.11892900 0.11453600
H 4.51744000 2.83782500 0.55866000
H 2.32728200 3.93787600 0.61500700
C -0.12249300 2.77086400 0.01246000
C -1.36923100 2.04177600 0.02408800
C -0.19298900 4.15208600 -0.13950700
C -2.60113700 2.76237600 -0.09938900
C -1.41058500 4.85429400 -0.24514800
H 0.71817200 4.73319900 -0.20836100
C -3.80105500 2.00920600 -0.13019500
C -2.60326300 4.17288200 -0.22100600
C -2.50714900 -0.00352600 0.08634300
H -1.39302000 5.93292800 -0.35790000
C -3.76855500 0.64071700 -0.05636200
H -3.55024900 4.69667600 -0.31103400
H -4.68996000 0.07866500 -0.13105100
C -2.38397800 -1.48718100 0.14465300
C -1.13351900 -2.18006800 0.00583400
C -3.51671000 -2.27882100 0.31492000
C -1.10437800 -3.60406400 -0.03039800
C -3.48849700 -3.68624600 0.30390100
H -4.47594300 -1.80192500 0.47177800

C 0.15122500 -4.23545600 -0.21174500
C -2.29869400 -4.34763400 0.11453700
C 1.27596700 -2.09417600 -0.20332500
H -4.41217900 -4.23805900 0.43905600
C 1.30929700 -3.51020100 -0.30186400
H 0.18675800 -5.31930400 -0.26989600
H -2.25384200 -5.43155500 0.08539100
H 2.24748900 -4.02783100 -0.43246100
N 0.06657600 -1.51167000 -0.08486800
N 1.29714400 0.80597100 -0.06796500
N -1.37930900 0.68987400 0.13411700
H -4.74772700 2.53082100 -0.23736500
H 0.03188700 -0.45353900 -0.03211900

Supplementary Table 28 Cartesian coordinates and energies of **2,8-diphenylquinoline•**H^+.

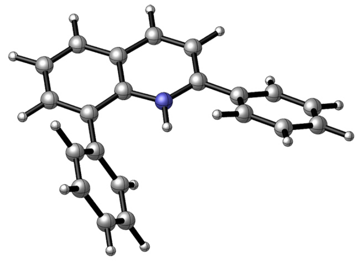

2,8-diphenylquinoline•H^+
B3LYP-D3/6-31G(d,p)

Zero-point correction=	0.311756 (Hartree/Particle)
Thermal correction to Energy=	0.327972
Thermal correction to Enthalpy=	0.328916
Thermal correction to Gibbs Free Energy=	0.266905
Sum of electronic and zero-point Energies=	-864.196005
Sum of electronic and thermal Energies=	-864.179789
Sum of electronic and thermal Enthalpies=	-864.178845
Sum of electronic and thermal Free Energies=	-864.240856

C 3.36261100 1.21846000 0.02176600
C 2.19468400 0.46821800 0.06859600
C 0.97107000 1.18641200 0.02508700
C 0.93500200 2.60843400 -0.02513000
C 2.15796700 3.32039000 -0.05119900
C 3.35011800 2.62922100 -0.03733400
H 4.31250900 0.69447000 0.04963600
C -0.33689900 3.23642400 -0.05932300
H 2.13836900 4.40470000 -0.08576300
H 4.29171300 3.16701200 -0.06088400
C -1.50111500 2.50197000 -0.06828800
C -1.44855300 1.09017100 -0.04836100
H -0.38310100 4.32084900 -0.09785400
H -2.46574400 2.98749100 -0.13555400
C 2.93232600 -3.13822100 -0.81377200
C 2.26436500 -3.82185200 0.20508700
C 1.57834300 -3.10791200 1.18883300
C 2.21622900 -1.01703600 0.12254000
C 1.55463000 -1.71291700 1.15168200
H 1.07672800 -3.63328300 1.99526900
H 1.06519800 -1.16093200 1.95066800
N -0.23237400 0.51598500 0.00622100
C -2.62943100 0.22282000 -0.10065800
C -3.83202800 0.63677500 0.50116200
C -2.57382100 -1.02947800 -0.74348700
C -4.94916200 -0.19207000 0.47065200
C -3.69716100 -1.84916300 -0.77539800
H -1.67363400 -1.34409600 -1.26488800
C -4.88455600 -1.43390600 -0.16641500
H -3.65177400 -2.80462400 -1.28716100
H -5.75964300 -2.07501800 -0.19346300
H -3.88270900 1.58626100 1.02391100
H -5.86879100 0.12791200 0.94889000
C 2.90803800 -1.74517400 -0.85800700
H 3.41474100 -1.21521300 -1.65888300
H 2.28555400 -4.90640200 0.23617600
H 3.46982500 -3.69073000 -1.57777700
H -0.17856600 -0.49969200 0.06593800

Supplementary Table 29 Cartesian coordinates and energies of **[12]CPP⊃TQ•**H^+.

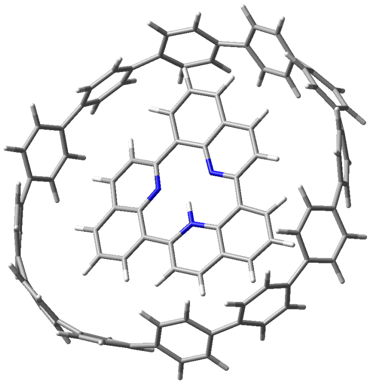

[12]CPP⊃TQ•H^+
B3LYP/6-31G(d,p)

Zero-point correction=	1.330966 (Hartree/Particle)
Thermal correction to Energy=	1.406032
Thermal correction to Enthalpy=	1.406976
Thermal correction to Gibbs Free Energy=	1.217265
Sum of electronic and zero-point Energies=	-3974.033903
Sum of electronic and thermal Energies=	-3973.958837
Sum of electronic and thermal Enthalpies=	-3973.957893
Sum of electronic and thermal Free Energies=	-3974.147604

C -2.81857600 -2.98370100 -0.59380600
C -1.86843200 -1.98992400 -0.36252700
C -2.34498600 -0.63919500 -0.19319300
C -3.73944700 -0.36210000 -0.30895500
C -4.65406800 -1.41314700 -0.54666400
C -4.19339300 -2.70536000 -0.67555600
H -2.51701900 -4.01309500 -0.74145700
C -4.13378500 0.99609800 -0.20269500
H -5.71335300 -1.19166900 -0.62850000
H -4.88423000 -3.51867700 -0.85692200
C -3.19762000 1.97372400 0.01469800
C -1.82523600 1.61147500 0.17463200
H -5.18219800 1.25565400 -0.31604300
H -3.51134100 3.00889800 0.04575000
C -0.78809500 2.63640600 0.46447400

C 0.62957400 2.40009600 0.32106400
C -1.18398200 3.89272900 0.90949800
C 1.54594400 3.46569000 0.59136900
C -0.27315800 4.93186700 1.18208000
H -2.23460300 4.10017300 1.07226000
C 2.92899600 3.19599200 0.44362500
C 1.07519900 4.73199700 1.01276700
C 2.39358900 0.93152200 -0.14787100
H -0.65126000 5.89257200 1.50927800
C 3.36327100 1.94500000 0.09082300
H 1.78725800 5.52922700 1.20139700
H 4.42571000 1.75181800 0.02168700
C 2.78404200 -0.46999300 -0.46395500
C 1.85092400 -1.56105000 -0.49032500
C 4.11727600 -0.78622400 -0.71071100
C 2.31139800 -2.89486100 -0.67880200
C 4.57184300 -2.09953600 -0.92715300
H 4.85653200 0.00453200 -0.73586200
C 1.35585600 -3.94187600 -0.63616200
C 3.68458700 -3.14873800 -0.89152000
C -0.43557600 -2.33961700 -0.38135000
H 5.62746700 -2.27322800 -1.10178500
C 0.01922700 -3.68047800 -0.48785700
H 1.69873700 -4.96852800 -0.72815200
H 4.01934300 -4.17105400 -1.02169900
H -0.68108800 -4.50260200 -0.45675600
N 0.49656800 -1.36764400 -0.34843400
N -1.45187900 0.34150400 0.06438600
N 1.09554100 1.18159300 -0.05234400
H 3.64749000 3.98717500 0.63137800
H 0.16880900 -0.37082700 -0.20172500
C -0.99125000 8.47348600 0.84792600
C -2.34444000 8.16448600 0.96524800
C -3.08942700 7.71203800 -0.13992000
C -2.46190300 7.79508800 -1.39590800
C -1.11747200 8.12715200 -1.51488800
C -0.31459400 8.36366400 -0.38253900
H -0.43972500 8.71768300 1.75034700
H -2.80990500 8.21841600 1.94481100
H -3.00423300 7.50404600 -2.28976600
H -0.67454100 8.12712200 -2.50489700
C 2.01615200 8.60363000 0.62351600
C 1.16837700 8.24633200 -0.44453500
C 1.76432000 7.54427800 -1.50919100
C 3.07981200 7.10530200 -1.44886000
C 3.87816600 7.33145300 -0.31380300
C 3.33348400 8.15032300 0.69287400
H 1.63613800 9.21311900 1.43748700
H 1.16622300 7.23902000 -2.35971300
H 3.45733900 6.48077500 -2.25197100
H 3.93672500 8.41507200 1.55667900
C 6.79257600 5.06745800 -1.15210700
C 6.95354900 4.35062500 0.04917500
C 6.30640100 4.86592100 1.18738400
C 5.41421100 5.93259700 1.09308700
C 5.11643100 6.52662900 -0.14695600
C 5.89424000 6.12471500 -1.24902100
H 7.34773700 4.76854300 -2.03588000
H 6.41739100 4.36367400 2.14334500
H 4.86087600 6.23347800 1.97795300
H 5.76266800 6.61972200 -2.20644200
C 8.11471400 2.43755800 1.25762200
C 7.58761800 3.00607300 0.08257100
C 7.50726500 2.17045900 -1.04757300
C 7.82160100 0.81697300 -0.97451200
C 8.22135400 0.22294400 0.23622100
C 8.41853300 1.08017600 1.33417600
H 8.26564300 3.05877800 2.13526700
H 7.11148200 2.56305500 -1.97862600
H 7.66909700 0.19465200 -1.85138900
H 8.79216300 0.67501700 2.26996500
C 7.29135200 -3.19268500 1.52474700
C 7.47964900 -4.01828800 0.40074000
C 8.19112100 -3.46503400 -0.68200600
C 8.54227700 -2.11623000 -0.70285000
C 8.21039100 -1.25774300 0.36301400
C 7.65049100 -1.84956300 1.50832800
H 6.75127100 -3.57273200 2.38538300
H 8.43865100 -4.08443800 -1.53905600
H 9.05302900 -1.71689100 -1.57430000
H 7.38784700 -1.22694900 2.35788500
C 6.26728200 -5.98778300 1.43845900
C 5.19591600 -6.87170400 1.37039300
C 4.50886600 -7.10124500 0.16188800
C 5.09740900 -6.57377700 -1.00246500
C 6.17433800 -5.69274200 -0.93501300
C 6.71299900 -5.28858400 0.30032100
H 6.72781300 -5.79699600 2.40290100
H 4.85663200 -7.34950600 2.28391600
H 4.64177400 -6.75721800 -1.97051800
H 6.51381000 -5.21995900 -1.85142000
C -6.30710600 5.86168100 -0.97450000
C -5.29618500 6.81858600 -1.03226800
C -4.36698000 6.96917400 0.01452600
C -4.58911200 6.20389800 1.17499600
C -5.60141900 5.25244400 1.23567900
C -6.44706500 5.01158500 0.13751500
H -6.98404800 5.75869100 -1.81755100
H -5.21268400 7.43827500 -1.91982500
H -3.90688200 6.29029000 2.01467700
H -5.67848300 4.62494500 2.11819700
C -7.39587600 3.01381900 -1.04656700
C -7.28869700 3.78746100 0.12331400
C -7.83679400 3.24541300 1.30086500
C -8.32001700 1.94149500 1.33973300
C -8.27769400 1.11163300 0.20340600
C -7.87889000 1.70755300 -1.00754600
H -7.00175400 3.39974400 -1.98180600
H -7.85298900 3.84132100 2.20846400
H -8.70182000 1.54924300 2.27741600

H -7.84769600 1.11093400 -1.91431100
C -7.65927300 -2.38341700 1.45384700
C -7.90390100 -3.18561500 0.32280300
C -8.55699100 -2.56039100 -0.75863300
C -8.79393200 -1.18666500 -0.77120300
C -8.40256500 -0.36581200 0.30391000
C -7.90431300 -1.01657200 1.44660000
H -7.16512100 -2.80441800 2.32158100
H -8.84854600 -3.14259500 -1.62718600
H -9.25963800 -0.74233500 -1.64601900
H -7.59589100 -0.43020900 2.30591900
C -6.92373700 -5.07886600 -1.03317300
C -7.25708900 -4.52308600 0.21838800
C -6.71256900 -5.16435800 1.34765800
C -5.72367200 -6.13301100 1.22575300
C -5.24509800 -6.54220800 -0.03276300
C -5.94987700 -6.06774400 -1.15517300
H -7.35640700 -4.67088000 -1.94084900
H -7.00324500 -4.85043100 2.34427200
H -5.25161500 -6.50384700 2.12961900
H -5.69130900 -6.42427800 -2.14758000
C -1.75920400 -7.35522000 -1.25334600
C -1.17647500 -7.98244400 -0.13762900
C -2.03612700 -8.34394400 0.91502100
C -3.38209000 -7.98795300 0.90579500
C -3.93555900 -7.23626300 -0.14797100
C -3.10198400 -6.98717400 -1.25559400
H -1.13429400 -7.07720400 -2.09735600
H -1.64001400 -8.89251600 1.76446500
H -4.00339700 -8.27393100 1.74868600
H -3.48466900 -6.42411100 -2.10094600
C 1.14096700 -8.32439800 -1.12395700
C 2.51852400 -8.12911900 -1.04450000
C 3.12623500 -7.64948200 0.13302100
C 2.29546600 -7.50449200 1.26063000
C 0.92336800 -7.71616200 1.18689200
C 0.30211400 -8.06972300 -0.02353800
H 0.70828900 -8.66572400 -2.05997500
H 3.12448100 -8.33103400 -1.92240100
H 2.71040700 -7.13203400 2.19093200
H 0.31217100 -7.51234500 2.06075800

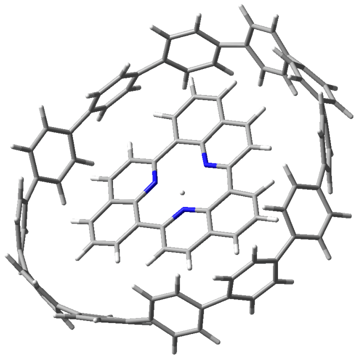

12CPP⊃TQ•H$^+$
B3LYP-D3/6-31G(d,p)
Zero-point correction= 1.334411 (Hartree/Particle)
Thermal correction to Energy= 1.408770
Thermal correction to Enthalpy= 1.409714
Thermal correction to Gibbs Free Energy= 1.224925
Sum of electronic and zero-point Energies= -3974.270630
Sum of electronic and thermal Energies= -3974.196271
Sum of electronic and thermal Enthalpies= -3974.195327
Sum of electronic and thermal Free Energies= -3974.380116

C -2.72890800 -3.18463200 -0.09169700
C -1.81201700 -2.13255300 -0.05246400
C -2.34158200 -0.78918600 -0.03777200
C -3.75405600 -0.58837700 -0.09324100
C -4.63082900 -1.69536000 -0.14556200
C -4.11769100 -2.97441900 -0.13094400
H -2.39471100 -4.21428300 -0.11180700
C -4.21123200 0.75434800 -0.10247300
H -5.70124400 -1.52735800 -0.19431200
H -4.77682600 -3.82999200 -0.16168300
C -3.31579900 1.79099200 -0.06069900
C -1.91330500 1.51421600 0.01605400
H -5.27746500 0.95401000 -0.15307900
H -3.69436400 2.80402200 -0.09295400
C -0.91105400 2.61399600 0.08982300
C 0.52019900 2.40965100 0.01710900
C -1.35552600 3.92427700 0.24710500
C 1.39262300 3.54550400 0.08639100
C -0.48875200 5.03136000 0.32017100
H -2.41358800 4.13570800 0.33649500
C 2.78877300 3.31176800 0.00957900
C 0.87105300 4.85201500 0.24092000
C 2.35326200 0.95452800 -0.14706000
H -0.90802800 6.02058900 0.44400400
C 3.27941700 2.03618300 -0.09780800
H 1.55129400 5.69476500 0.29878600
H 4.35073900 1.88310800 -0.12960500
C 2.80087700 -0.46520700 -0.22334800
C 1.90053500 -1.58491100 -0.16398500
C 4.15693800 -0.76495300 -0.34202800
C 2.41038500 -2.91589300 -0.17975800
C 4.66034500 -2.07820400 -0.37184300
H 4.88044900 0.03787300 -0.41290300
C 1.48471700 -3.98845500 -0.11363900
C 3.80145200 -3.14683400 -0.27159800
C -0.36568500 -2.43063500 -0.06173900
H 5.72700100 -2.23470600 -0.46643000
C 0.13496500 -3.76078400 -0.05783700
H 1.85945800 -5.00816300 -0.11187400
H 4.16941900 -4.16545200 -0.27297900
H -0.53750700 -4.60575300 -0.01883700
N 0.53387000 -1.42662300 -0.09824700
N -1.48473500 0.25558400 0.02454800
N 1.04394900 1.16318800 -0.10417900
H 3.46879300 4.15636600 0.05247700
H 0.16778500 -0.43167500 -0.07753100
C -1.07526100 8.56498600 0.91901700
C -2.39952200 8.16147400 1.05221200
C -3.13625100 7.71253600 -0.05850100
C -2.55982100 7.90695100 -1.32586400
C -1.24590500 8.33835500 -1.45991700
C -0.43087100 8.55994900 -0.33343700
H -0.51179800 8.77833900 1.82062400
H -2.83916300 8.11762000 2.04373300
H -3.10786000 7.61863000 -2.21721100
H -0.82732400 8.41407400 -2.45750700
C 1.89014000 8.77288400 0.67189500

C 1.05034400 8.47752200 -0.42034900
C 1.64524300 7.82564000 -1.51660300
C 2.94589100 7.34410300 -1.45552200
C 3.71824600 7.48084400 -0.28960500
C 3.18862000 8.27734400 0.74135700
H 1.51033800 9.35048300 1.50824100
H 1.05195600 7.57258600 -2.38712700
H 3.31758700 6.74239100 -2.27853100
H 3.78517700 8.47504200 1.62698300
C 6.55726200 5.11630200 -1.08709600
C 6.64758300 4.37413300 0.10479900
C 5.95509200 4.86071300 1.22636400
C 5.10130800 5.95520900 1.12301600
C 4.89974200 6.60563200 -0.10766600
C 5.70275000 6.20708700 -1.19150000
H 7.14272400 4.81602800 -1.95064500
H 6.00243000 4.31653000 2.16447600
H 4.49911800 6.23621700 1.98141100
H 5.62880700 6.73684000 -2.13626900
C 7.88838300 2.51213900 1.29649800
C 7.28772100 3.03830400 0.13911400
C 7.16952300 2.18863100 -0.97552000
C 7.55845100 0.85585600 -0.90869600
C 8.07547900 0.30714700 0.27850300
C 8.27094800 1.17525300 1.36604200
H 8.04530500 3.15520600 2.15715500
H 6.69803900 2.55545100 -1.88184800
H 7.38265700 0.21034600 -1.76388700
H 8.71381200 0.79411100 2.28139200
C 7.43718600 -3.18229800 1.51603000
C 7.63658300 -3.94999700 0.35394800
C 8.30094300 -3.33023200 -0.72147700
C 8.56813200 -1.96530700 -0.70656500
C 8.19683100 -1.16534500 0.39001900
C 7.71470600 -1.81980700 1.53534800
H 6.93166600 -3.62076000 2.36983600
H 8.55549900 -3.90936000 -1.60377400
H 9.02611400 -1.50237100 -1.57545300
H 7.43022900 -1.23651900 2.40552200
C 6.47365800 -6.00011500 1.28566600
C 5.39058100 -6.86575700 1.18507300
C 4.66175600 -6.98607600 -0.01415400
C 5.20426600 -6.36457800 -1.15302600
C 6.29073800 -5.50254400 -1.05366700
C 6.88106400 -5.21609800 0.19022900
H 6.97086900 -5.88575600 2.24398000
H 5.07374200 -7.41459100 2.06626100
H 4.69631500 -6.44417700 -2.10825200
H 6.58456200 -4.93886900 -1.93276000
C -6.28343800 5.72780700 -0.83934900
C -5.32935600 6.74096300 -0.89068500
C -4.33532100 6.85598900 0.09751900
C -4.39987200 5.96814200 1.18759000
C -5.35724900 4.96298000 1.24405700
C -6.29358400 4.79301000 0.20960900
H -7.02553500 5.65222000 -1.62866500
H -5.34517800 7.44122400 -1.72023500
H -3.63978800 6.01045600 1.96092000
H -5.32944700 4.24963600 2.06211000
C -7.26642200 2.81874200 -0.98491500
C -7.12868500 3.56973500 0.19398400
C -7.65591300 3.02681500 1.37904500
C -8.17607300 1.73876600 1.40880300
C -8.18045600 0.93044300 0.25722900
C -7.77874700 1.52429100 -0.95326300
H -6.87558100 3.21278400 -1.91825800
H -7.63055400 3.61395300 2.29203700
H -8.55226300 1.34045300 2.34594100
H -7.76811600 0.93289600 -1.86348400
C -7.65247800 -2.59633600 1.43105200
C -7.92337400 -3.35567300 0.27766400
C -8.58388000 -2.69540200 -0.77665500
C -8.79021000 -1.31930600 -0.75020700
C -8.35766100 -0.53881900 0.33791300
C -7.86578000 -1.22491400 1.46168200
H -7.14469200 -3.05117300 2.27355300
H -8.89457800 -3.25289700 -1.65456100
H -9.25516100 -0.83783100 -1.60519600
H -7.52420400 -0.66635100 2.32672400
C -6.85441500 -5.10800900 -1.16672000
C -7.26079700 -4.67409400 0.10987300
C -6.74316600 -5.38112900 1.21188400
C -5.70076400 -6.28714800 1.05956100
C -5.15230000 -6.56146400 -0.20657700
C -5.83339100 -6.03958000 -1.32133000
H -7.25547800 -4.63172500 -2.05481400
H -7.09182600 -5.15440900 2.21377700
H -5.24184700 -6.70492100 1.94971400
H -5.50624100 -6.29781800 -2.32347400
C -1.58290200 -7.01060300 -1.34142200
C -1.01941100 -7.77444800 -0.30561600
C -1.88686600 -8.29455100 0.66932800
C -3.24681500 -7.99843700 0.65255800
C -3.79649100 -7.14970500 -0.32569900
C -2.93792500 -6.69908400 -1.34648500
H -0.93666900 -6.59210800 -2.10766300
H -1.48641500 -8.92937400 1.45417700
H -3.88663400 -8.41508700 1.42412500
H -3.31590500 -6.02773500 -2.11034900
C 1.27292200 -8.07487200 -1.32035200
C 2.65203100 -7.91270700 -1.24363500
C 3.27432900 -7.51072100 -0.04627200
C 2.46148200 -7.40968800 1.09892700
C 1.08460300 -7.59535100 1.02800900
C 0.45456100 -7.87085000 -0.19621300
H 0.81786600 -8.34839100 -2.26763600
H 3.24986900 -8.07345400 -2.13500600
H 2.89471600 -7.09171300 2.04121600
H 0.47906200 -7.43460600 1.91503300

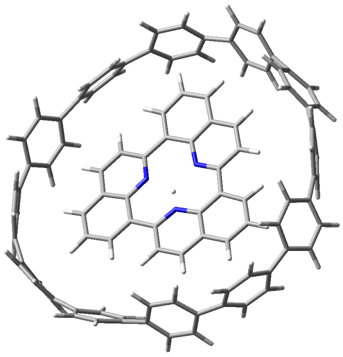

12CPP⊃TQ•H⁺
B3LYP-D3BJ/6-31G(d,p)

Zero-point correction=	1.334868 (Hartree/Particle)
Thermal correction to Energy=	1.409458
Thermal correction to Enthalpy=	1.410402
Thermal correction to Gibbs Free Energy=	1.224529

Sum of electronic and zero-point Energies= -3974.546562
Sum of electronic and thermal Energies= -3974.471972
Sum of electronic and thermal Enthalpies= -3974.471028
Sum of electronic and thermal Free Energies= -3974.656901

C -2.93270800 -2.99925500 -0.08418000
C -1.94917600 -2.00995300 -0.04997000
C -2.38598000 -0.63553000 -0.03353400
C -3.78163400 -0.34147200 -0.07717700
C -4.73004900 -1.38731000 -0.12358000
C -4.30420700 -2.69785200 -0.11504100
H -2.66436200 -4.04730300 -0.10459700
C -4.14907900 1.02799600 -0.08031900
H -5.78634900 -1.14692000 -0.16246200
H -5.01915600 -3.50716000 -0.14377700
C -3.18663900 2.00231400 -0.04437900
C -1.80605600 1.63319500 0.01625400
H -5.19985600 1.29721000 -0.12148300
H -3.49585900 3.03810800 -0.06877300
C -0.73538500 2.66385300 0.07894600
C 0.67704500 2.36219900 0.01490900
C -1.09283000 4.00207000 0.21592800
C 1.62323300 3.43641000 0.07719400
C -0.15445300 5.04885900 0.28125600
H -2.13496100 4.28226600 0.29392400
C 3.00036100 3.10902900 0.01089000
C 1.19017700 4.77609700 0.21380600
C 2.40705900 0.78668900 -0.13123900
H -0.50583900 6.06537600 0.39184200
C 3.40344400 1.80266900 -0.08490400
H 1.92651600 5.56971900 0.26671600
H 4.46094200 1.57535500 -0.11167200
C 2.75994300 -0.65720700 -0.20303600
C 1.78707000 -1.71217500 -0.15245000
C 4.09369200 -1.04452700 -0.31236800
C 2.20641400 -3.07345300 -0.17150300
C 4.50831700 -2.38773800 -0.34540200
H 4.86676000 -0.28944300 -0.37496200
C 1.21113100 -4.08139600 -0.11515900
C 3.57893900 -3.39657600 -0.25723700
C -0.52830800 -2.40292900 -0.06156600
H 5.56207900 -2.61490700 -0.43485000
C -0.11983300 -3.76305300 -0.06309300
H 1.51773100 -5.12294200 -0.11716600
H 3.87675900 -4.43754100 -0.26526100
H -0.84958300 -4.55846400 -0.03053000
N 0.43521500 -1.46146800 -0.09205300
N -1.46087400 0.34930500 0.01955900
N 1.11486400 1.08228300 -0.09330500
H 3.73450800 3.90664800 0.05166400
H 0.13590300 -0.44430900 -0.07168300
C -0.49229400 8.58679000 0.92976300
C -1.83798600 8.26983500 1.06718800
C -2.60805600 7.88826100 -0.04460900
C -2.02943700 8.05807000 -1.31340600
C -0.69148700 8.40244600 -1.45142700
C 0.13945300 8.55782800 -0.32717000
H 0.09318700 8.74848900 1.82751300
H -2.27406700 8.23964200 2.06018100
H -2.60254200 7.81639100 -2.20224100
H -0.27150700 8.46055800 -2.44913800
C 2.46636500 8.61232500 0.67012100
C 1.60844500 8.38120500 -0.42178200
C 2.15126800 7.69848500 -1.52451200
C 3.41518600 7.12916000 -1.47048300
C 4.19594500 7.20656400 -0.30606900
C 3.72715600 8.02966600 0.73203900
H 2.12612500 9.20760300 1.51051300
H 1.53565500 7.49302600 -2.39151800
H 3.74505600 6.50728400 -2.29565400
H 4.33879200 8.17913000 1.61626500
C 6.84880700 4.64621600 -1.11507900
C 6.90459700 3.91391700 0.08372000
C 6.25835800 4.45301300 1.20746500
C 5.47914700 5.60012900 1.10261300
C 5.31125200 6.25188600 -0.13105500
C 6.06897400 5.79008000 -1.22083300
H 7.40224300 4.29794900 -1.98105700
H 6.28090000 3.91368600 2.14869000
H 4.90573500 5.93040700 1.96251600
H 6.01812000 6.31518200 -2.16916300
C 8.00927200 1.98439600 1.29102500
C 7.45869400 2.54372500 0.12555600
C 7.30114000 1.70375100 -0.99003700
C 7.60315800 0.35018500 -0.91702300
C 8.06871100 -0.22626400 0.27702300
C 8.30517700 0.62729100 1.36658200
H 8.19489500 2.61822200 2.15220900
H 6.86515200 2.10057000 -1.90079100
H 7.39797600 -0.28652100 -1.77152200
H 8.71122200 0.21967700 2.28705200
C 7.20756700 -3.65692600 1.52620300
C 7.34544900 -4.43486500 0.36362500
C 8.03962200 -3.86442800 -0.71867600
C 8.39904600 -2.52217100 -0.70759900
C 8.09223600 -1.70021700 0.39131500
C 7.57667300 -2.31743100 1.54148500
H 6.67967200 -4.06219600 2.38222800
H 8.24472200 -4.46070100 -1.60183300
H 8.87961500 -2.09103400 -1.58003000
H 7.33961400 -1.71368900 2.41129100
C 6.03651800 -6.38341900 1.30624100
C 4.89845100 -7.17326100 1.20434000
C 4.17584000 -7.25597800 -0.00039800
C 4.76440100 -6.68196200 -1.13982700
C 5.90536200 -5.89599500 -1.03906100
C 6.50504000 -5.64327400 0.20630300
H 6.53256800 -6.29206000 2.26707900
H 4.53580000 -7.68953800 2.08690500
H 4.25665700 -6.73652000 -2.09631900
H 6.24507400 -5.35890200 -1.91757900

C -5.86794900 6.11186200 -0.83276600
C -4.85022300 7.05917800 -0.88463100
C -3.85917800 7.11524700 0.11002300
C -3.98702700 6.24339500 1.20584900
C -5.00760000 5.30417100 1.26274600
C -5.94439200 5.18893400 0.22266000
H -6.60857200 6.07872500 -1.62575000
H -4.81345800 7.75256600 -1.71865500
H -3.22962100 6.24447300 1.98208300
H -5.03372800 4.59547100 2.08415800
C -7.05058600 3.29310100 -0.97395600
C -6.85425700 4.02405500 0.20797600
C -7.40270000 3.51008100 1.39512100
C -8.00813700 2.26104100 1.42174500
C -8.08004400 1.46634000 0.26435600
C -7.64835100 2.03718000 -0.94545600
H -6.64114400 3.66700400 -1.90700300
H -7.32841200 4.08829600 2.31054600
H -8.40098300 1.88090200 2.35900900
H -7.68650500 1.45052700 -1.85736200
C -7.81018100 -2.08793800 1.42350600
C -8.11784600 -2.81707200 0.26151400
C -8.71772500 -2.10984700 -0.79683500
C -8.83095600 -0.72441600 -0.76315500
C -8.35986700 0.01577200 0.33566600
C -7.92983300 -0.70641200 1.46076100
H -7.34476800 -2.58491500 2.26607600
H -9.05146100 -2.64104600 -1.68199800
H -9.25006800 -0.20626000 -1.61978400
H -7.56204500 -0.17457900 2.33121900
C -7.15348500 -4.61711700 -1.18616800
C -7.54104700 -4.17116300 0.09055700
C -7.08042500 -4.91559700 1.19153100
C -6.10138700 -5.88824400 1.03915600
C -5.56517000 -6.18836200 -0.22512600
C -6.19638500 -5.61227700 -1.34097600
H -7.51488200 -4.10435000 -2.07037700
H -7.42028700 -4.67012900 2.19151200
H -5.67633800 -6.34380600 1.92695800
H -5.87618100 -5.88287800 -2.34150800
C -2.04208200 -6.88833900 -1.35699500
C -1.53214500 -7.67595700 -0.31254200
C -2.43005000 -8.12629200 0.66817500
C -3.76568500 -7.73940000 0.64845900
C -4.25560200 -6.86747400 -0.33937000
C -3.37195300 -6.48667200 -1.36534300
H -1.36842100 -6.52306800 -2.12605100
H -2.07240300 -8.77836400 1.45891200
H -4.43206500 -8.10170600 1.42427500
H -3.70763900 -5.80044200 -2.13490300
C 0.73467800 -8.11153900 -1.32440300
C 2.11988200 -8.03774500 -1.24296300
C 2.76055600 -7.68883500 -0.04010900
C 1.95465800 -7.54570600 1.10379900
C 0.57012200 -7.64232600 1.02790500
C -0.07061700 -7.86679700 -0.20014000
H 0.26556800 -8.34756200 -2.27438400
H 2.71011500 -8.22638500 -2.13330100
H 2.40734200 -7.26452700 2.04803800
H -0.02764300 -7.45002700 1.91328500

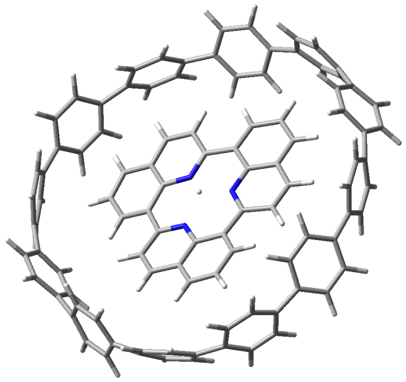

12CPP⊃TQ•H+
LC-BLYP/6-31G(d,p)

Zero-point correction=	1.372756 (Hartree/Particle)
Thermal correction to Energy=	1.444891
Thermal correction to Enthalpy=	1.445836
Thermal correction to Gibbs Free Energy=	1.264201
Sum of electronic and zero-point Energies=	-3961.774066
Sum of electronic and thermal Energies=	-3961.701930
Sum of electronic and thermal Enthalpies=	-3961.700986
Sum of electronic and thermal Free Energies=	-3961.882621

C 1.72386500 3.75804200 -0.18268000
C 1.15226600 2.51483300 -0.08606300
C 2.02241200 1.38227600 -0.07993300
C 3.40431200 1.56866600 -0.20305700
C 3.93814500 2.86451400 -0.30350700
C 3.10800300 3.93957300 -0.27977800
H 1.11490600 4.65211500 -0.21214600
C 4.20704600 0.41054600 -0.24249700
H 5.01162800 2.99072700 -0.40148100
H 3.50259900 4.94336700 -0.35599800
C 3.64174900 -0.81536200 -0.16044500
C 2.23590400 -0.92430100 0.00132200
H 5.28247700 0.51294100 -0.35441700
H 4.27104900 -1.69183000 -0.22888800
C 1.58682900 -2.25116500 0.14241900
C 0.17076600 -2.45053700 0.06329700
C 2.36645800 -3.35030200 0.37965600
C -0.34769900 -3.74883000 0.20015600
C 1.84177000 -4.64332600 0.51837300
H 3.43853000 -3.24630300 0.48648000
C -1.74480200 -3.91209400 0.12799700
C 0.50543700 -4.84509200 0.42654800
C -1.96373100 -1.56487200 -0.15290400
H 2.51546700 -5.47040100 0.70144700
C -2.55676900 -2.84217400 -0.03504900
H 0.07727600 -5.83654300 0.53325800
H -3.62899900 -2.98393300 -0.05229200
C -2.78478400 -0.33363600 -0.27442500

C -2.23517900 0.97804100 -0.19479000
C -4.14232600 -0.42558400 -0.43865800
C -3.07830400 2.09382200 -0.21366500
C -4.98735800 0.68988400 -0.48407000
H -4.60650400 -1.39895200 -0.53431000
C -2.49554700 3.37203500 -0.10192100
C -4.46683400 1.93443100 -0.34795500
C -0.31652000 2.39509700 -0.06082500
H -6.05319900 0.54585900 -0.61336900
C -1.15395300 3.52806500 -0.02474900
H -3.14408000 4.24406700 -0.08253700
H -5.09927200 2.81412300 -0.35586700
H -0.74000900 4.52218400 0.05553900
N -0.88709100 1.20118300 -0.11299800
N 1.49256500 0.14941000 0.03444900
N -0.66961100 -1.40814800 -0.12080000
H -2.16428500 -4.90833300 0.22699400
H -0.25692300 0.34412500 -0.09354900
C 3.20865900 -7.92701700 0.90592300
C 4.40359500 -7.24682200 1.02803600
C 4.98821700 -6.62627600 -0.06894900
C 4.44158500 -6.90054100 -1.31552900
C 3.26297000 -7.60353100 -1.43890600
C 2.55939400 -8.03351400 -0.31952700
H 2.73032200 -8.28936100 1.80847000
H 4.84377000 -7.12888700 2.01266600
H 4.88926300 -6.47133400 -2.20527900
H 2.84636100 -7.73833500 -2.42987100
C 0.38135100 -8.81099600 0.69423100
C 1.10126700 -8.31184800 -0.38748800
C 0.36433000 -7.82615300 -1.46078000
C -1.00581400 -7.70613100 -1.39705600
C -1.70591800 -8.05831400 -0.25169900
C -0.99162700 -8.67874800 0.76613200
H 0.90137800 -9.26573600 1.53000400
H 0.86850800 -7.42325300 -2.32973900
H -1.52733600 -7.22294400 -2.21599700
H -1.51156800 -9.02730600 1.65287400
C -5.06772500 -6.60822800 -1.07983600
C -5.36677600 -5.91671300 0.08888200
C -4.58584100 -6.17536300 1.20669600
C -3.46838800 -6.98245800 1.12211100
C -3.08709000 -7.55284700 -0.08519800
C -3.94934900 -7.40883200 -1.16609500
H -5.70056300 -6.48531100 -1.95258700
H -4.79566100 -5.66101700 2.13862900
H -2.82255400 -7.08317100 1.98840600
H -3.71602700 -7.89748400 -2.10638400
C -7.04663900 -4.43401100 1.24281300
C -6.33942200 -4.80094900 0.10425300
C -6.43541400 -3.97349500 -1.00757600
C -7.12785100 -2.78245900 -0.95193600
C -7.74611700 -2.36582600 0.21995800
C -7.73451700 -3.23827900 1.30073000
H -7.03649000 -5.08301200 2.11246500
H -5.89456100 -4.22825000 -1.91276800
H -7.11867300 -2.12405400 -1.81464800
H -8.24878900 -2.96405900 2.21628700
C -7.89509600 1.15073400 1.45321700
C -8.26640000 1.85353500 0.31417600
C -8.76027000 1.11820200 -0.75851200
C -8.71613300 -0.26116600 -0.75344500
C -8.18684500 -0.95704800 0.32840200
C -7.85962900 -0.22784000 1.46229500
H -7.50690800 1.68658300 2.31191400
H -9.13124600 1.63134700 -1.63968800
H -9.05182100 -0.80704900 -1.62933700
H -7.45417600 -0.73944600 2.32866600
C -7.65587700 4.09293200 1.28596700
C -6.84693400 5.20637600 1.20952300
C -6.19503300 5.54239700 0.02676300
C -6.54773900 4.83310400 -1.11335400
C -7.36198300 3.72106200 -1.03769700
C -7.84927700 3.26973300 0.18160600
H -8.09424900 3.82452000 2.24140400
H -6.67450200 5.79267800 2.10574000
H -6.08543400 5.07358600 -2.06431500
H -7.50632600 3.12160800 -1.92982100
C 7.54419800 -3.96880500 -0.87246000
C 6.86722600 -5.17183900 -0.91769100
C 5.97241000 -5.53051300 0.08327800
C 5.86662100 -4.68625300 1.18191500
C 6.54691500 -3.48885900 1.23098900
C 7.35958800 -3.08254600 0.18087100
H 8.21059700 -3.70169800 -1.68653300
H 7.01493800 -5.82975500 -1.76782800
H 5.17345800 -4.92885500 1.98017700
H 6.38032600 -2.81814400 2.06742800
C 7.72683900 -0.93259500 -1.00939000
C 7.83492900 -1.68169100 0.15376600
C 8.21662700 -1.02036600 1.31512100
C 8.34916100 0.35101800 1.34088400
C 8.09949700 1.11624100 0.20731600
C 7.85641200 0.44251500 -0.98270400
H 7.44277900 -1.42155900 -1.93569300
H 8.37384100 -1.58833500 2.22631000
H 8.60993400 0.84150500 2.27282900
H 7.66663100 1.00796100 -1.88931900
C 6.63060100 4.32921000 1.40023000
C 6.69695000 5.14719100 0.27893800
C 7.48849500 4.70592600 -0.77716200
C 8.04946800 3.44382300 -0.77370600
C 7.85336600 2.57373100 0.29281300
C 7.19729300 3.07428200 1.40877700
H 6.02584800 4.62048300 2.24984200
H 7.63115800 5.33590300 -1.64847600
H 8.61570400 3.11473000 -1.63913300
H 7.02590300 2.43014800 2.26393800
C 5.26231200 6.65522000 -1.10311700
C 5.73181500 6.26819100 0.14739500

C 5.05503700 6.76979300 1.25364800
C 3.84786300 7.41980000 1.11587700
C 3.28208700 7.63091500 -0.13555500
C 4.06826300 7.33376000 -1.24200900
H 5.77724600 6.33041300 -1.99997200
H 5.42572800 6.57331100 2.25287800
H 3.29033800 7.67644700 2.00986800
H 3.70449300 7.55687400 -2.23933200
C -0.26078700 7.30306700 -1.29532600
C -0.98268300 7.84709800 -0.24199000
C -0.27601800 8.51340200 0.75081300
C 1.10385300 8.55120300 0.73852500
C 1.83166700 7.90867200 -0.25642800
C 1.11819600 7.32806800 -1.29856900
H -0.78659100 6.78176000 -2.08919300
H -0.81618600 8.98987000 1.56279400
H 1.62436700 9.06291800 1.54113800
H 1.64924700 6.81201500 -2.09092100
C -3.27761600 7.57042000 -1.22888400
C -4.54598100 7.03200800 -1.15803500
C -5.00778800 6.42999400 0.00785000
C -4.19260600 6.50348000 1.13139900
C -2.93077700 7.05765000 1.06606500
C -2.42699300 7.54681600 -0.13063700
H -2.92925600 7.99517900 -2.16497200
H -5.17570100 7.05127800 -2.04109300
H -4.51264000 6.04260900 2.05913600
H -2.29213300 7.03283000 1.94323800

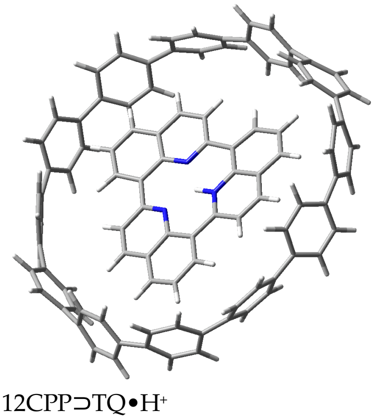

12CPP⊃TQ•H+
BMK/6-31G(d,p)
Zero-point correction= 1.336167 (Hartree/Particle)
Thermal correction to Energy= 1.409501
Thermal correction to Enthalpy= 1.410445
Thermal correction to Gibbs Free Energy= 1.228964
Sum of electronic and zero-point Energies= -3971.324287
Sum of electronic and thermal Energies= -3971.250953
Sum of electronic and thermal Enthalpies= -3971.250009
Sum of electronic and thermal Free Energies= -3971.431490
C 1.93338200 -3.71707700 -0.13414700
C 1.29584100 -2.47672500 -0.07673900
C -0.15196000 -2.46597500 -0.06006100
C -0.86231000 -3.70217900 -0.12836800
C -0.16115600 -4.93370500 -0.19066900
C 1.21839100 -4.93445000 -0.18077700
H 3.01642600 -3.78883800 -0.16654500
C -2.28350700 -3.62843900 -0.14208000
H -0.72190400 -5.86598000 -0.24505100
H 1.77039500 -5.86766400 -0.22055500
C -2.91268400 -2.41038900 -0.09103900
C -2.12454600 -1.21208200 0.00342600
H -2.86571300 -4.54824200 -0.20293100
H -3.99627800 -2.38087900 -0.12960500
C -2.77534200 0.13484900 0.09425400
C -2.04864100 1.38921300 0.01941900
C -4.15419500 0.20877100 0.27263800
C -2.77499800 2.62116400 0.11313600
C -4.85998500 1.43243400 0.36398500
H -4.74606800 -0.69646700 0.36832200
C -2.03629000 3.83444800 0.04270100
C -4.18472000 2.62786100 0.28724400
C -0.01945400 2.54110200 -0.16200900
H -5.93682700 1.41060400 0.50083100
C -0.67011800 3.81273600 -0.08397800
H -4.70605200 3.58059100 0.36674900
H -0.12269500 4.74982300 -0.10680800
C 1.47167300 2.42616000 -0.25776300
C 2.17818800 1.17085900 -0.19055100
C 2.25109000 3.57318500 -0.39540600
C 3.60348800 1.15253000 -0.20597700
C 3.66431600 3.55504700 -0.42838300
H 1.76886300 4.54294800 -0.47918600
C 4.25857100 -0.10789200 -0.12119500
C 4.33874000 2.36081900 -0.31253900
C 2.11790500 -1.23932500 -0.07644500
H 4.20434600 4.49212500 -0.53593500
C 3.54392500 -1.27732400 -0.05851300
H 5.34910400 -0.13610800 -0.11265900
H 5.42564400 2.31957400 -0.31343700
H 4.07612300 -2.21977700 -0.00215600
N 1.52711500 -0.03688500 -0.12022200
N -0.80391200 -1.28439600 0.01475600
N -0.70027300 1.41129600 -0.12370700
H -2.56857600 4.78310700 0.10545200
H 0.47392600 -0.00673400 -0.10264800
C -8.30736800 2.55792400 0.93982100
C -8.52651800 1.18462800 1.06333600
C -8.41731100 0.33030200 -0.05239200
C -8.31067200 0.93893800 -1.31815300
C -8.12013500 2.31467700 -1.44291000
C -7.99250200 3.14070300 -0.30650800
H -8.28259200 3.15764700 1.84574300
H -8.70431500 0.76484400 2.05123000
H -8.26971400 0.32165100 -2.21280000
H -7.98347900 2.72740600 -2.43848200
C -7.17571500 5.31746500 0.73655300
C -7.28339000 4.45685500 -0.37778200
C -6.46336900 4.74798500 -1.48747400
C -5.47847500 5.73190400 -1.42528200
C -5.25978400 6.47112400 -0.24695200

C -6.18066800 6.29556000 0.80552400
H -7.83989800 5.19367300 1.58837600
H -6.50450700 4.12626800 -2.37643200
H -4.79694200 5.84448900 -2.26529200
H -6.09493400 6.90310600 1.70458300
C -1.94878800 8.12201500 -1.09639600
C -1.21908300 7.91707300 0.09285200
C -1.92931900 7.48130000 1.22797200
C -3.27997500 7.13522500 1.14193500
C -3.97111800 7.20365600 -0.08422900
C -3.29594300 7.77188000 -1.18372100
H -1.44823400 8.53342900 -1.97057500
H -1.40054500 7.31899400 2.16484700
H -3.77244300 6.70599700 2.01214000
H -3.82250300 7.90908300 -2.12604100
C 1.00283300 8.29366100 1.27659300
C 0.27114100 7.97249800 0.11541100
C 0.99439700 7.53698400 -1.01321400
C 2.37429600 7.33958300 -0.95268000
C 3.09010200 7.56225200 0.24069800
C 2.38374400 8.09049100 1.33904600
H 0.48288500 8.68906700 2.14702700
H 0.46184100 7.27717000 -1.92551200
H 2.89000300 6.92845500 -1.81841100
H 2.91682000 8.32474100 2.25865600
C 5.97666700 5.48700100 1.49437800
C 6.74789000 5.31313500 0.32747000
C 6.47052500 6.16092900 -0.76587900
C 5.35461000 6.99996200 -0.75714400
C 4.48112300 7.03551500 0.35009500
C 4.86869400 6.33549900 1.50792900
H 6.16268100 4.85914300 2.36214000
H 7.09830900 6.12373300 -1.65381600
H 5.13056200 7.59916900 -1.63769000
H 4.22680900 6.35213200 2.38610000
C 8.10886200 3.39762500 1.30272700
C 8.45557900 2.04867100 1.22056800
C 8.28765600 1.32397100 0.02117200
C 7.95163000 2.05970000 -1.13219700
C 7.60964600 3.41070700 -1.05104500
C 7.58132800 4.08188500 0.18769400
H 8.19758300 3.90872800 2.25911100
H 8.81383500 1.54123300 2.11362800
H 7.83472500 1.55097800 -2.08602300
H 7.23119100 3.90533100 -1.94247100
C -7.89729600 -3.36005500 -0.86761700
C -8.43289400 -2.06992100 -0.91341800
C -8.15528100 -1.13020800 0.09981900
C -7.40730900 -1.57074500 1.21086700
C -6.88065100 -2.86089400 1.26167700
C -7.07260000 -3.76619800 0.19964900
H -8.10828200 -4.05582900 -1.67779100
H -9.05383500 -1.78309100 -1.75982300
H -7.16246200 -0.87070400 2.00657800
H -6.24555400 -3.14380200 2.09870800
C -5.63299000 -5.43342700 -1.01013600
C -6.27224500 -5.02362900 0.17524500
C -5.98087200 -5.73331000 1.35760700
C -4.99565500 -6.71987700 1.37947100
C -4.25417800 -7.03176800 0.22134300
C -4.64142600 -6.41809200 -0.98698500
H -5.84518000 -4.91222600 -1.94170900
H -6.51283800 -5.48758200 2.27463900
H -4.77567000 -7.23102800 2.31435000
H -4.09476700 -6.63875900 -1.90153900
C -0.80908300 -8.00262700 1.41464300
C -0.21578500 -8.55363100 0.26030500
C -1.07505300 -8.85894800 -0.81705600
C -2.41876700 -8.47627600 -0.80269200
C -2.96726500 -7.78259700 0.29579400
C -2.15132900 -7.62895900 1.43346400
H -0.19502100 -7.74669500 2.27304900
H -0.68411500 -9.35886100 -1.70040400
H -3.03893000 -8.68707900 -1.67185700
H -2.53523200 -7.10591800 2.30590000
C 1.86717600 -8.37678800 -1.15201300
C 1.27244700 -8.51991400 0.11943800
C 2.11179900 -8.34091000 1.23900600
C 3.39415100 -7.81053100 1.09969900
C 3.90875100 -7.47008400 -0.16764000
C 3.16272100 -7.87604100 -1.29282800
H 1.28085600 -8.55378300 -2.05009100
H 1.73419400 -8.53192800 2.24014000
H 3.95613700 -7.56215500 1.99704000
H 3.56096200 -7.71989800 -2.29293300
C 5.86655300 -4.44755100 -1.32497300
C 6.75873100 -4.22609200 -0.25840400
C 6.84327600 -5.21220800 0.74357900
C 6.00540100 -6.33000600 0.72569300
C 5.03424100 -6.49666800 -0.28282400
C 5.01750400 -5.55484100 -1.33248700
H 5.78393700 -3.70475600 -2.11650500
H 7.56183200 -5.09361100 1.55266400
H 6.09037800 -7.06730400 1.52122100
H 4.27600400 -5.64195400 -2.12311100
C 7.97180400 -2.24198000 -1.27146600
C 8.36511900 -0.90469000 -1.19790800
C 8.22262200 -0.16754700 -0.00346100
C 7.79277000 -0.86623100 1.14388500
C 7.41737700 -2.20918700 1.07541100
C 7.44097900 -2.90537100 -0.14761400
H 8.05979900 -2.77550900 -2.21613900
H 8.76108700 -0.42131900 -2.08832200
H 7.66465900 -0.33443000 2.08352200
H 7.02004600 -2.69904100 1.96244700

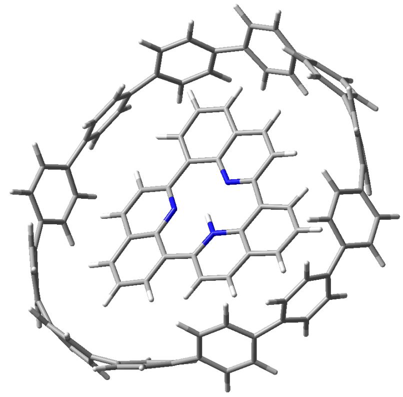

12CPP⊃TQ•H+
ωB97/6-31G(d,p)
Zero-point correction= 1.351332 (Hartree/Particle)
Thermal correction to Energy= 1.424992
Thermal correction to Enthalpy= 1.425936
Thermal correction to Gibbs Free Energy= 1.242163
Sum of electronic and zero-point Energies= -3973.278786
Sum of electronic and thermal Energies= -3973.205125
Sum of electronic and thermal Enthalpies= -3973.204181
Sum of electronic and thermal Free Energies= -3973.387954
C -2.10011800 -3.61482100 -0.09565600
C -1.39988500 -2.42045000 -0.04433300
C -2.16270000 -1.19542900 -0.04063900
C -3.57272800 -1.25265100 -0.12044400
C -4.23823500 -2.50244200 -0.18289500
C -3.50927800 -3.66340400 -0.15496900
H -1.58086800 -4.57020400 -0.11378600
C -4.26955900 -0.01357500 -0.14756000
H -5.32624200 -2.52402000 -0.25332100
H -4.00225000 -4.63082100 -0.19405600
C -3.58145200 1.16450100 -0.10215400
C -2.15297900 1.13921800 0.00289200
H -5.35884100 -0.01485900 -0.21668400
H -4.13508000 2.09780100 -0.15170600
C -1.36305000 2.41180600 0.08825700
C 0.08122000 2.46917400 0.01406700
C -2.03614700 3.60590500 0.26307100
C 0.72887500 3.72829400 0.10401900
C -1.38244300 4.85673400 0.35458700
H -3.11966000 3.61857100 0.35556900
C 2.14760800 3.75362100 0.03369300
C -0.01913200 4.92244200 0.27815200
C 2.13616600 1.36707100 -0.15621500
H -1.97723700 5.75627100 0.49455700
C 2.85915100 2.59374100 -0.08421500
H 0.50789800 5.87380100 0.35743500
H 3.94541000 2.63320400 -0.10655900
C 2.83958100 0.04384700 -0.24127200
C 2.15918100 -1.22072200 -0.16526000
C 4.21620000 -0.00190400 -0.37732900
C 2.89446400 -2.42601200 -0.17431800
C 4.95462900 -1.20548000 -0.40547500
H 4.78110400 0.92385900 -0.46365700
C 2.17949800 -3.65171100 -0.08801700
C 4.30662800 -2.40447600 -0.28160300
C 0.08791900 -2.45382800 -0.04420200
H 6.03646000 -1.16549100 -0.51771400
C 0.81531000 -3.67545500 -0.02469700
H 2.74030800 -4.58885300 -0.07787100
H 4.85085100 -3.34743200 -0.27814700
H 0.30380100 -4.63078100 0.03124000
N 0.78391700 -1.31372400 -0.09345200
N -1.50570400 -0.00912400 0.03250400
N 0.82096400 1.33280000 -0.12390300
H 2.66323200 4.71317100 0.09270000
H 0.24823500 -0.40591800 -0.08126300
C -2.44179200 8.26150000 0.93571300
C -3.69264200 7.66849800 1.07489400
C -4.35405000 7.12121200 -0.03239200
C -3.82013700 7.38835000 -1.29927900
C -2.58435100 8.00845100 -1.43909900
C -1.81674800 8.35206800 -0.31687100
H -1.90599700 8.55581800 1.83611800
H -4.11671400 7.55142600 2.07140700
H -4.32643400 7.01490100 -2.18837700
H -2.17811500 8.13851900 -2.44014500
C 0.45111500 8.93911300 0.66864400
C -0.33091700 8.49821000 -0.41068900
C 0.34340700 7.92199400 -1.49577300
C 1.70554900 7.65687800 -1.44072400
C 2.45083100 7.94755100 -0.29221100
C 1.81348800 8.66057900 0.73246800
H -0.01573200 9.45781400 1.50443000
H -0.21452500 7.55823700 -2.35517200
H 2.16901200 7.10595900 -2.25796500
H 2.37932500 8.96133100 1.61379400
C 5.64840400 6.10213700 -1.10938300
C 5.86604700 5.38956600 0.07860800
C 5.10751600 5.73887700 1.20183500
C 4.07948100 6.67311600 1.10558600
C 3.77322300 7.28264000 -0.11787000
C 4.62005300 7.03157300 -1.20657900
H 6.26997000 5.89831500 -1.98053900
H 5.25689700 5.20581200 2.14017900
H 3.43862000 6.85031400 1.96883600
H 4.44329900 7.53939600 -2.15401600
C 7.42966600 3.77253300 1.25320300
C 6.72898700 4.17504200 0.10837500
C 6.72232800 3.31168900 -0.99529700
C 7.32213700 2.05929500 -0.92682000
C 7.94295800 1.62049000 0.25013700
C 8.02399100 2.51536800 1.32443200
H 7.49089000 4.44332200 2.10952900
H 6.17990900 3.59548500 -1.89664200
H 7.24006600 1.38006900 -1.77551400
H 8.53730400 2.21470100 2.23730300
C 7.82267900 -1.91746400 1.50321400
C 8.14759200 -2.64879100 0.35332500
C 8.71273600 -1.95090600 -0.72478500
C 8.77521200 -0.56127300 -0.71881300

C 8.28763700 0.17574000 0.37063600
C 7.89607000 -0.52842800 1.51431200
H 7.38279400 -2.42615500 2.35934300
H 9.04771800 -2.49524000 -1.60699000
H 9.15624800 -0.03755800 -1.59501000
H 7.52319000 0.01747900 2.38008200
C 7.32352700 -4.84973300 1.30609700
C 6.39654100 -5.88164000 1.21526100
C 5.70543000 -6.12943000 0.01899100
C 6.12564700 -5.43633500 -1.12282800
C 7.05690400 -4.40661100 -1.03286500
C 7.59660600 -4.02775600 0.20266200
H 7.79179000 -4.63913200 2.26680700
H 6.16413600 -6.46413000 2.10574800
H 5.63146000 -5.61061300 -2.07731200
H 7.25595700 -3.80349100 -1.91745300
C -7.16798600 4.68141000 -0.80483800
C -6.38983000 5.83588000 -0.84858700
C -5.41446300 6.08542900 0.12611200
C -5.31206300 5.18491800 1.19558900
C -6.09414100 4.03744300 1.24481200
C -7.00471800 3.74338100 0.22214000
H -7.89937800 4.49539900 -1.59090600
H -6.52403400 6.53909100 -1.66983500
H -4.55310000 5.34218900 1.96135000
H -5.94337700 3.31976700 2.05096400
C -7.60808600 1.63502800 -0.98428500
C -7.62418400 2.38896600 0.19440700
C -8.06388100 1.76579800 1.37034700
C -8.35442700 0.40728300 1.39188500
C -8.20510000 -0.37859200 0.24029100
C -7.89172400 0.27131700 -0.96071200
H -7.27878400 2.09900500 -1.91372700
H -8.14291700 2.34799500 2.28783300
H -8.66094500 -0.05922600 2.32724800
H -7.77107300 -0.31242600 -1.87286600
C -7.07840200 -3.76663800 1.40406600
C -7.24351800 -4.56239900 0.26244400
C -8.00291700 -4.02728400 -0.78959100
C -8.43069600 -2.70273400 -0.76590600
C -8.12620400 -1.86552400 0.31714000
C -7.51279200 -2.44780000 1.43261100
H -6.49158400 -4.13323600 2.24331600
H -8.21825100 -4.63329000 -1.66853300
H -8.96916700 -2.30136400 -1.62388300
H -7.26186800 -1.83068600 2.29382600
C -5.91773000 -6.13837600 -1.17362100
C -6.37968100 -5.77144900 0.09881200
C -5.76417500 -6.37926000 1.20274000
C -4.59964100 -7.12275800 1.05165100
C -4.02762500 -7.32081400 -0.21230400
C -4.77116100 -6.91115500 -1.32724900
H -6.38907900 -5.72513900 -2.06351300
H -6.13952900 -6.19308700 2.20726700
H -4.07490200 -7.46388200 1.94286400
H -4.40245300 -7.11718600 -2.33113800
C -0.42590600 -7.18304100 -1.31563200
C 0.23843700 -7.86684800 -0.29048800
C -0.53468100 -8.53916200 0.66411600
C -1.92470100 -8.46406700 0.63858100
C -2.58881900 -7.69732000 -0.32899800
C -1.81358600 -7.09355000 -1.32938500
H 0.15600600 -6.65434000 -2.07107300
H -0.03983700 -9.11182900 1.44797400
H -2.49973500 -8.98532600 1.40289600
H -2.29600300 -6.47857300 -2.08809700
C 2.56434400 -7.81500900 -1.27877000
C 3.89545300 -7.42016400 -1.19697600
C 4.41968100 -6.88749700 -0.00960400
C 3.59290500 -6.88288100 1.12285700
C 2.26672000 -7.29806200 1.04758100
C 1.71528400 -7.71518200 -0.16880900
H 2.16872600 -8.18483800 -2.22422500
H 4.52615900 -7.49584200 -2.08159900
H 3.96240800 -6.46954200 2.05996100
H 1.62727100 -7.21847100 1.92649300

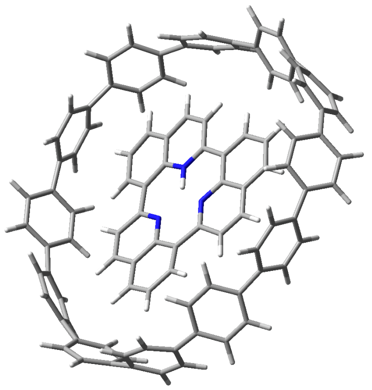

12CPP⊃TQ•H+
ωB97D/6-31G(d,p)

Zero-point correction=	1.348175 (Hartree/Particle)
Thermal correction to Energy=	1.421926
Thermal correction to Enthalpy=	1.422870
Thermal correction to Gibbs Free Energy=	1.238470
Sum of electronic and zero-point Energies=	-3972.736013
Sum of electronic and thermal Energies=	-3972.662263
Sum of electronic and thermal Enthalpies=	-3972.661318
Sum of electronic and thermal Free Energies=	-3972.845718

C 2.59572600 3.26638100 -0.07321200
C 1.72605300 2.18685800 -0.03195300
C 2.30052600 0.86781000 -0.04334800
C 3.70635300 0.71851200 -0.13360400
C 4.54249100 1.85451400 -0.19161100
C 3.98960200 3.10806300 -0.14407000
H 2.22553400 4.28431800 -0.07423000
C 4.21139600 -0.60396100 -0.17594400
H 5.61701900 1.72234400 -0.27177600
H 4.61684900 3.98869900 -0.17757700
C 3.35820500 -1.66841700 -0.13375600
C 1.95438000 -1.43929400 -0.01400300

H 5.28358600 -0.76304100 -0.25287900
H 3.76887900 -2.66772900 -0.19768600
C 0.99359100 -2.57717600 0.07732800
C -0.43909600 -2.42528200 0.01407200
C 1.48669700 -3.85792500 0.25155900
C -1.26424300 -3.57923600 0.11569800
C 0.66201900 -4.99343400 0.35575400
H 2.55315800 -4.02817300 0.33519000
C -2.66648700 -3.39705300 0.05492000
C -0.69720800 -4.86231800 0.29220400
C -2.31232900 -1.04060700 -0.14938400
H 1.11810800 -5.96553800 0.49418500
C -3.20086300 -2.14605700 -0.06318400
H -1.34979200 -5.72520200 0.38172900
H -4.27760300 -2.02952200 -0.07254500
C -2.81057500 0.36352800 -0.24108000
C -1.95546900 1.51130600 -0.16163100
C -4.16540200 0.60991500 -0.39102100
C -2.50977800 2.81293700 -0.17416800
C -4.71696500 1.90242900 -0.42638200
H -4.85602700 -0.21983700 -0.48423300
C -1.62914600 3.91747300 -0.08023200
C -3.90418300 2.99475600 -0.29314600
C 0.26664800 2.43359200 -0.02942100
H -5.78717400 2.01979700 -0.55034600
C -0.27749600 3.74185400 -0.00792600
H -2.04411900 4.92238500 -0.07196300
H -4.30764700 4.00084000 -0.29260300
H 0.36189600 4.61121100 0.05354100
N -0.58687900 1.40398100 -0.08363100
N 1.48234600 -0.20678200 0.02725600
N -1.00480100 -1.19998900 -0.12626300
H -3.31547000 -4.26525900 0.12156700
H -0.18214400 0.42743800 -0.07433000
C 1.27954400 -8.50495000 0.90221000
C 2.59433500 -8.08322200 1.04884900
C 3.32636100 -7.62205800 -0.05138800
C 2.76945700 -7.81641500 -1.32029800
C 1.46738800 -8.27048600 -1.46794900
C 0.65429000 -8.50885600 -0.35209600
H 0.70790800 -8.72538700 1.79721500
H 3.02356000 -8.02994000 2.04453100
H 3.32193600 -7.51309800 -2.20404600
H 1.05724100 -8.35070200 -2.46881700
C -1.67194600 -8.81709800 0.60855100
C -0.82990400 -8.45086600 -0.45141600
C -1.41256800 -7.75397100 -1.51723700
C -2.72330800 -7.30875800 -1.45641000
C -3.50762200 -7.53006900 -0.31998600
C -2.98090400 -8.35655200 0.67958200
H -1.28979500 -9.42540500 1.42195900
H -0.80671400 -7.43811800 -2.35824700
H -3.09829400 -6.67303100 -2.25216600
H -3.58683800 -8.60778100 1.54507800
C -6.41878800 -5.26183800 -1.09390800
C -6.52194600 -4.53084300 0.09646800
C -5.81289300 -4.99259200 1.21008300
C -4.92977900 -6.05982900 1.10010400
C -4.71999100 -6.69634000 -0.12875900
C -5.53342400 -6.32342000 -1.20551800
H -7.01495500 -4.97386200 -1.95438000
H -5.87350700 -4.45160400 2.14940500
H -4.31553700 -6.32910500 1.95410600
H -5.44202300 -6.84531400 -2.15316900
C -7.82819400 -2.71717100 1.28500400
C -7.19924200 -3.21124200 0.13623500
C -7.08036600 -2.35373300 -0.96421000
C -7.50022900 -1.03373900 -0.89023700
C -8.04505300 -0.51518100 0.29030500
C -8.23995900 -1.39204700 1.36254100
H -7.98129400 -3.37265800 2.13695700
H -6.58756100 -2.70302000 -1.86638900
H -7.33005200 -0.37375500 -1.73603000
H -8.70138500 -1.02834700 2.27578700
C -7.43255300 2.97012900 1.52124300
C -7.68857600 3.73531400 0.37710900
C -8.36770800 3.11553800 -0.68044200
C -8.60877800 1.74815900 -0.66601400
C -8.19233900 0.95613300 0.41194100
C -7.68408200 1.60532700 1.54079600
H -6.90786400 3.41572500 2.35987200
H -8.65588500 3.69546300 -1.55191100
H -9.07964600 1.27989500 -1.52514400
H -7.36225200 1.01998300 2.39653100
C -6.59022200 5.82207000 1.29343100
C -5.53423300 6.71615000 1.18849200
C -4.81042500 6.84289500 -0.00551500
C -5.31967400 6.19408900 -1.13589600
C -6.38013600 5.30435600 -1.03244300
C -6.96678800 5.02256900 0.20643200
H -7.08648300 5.69928800 2.25121900
H -5.23410000 7.28123100 2.06536900
H -4.80520900 6.27753800 -2.08758300
H -6.65340300 4.71870800 -1.90420400
C 6.44825400 -5.59219100 -0.78499700
C 5.52300700 -6.62856400 -0.83856300
C 4.50583000 -6.73705000 0.11688700
C 4.50733400 -5.82308400 1.17831500
C 5.43479500 -4.79424900 1.23713700
C 6.39620600 -4.63443300 0.23308300
H 7.21239200 -5.51648400 -1.55289700
H 5.57988500 -7.34926700 -1.64865600
H 3.72209500 -5.86706100 1.92670000
H 5.36523400 -4.05869200 2.03303600
C 7.30563200 -2.63618200 -0.95903800
C 7.19825000 -3.38666500 0.21573300
C 7.70863200 -2.83537500 1.39680900
C 8.18787300 -1.53439900 1.42714700
C 8.16390900 -0.73228900 0.27953000
C 7.77817500 -1.32922200 -0.92679900

H 6.92595700 -3.04343600 -1.89145200
H 7.69816900 -3.42361600 2.30938200
H 8.54939100 -1.12246300 2.36425700
H 7.75050400 -0.73499800 -1.83521000
C 7.48048500 2.76681800 1.42395400
C 7.79035500 3.53325800 0.29379800
C 8.50595500 2.90124900 -0.73271100
C 8.74604500 1.53298700 -0.70253300
C 8.29150600 0.74424200 0.36211300
C 7.72796500 1.40391300 1.45916400
H 6.91901700 3.20594200 2.24062600
H 8.83657200 3.47180000 -1.59498500
H 9.25625900 1.06568200 -1.53945500
H 7.36251500 0.82896400 2.30369600
C 6.69726200 5.24701000 -1.16625900
C 7.10497000 4.84085000 0.11142800
C 6.57669000 5.54337400 1.20254700
C 5.52065000 6.42739600 1.03776900
C 4.97872300 6.67807200 -0.22889000
C 5.66473600 6.15997400 -1.33392900
H 7.10744800 4.76312600 -2.04628500
H 6.92639500 5.33053500 2.20723500
H 5.04849700 6.84566800 1.92089900
H 5.33262500 6.39960500 -2.33911400
C 1.39647800 6.99316900 -1.33127900
C 0.83230100 7.78604900 -0.32676300
C 1.69045600 8.37133500 0.60961200
C 3.05506800 8.10995500 0.58798800
C 3.60804300 7.23546100 -0.35557700
C 2.75622100 6.71659900 -1.33969800
H 0.75040500 6.52798300 -2.07062700
H 1.28149800 9.02641600 1.37302400
H 3.69359800 8.57333300 1.33367100
H 3.14521800 6.02144400 -2.07687300
C -1.47217300 8.05357800 -1.31366600
C -2.84336400 7.85403100 -1.22889300
C -3.43683500 7.40899300 -0.03992000
C -2.61752600 7.29616000 1.09121400
C -1.24906300 7.51904000 1.01322200
C -0.64510500 7.84303300 -0.20502200
H -1.03164600 8.35804000 -2.25830900
H -3.45506100 8.01756600 -2.11046400
H -3.03655000 6.94378500 2.02798400
H -0.62957700 7.35544800 1.89014600

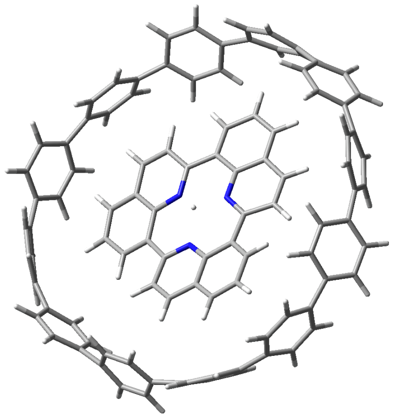

12CPP⊃TQ•H+
LC-ωPBE/6-31G(d,p)
Zero-point correction= 1.359862 (Hartree/Particle)
Thermal correction to Energy= 1.433334
Thermal correction to Enthalpy= 1.434278
Thermal correction to Gibbs Free Energy= 1.249186
Sum of electronic and zero-point Energies= -3970.980799
Sum of electronic and thermal Energies= -3970.907328
Sum of electronic and thermal Enthalpies= -3970.906383
Sum of electronic and thermal Free Energies= -3971.091476
C 1.01068100 4.00527300 -0.22271700
C 0.67741000 2.67424900 -0.09974400
C 1.74313300 1.71581400 -0.11445200
C 3.06792100 2.14883700 -0.30131100
C 3.35369300 3.52419800 -0.43053000
C 2.33920100 4.43477200 -0.37292500
H 0.24748500 4.77362600 -0.23599100
C 4.06660100 1.14980700 -0.37780900
H 4.38159200 3.84302900 -0.57587700
H 2.54243800 5.49392800 -0.46702500
C 3.73218200 -0.16270000 -0.26492900
C 2.37560400 -0.51602500 -0.02191500
H 5.10015900 1.44145400 -0.54063000
H 4.49961300 -0.91882200 -0.36962800
C 1.98270900 -1.93621100 0.18022000
C 0.62434100 -2.39636800 0.11016100
C 2.95401600 -2.86065700 0.47975900
C 0.34907500 -3.76403500 0.31804800
C 2.67224400 -4.22365600 0.68730600
H 3.98671000 -2.54955000 0.58562100
C -1.00001400 -4.18148800 0.26162000
C 1.39097200 -4.67462300 0.60383300
C -1.63717400 -1.92088000 -0.14056800
H 3.48450800 -4.90439700 0.91193600
C -1.99473300 -3.27903500 0.05138500
H 1.15165900 -5.72210600 0.76152600
H -3.02611400 -3.60799900 0.05815400
C -2.66991200 -0.86059600 -0.30820500
C -2.37491400 0.53439300 -0.21759400
C -3.98619800 -1.20374600 -0.51843700
C -3.41407400 1.47876700 -0.24927000
C -5.02539000 -0.26061800 -0.58670900
H -4.25518300 -2.24716400 -0.63226400
C -3.07920200 2.84534700 -0.10684600

C -4.74942500 1.06300700 -0.42297500
C -0.74843500 2.28630200 -0.05320600
H -6.04344400 -0.59802200 -0.74690600
C -1.78463600 3.24668000 -0.00462200
H -3.87761800 3.58259500 -0.08735900
H -5.53564100 1.80930500 -0.43828100
H -1.56147300 4.29885100 0.09975200
N -1.08977400 1.00319500 -0.11662500
N 1.44672600 0.40792200 0.04234800
N -0.38816600 -1.52814600 -0.13006900
H -1.23546800 -5.23061100 0.41203500
H -0.31209600 0.27862100 -0.09788100
C 4.52683200 -7.28687400 0.87783200
C 5.61547100 -6.43592700 1.00269900
C 6.08828000 -5.70877900 -0.09116800
C 5.56848100 -6.03877700 -1.34278600
C 4.50275900 -6.91238800 -1.46996200
C 3.88182300 -7.46632100 -0.34848000
H 4.12095900 -7.73749400 1.77789000
H 6.04961500 -6.27798900 1.98571500
H 5.93587900 -5.53189900 -2.22973400
H 4.10216500 -7.09505900 -2.46136800
C 1.86509600 -8.65821600 0.63671600
C 2.47977200 -7.97220500 -0.41524600
C 1.65369300 -7.54335900 -1.45562800
C 0.27871100 -7.67188100 -1.38760900
C -0.34139600 -8.22499500 -0.26807400
C 0.48362800 -8.77280300 0.71563800
H 2.46591400 -9.07417000 1.43959200
H 2.07188800 -6.99849800 -2.29396800
H -0.32847200 -7.23553100 -2.17488600
H 0.04109100 -9.26725200 1.57592000
C -3.93369000 -7.43074200 -1.08874200
C -4.35493900 -6.80179800 0.08517500
C -3.53703800 -6.92192300 1.20834100
C -2.28558100 -7.51514600 1.12234100
C -1.80057200 -7.99564000 -0.09400900
C -2.68005100 -8.01150300 -1.17838400
H -4.57965000 -7.42865500 -1.96184400
H -3.83666100 -6.46020400 2.14454100
H -1.63477400 -7.50584600 1.99243100
H -2.36035400 -8.44568200 -2.12128800
C -6.27174600 -5.61503100 1.24292700
C -5.51107200 -5.86552500 0.09935800
C -5.74873000 -5.06556400 -1.01936000
C -6.62743600 -3.99638700 -0.96328600
C -7.29733900 -3.67458800 0.21726900
C -7.14448100 -4.53854700 1.30233600
H -6.15945800 -6.25378700 2.11419200
H -5.17857900 -5.23098600 -1.92861700
H -6.72903800 -3.34965500 -1.83044800
H -7.69396100 -4.35005300 2.22026300
C -7.94705800 -0.19596300 1.44685900
C -8.46299900 0.43815600 0.31605700
C -8.86711300 -0.37413700 -0.74682900
C -8.60942700 -1.73705100 -0.74177800
C -7.94609900 -2.34093500 0.32968900
C -7.69735800 -1.55831400 1.45583400
H -7.62306600 0.39811500 2.29540100
H -9.34374300 0.07001300 -1.61599800
H -8.88684100 -2.33338600 -1.60641800
H -7.19211800 -1.99600900 2.31152000
C -8.25423600 2.75952700 1.29468500
C -7.65311000 4.00494200 1.22129400
C -7.06188500 4.45414500 0.03696700
C -7.27886700 3.69036600 -1.10983300
C -7.88827000 2.44647400 -1.03787600
C -8.29416500 1.91164600 0.18499800
H -8.64657400 2.41862800 2.24827000
H -7.59603400 4.61435200 2.11826900
H -6.86476400 4.01148600 -2.06070600
H -7.92563300 1.83297600 -1.93308000
C 8.20618900 -2.66690800 -0.88549400
C 7.72918200 -3.96887900 -0.93492700
C 6.90297800 -4.47441400 0.07067700
C 6.67721400 -3.66292800 1.18350700
C 7.15683500 -2.36547600 1.23600800
C 7.88273700 -1.82139600 0.17635600
H 8.81810000 -2.29402400 -1.70202900
H 7.97867600 -4.58971700 -1.79044200
H 6.04120200 -4.01869800 1.98863400
H 6.88926400 -1.73675700 2.08023000
C 7.85762200 0.37110900 -1.01756000
C 8.10667900 -0.35169700 0.14854100
C 8.37206600 0.37118600 1.31311800
C 8.24504700 1.74955000 1.34260000
C 7.84663500 2.46026000 0.20859800
C 7.73201200 1.75291900 -0.98844100
H 7.66486800 -0.15888500 -1.94602600
H 8.64219300 -0.15791000 2.22246000
H 8.41668700 2.27899900 2.27526400
H 7.44234900 2.27627700 -1.89557200
C 5.75160100 5.32397900 1.40422600
C 5.71805900 6.18707900 0.30750700
C 6.61702900 5.92594300 -0.73070400
C 7.39771900 4.77808800 -0.73685600
C 7.32364000 3.84949900 0.30353700
C 6.53745100 4.18610600 1.40472600
H 5.07053400 5.47303400 2.23441700
H 6.68211500 6.60366800 -1.57654800
H 8.04914700 4.58612000 -1.58479700
H 6.45264600 3.49231600 2.23529500
C 4.06725700 7.48545000 -1.07278900
C 4.58058000 7.14331900 0.18077900
C 3.81900300 7.51041400 1.29260200
C 2.52178000 7.97166700 1.15185800
C 1.93991600 8.12668200 -0.10750100
C 2.77876300 7.98021800 -1.21389200
H 4.63709500 7.26885100 -1.97070600
H 4.20732800 7.35490200 2.29370900

H 1.92330400 8.11910600 2.04536700
H 2.39789600 8.17397500 -2.21223800
C -1.51969500 7.26321500 -1.29681500
C -2.32341100 7.66707300 -0.23144000
C -1.73153300 8.43512500 0.77179200
C -0.36965600 8.69617900 0.76312300
C 0.45802400 8.18383700 -0.23826000
C -0.15648200 7.51126500 -1.29631500
H -1.95325900 6.67416700 -2.10042000
H -2.34160800 8.81212700 1.58778900
H 0.05949600 9.27973900 1.57213100
H 0.45014200 7.09815400 -2.09651600
C -4.56387000 7.01559000 -1.20721700
C -5.71998800 6.25351900 -1.13601800
C -6.04942000 5.54613600 0.02296600
C -5.23945700 5.73958700 1.14389800
C -4.09264800 6.51462200 1.07842300
C -3.69984400 7.11666200 -0.11597000
H -4.31431800 7.52054900 -2.13613900
H -6.35883000 6.18383000 -2.01140600
H -5.45816300 5.20816000 2.06484800
H -3.44676300 6.58313700 1.94926400

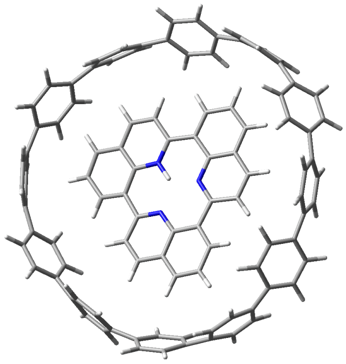

12CPP⊃TQ•H+
M06-2X/6-31G(d,p)

Zero-point correction=	1.343568 (Hartree/Particle)
Thermal correction to Energy=	1.417618
Thermal correction to Enthalpy=	1.418562
Thermal correction to Gibbs Free Energy=	1.234516
Sum of electronic and zero-point Energies=	-3972.414113
Sum of electronic and thermal Energies=	-3972.340064
Sum of electronic and thermal Enthalpies=	-3972.339120
Sum of electronic and thermal Free Energies=	-3972.523165

C 1.58147100 3.87615100 -0.05944500
C 1.05229500 2.59396600 -0.02411000
C 1.97498200 1.48907300 -0.05587500
C 3.36569300 1.74492000 -0.15678600
C 3.84879800 3.07166500 -0.21312800
C 2.96489100 4.11983400 -0.14600600
H 0.93770400 4.74793500 -0.04298500
C 4.22514500 0.61899400 -0.20654200
H 4.91805700 3.24383600 -0.30989700
H 3.31801500 5.14371200 -0.17316400
C 3.70908700 -0.64469500 -0.16518100
C 2.29516500 -0.82266500 -0.04419800
H 5.29931300 0.77361200 -0.28401400
H 4.38718900 -1.48608000 -0.23133900
C 1.69644400 -2.18703300 0.03727100
C 0.27628400 -2.44325400 -0.00090100
C 2.53339200 -3.28072200 0.17505300
C -0.18842400 -3.78527300 0.09381300
C 2.06346900 -4.60689300 0.27056100
H 3.60678400 -3.14544600 0.23828800
C -1.58723200 -4.00666800 0.05756200
C 0.71914500 -4.86179300 0.23711100
C -1.91479100 -1.64222400 -0.11616200
H 2.77959800 -5.41345600 0.38086800
C -2.45614500 -2.95629800 -0.03449900
H 0.33142200 -5.87355800 0.32782400
H -3.52295800 -3.14629900 -0.02731400
C -2.79145400 -0.43605400 -0.18039700
C -2.29215600 0.90674800 -0.10087300
C -4.16423400 -0.58031100 -0.30427800
C -3.19229700 1.99972500 -0.08534900
C -5.06115500 0.50594600 -0.30730900
H -4.59333900 -1.57143300 -0.40177500
C -2.65662900 3.30932100 -0.00373200
C -4.58566000 1.78312200 -0.16930300
C -0.41719000 2.41844600 -0.00108400
H -6.12504600 0.31830000 -0.41055900
C -1.30834800 3.52330100 0.03725400
H -3.34131000 4.15560900 0.01557300
H -5.25266300 2.63896100 -0.13752100
H -0.93742600 4.53786600 0.08871900
N -0.94738900 1.19043800 -0.04949700
N 1.49397100 0.22630700 0.00719400
N -0.61497700 -1.42570900 -0.11202900
H -1.96071100 -5.02634000 0.11882700
H -0.28323100 0.36613600 -0.05794100
C 3.35967400 -7.91759400 0.91270800
C 4.54392900 -7.20702100 1.05761500
C 5.14741900 -6.58732600 -0.04452900
C 4.64680900 -6.89913100 -1.31465800
C 3.48169400 -7.63835800 -1.46095200
C 2.75004300 -8.06188400 -0.34223900
H 2.85425600 -8.26666400 1.80675800
H 4.95384600 -7.05573100 2.05156000
H 5.11247200 -6.47016800 -2.19660500
H 3.09484500 -7.80819900 -2.45996400
C 0.56949200 -8.90452900 0.63115200
C 1.29661600 -8.36037700 -0.43915800
C 0.56338700 -7.83742100 -1.51284700
C -0.81735700 -7.72299900 -1.45321300
C -1.52419300 -8.11766400 -0.31145700
C -0.81221500 -8.77542800 0.70033400
H 1.09037900 -9.39155700 1.44918600
H 1.07747900 -7.39363900 -2.35760000
H -1.33889500 -7.20820700 -2.25437200
H -1.34207600 -9.15623300 1.56869100
C -4.91041200 -6.66639100 -1.11168800

C -5.21050600 -5.99619500 0.08257400
C -4.41431300 -6.26123400 1.20271100
C -3.28478300 -7.06494700 1.09880000
C -2.90857600 -7.61955500 -0.13096000
C -3.77867000 -7.46123400 -1.21783100
H -5.55594600 -6.53655900 -1.97510800
H -4.62316100 -5.75665600 2.14141400
H -2.62550800 -7.17014700 1.95574900
H -3.54377400 -7.93795800 -2.16483200
C -6.93754200 -4.58178700 1.27500800
C -6.21062400 -4.90257100 0.12089900
C -6.32786100 -4.05305900 -0.98680500
C -7.07457500 -2.88560700 -0.91457200
C -7.72499900 -2.51809600 0.27054300
C -7.67915100 -3.40854400 1.35009800
H -6.90668400 -5.25086300 2.12985400
H -5.76469900 -4.26959800 -1.88976300
H -7.08285200 -2.20500700 -1.76149400
H -8.21355200 -3.16872400 2.26477200
C -7.99311300 1.00609700 1.50547300
C -8.37530200 1.68944100 0.34379200
C -8.86197300 0.92961500 -0.73028300
C -8.78622000 -0.45634400 -0.71041500
C -8.23235200 -1.13004600 0.38812100
C -7.92771200 -0.38134700 1.52984200
H -7.61219800 1.56336000 2.35534400
H -9.24652300 1.42997300 -1.61396700
H -9.10846000 -1.02372700 -1.57860200
H -7.50528100 -0.87761500 2.39838300
C -7.79519300 3.96795800 1.27354000
C -6.98901900 5.09333400 1.17442200
C -6.31893000 5.39945400 -0.02000500
C -6.64172500 4.64284000 -1.15326300
C -7.45458200 3.52100200 -1.05590500
C -7.96667800 3.10686500 0.18014100
H -8.25289300 3.72392200 2.22749900
H -6.83901200 5.71573500 2.05119100
H -6.15549700 4.85241000 -2.10058200
H -7.57137100 2.88381100 -1.92693800
C 7.64826700 -3.83553700 -0.79287700
C 7.01563000 -5.07241400 -0.84766500
C 6.07889600 -5.44596300 0.12505400
C 5.87263100 -4.57632600 1.20460200
C 6.51007200 -3.34581500 1.26439600
C 7.37479300 -2.93401600 0.24314700
H 8.35239600 -3.55699600 -1.57164000
H 7.23346200 -5.74710300 -1.67026800
H 5.13552000 -4.82661900 1.96187200
H 6.27153100 -2.65858700 2.07111400
C 7.74805100 -0.77493400 -0.95599100
C 7.82203900 -1.52121000 0.22530100
C 8.14985000 -0.85008300 1.41083400
C 8.26950900 0.53129500 1.43674100
C 8.05756300 1.29270600 0.27924700
C 7.86385800 0.61079200 -0.92899100
H 7.50130500 -1.27378200 -1.88891500
H 8.28014100 -1.41890100 2.32671700
H 8.49412900 1.03160900 2.37382200
H 7.69626200 1.17537200 -1.84175400
C 6.51180600 4.51048600 1.41975300
C 6.62137000 5.32935400 0.28792600
C 7.46063600 4.89012100 -0.74719800
C 8.03122300 3.62318200 -0.71853600
C 7.79516400 2.75031200 0.35290000
C 7.08722600 3.24940700 1.45266100
H 5.86132600 4.80065400 2.23734300
H 7.63412100 5.52595900 -1.60985300
H 8.63830000 3.29295800 -1.55637400
H 6.88060900 2.60005800 2.29746600
C 5.16339000 6.75232800 -1.16585800
C 5.64525000 6.43578700 0.11249600
C 4.96432800 6.98418000 1.20868300
C 3.73599400 7.60826200 1.04453700
C 3.15954900 7.74432600 -0.22552200
C 3.95123100 7.40947600 -1.33222000
H 5.68031200 6.38443000 -2.04609600
H 5.35020300 6.84185500 2.21269500
H 3.17229500 7.89529900 1.92659000
H 3.57489500 7.57912500 -2.33625300
C -0.38197800 7.20229200 -1.35973300
C -1.12682200 7.79071900 -0.33127900
C -0.44414100 8.54297700 0.63231700
C 0.94231900 8.63464900 0.61362900
C 1.69689600 7.95836600 -0.35490100
C 1.00427200 7.27742900 -1.36587200
H -0.89188900 6.61141400 -2.11604600
H -1.00726100 9.04782600 1.41194000
H 1.44692800 9.21683800 1.37867100
H 1.55835200 6.72449900 -2.11825100
C -3.41121700 7.43019800 -1.33131100
C -4.68111700 6.87543700 -1.24920500
C -5.14507000 6.30765200 -0.05365300
C -4.33718300 6.43440800 1.08511400
C -3.07422600 7.00848200 1.00863100
C -2.56714700 7.46144700 -0.21412800
H -3.05744200 7.82926100 -2.27750200
H -5.30952200 6.85687200 -2.13409900
H -4.65895500 5.99679200 2.02481500
H -2.43820400 7.02653900 1.88922500

Supplementary Table 30 Cartesian coordinates and energies of **[12]CPP**.

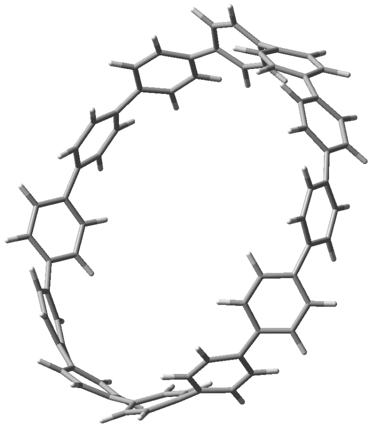

[12]CPP
B3LYP-D3/6-31G(d,p)
Zero-point correction= 0.971066 (Hartree/Particle)
Thermal correction to Energy= 1.024564
Thermal correction to Enthalpy= 1.025508
Thermal correction to Gibbs Free Energy= 0.880690
Sum of electronic and zero-point Energies= -2771.834341
Sum of electronic and thermal Energies= -2771.780843
Sum of electronic and thermal Enthalpies= -2771.779899
Sum of electronic and thermal Free Energies= -2771.924717
C 2.79296600 -7.80145700 1.01852200
C 4.06743700 -7.26950600 1.19137600
C 4.80978900 -6.78277400 0.09984600
C 4.28945600 -7.03067500 -1.18380500
C 3.02754300 -7.58440500 -1.35790100
C 2.20952000 -7.89486700 -0.25727600
H 2.20664400 -8.05755700 1.89584600
H 4.45936800 -7.16559400 2.19847500
H 4.83502900 -6.69729400 -2.06040900
H 2.64197600 -7.71007700 -2.36500600
C -0.05652800 -8.79647700 0.48217500
C 0.74533000 -8.06836800 -0.41511600
C 0.08296800 -7.31117000 -1.39751000
C -1.29892700 -7.19754100 -1.41337900
C -2.09821400 -7.83544500 -0.44837000
C -1.44581000 -8.68345700 0.46514600
H 0.41385100 -9.43785300 1.22203400
H 0.66131400 -6.71369700 -2.09408800
H -1.75530200 -6.51418700 -2.12140600
H -2.03060800 -9.23974200 1.19209100
C -5.39993000 -6.19806700 -1.26094000
C -5.89387200 -5.88774300 0.01903200
C -5.27061600 -6.51737300 1.11199300
C -4.10307300 -7.25621900 0.94980400
C -3.51652100 -7.42224900 -0.31720600
C -4.24758900 -6.95722100 -1.42507000
H -5.86396800 -5.75875100 -2.13815600
H -5.66100300 -6.36674000 2.11377200
H -3.58857200 -7.62528300 1.83179000
H -3.86893100 -7.12841600 -2.42805300
C -6.71391600 -3.92582700 1.32055100
C -6.84554700 -4.76324700 0.19842800
C -7.73530000 -4.34641500 -0.80962700
C -8.33881300 -3.09196600 -0.76769600
C -8.09184500 -2.20202900 0.29218700
C -7.32507900 -2.68011500 1.36854100
H -6.03020400 -4.19778000 2.11756700
H -7.92400300 -4.99345600 -1.66112700
H -8.97888100 -2.78116800 -1.58860700
H -7.10437400 -2.01961600 2.20102000
C -8.50661900 1.39866600 1.35956800
C -8.10955800 2.07863500 0.19385000
C -8.02102700 1.33085400 -0.99246200
C -8.15518900 -0.05426700 -0.97938000
C -8.38318100 -0.74965300 0.22042500
C -8.64014300 0.01466200 1.37279200
H -8.65950600 1.95775700 2.27811800
H -7.71939400 1.82331400 -1.91194800
H -7.95492600 -0.61230000 -1.88897700
H -8.89459800 -0.48789600 2.30147500
C -6.71445800 3.79452000 1.31986200
C -5.89588400 4.91388200 1.26851200
C -5.87528100 5.75746300 0.14307000
C -6.80947500 5.48749100 -0.87457300
C -7.62261200 4.35711000 -0.82920100
C -7.54948800 3.45089600 0.24311100
H -6.61896500 3.11339700 2.15938200
H -5.18294700 5.06891300 2.07092000
H -6.87642300 6.14988000 -1.73252800
H -8.29992700 4.15528700 -1.65425300
C 7.67858100 -4.45615500 -0.74397100
C 6.87973900 -5.59554700 -0.75429500
C 5.94481500 -5.84088900 0.26945600
C 5.97788800 -4.98261700 1.38359100
C 6.79086900 -3.85533800 1.40451700
C 7.59695500 -3.52266800 0.30352500
H 8.34235900 -4.26085400 -1.58150000
H 6.94717200 -6.27731300 -1.59662500
H 5.27360400 -5.12736200 2.19592500
H 6.70560500 -3.15632900 2.23104000
C 7.84470800 -1.43529000 -1.01602300
C 8.09843500 -2.13328000 0.17608600
C 8.60095100 -1.39510100 1.26079500
C 8.71797900 -0.00931700 1.19510700
C 8.31092300 0.70784100 0.05319600
C 7.93845100 -0.05087300 -1.07206200
H 7.45226800 -1.97341800 -1.87344800
H 8.86415500 -1.91027500 2.18036600
H 9.08787000 0.52425300 2.06501100
H 7.60821800 0.44774000 -1.97640300
C 7.19932600 4.10119600 1.29997900
C 6.81715000 4.75078000 0.11153000
C 7.23840600 4.15200600 -1.09031200
C 7.85759600 2.90809200 -1.10637500
C 8.05650200 2.17088700 0.07558700
C 7.78339600 2.84019100 1.28279700
H 6.97540600 4.55253200 2.26088000

H 7.00389800 4.61964800 -2.04056500
H 8.12602600 2.47721400 -2.06534600
H 7.94583400 2.33687200 2.23001300
C 5.58248000 6.64614900 -1.03822000
C 5.83601400 5.86630100 0.10715000
C 4.96363100 6.04124300 1.19771000
C 3.83043400 6.83875600 1.10141100
C 3.49790500 7.48580900 -0.09974600
C 4.43340000 7.42444000 -1.14592300
H 6.26659300 6.60963600 -1.88002200
H 5.10562200 5.46305000 2.10377200
H 3.12933000 6.86479300 1.93010900
H 4.23629200 7.96149100 -2.06950800
C 0.00303100 7.64061200 -1.45541400
C -0.72996900 8.18405300 -0.38421400
C 0.01061700 8.75701600 0.66775000
C 1.39640700 8.64069800 0.72415400
C 2.10979500 7.97224900 -0.28493200
C 1.38874300 7.54672800 -1.41239500
H -0.51756400 7.18023000 -2.28797000
H -0.50526700 9.25934900 1.48009200
H 1.93186900 9.03632400 1.58264900
H 1.90964800 7.02566000 -2.21000800
C -2.96949100 7.60653000 -1.41186600
C -4.21459400 7.00144100 -1.27788200
C -4.76360200 6.72768300 -0.01224500
C -4.07072000 7.23652800 1.10101800
C -2.81254800 7.81066700 0.96870700
C -2.19163600 7.93951100 -0.28753100
H -2.56928800 7.75637800 -2.40950500
H -4.72330000 6.65482300 -2.17186800
H -4.48951100 7.12002400 2.09559900
H -2.27098600 8.07853200 1.86974300

Supplementary Table 31 Cartesian coordinates and energies of **[12]CPP⊃(TQ•H⁺)₂**.

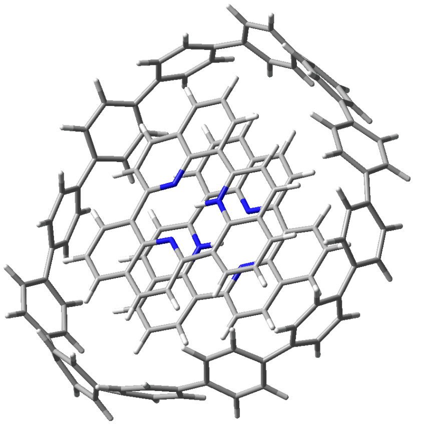

[12]CPP⊃(TQ•H⁺)₂
B3LYP-D3/6-31G(d,p)
Zero-point correction= 1.697161 (Hartree/Particle)
Thermal correction to Energy= 1.792102
Thermal correction to Enthalpy= 1.793046
Thermal correction to Gibbs Free Energy= 1.568880
Sum of electronic and zero-point Energies= -5176.653389
Sum of electronic and thermal Energies= -5176.558448
Sum of electronic and thermal Enthalpies= -5176.557504
Sum of electronic and thermal Free Energies= -5176.781669

C 3.26792200 -2.71909900 1.61985700
C 2.18543700 -1.83894200 1.65340100
C 2.47804500 -0.42611600 1.70263000
C 3.83603600 0.00963900 1.68250500
C 4.88670200 -0.93173100 1.63157700
C 4.60164900 -2.27836200 1.61120100
H 3.11254000 -3.78847600 1.57681800
C 4.06159100 1.40889200 1.67873700
H 5.90975200 -0.57932700 1.58687800
H 5.40404700 -3.00409300 1.55777400
C 3.00568600 2.28077300 1.72549300
C 1.66885000 1.77257500 1.78483500
H 5.07855200 1.78564100 1.61772100
H 3.21233500 3.34171000 1.69755500
C 0.49416300 2.68630100 1.85326100
C -0.88220600 2.23773300 1.81437400
C 0.71008400 4.06073300 1.93835600
C -1.93302800 3.21150800 1.83290900
C -0.32967200 5.00791500 1.95675900
H 1.71853800 4.45054600 1.98283300
C -3.26997800 2.74337300 1.75460600
C -1.63781700 4.59209300 1.89614400
C -2.44224600 0.48730800 1.70431900
H -0.09017600 6.06466800 1.99061500
C -3.53710900 1.40110000 1.69107100
H -2.45203000 5.30601000 1.87474300
H -4.56366400 1.06814400 1.61351100
C -2.64363200 -0.98676600 1.64943500
C -1.56443900 -1.93448600 1.61078500
C -3.93357700 -1.51328000 1.61098800
C -1.83888700 -3.32936600 1.51538400
C -4.20719800 -2.88912400 1.52287800
H -4.78612300 -0.84772600 1.62623800
C -0.74187400 -4.22410300 1.44220400
C -3.17287500 -3.79164700 1.46662700
C 0.81353700 -2.38004000 1.60554100
H -5.23434800 -3.23034200 1.47059700
C 0.55028400 -3.77061000 1.48806000
H -0.93651700 -5.28779800 1.33496100
H -3.35811200 -4.85392400 1.36548300
H 1.35962300 -4.48168400 1.41177600
N -0.24501000 -1.54696900 1.65144000
N 1.45794800 0.46028700 1.75546700
N -1.18835100 0.91701400 1.75036400
H -4.08267400 3.46409700 1.72953300
H -0.05521500 -0.50798600 1.71006300
C -1.47553700 8.43893500 -1.30764800
C -0.09026600 8.43865900 -1.41801500
C 0.73442000 8.39172200 -0.27804900
C 0.09736800 8.59063000 0.96216000
C -1.28784200 8.62052200 1.07063000
C -2.11336300 8.42099000 -0.05233100

H -2.05988500 8.35817700 -2.21765400
H 0.34580500 8.40896800 -2.41065300
H 0.68261100 8.64121700 1.87419200
H -1.72454800 8.72831400 2.05771000
C -4.36380500 7.78767000 -1.03515000
C -3.50594300 7.92454200 0.07433700
C -3.92112000 7.30674600 1.26892700
C -4.98674100 6.41660400 1.29153000
C -5.70161000 6.11038300 0.12126500
C -5.43538100 6.90037200 -1.01297300
H -4.16437200 8.33699100 -1.94916800
H -3.33993300 7.42971600 2.17581700
H -5.19172200 5.87454500 2.20944700
H -6.04136900 6.78259300 -1.90603400
C -7.58994300 2.99696800 1.17655800
C -7.47574700 2.18976500 0.03032200
C -6.95882700 2.78590300 -1.13332200
C -6.48350600 4.09250600 -1.12179200
C -6.49832200 4.86459600 0.05423800
C -7.11394600 4.30363100 1.18809800
H -8.05823100 2.59704000 2.07043100
H -6.86978700 2.20116600 -2.04433300
H -6.02612000 4.49105400 -2.02186900
H -7.21614700 4.89440700 2.09300800
C -8.20486000 0.04051800 -1.07670600
C -7.76368400 0.73873700 0.06173400
C -7.48687400 -0.01419000 1.21621400
C -7.54664000 -1.40302700 1.20090000
C -7.88218700 -2.10738900 0.02998300
C -8.26466700 -1.34898900 -1.09177800
H -8.50576200 0.59212800 -1.96184400
H -7.16030300 0.48850500 2.12200800
H -7.25715900 -1.94764100 2.09392300
H -8.60696200 -1.85184100 -1.99076900
C -6.46543600 -5.34094600 -1.24354600
C -6.26925100 -6.06536300 -0.05389300
C -6.96724100 -5.61864500 1.08474600
C -7.62790300 -4.39422500 1.08680000
C -7.62037700 -3.56283300 -0.04973800
C -7.12019900 -4.11541400 -1.24113400
H -5.99312600 -5.67183200 -2.16245300
H -6.94742500 -6.20549200 1.99761200
H -8.11136700 -4.05681500 1.99841200
H -7.13690200 -3.53395200 -2.15756400
C -4.57632700 -7.62570900 -1.11340700
C -3.26147200 -8.07888900 -1.11047100
C -2.45272400 -7.95463200 0.03544000
C -3.08784700 -7.56345900 1.22615700
C -4.40322100 -7.11315500 1.22308700
C -5.13999500 -7.02326500 0.02823100
H -5.15161900 -7.69110100 -2.03146000
H -2.84311900 -8.49210800 -2.02319800
H -2.51902100 -7.49893100 2.14851500
H -4.81148800 -6.70691500 2.14255400
C 4.19043700 7.22251900 0.77410100
C 3.01904800 7.97138200 0.74535600
C 2.13774400 7.91790600 -0.35172900
C 2.57846200 7.18968300 -1.47314300
C 3.74107400 6.43153100 -1.43996600
C 4.54800500 6.37178700 -0.28985800
H 4.82898400 7.29525400 1.64813500
H 2.78212500 8.59121600 1.60354400
H 1.96687200 7.14277600 -2.36724900
H 3.97993700 5.82699100 -2.30824600
C 6.09879500 4.92378800 1.07038800
C 5.63602200 5.37231300 -0.18099800
C 6.17515800 4.73936100 -1.31693700
C 7.03582100 3.65475500 -1.20463600
C 7.43937700 3.15607600 0.04894600
C 6.98844600 3.86094400 1.18124400
H 5.73555600 5.38712700 1.98158500
H 5.91485800 5.08819800 -2.31044000
H 7.36690500 3.16905500 -2.11587200
H 7.31450000 3.56640600 2.17298500
C 8.98857600 -0.14080100 -0.96920900
C 8.69227300 -0.94275700 0.14909700
C 8.34737300 -0.26981900 1.33460800
C 8.06639900 1.09123600 1.33494200
C 8.13757100 1.85242700 0.15447900
C 8.72401300 1.22397000 -0.96351000
H 9.37958600 -0.59616000 -1.87369800
H 8.17068800 -0.83466900 2.24420300
H 7.68779900 1.53208200 2.25068900
H 8.93260400 1.79505100 -1.86188300
C 8.36732700 -3.24668400 1.15928800
C 8.42094000 -2.39545600 0.03910500
C 7.89659800 -2.89574600 -1.16662100
C 7.15459100 -4.06998800 -1.19642200
C 6.91463200 -4.80675500 -0.02370100
C 7.63214700 -4.42764200 1.12748300
H 8.85767800 -2.95686500 2.08339000
H 7.95698500 -2.29832300 -2.07039800
H 6.66304400 -4.34897500 -2.12299700
H 7.57209200 -5.03524300 2.02513600
C 3.76469900 -6.48855200 1.18986400
C 3.23804000 -7.04762500 0.01257000
C 4.05909500 -7.04305900 -1.12950200
C 5.29995200 -6.41513600 -1.12256500
C 5.77809600 -5.75555700 0.02522100
C 5.00498900 -5.86003500 1.19616200
H 3.17132100 -6.48954500 2.09970200
H 3.72291900 -7.53894900 -2.03469200
H 5.90250300 -6.43156000 -2.02518700
H 5.34033000 -5.37965100 2.10953200
C 1.18961100 -8.05325400 1.09228900
C -0.18023100 -8.29438400 1.09392700
C -0.97411100 -7.99878900 -0.02961000
C -0.31236100 -7.54809000 -1.18680400
C 1.05657700 -7.30703300 -1.18866000
C 1.83263700 -7.50794600 -0.03359000

H 1.76996100 -8.29681600 1.97666000
H -0.64000200 -8.71654000 1.98194600
H -0.88433200 -7.31932900 -2.08008900
H 1.51905400 -6.90323400 -2.08470400
C 4.02056600 1.20775300 -1.62685300
C 2.67812100 0.82238300 -1.63272100
C 2.39154400 -0.59186800 -1.67987200
C 3.46900200 -1.52730300 -1.68288200
C 4.80677000 -1.07385700 -1.65837100
C 5.07453600 0.27757900 -1.64143500
H 4.29934000 2.25349100 -1.59536900
C 3.12256900 -2.90279300 -1.67493000
H 5.61066300 -1.80045900 -1.63102100
H 6.09867000 0.63322200 -1.60912700
C 1.80692700 -3.28702600 -1.69816800
C 0.77737300 -2.29246100 -1.73867300
H 3.90943600 -3.65130200 -1.63317500
H 1.57513800 -4.34268800 -1.66528400
C -0.66430300 -2.66914000 -1.79029000
C -1.75330600 -1.71530200 -1.75030200
C -1.00862300 -4.01781700 -1.86072100
C -3.10329300 -2.19628000 -1.76199800
C -2.33792700 -4.47866300 -1.87343600
H -0.23740900 -4.77507400 -1.88964100
C -4.14803600 -1.24066400 -1.67559200
C -3.37659700 -3.58124700 -1.82013000
C -2.49913800 0.50671600 -1.63422400
H -2.53658000 -5.54420600 -1.89671300
C -3.86495500 0.09829900 -1.60972000
H -4.40629700 -3.91709200 -1.79571700
H -4.67633300 0.80892000 -1.52278700
C -2.10297400 1.93971700 -1.58088500
C -0.73753400 2.38301000 -1.54719100
C -3.07892100 2.93389900 -1.53888700
C -0.44198900 3.77222800 -1.44618300
C -2.78842600 4.30720100 -1.45323500
H -4.12477700 2.65826400 -1.55232100
C 0.91705100 4.15701000 -1.37053200
C -1.48177900 4.72887000 -1.39727000
C 1.62619100 1.85647500 -1.56312700
H -3.59886400 5.02481800 -1.40674200
C 1.92893000 3.23604500 -1.43290300
H 1.15794000 5.20699400 -1.24985400
H -1.23130700 5.78086400 -1.30679600
H 2.95110000 3.57688400 -1.35777900
N 0.32383500 1.50804600 -1.59996800
N 1.10305000 -1.00398300 -1.71103800
N -1.51508700 -0.38117100 -1.68862400
H -5.17881800 -1.58340900 -1.64492200
H 0.08865600 0.47902500 -1.66053000

Supplementary Table 32 Cartesian coordinates and energies of **TQ•H⁺/coronene**.

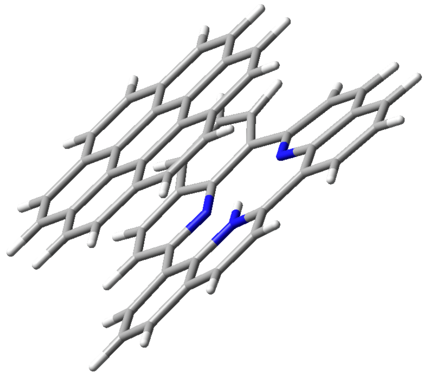

TQ•H⁺/coronene
B3LYP-D3/6-31G(d,p)

Zero-point correction=	0.641222 (Hartree/Particle)
Thermal correction to Energy=	0.676669
Thermal correction to Enthalpy=	0.677613
Thermal correction to Gibbs Free Energy=	0.576726
Sum of electronic and zero-point Energies=	-2124.071609
Sum of electronic and thermal Energies=	-2124.036162
Sum of electronic and thermal Enthalpies=	-2124.035218
Sum of electronic and thermal Free Energies=	-2124.136105

C 0.65870000 3.83472600 -1.67438900
C 0.11923700 2.55968900 -1.51732300
C -1.28286800 2.46855200 -1.19721400
C -2.06232300 3.66012100 -1.09682600
C -1.45673100 4.92526700 -1.26965600
C -0.11035200 5.00542800 -1.54647200
H 1.70693200 3.96119100 -1.90895900
C -3.44846700 3.49631300 -0.84963800
H -2.06410900 5.82200300 -1.18741700
H 0.36814600 5.96951600 -1.67949900
C -3.98007900 2.24439100 -0.68875200
C -3.12244800 1.10136800 -0.73158000
H -4.08626300 4.37460400 -0.80684700
H -5.04697600 2.13848600 -0.54797900
C -3.65406300 -0.26632600 -0.48257700
C -2.91024400 -1.47606000 -0.74536000
C -4.92662000 -0.40893600 0.06265900
C -3.53175000 -2.74317200 -0.50306600
C -5.52482300 -1.66006600 0.31126700
H -5.49706700 0.47006900 0.33456000
C -2.76677600 -3.90797700 -0.76014400
C -4.84524600 -2.81771500 0.01621500
C -0.90693700 -2.51435100 -1.37406100
H -6.52292600 -1.69714600 0.73450500
C -1.46738300 -3.81023700 -1.17950700
H -5.29207100 -3.79150000 0.19415200
H -0.88983600 -4.71099400 -1.33457400
C 0.51572400 -2.32136100 -1.76691400
C 1.14562400 -1.03355900 -1.85229200
C 1.32019500 -3.42349200 -2.04421300
C 2.52786700 -0.93279300 -2.17953800
C 2.68302600 -3.32749300 -2.37745300
H 0.89185800 -4.41595400 -2.00632700
C 3.10693400 0.35772200 -2.22511900

C 3.28917500 -2.09629100 -2.43391500
C 0.98009100 1.37452100 -1.70746300
H 3.24698700 -4.23189700 -2.57724600
C 2.36000700 1.48388500 -2.00548500
H 4.16718100 0.44609300 -2.43832600
H 4.34278700 -1.99475000 -2.67106800
H 2.84185300 2.44814200 -2.02718000
N 0.45576100 0.13518800 -1.63117400
N -1.83130700 1.25037300 -0.99642400
N -1.62858600 -1.42034000 -1.18428600
H -3.21910300 -4.88175500 -0.59508200
H -0.56639300 0.04747200 -1.37213500
C 2.28760600 -3.58789400 1.29381000
C 2.88729700 -2.30343600 1.16137400
C 2.08718100 -1.14876000 1.37852800
C 0.71036800 -1.28939900 1.72653200
C 0.14121400 -2.58397100 1.86186700
C 0.96432400 -3.72284800 1.63338000
C 2.66102300 0.15137800 1.25027200
C -0.09289200 -0.13040800 1.94326700
C 0.48004800 1.16956200 1.81492800
C 1.85654000 1.31064400 1.46618300
C -0.31890800 2.32378200 2.03630600
C -1.68918400 2.15298400 2.38251500
C -2.23893300 0.90195800 2.50951300
C -1.46123000 -0.27103400 2.29835600
C -2.00510400 -1.58021700 2.42871600
C -1.23323100 -2.69414700 2.21821300
H -1.66694500 -3.68461400 2.31862600
H -3.05439400 -1.68495800 2.68901200
H 2.90200100 -4.46868100 1.12972900
H 0.52605500 -4.71131100 1.73991600
H -2.29877800 3.03673900 2.54762400
H -3.28599900 0.78971800 2.77568600
C 4.25851900 -2.13240100 0.81837300
C 4.81160900 -0.88200700 0.69578500
C 4.03278700 0.29163200 0.90434100
H 4.86921700 -3.01665800 0.65938400
H 5.86246500 -0.77243100 0.44206400
C 4.57618600 1.60245400 0.77290700
C 3.80189100 2.71714200 0.97724800
C 2.42658700 2.60655400 1.33384100
H 5.62647900 1.70778600 0.51499800
H 4.23775500 3.70823300 0.88408200
C 0.28035700 3.60848100 1.90308800
H -0.33261600 4.48917000 2.07134900
C 1.60320800 3.74493800 1.56458700
H 2.04229800 4.73404700 1.46945600

Supplementary Table 33 Cartesian coordinates and energies of **[12]CPP⊃(TQ•H+/coronene)**.

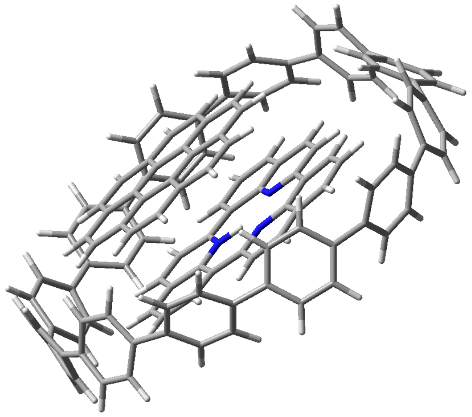

[12]CPP⊃(TQ•H+/coronene)
B3LYP-D3/6-31G(d,p)

Zero-point correction=	1.616746 (Hartree/Particle)
Thermal correction to Energy=	1.706687
Thermal correction to Enthalpy=	1.707632
Thermal correction to Gibbs Free Energy=	1.493164
Sum of electronic and zero-point Energies=	-4896.011106
Sum of electronic and thermal Energies=	-4895.921165
Sum of electronic and thermal Enthalpies=	-4895.920220
Sum of electronic and thermal Free Energies=	-4896.134687

C -2.33535400 3.16381600 -1.59671900
C -1.42974600 2.12241800 -1.39875400
C -0.04596900 2.47763400 -1.20729700
C 0.33576800 3.85347100 -1.21200600
C -0.63018300 4.86411700 -1.41260600
C -1.94735600 4.51388000 -1.60940700
H -3.38943300 2.95723300 -1.72558200
C 1.70845700 4.13509000 -0.99854600
H -0.32946600 5.90752900 -1.39098500
H -2.70440900 5.27473500 -1.74676700
C 2.60471200 3.11848200 -0.80707300
C 2.14854300 1.76140300 -0.80944500
H 2.04140300 5.16716500 -0.98367300
H 3.64428000 3.37056200 -0.64734400
C 3.08973300 0.62959400 -0.57966100
C 2.70502800 -0.76489400 -0.62120300
C 4.42181500 0.90845500 -0.28420500
C 3.70254400 -1.77154300 -0.40426100
C 5.38827100 -0.08784400 -0.04904400
H 4.76400200 1.93294800 -0.21606100
C 3.29628800 -3.12838100 -0.47000200
C 5.04119900 -1.41602700 -0.11548300
C 1.03848600 -2.39933200 -0.85222200
H 6.40492800 0.20756000 0.17571600
C 1.98313300 -3.45385600 -0.68607800
H 5.77746900 -2.19554600 0.04979100
H 1.69575200 -4.49663100 -0.71909400
C -0.41556500 -2.66505300 -1.03488400
C -1.39639100 -1.62702500 -1.19970900
C -0.89427600 -3.97278700 -1.01943200
C -2.77535200 -1.95925000 -1.33796200
C -2.25515300 -4.30452500 -1.15766400

H -0.20362000 -4.79404000 -0.87720500
C -3.70369100 -0.89869000 -1.47070600
C -3.19201200 -3.31123600 -1.31029800
C -1.91999600 0.72862200 -1.37203500
H -2.55217600 -5.34595700 -1.12200500
C -3.29506200 0.40925200 -1.49640600
H -4.76196700 -1.13451000 -1.53800900
H -4.24864400 -3.54386100 -1.39255200
H -4.03859900 1.18877200 -1.55579500
N -1.05389800 -0.29473000 -1.22649000
N 0.86348800 1.49571800 -1.01126600
N 1.41604800 -1.12854400 -0.83797000
H 4.03823700 -3.90856800 -0.33259900
H -0.02937100 -0.06202900 -1.09247500
C 8.95462200 0.38166400 0.64349300
C 8.62850300 1.72224800 0.81088100
C 8.24670700 2.51679100 -0.28434100
C 8.42308800 1.96948900 -1.56650800
C 8.77279700 0.63439600 -1.73407300
C 8.92961900 -0.22259400 -0.62833200
H 9.11788400 -0.22211100 1.52940200
H 8.59336900 2.13649300 1.81371300
H 8.18362400 2.56424000 -2.44265100
H 8.83453200 0.23694300 -2.74150700
C 9.02152800 -2.57452100 0.31581700
C 8.75145700 -1.69204400 -0.74876800
C 8.02566600 -2.21144700 -1.83618800
C 7.45075500 -3.47406800 -1.78469800
C 7.56103100 -4.27419200 -0.63531700
C 8.42927200 -3.83171800 0.37832000
H 9.65422100 -2.25548800 1.13772700
H 7.78892900 -1.57879800 -2.68352200
H 6.79459000 -3.78245400 -2.59221700
H 8.60482800 -4.46030500 1.24637100
C 4.93898800 -6.89114500 -1.38299000
C 4.20493900 -6.90005300 -0.18263900
C 4.76697900 -6.24570300 0.92675400
C 5.93489800 -5.49955400 0.80305000
C 6.58600500 -5.37096300 -0.43685900
C 6.10220400 -6.14151300 -1.50863800
H 4.57673900 -7.45442500 -2.23733900
H 4.23156000 -6.23145100 1.87094300
H 6.28325300 -4.91933400 1.65187900
H 6.62422600 -6.12193200 -2.46043100
C 2.23613400 -7.90694700 1.05250600
C 2.81492100 -7.40809100 -0.12812900
C 1.97790300 -7.25466600 -1.24788300
C 0.61405700 -7.50911500 -1.16727700
C 0.02194700 -7.92066100 0.03952300
C 0.86894700 -8.15320100 1.13646100
H 2.86295100 -8.09360400 1.91908800
H 2.38452200 -6.85601500 -2.17176700
H -0.01398600 -7.30759200 -2.02978200
H 0.44949400 -8.52166500 2.06791500
C -3.39453400 -6.94808700 1.26187100
C -4.19230800 -7.13426600 0.11753800
C -3.63271100 -7.89119400 -0.93075200
C -2.29083300 -8.25549100 -0.91901300
C -1.45312200 -7.90436100 0.15440000
C -2.05897800 -7.33047700 1.28302000
H -3.78276500 -6.39747400 2.11132200
H -4.23522200 -8.15171500 -1.79454700
H -1.87519100 -8.78418400 -1.77145000
H -1.45004700 -7.07597900 2.14496500
C -6.05792400 -5.67519200 1.00702200
C -6.88514100 -4.57845700 0.79701300
C -7.15437200 -4.09576700 -0.49800600
C -6.70520500 -4.89441900 -1.56680500
C -5.84905700 -5.96744300 -1.35774300
C -5.42310200 -6.32759600 -0.06567200
H -5.84711400 -5.97476300 2.02833600
H -7.25360600 -4.04682500 1.66714900
H -6.96213700 -4.63761700 -2.58838900
H -5.43574000 -6.46714600 -2.22674500
C 6.55267200 5.86911300 -0.91028300
C 7.47939200 4.83270000 -1.00527700
C 7.47003300 3.76306200 -0.09305600
C 6.53843500 3.81507800 0.96013000
C 5.61663100 4.84786800 1.05592000
C 5.57995100 5.88202700 0.10372200
H 6.57820700 6.67565200 -1.63715400
H 8.21297900 4.84740900 -1.80571700
H 6.48216000 2.99357000 1.66697300
H 4.86278000 4.81131800 1.83602900
C 3.76013400 7.17392600 -1.04837900
C 4.43500700 6.82074900 0.13165200
C 3.86375300 7.22278200 1.35275600
C 2.59761100 7.78898000 1.39887500
C 1.84224800 7.97220800 0.22596100
C 2.48603300 7.73786300 -1.00176900
H 4.18259400 6.89845100 -2.01009700
H 4.40516800 7.05158000 2.27802500
H 2.16934500 8.05347800 2.36061300
H 1.94298400 7.88714300 -1.93021900
C -1.71653600 7.35161700 1.24437700
C -2.45979100 7.84894400 0.15880200
C -1.76399500 8.61975400 -0.79176900
C -0.37923600 8.76412100 -0.73359900
C 0.37329400 8.15254600 0.28505600
C -0.33978300 7.50162200 1.30697000
H -2.20018700 6.74451600 2.00094800
H -2.30434200 9.08645800 -1.60948700
H 0.12656900 9.33643000 -1.50550500
H 0.20002400 7.01233000 2.11084800
C -4.32278900 7.13421800 -1.36274700
C -3.83470700 7.33769700 -0.05853800
C -4.58692900 6.78707700 0.99606500
C -5.61925300 5.88928700 0.74956800
C -5.96116800 5.52287400 -0.56383400
C -5.36517000 6.24742000 -1.61029600

H -3.81262700 7.58473700 -2.20813700
H -4.31217200 6.99642400 2.02458300
H -6.09574700 5.39215500 1.58907600
H -5.66828200 6.05906300 -2.63576300
C -6.72113800 2.11270700 -1.94223200
C -7.64381000 1.58262600 -1.02079300
C -8.16708300 2.46915600 -0.06131000
C -7.70548300 3.77872700 0.04492600
C -6.70842800 4.27074800 -0.81450900
C -6.27176300 3.42133900 -1.84675800
H -6.28462400 1.47462800 -2.70266200
H -8.93199400 2.12921400 0.62926700
H -8.11259200 4.42279000 0.81847400
H -5.50729100 3.76540400 -2.53620900
C -7.57632300 -0.69610200 -2.08643400
C -7.44888100 -2.07129700 -1.95294100
C -7.65697000 -2.71782300 -0.71854900
C -8.15614400 -1.91950000 0.32736800
C -8.25332800 -0.53734400 0.20258600
C -7.88647500 0.12188000 -0.98467600
H -7.38296700 -0.25130900 -3.05670800
H -7.11498800 -2.63481300 -2.81646900
H -8.41264600 -2.36730900 1.28135900
H -8.54579600 0.03789200 1.07490900
C -0.16786400 -3.55798800 2.46415300
C 0.38672500 -2.24698000 2.49048000
C -0.48105000 -1.13358800 2.32563600
C -1.88107200 -1.33994300 2.13882000
C -2.40780100 -2.65937500 2.12429100
C -1.51467500 -3.75460400 2.29101100
C 0.04806000 0.19123100 2.33321400
C -2.74968800 -0.22397700 1.94524300
C -2.21779900 1.10033100 1.93923500
C -0.82010900 1.30738700 2.14449800
C -3.07576900 2.21044300 1.70791600
C -4.46001700 1.97024500 1.47111300
C -4.97323800 0.69693900 1.49080400
C -4.13963100 -0.43298200 1.73102000
C -4.63942200 -1.76678000 1.72330300
C -3.80411600 -2.83860300 1.91383200
H -4.20266900 -3.84714500 1.87595800
H -5.69709900 -1.92503600 1.53767600
H 0.49891800 -4.40977700 2.57098600
H -1.91884500 -4.75923000 2.25362400
H -5.11237900 2.81460300 1.26609800
H -6.02860700 0.53429400 1.30043200
C 1.78061400 -2.00964800 2.65985100
C 2.28842800 -0.73559400 2.67659000
C 1.44175400 0.39815100 2.51862600
H 2.44422600 -2.86294500 2.76570800
H 3.35684800 -0.57567900 2.79061300
C 1.94232800 1.73018800 2.51874300
C 1.10802300 2.80422700 2.33354600
C -0.29034800 2.62535200 2.13661200
H 3.00998300 1.88339000 2.64918800
H 1.51119400 3.81298900 2.31483000
C -2.51460200 3.51958600 1.69393000
H -3.16623500 4.36362200 1.48733000
C -1.17370400 3.71766100 1.90434400
H -0.76017700 4.71984800 1.86048800

18. References

1. Fulmer, G. R., Miller, A. J. M., Sherden, N. H., Gottlieb, H. E., Nudelman, A., Stoltz, B. M., Bercaw, J. E. & Goldberg, K. I. NMR chemical shifts of trace impurities: common laboratory solvents, organics, and gases in deuterated solvents relevant to the organometallic chemist. *Organometallics* **29**, 2176-2179 (2010).
2. Molander, G. A., Trice, S. L. J., Kennedy, S. M., Dreher, S. D. & Tudge, M. T. Scope of the palladium-catalyzed aryl borylation utilizing bis-boronic acid. *J. Am. Chem. Soc.* **134**, 11667-11673 (2012).
3. Molander, G. A., Trice, S. L. J. & Tschaen, B. A modified procedure for the palladium catalyzed borylation/Suzuki-Miyaura cross-coupling of aryl and heteroaryl halides utilizing bis-boronic acid. *Tetrahedron* **71** , 5758-5764 (2015).
4. Wickramasinghe, L. D., Zhou, R., Zong, R., Vo, P., Gagnon, K. J. & Thummel, R. P. Iron complexes of square planar retradentate polypyridyl-type ligands as catalysts for water oxidation. *J. Am. Chem. Soc.* **137**,13260-13263 (2015).
5. Xu, Y., Yan, G., Ren, Z. & Dong, G. Diverse sp^3 C−H functionalization through alcohol β-sulfonyloxylation. *Nat. Chem.* **7**, 829-834 (2015).
6. Zhdanko, A. & Maier, M. E. Explanation of counterion effects in gold(I)-catalyzed hydroalkoxylation of alkynes. *ACS Catal.* **4**, 2770-2775 (2014).
7. http://supramolecular.org.
8. Thordarson, P. Determining association constants from titration experiments in supramolecular chemistry. *Chem. Soc. Rev.* **40**, 1305-1323 (2011).
9. Brynn Hibbert, D. & Thordarson, P, The death of the Job plot, transparency, open science and online tools, uncertainty estimation methods and other developments in supramolecular chemistry data analysis. *Chem. Commun.* **52**, 12792-12805 (2016).
10. Moser, A., Range, K. & York, D. M. Accurate proton affinity and gas-phase basicity values for molecules important in biocatalysis. *J. Phys. Chem. B* **114**, 13911-13921 (2010).
11. Jeziorska, M., Cencek, W., Patkowski, K., Jeziorski, B. & Szalewicz, K. Pair potential for helium from symmetry-adapted perturbation theory calculations and from supermolecular data. *J. Chem. Phys.* **127**, 124303 (2007).
12. Hohenstein, E. G., Parrish, R. M., Sherrill, C. D., Turney, J. M. & Schaefer, H. F., 3rd. Large-scale symmetry-adapted perturbation theory computations via density fitting and laplace transformation techniques: investigating the fundamental forces of DNA-intercalator interactions. *J. Chem. Phys.* **135**, 174107 (2011).
13. Contreras-Garcia, J., Johnson, E. R., Keinan, S., Chaudret, R., Piquemal, J. P., Beratan, D. N. & Yang, W. Nciplot: A program for plotting non-covalent interaction regions. *J. Chem. Theory. Comput.* **7**, 625-632 (2011).
